# Axially rigid steerable needle with compliant active tip control

**DOI:** 10.1371/journal.pone.0261089

**Published:** 2021-12-16

**Authors:** M. de Vries, J. Sikorski, S. Misra, J. J. van den Dobbelsteen

**Affiliations:** 1 Department of Biomechanical Engineering, Delft University of Technology, Delft, the Netherlands; 2 Department of Biomechanical Engineering, University of Twente, Enschede, the Netherlands; 3 Department of Biomedical Engineering, University of Groningen and University Medical Center Groningen, Groningen, the Netherlands; Tsinghua University, CHINA

## Abstract

Steerable instruments allow for precise access to deeply-seated targets while sparing sensitive tissues and avoiding anatomical structures. In this study we present a novel omnidirectional steerable instrument for prostate high-dose-rate (HDR) brachytherapy (BT). The instrument utilizes a needle with internal compliant mechanism, which enables distal tip steering through proximal instrument bending while retaining high axial and flexural rigidity. Finite element analysis evaluated the design and the prototype was validated in experiments involving tissue simulants and *ex-vivo* bovine tissue. Ultrasound (US) images were used to provide visualization and shape-reconstruction of the instrument during the insertions. In the experiments lateral tip steering up to 20 mm was found. Manually controlled active needle tip steering in inhomogeneous tissue simulants and *ex-vivo* tissue resulted in mean targeting errors of 1.4 mm and 2 mm in 3D position, respectively. The experiments show that steering response of the instrument is history-independent. The results indicate that the endpoint accuracy of the steerable instrument is similar to that of the conventional rigid HDR BT needle while adding the ability to steer along curved paths. Due to the design of the steerable needle sufficient axial and flexural rigidity is preserved to enable puncturing and path control within various heterogeneous tissues. The developed instrument has the potential to overcome problems currently unavoidable with conventional instruments, such as pubic arch interference in HDR BT, without major changes to the clinical workflow.

## 1. Introduction

Percutaneous needles are commonly used in minimally invasive diagnostics and therapeutic procedures. The trajectory and endpoint of the inserted needle influence the effectiveness of the procedure [1, 2]. Misplacement may cause misdiagnosis, additional tissue damage and less effective therapy outcomes [3–6]. One medical procedure that requires optimization is prostate brachytherapy (BT) [7, 8]. This technique employs ionizing radiation via ≤ 25 rigid implant needles under ultrasound (US) guidance to kill or stunt the growth of malignant tumorcells [9, 10]. Conventional high-dose-rate (HDR) BT needles are rigid and restricted to linear insertion paths so that deep-seated targets close to sensitive tissues or organs are challenging to reach. Physicians are challenged in needle placement by catheter displacement and deformation, tissue movement and deformation, needle deflection and imaging limitations [7, 11–18]. These transformations are a result of respiratory motion, movement of the patient, tissue anisotropy and inhomogeneity, anatomical structure interference, intermediate calcifications, edema and tissue compression and stretching [5, 13, 19–26]. To counteract these issues physicians are often limited to suboptimal correcting actions such as needle base or tissue manipulations [24, 27]. Reinsertion of the needle is in fact much required [5]. This induces additional tissue damage, postprocedural tissue swelling and increases patient discomfort and procedure time [5, 27–30]. Therefore, needle targeting demands improvement [11]. Another problem that arises with HDR BT needles is accessibility in large prostates. The Groupe Européen de Curiethérapie and the European SocieTy for Radiotherapy & Oncology (GEC-ESTRO) and American Brachytherapy Society (ABS) guidelines state that a prostate of respectively > 50 cubic centimetres (cc) and > 60 cc is technically more challenging [31]. They report this limit as a relative contraindication for prostate BT because of blockage of the anterolateral area of the prostate [24, 32–34]. This phenomenon, known as pubic arch interference (PAI), hampers a homogeneous irradiation of the whole gland and excludes a large patient group.

Steerable needles could play a part in correcting for needle bending, sparing sensitive tissues and avoiding anatomical structures, such as the pubic arch in prostate BT [30]. Multiple passive and active steering techniques have been proposed in academic literature and evaluated in experimental set-ups, such as pre-curved needles, cable-driven instruments or actuated needle tips [7, 21, 35–45]. On the contrary, only a few active steerable instruments have been commercialized: the Pakter Curved Needle Set (Cook Medical Inc., Bloomington, IN, USA), the Morrison Steerable Needle (AprioMed AB, Uppsala, Sweden), the Osseoflex SN Steerable Needle (Merit Medical Systems, South Jordan, UT, USA) and the Seeker Steerable Biopsy Needle (PneumRx, Mountain View, CA, USA) [27]. The last three instruments are cable driven and induce pivoting of the needle tip by cable pulling [4]. However, such designs have reduced axial and flexural rigidity and lack accurate control for penetrating stiffer tissue and membranes without buckling, thereby limiting the clinical applicability [46].

This work describes an omnidirectional steerable instrument that can be manually controlled where axial and flexural rigidity are preserved for increased controllability during needle insertions. The instrument is based on a single-piece compliant structure that enables distal tip steering through proximal instrument bending and is compatible with the existing approach for prostate HDR BT. We present the design of the steerable needle in section 2 which is evaluated with computational simulations in section 3. Steering performance of the prototype is assessed in US-guided experiments with tissue simulants and *ex-vivo* bovine tissue in section 4. This section demonstrates the ability to accurately steer the needle along curved paths and add value to HDR BT.

## 2. Needle design

**Fig 1:**
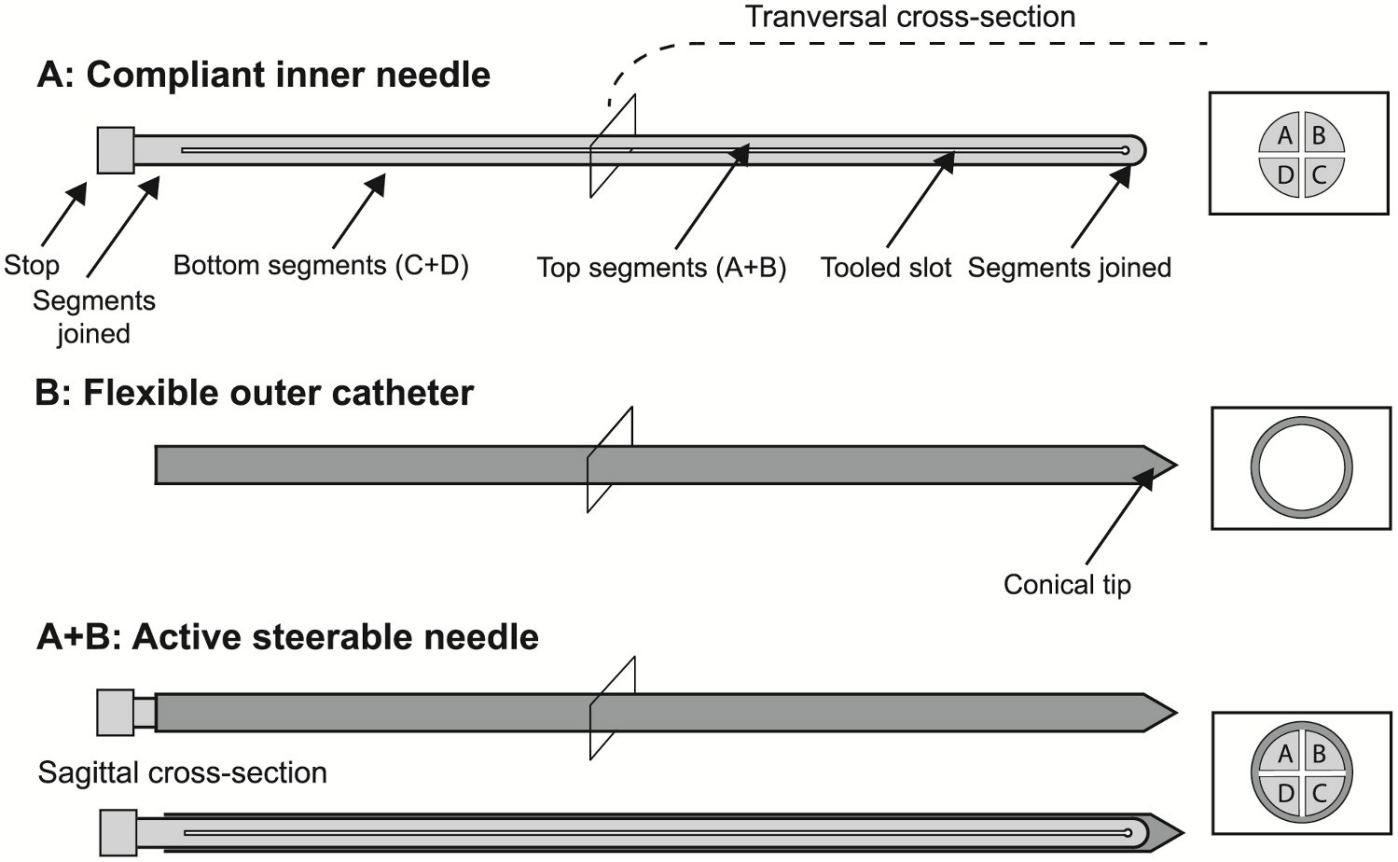
Schematic of the steerable needle assembly. (A) Inner needle. (B) Outer catheter. The assembly of (A) and (B) forms the active steerable needle (A+B). The boxes show the transversal cross-section of each part.

The instrument comprises a commercially available flexible outer catheter of polyamide (ProGuide^®^ sharp 6F needle, Elekta Instrument AB, Stockholm, Sweden) with a conical distal tip and a steerable patented steel inner needle (PWS140A, Eileen’s Emporium, Gloucester, United Kingdom) (Fig 1). The inner needle is a single-piece long slender rod, containing four parallel running segments, with a length of 205 mm and diameter of 1.40 mm.

**Fig 2:**
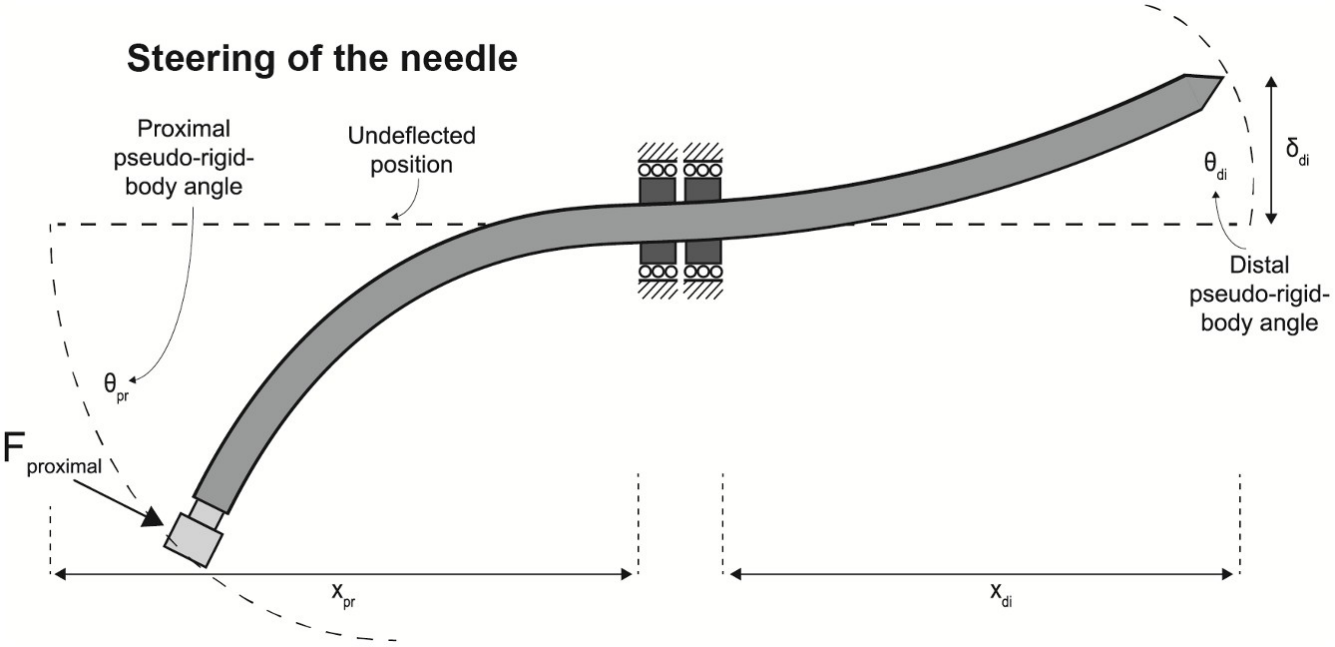
Schematic of needle steering. An applied force on the proximal end of the needle (F_proximal_) results in deflection of the distal end of the needle (δ_di_). The roller supports in the middle function as flexure bearings for needle deflection.

**Fig 3:**
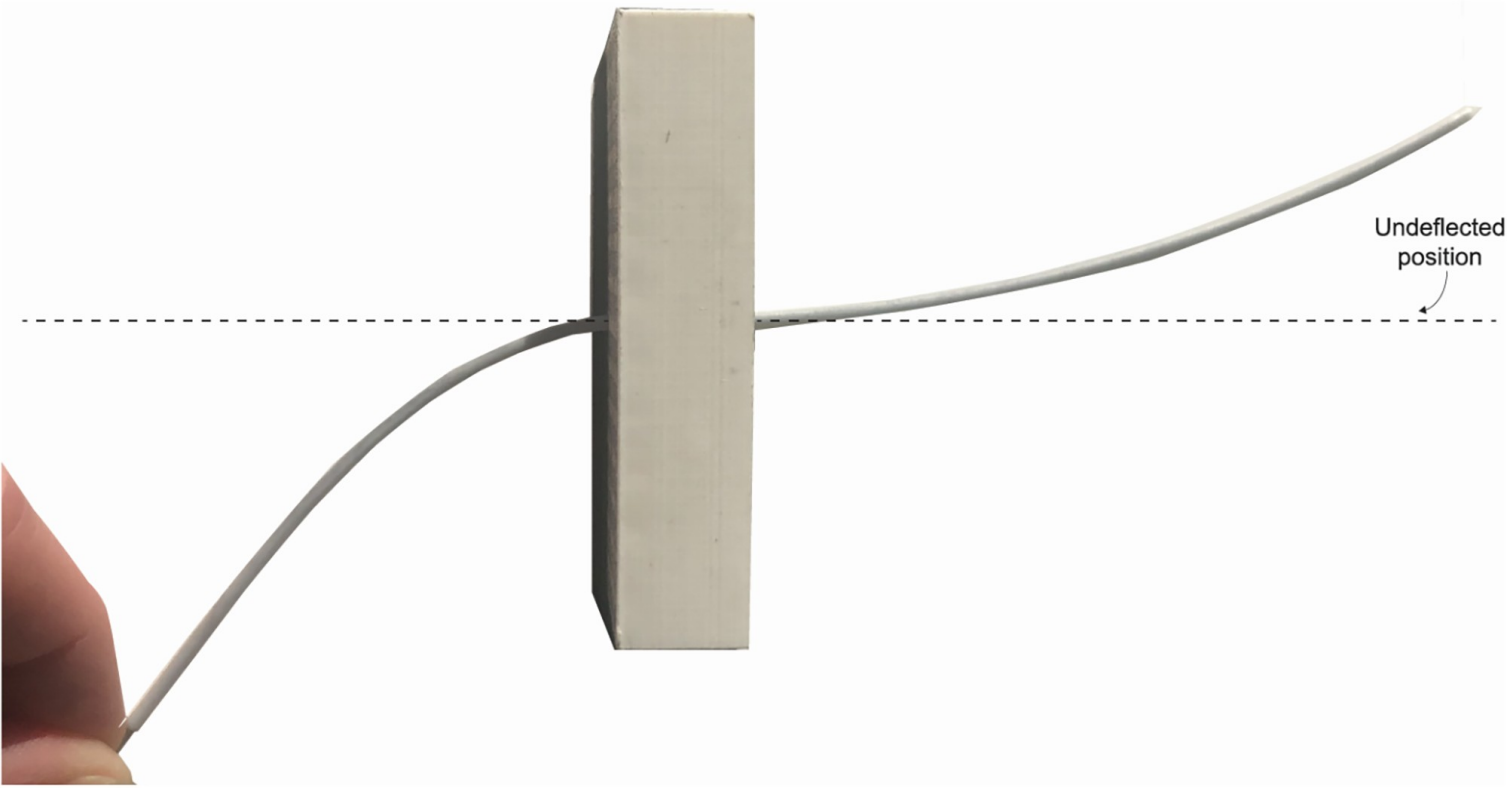
Needle steering with the manufactured prototype. An applied force on the proximal end of the needle results in deflection of the distal end of the needle. The needle guide in the middle functions as flexure bearings for needle deflection.

The inner needle contains a compliant mechanism as it transmits input forces at the proximal end to articulate the distal tip. A lateral input force induces instrument deflection which causes axial pushing and pulling of the segments. This topological synthesis is achieved by an electrical discharge machined slot of 0.12 mm over a length of 185 mm in both the sagittal and coronal plane, effectively splitting the rod in four equal quarters while leaving both ends of the needle joined. Simultaneous translation of the four segments in different ratios results in omnidirectional tip steering. The flexible catheter follows the deflections of the inner needle, which can be withdrawn from the instrument leaving behind a work channel with a lumen of 1.45 mm. Figs 2 and 3 show the schematic and actual use of the needle during steering, respectively.

**Fig 4:**
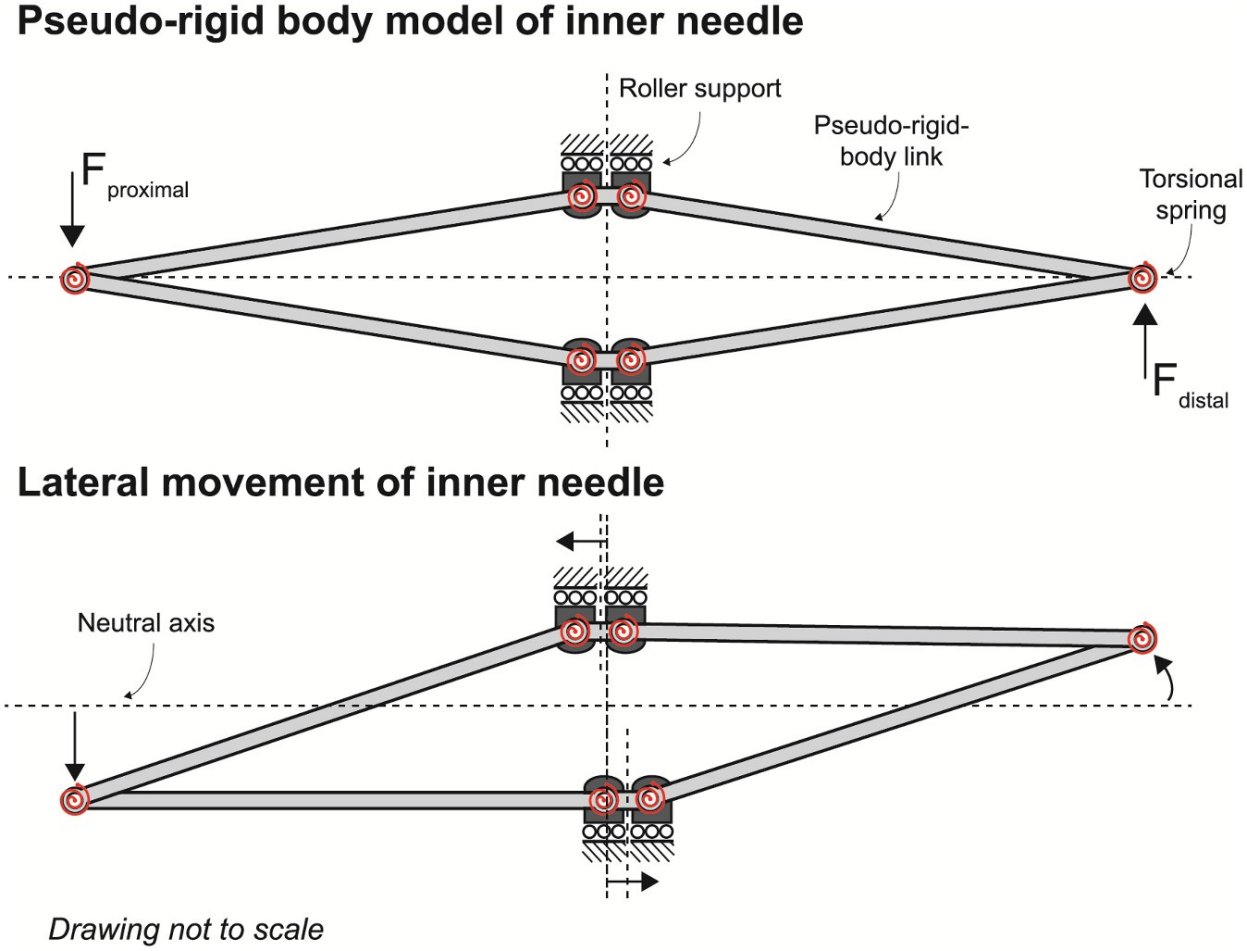
Pseudo-rigid body model of the inner needle. The inner needle is modelled with six pseudo-rigid-body links and six torsional springs. The horizontal segments and roller supports indicate the needle guiding. Downward movement of the proximal end results in axial pushing of the bottom segment and axial pulling of the upper segment resulting in upward movement of the distal tip.

The segments of the inner needle can be translated in axial direction inside the outer catheter while moving in the direction normal to its plane is restricted by the roller support. The roller support and design of the inner needle result in a flexure bearing and a compliant mechanism for the kinematic requirement of distal tip steering. The translation of segments with respect to each other is referred to as relative translation. The output deflection occurs in opposite direction to the input force, as the four segments move relative to each other during bending. This is indicated by the pseudo-rigid body (PRB) model in Fig 4.

An increase in the rotational degrees-of-freedom (DoF) from 0 for the conventional rigid inner needle to 2 for the novel steerable needle is accomplished by its design. This is at the expense of a decrease of 11.8% in principal moment of inertia for the cross-section illustrated in Fig 1A compared to a rigid inner needle. This results in a flexural rigidity (*E* * *I*) of 34.85 * 10^−3^ (*N* * m^2^) for the steerable inner needle, where *E* = 205 *Gpa* (CES EduPack 2019, Granta Design Limited, Cambridge, United Kingdom) and *I = 0*.*17 mm*^*4*^. The axial rigidity (*E* * *A*) of the inner needle reflects the ability to resist axial loads. A decrease in area from 1.54 to 1.22 mm^2^ results in a 20.8% reduction in axial rigidity.

## 3. Computational simulation

### 3.1. Finite element model

A static finite element analysis in ABAQUS/CAE 2017 (Simulia, Johnston, RI, USA) evaluates the configuration of the inner needle, assesses theoretical maximum stress and deflection and relates input bending and output steering outside of a medium. Table 1 shows the properties of the inner needle and outer catheter used in the finite element model. Fig 2 shows the critical dimensions used in the analysis.

**Table 1:** Properties of the finite element model.

**Outer catheter**	Length	200 mm
	Outer diameter	2.0 mm
	Inner diameter	1.5 mm
	Young’s modulus	1 GPa
	Simplification	Two open ends
**Inner needle**	Length	208 mm
	Outer diameter	1.4 mm
	Young’s modulus	205 GPa
	Simplification	1 slot over 185 mm
		No starting hole for wire-EDM
		No proximal stop
**Needle guide**	Length	10 mm
	Simplification	Modelled as roller boundary condition

The outer catheter is modelled as a homogenous shell with two open ends and linear quadratic S4R nodes. The inner needle contains one slot over 185 mm, splitting the needle into two halves. The inner needle contains linear hexahedral shaped elements (C3D8R nodes) on which 3D stress analysis is performed. The needle guide is modelled as a roller boundary condition constraining shift and rotation of the outer catheter. Input bending around the Z-axis is allowed over the whole proximal needle length up to the needle guide. Distal tip angle (θ_di_) and deflection (δ_di_) are measured for multiple PRB angles (θ_pr_: 10°, 30°, 50°, 70°, 90°) and multiple proximal needle lengths (X_pr_: 20 mm, 50 mm, 80 mm, 110 mm). Relative translation between the upper and lower segment is measured on predefined nodes in the centre of the roller support.

### 3.2. Finite element analysis

**Fig 5:**
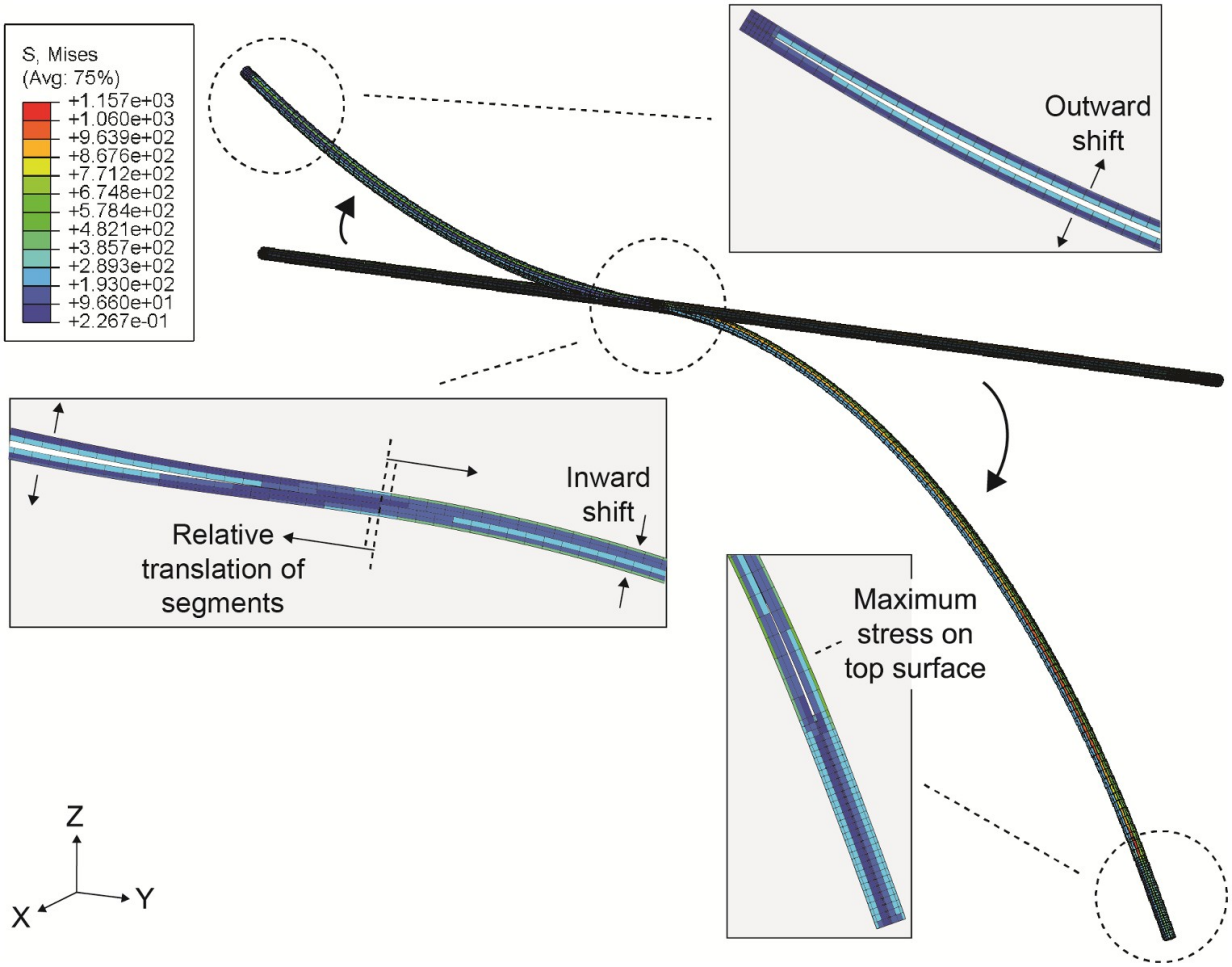
The finite element model of the active steerable needle in normal and bent condition. The boxes show the configuration of the inner needle during bending. The arrows indicate the direction of the shift of the segments. Stress and bending apply for the computational model with X_pr_ = 120 mm and θ_pr_ = 70°.

Geometric chances occur in the model during bending. Fig 5 shows the computational model of the inner needle in straight and bent condition. Simultaneous pushing and pulling the upper and lower segment result in a relative translation causing needle steering. Outward shift of the segments from the neutral line is restricted by the catheter, however inward shift is probable. Maximum stresses during downward bending are found at the top surface on the proximal end of the upper segment.

**Fig 6:**
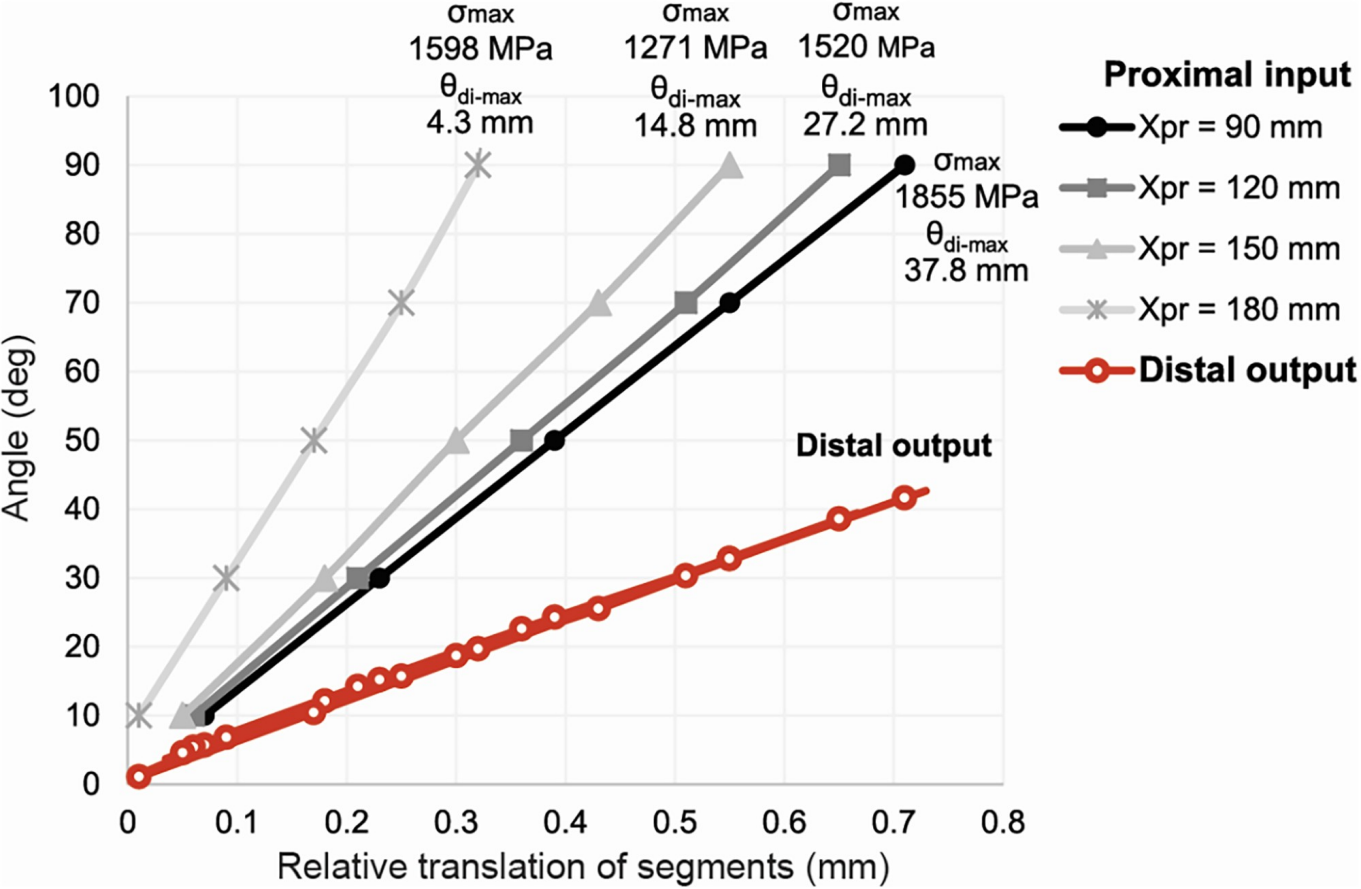
The relationship between proximal input angle and relative translation between segments for different proximal needle lengths. The red line indicates the distal output. Markers on the distal output line relate to the vertically aligned markers on the input lines. Maximum stress and deflection is described for all proximal inputs.

Fig 6 gives an overview of the proximal PRB input angle and the corresponding relative translation of the segments. A shorter proximal needle length increases distal tip angle and deflection while affecting maximum stress. Maximum stress (1855 MPa), maximum distal tip deflection (37.8 mm) and maximum distal angle (41.6°) are found for the sharpest angle (90°). This indicates that the steerable needle stays below the elastic limit (~ 2700–3300 MPa) of the patented steel material (CES EduPack 2019, Granta Design Limited, Cambridge, United Kingdom). This information shows us the feasibility of the steerable instrument.

## 4. Experimental evaluation

A prototype is manufactured in order to translate the use of the steerable instrument into practice. Three validation experiments determine steering characteristics in soft tissue simulants and *ex-vivo* bovine tissue using US imaging.

### 4.1. Experiment 1—Fixed-bent needle steering

The influence of the initial insertion depth on needle steering and the endpoint precision are assessed inside a homogeneous medium for set curvatures.

#### 4.1.1. Materials and method

*4*.*1*.*1*.*1*. *Set-up and procedure*. A porcine gelatin tissue simulant (Gelatine, Dr. Oetker, Bielefeld, Germany) of 10 wt.% at room temperature (~20°C) and a mould with predefined curvatures to ensure reproducibility of the bending are used. The tissue simulant is confined within in a transparent Poly (methyl methacrylate) (PMMA) container (200 x 112 x 112 mm). The front plate of the container (thickness: 12 mm) contains holes (⌀ 2.1 mm) functioning as a needle guide. Rectangular compartments in the front face of the tissue simulant are filled with air. These compartments are located above every insertion hole and function as reference for coordinate <0,0,0> on the US images. The mould is attached to a linear slide which allows for controlled translation. The slide can be lifted in X-direction using blocks and the container can be moved in Y-direction for a new puncture. The needle is inserted in the tissue simulant for 20 mm in a straight orientation to overcome puncture and cutting forces. After every insertion, US imaging with the Robotic Utrasound System (RobUSt) [47] is used with a sweep over two lengths of the tissue simulant. Dependence of steering performance on insertion depth is evaluated for two, initially straight, insertion paths (d_straight_ = 0 mm and d_straight_ = 40 mm). Two different proximal bending angles (slight angle: 22.5° and sharp angle: 45°) are applied to determine steering capability over a length of 100 mm.

**Fig 7:**
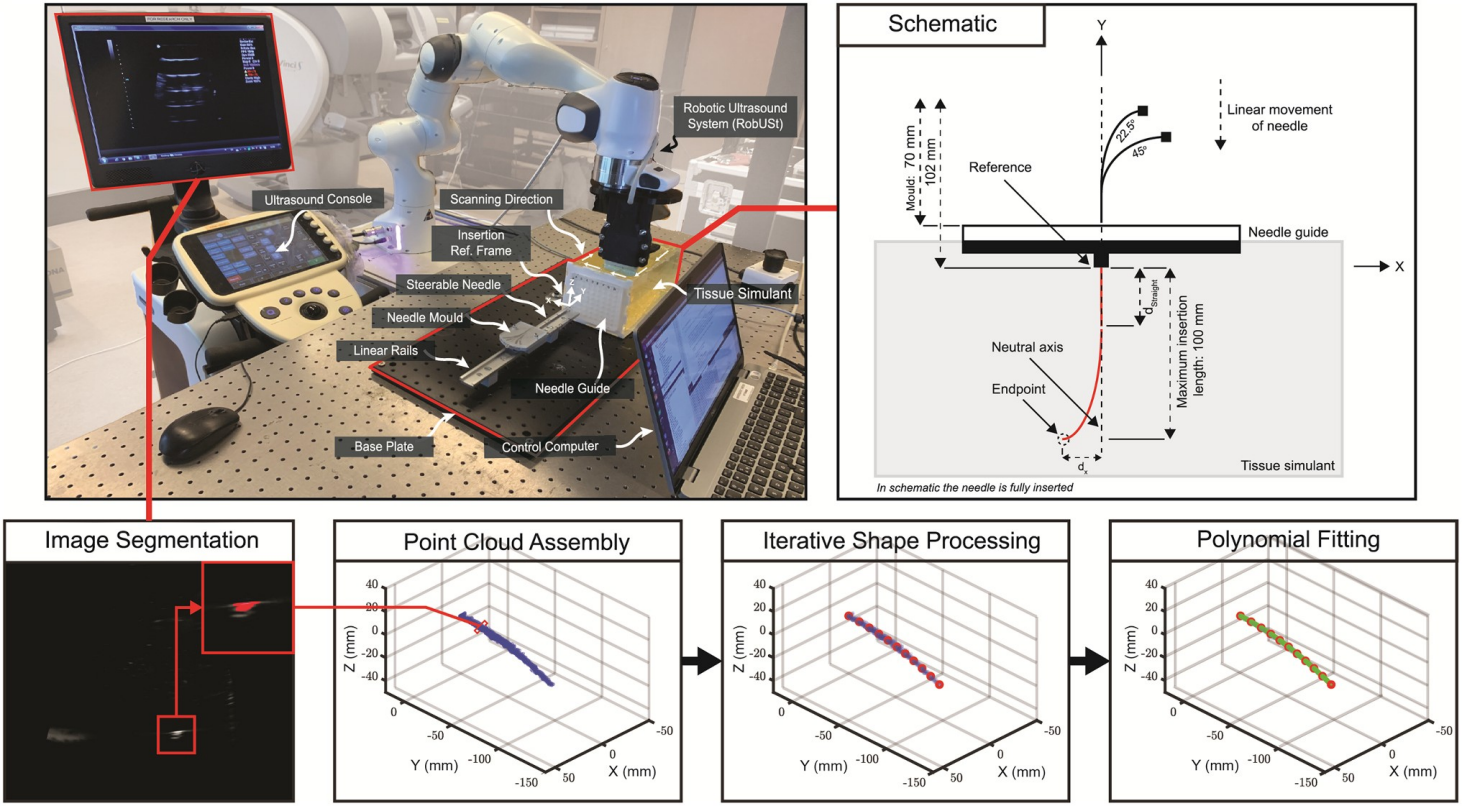
Set-up and pipeline for shape reconstruction of the fixed-bent needle steering experiment. The Robotic Ultrasound System (RobUSt) is used for shape reconstruction of the steerable instrument. For each insertion, RobUSt performs a volumetric scan of the tissue simulant, recording US images along with corresponding transducer pose data, expressed in the global reference frame at the base of the robotic manipulator. The silhouette of the instrument is segmented from each frame. All segmented data are assembled in a point cloud. The point cloud is processed using an iterative shape reconstruction algorithm, described in detail in Suligoj et al. [47]. The iterative shape algorithm first generates a series of points along the instrument (red dots). These points are used to fit a third order polynomial (green) describing the continuous shape of the instrument.

*4*.*1*.*1*.*2*. *Image processing and analysis*. Fig 7 shows a schematic of the experimental set-up and the process of 3D needle shape reconstruction using RobUSt, which comprises of a Sonix Touch L14-5/38 (BK Medical, Peabody, MA, USA) transducer mounted on a Panda (Franka Emika GmbH, Munich, Germany) robotic arm. RobUSt is used to scan a predefined volume of the tissue simulant. During the scan, 2D US images are recorded at 20 Hz, along with the corresponding information about the transducer pose expressed in the RobUSt reference frame. The set of US images is segmented using constant intensity thresholding to create a point cloud containing the silhouette of the instrument. Each silhouette is processed by a shape reconstruction algorithm that extracts a continuous shape of the needle described by a 3^rd^ order polynomial with coefficients $\left(A_{i},B_{i},\;C_{i},\;D_{i}\in R^{3}\right)$ and length $\left(l_{i}\in R^{+}\right)$ [48]. The location $\left(p_{i}(s)\in R^{3}\right)$ of a point lying on the shape at the distance (*s* ∈ [0; *l*_*i*_]) can be calculated from polynomial coefficients as:

$$p_{i}\left(s\right)=A_{i}+{sB}_{i}+{s^{2}C}_{i}+{s^{3}D}_{i}=\left[I_{3}\;{\;I}_{3}s\;I_{3}s^{2}\;I_{3}s^{3}\right]\left[\begin{array}{c} A_{i}\\ B_{i}\\ C_{i}\\ D_{i} \end{array}\right]$$ (1)

where *I*_3_ is the 3x3 identity matrix.

The last point on the reconstructed 3D needle shape in the XY-plane determines the achieved lateral needle steering. The uncontrolled needle deflection at the distal end is assessed in XZ-plane. The trials of the same condition are averaged and standard deviation (± σ) is reported.

#### 4.1.2. Results

Table 2 gives an overview of steering for all experimental conditions (EC’s). The results show that an increased bending input at the proximal end enlarges the angle for lateral tip steering in the tissue simulant what is expected from section 3. Maximum fixed-bent needle steering of 20.2 mm laterally over a distance of 97.7 mm is found. In Z-direction an unwanted error occurred in all trials which increased with insertion depth.

**Table 2:** Steering capability of the steerable needle in the fixed-bent needle steering experiment.

N = 33		Proximal bending angle		
			22.5°	45°
**d**_**straight**_	**0 mm** Mean.error (mm) ± σ		**EC1**	**EC3**
		*n*	*8*	*9*
		Y (length)	0.5 ± 0.2	1.7 ± 1.7
		X (steering)	9.3 ± 1.5	17.3 ± 1.7
		Z (error)	-3.9 ± 0.9	-3.8 ± 4.3
	**40 mm** Mean.error (mm) ± σ		**EC2**	**EC4**
		*n*	*8*	*8*
		Y (length)	0.2 ± 0.2	1.2 ± 0.7
		X (steering)	2.6 ± 0.9	8.7 ± 1.1
		Z (error)	-2.7 ± 1.3	-4.9 ± 1.5

The mean error ± standard deviation (± σ) in X,- Y,- and Z-direction for two different proximal bending angles and two initial straight insertion depths in a homogeneous tissue simulant.

**Fig 8:**
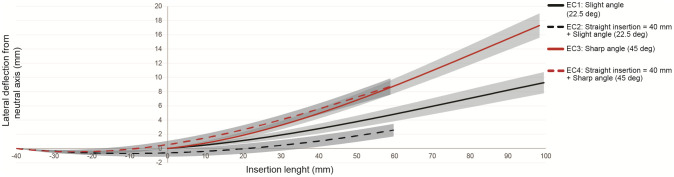
Lateral deflection of the steerable needle over insertion length in the fixed-bent needle steering experiment. The mean error ± σ in lateral direction for two different proximal bending angles and two initial straight insertion depths. The curves characterized by steering after 40 mm are superimposed on the curves of steering from <0,0,0> to determine what the influence of the initial depth is on needle steering.

Fig 8 shows smooth curvatures for all conditions. Superimposing the needle trajectories of EC2 over EC1 and EC4 over EC3 indicate that steering response of the needle does not depend on the initial insertion depth. A mean distal tip error of 2.2 mm and 0.1 mm were respectively obtained. Noteworthy is that uncontrolled absolute deflections of 1.2 mm and 2.0 mm from the neutral axis were already observed after 40 mm for EC2 and EC4.

### 4.2. Experiment 2—Clinical use case: Active needle tip steering in prostate tissue simulant

In prostate BT, rigid needles are inserted over approximately 90 mm into the prostate gland for internal irradiation using transrectal US for real-time visualization [49]. In this experiment, manual active steering and real-time needle guidance via 2D US imaging evaluates endpoint accuracy in inhomogeneous tissue simulants. The accuracy is assessed over a maximum lateral distance of 15 mm. Straight needle insertions with the steerable inner needle and a commercially available rigid HDR BT inner needle (ProGuide^®^ Obturator, Elekta Instrument AB, Stockholm, Sweden) are performed to place the use of steering in context.

#### 4.2.1. Materials and method

**Fig 9:**
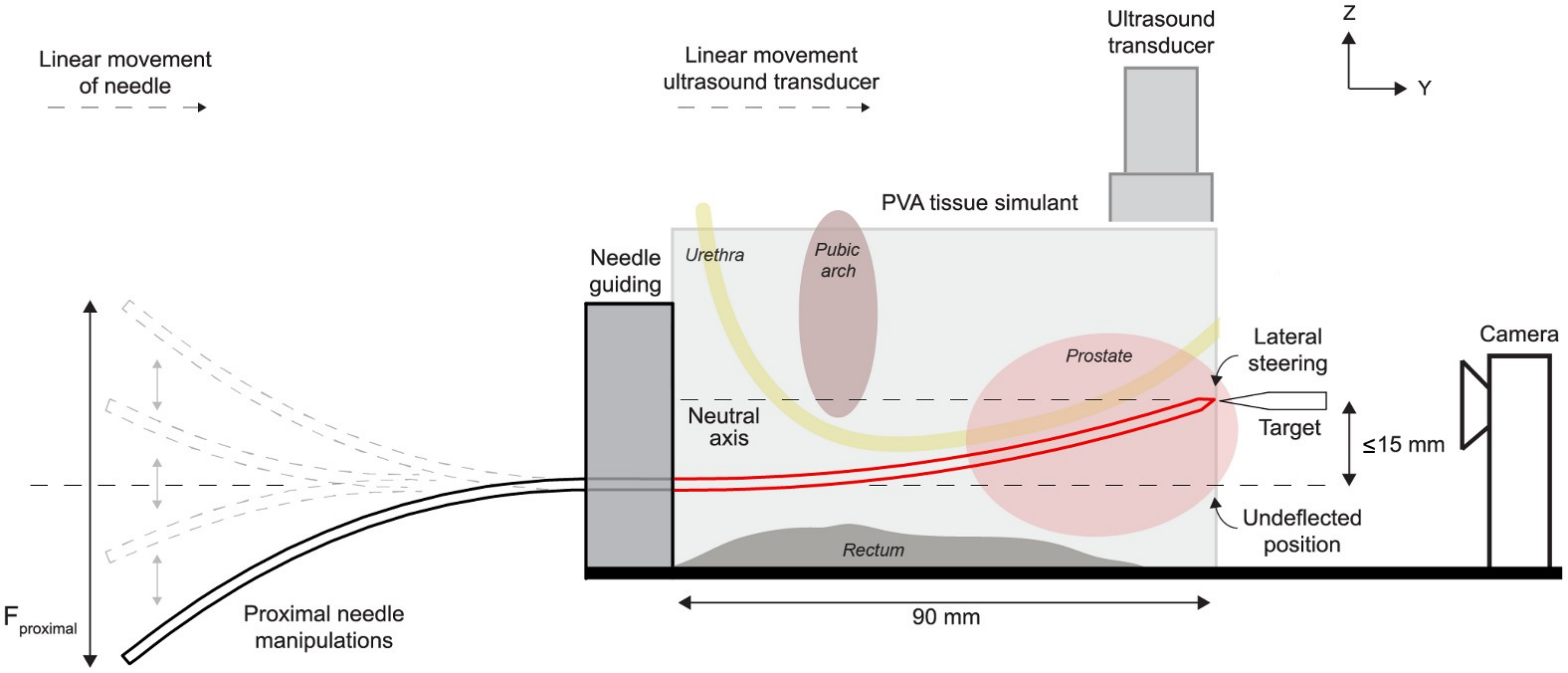
Set-up of active needle steering experiment in prostate tissue simulant. Proximal needle manipulations in Z-direction allow for steering at the distal tip. Insertion of the needle in the prostate tissue simulant and movement of the US transducer is performed bimanually. The camera visualizes the back surface of the tissue simulant in transversal plane. The prostate, pubic arch, urethra and rectum are illustrated in the figure in sagittal plane to demonstrate the clinical applicability of the steerable needle for prostate brachytherapy. The pubic arch and urethra are circumvented to reach occluded prostate tissue.

*4*.*2*.*1*.*1*. *Set-up*. 2D US imaging (Phillips HD7 XE) is used for both needle guidance and definition of the target site located at the distal surface of the tissue simulant illustrated in Fig 9. The target is a horizontal PETG wedge mounted on a slide. Images are taken after every puncture with a Dino-Lite Digital PC-Microscope (AM73915MZTL 5 Megapixel, 10–140x) fixated in a vertical assembly. Both are aligned with the neutral axis.

*4*.*2*.*1*.*2*. *Tissue simulant design*. Two tissue-mimicking blocks (350 x 130 x 90 mm) of poly(vinyl alcohol) (PVA) are manufactured. One soft tissue simulant approaches the Young’s modulus of muscular and adipose tissue (~ 15 kPa) [50]. One stiff tissue simulant mimics the Young’s modulus of prostatic tissue (~ 50 kPa) [51]. As stiffness of the phantom can influence needle deflection, needle steering is performed in both tissue simulants [52].

PVA is a synthetic polymer that can mimic mechanical properties of human tissue by performing freeze-thaw cycles (FTC’s) and is suitable for US visualization [53–55]. These FTC’s create a polyvinyl alcohol cryogen (PVA-C) due to polymer cross-linking, used in earlier research as a simulant for prostate tissue [56]. Within this study the soft and stiff tissue simulants are made with 10 wt.% PVA-C with 1 and 5 FTC’s, respectively. The Young’s modulus of the tissue simulant was evaluated by a compression test. The PVA powder (SELVOL^™^, Sekisui Specialty Chemicals America, Dallas, Texas) is dissolved in a 60:40 mixture of water and coolant (Talamex, Lankhorst Taselaar B.V., Heerenveen, The Netherlands) to prevent expansion of the volume.

*4*.*2*.*1*.*3*. *Procedure*. Manual active steering towards a target is assessed. The experimenter bimanually controls the steerable needle and the US transducer for visualization of the tip position and the target location in sagittal plane. When reaching the distal wall of the PVA block the target is removed and the needle is punctured out of the tissue simulant to define the end-location of the needle tip and error in Z-direction with the Dino-Lite Digital PC-Microscope. Additionally, needle insertions without steering and with the ProGuide^®^ Obturator are performed. The endpoint accuracy of the needle is evaluated in fourteen experimental conditions, described in section 4.2.2. All experimental conditions are randomized for each tissue simulant of ~20°. First, all insertions in the soft tissue simulant are performed followed by insertions in the stiff tissue simulant.

*4*.*2*.*1*.*4*. *Data and statistical analysis*. The mean absolute errors ± σ are evaluated for all experimental conditions and statistical analysis (IBM SPSS Statistics 25) is performed to investigate performance of the developed steerable needle. To verify the non-normal error distribution a Shapiro-Wilk’s test, the normal Q-Q plots and a visual inspection of their histograms are performed. If normality is found, a one-way ANOVA is done and Tukey’s HSD procedure evaluates the differences between conditions. Unequal sample sizes are analysed in a Games-Howell test. A p-value < .05 is considered statistically significant.

#### 4.2.2. Results

Table 3 shows the mean absolute errors ± σ of all experimental conditions for both the adipose tissue simulant and the prostatic tissue simulant. Active steering (EC1—EC5) results in a mean endpoint error of 1.44 mm.

**Table 3:** The endpoint errors of the steerable needle and the reference needle per tissue simulant.

N = 200		Active steering					No steering	*ProGuide*^*®*^ *Obturator*
		*lateral*		*lateral out-of-path*		*neutral axis*	*neutral axis*	*reference*
		EC1	EC2	EC3	EC4	EC5	EC6	*EC7*
		7.5 mm	15 mm	7.5 mm	15 mm			
**Adipose tissue simulant (soft)**	*n*	*10*	*10*	*10*	*10*	*20*	*20*	*20*
	| Mean absolute error (mm) | ± σ	1.52	2.45	1.19	1.59	1.45	2.42	*2*.*01*
		±	±	±	±	±	±	±
		0.93	1.70	0.80	1.12	0.83	0.82	*0*.*87*
**Prostatic tissue simulant (stiff)**	*n*	*10*	*10*	*10*	*10*	*20*	*20*	*20*
	| Mean absolute error (mm) | ± σ	1.27	1.64	1.23	0.91	1.15	6.86	*2*.*03*
		±	±	±	±	±	±	±
		1.13	1.40	0.78	1.14	0.83	1.74	*0*.*62*
**Total**		1.39	2.05	1.21	1.25	1.30	4.64	*2*.*02*
		±	±	±	±	±	±	±
		1.02	1.57	0.77	1.16	0.84	2.62	*0*.*75*

The endpoint error is measured after every puncture showing the mean absolute error and standard deviation (± σ) in millimeters per experimental condition. The rigid ProGuide^®^ Obturator is used as reference. In EC1, EC2 and EC5 active needle steering is allowed over the entire insertion length. In EC3 and EC4 the needle is first inserted without steering over 90 mm and then withdrawn for 50 mm. Subsequently, the needle is actively steered out of the created needle path towards the target. In EC6 the steerable needle is inserted over 90 mm without active steering. In EC7 the rigid ProGuide^®^ Obturator is inserted. The absence of US guidance for EC6 and EC7 allows for determination of the Euclidean distance between the endpoint of the needle tip and the neutral axis.

A Kruskal-Wallis test showed no statistically significant difference in obtained error for the experimental conditions related to active lateral needle steering (EC1 –EC4) in both soft tissue simulants. A one-way ANOVA analysis yielded statistically significant variation between the normally distributed EC5, EC6 and EC7 for both the low stiffness tissue simulant *F*(2,57) = 6.612, *p* = .003 and the high stiffness tissue simulant *F*(2,57) = 138.500, *p* < .001. A post hoc Tukey test showed that the reference group with the conventional rigid ProGuide^®^ Obturator (EC7) did not differ significantly from the group with active steering on the neutral axis (EC5) in both tissue simulants. EC6 differed significantly from EC5 (*p* = .002) in the soft tissue simulant and from both EC5 (*p* < .001) and EC7 (*p* < .001) in the stiff tissue simulant.

The Games-Howell post hoc test indicated no significant differences in endpoint accuracy in the adipose tissue simulant between the steerable needle and the ProGuide^®^ Obturator. In the prostatic tissue simulant the actively steered needle on the neutral axis (M = 1.15, σ = .83) had a statistically significant smaller error than the conventional rigid HDR BT inner needle (M = 2.03, σ = .62, *p* = .009). Noteworthy is that no steering with the steerable needle resulted in significant differences from EC3 (*p* = .014) and EC5 (*p* = .012) in the adipose tissue simulant and in statistically significant variation from all experimental conditions in the prostatic tissue simulant (*p* < .001).

### 4.3 Experiment 3—Active needle tip steering in *ex-vivo* tissue

Manual active needle tip steering towards targets is executed in *ex-vivo* bovine tissue with real-time US needle tracking. This experiment evaluates adaptive needle path control during insertions and final endpoint accuracy in a more challenging medium.

#### 4.3.1. Materials and method

*4*.*3*.*1*.*1*. *Set-up and procedure*. The PMMA container and the RobUSt system are used in this set-up. The container holds inhomogeneous bovine tissue (Blade steak, Benali, Rotterdam, The Netherlands) embedded in 20 wt.% porcine gelatin (Gelatine, Dr. Oetker, Bielefeld, Germany). On each trial, the steerable needle is inserted through a layer of gelatin into the bovine tissue reaching the initial needle position. Omnidirectional needle steering is allowed from this position over 90 mm towards the target (⌀ 0.5 mm). Continuous back and forth US scanning in Y-direction over the top surface of the tissue is executed to determine location of the needle tip and target in transversal plane. A cursor is used to locate the target when the US transducer is in the same plane.

**Fig 10:**
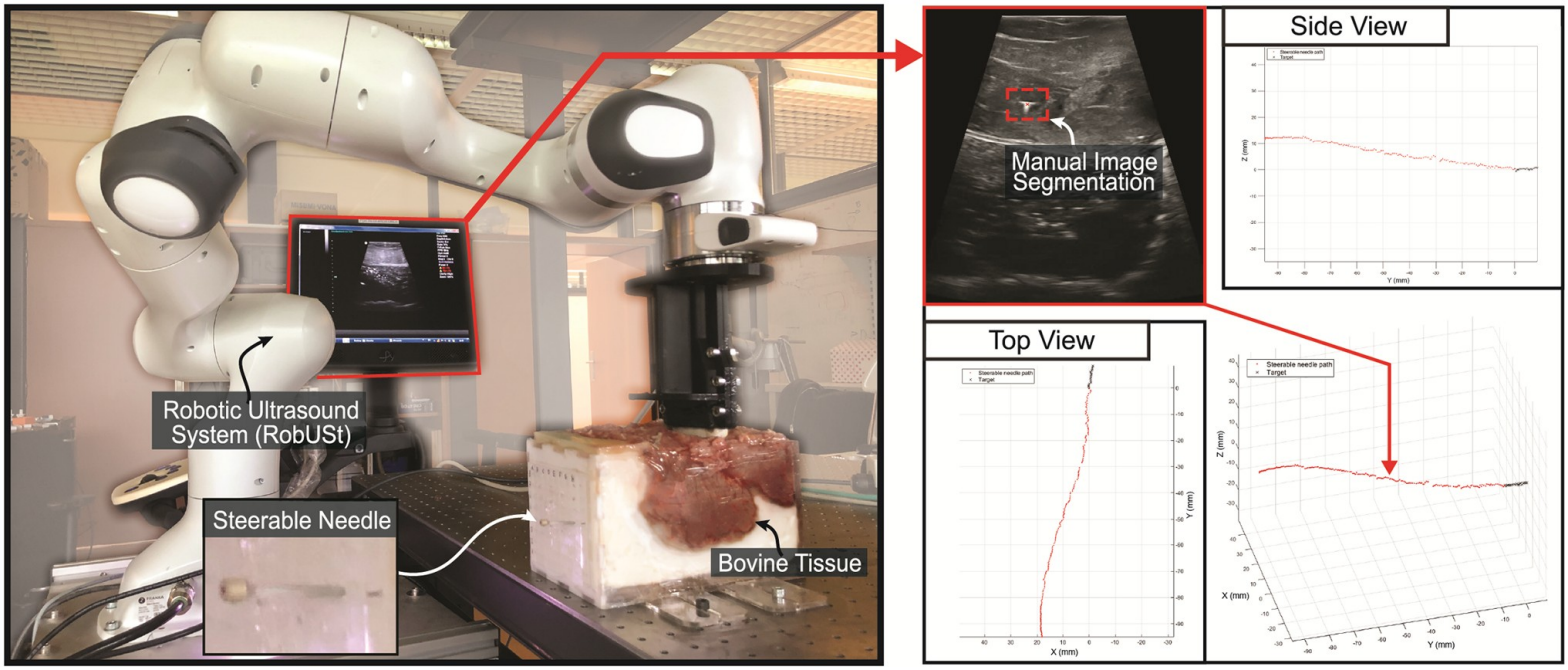
Set-up of active needle steering experiment in *ex-vivo* tissue. The trajectory of the steerable needle (red) over a length of 90 mm towards a target (black) located laterally from the neutral axis. Control of the steerable needle was performed in 3D. Segmentation of the needle and target from the US images are used for analysis.

*4*.*3*.*1*.*2*. *Image processing and data analysis*. Needle tracking inside the *ex-vivo* tissue is performed using RobUSt in a fashion similar to section 4.1.1. Nevertheless, due to environmental clutter within the bovine tissue the silhouette segmentation of the needle and target is performed manually for each image without employing the polynomial reconstruction. The segmented shapes are used to identify the position of the tip of the instrument with respect to the target. Fig 10 shows the set-up of the experiment and the trajectory of the needle towards the target for one of the trials.

#### 4.3.2. Results

Needle steering in X,- and Z-direction towards the targets was required over distances ranging from 3.3 to 17.3 mm and 0.9 to 11.8 mm, respectively. Steering towards the target over a 90 mm insertion depth resulted in a 3D error of 2.0 ± 2.6 mm and a 2D error in the plane of the target of 0.9 ± 2.0 mm. The 2D error did not consider the error in Y-direction as insertion depth was a set value.

## 5. Discussion

This work presents a novel omnidirectional steerable instrument for prostate HDR BT. The instrument comprises a commercially available outer catheter and a compliant steerable inner needle, which replaces the conventional rigid non-steerable inner needle currently used in HDR BT. Steering of the inner needle allows for adjustments of the catheter pathway and withdrawal of the inner needle creates a work channel for the medical procedure. Thus, the clinician can overcome pubic arch interference and correct for deflections to obtain a homogeneous dose distribution.

To the authors’ best knowledge, this is the first steerable needle whereby active tip control is achieved by exploiting structural mechanics, without the use of external actuation means such as magnetic forces, optical heating, robotic actuation or a joystick at the proximal end. The internal compliant mechanism enables distal tip steering with two rotational DoF through proximal needle bending. A reduction in flexural and axial rigidity of 11.8% and 20.8% is observed respectively compared to a rigid inner needle. However, the flexural rigidity of the proposed design is much higher than for the designs reported in literature [42, 46]. Moreover, no buckling was observed in our prostate tissue mimicking and *ex-vivo* experiments, suggesting that the design has sufficient axial rigidity for placement of HDR BT catheters.

The computational model showed a linear relation between input angle and relative translation of the segments associated with distal tip deflection. Validation experiments demonstrated needle steering up to 20 mm and controllability of the prototype in tissue simulants and in *ex-vivo* tissue. The fixed-bent needle steering experiment showed that steering in tissue was history-independent. The clinical use case experiment related to prostate brachytherapy showed high endpoint accuracy of the steerable needle in a soft and stiff inhomogeneous tissue simulant. These errors were comparable to those of a rigid HDR BT needle. The results of the *ex-vivo* experiment indicated that path corrections were feasible in a more challenging medium and off axis targets can be accurately reached following curved needle paths.

The results from the finite element analysis demonstrate that the inner needle stays in the elastic region of the material and will return to its initial position after removal of the applied input. It should be noted that the computational model is a simplification of the developed needle. The input is modelled as a circular deflection around the X-axis, while in real practice a lateral force results in a more hyperbolic shape at the proximal needle end. This input, approached in Exp. 1, is more representative for the final clinical application as the circular curvature is hard to obtain and workspace outside the body can be limited. The friction coefficient was kept constant while the simulation indicates that segments are translating over each other. Therefore, friction can play a role in the relationship between proximal input and distal output. This aspect is not addressed in this study.

The fixed-bent needle steering experiment demonstrated steering regardless the initial straight insertion depth due to articulations at the distal tip. This is a distinct advantage over non-holonomic asymmetric tip needles because their steering solely depends on needle-tissue interaction and pushing the needle through tissue is required to generate needle deflection. Small differences in lateral steering were found for the slight angle in Exp. 1 as spontaneous deflections in the opposite direction occurred over the initial straight insertion depth and insertion speed was not kept constant. In addition, uncontrolled deflection increased with insertion depth. This spontaneous deflection can be a consequence of the decreased flexural rigidity of the needle. Robotic control could counter this error but requires feedback control via imaging, electromagnetic tracking or shape sensing. We compensate for the error by steering manually. This affects medical device management, costs and clinical workflow to a lower extent than teleoperation.

Earlier studies reported needle placement errors up to 3 mm in a prostate and 8 mm in a prostate phantom for conical tip and bevel tip brachytherapy needles, respectively [5, 20]. A 3 mm error together with the 5 mm perineal template grid could theoretically cause a catheter void of 11 mm in very extreme cases [57]. In practice, rigid brachytherapy needle withdrawal and reinsertion are often required [5]. The steerable needle do not have to be fully withdrawn for error compensation which potentially reduces tissue trauma. The steerable needle can be partially withdrawn and steering out of the initial path allows for targeting with high accuracy indicated by EC3 and EC4 in Exp. 2.

In the clinical use case experiment we found similar endpoint errors for the rigid ProGuide^®^ Obturator in 2D and the steerable inner needle in one direction regardless the degree of active steering. But, results indicated that active steering is essential as its absence decreased the endpoint accuracy. In *ex-vivo* tissue the steerable needle obtained a similar error in 3D and lower error in 2D as compared to the ProGuide^®^ Obturator in 2D in Exp. 2. In conclusion, the endpoint error of the developed steerable needle stays below the mean acceptable error of 2.7 mm for targeted lesions reported after a questionnaire under 125 interventional radiologists [11].

The bovine tissue of Exp. 3 contained high amounts of connective tissue which complicated smooth insertions. Besides, it was a challenge to control needle steering in two planes during insertion with visual feedback processing. Small needle retractions were therefore allowed in all trials. We found that the axial and flexural rigidity of the developed steerable needle enables puncturing and path control in inhomogeneous tissue simulants and in *ex-vivo* bovine tissue. Furthermore, the developed steerable needle is compatible with currently applied devices in HDR BT and may improve needle targeting without major changes to the clinical workflow.

## 6. Conclusion

A novel axially rigid steerable instrument for HDR BT was developed and validated in this work. Manual omnidirectional steering is possible with the aid of a compliant mechanism. Validation experiments show high targeting accuracy in tissue simulants and *ex-vivo* tissue while visualization of the needle is possible with US. The developed steerable needle has the ability to steer along curved paths preserving its axial and flexural rigidity. The needle has the potential to add value to medical procedures currently performed with rigid needles and enlarge the patient group eligible for prostate HDR BT.

## Supporting information

S1 File Exp. 1—Raw data. (ZIP)

S2 File Exp. 2—Raw data of soft phantom. (ZIP)

S3 File Exp. 2—Raw data of stiff phantom. (ZIP)

S4 File Exp. 3—Raw data of insertion 1–6. (ZIP)

S5 File Exp. 3—Raw data of insertion 7–11 & overview. (ZIP)

S6 File FEA video of steerable needle. (ZIP)
