# Supplementary figures and images for: Axially rigid steerable needle with compliant active tip control (part 1 of 2)
Source: PLoS One. 2021 Dec 16;16(12):e0261089. doi: 10.1371/journal.pone.0261089 (PMC8675730; doi:10.1371/journal.pone.0261089)

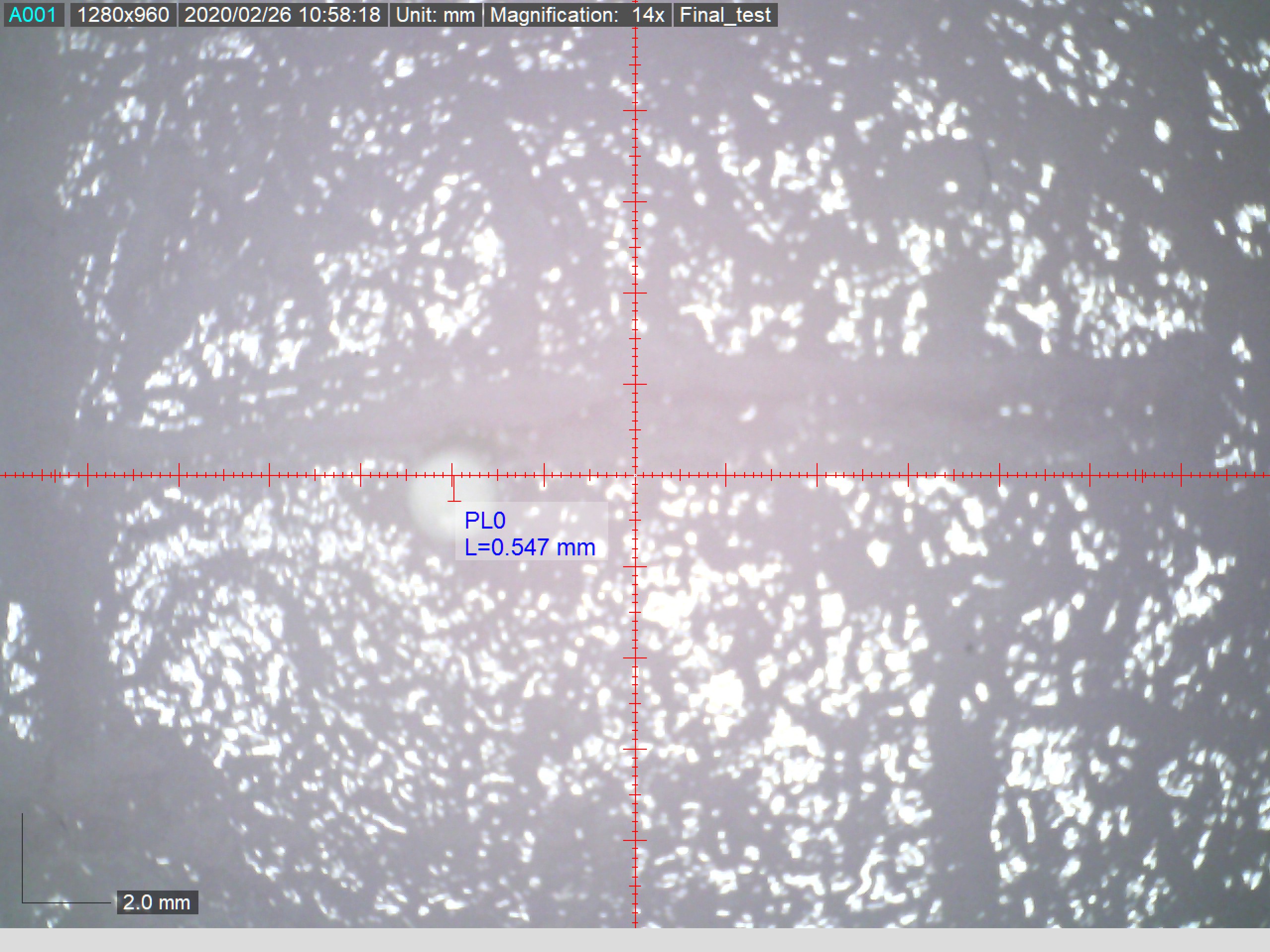

Supplement: S2 File — (ZIP) [file pone.0261089.s002.zip › Soft phantom/photos0.jpg]

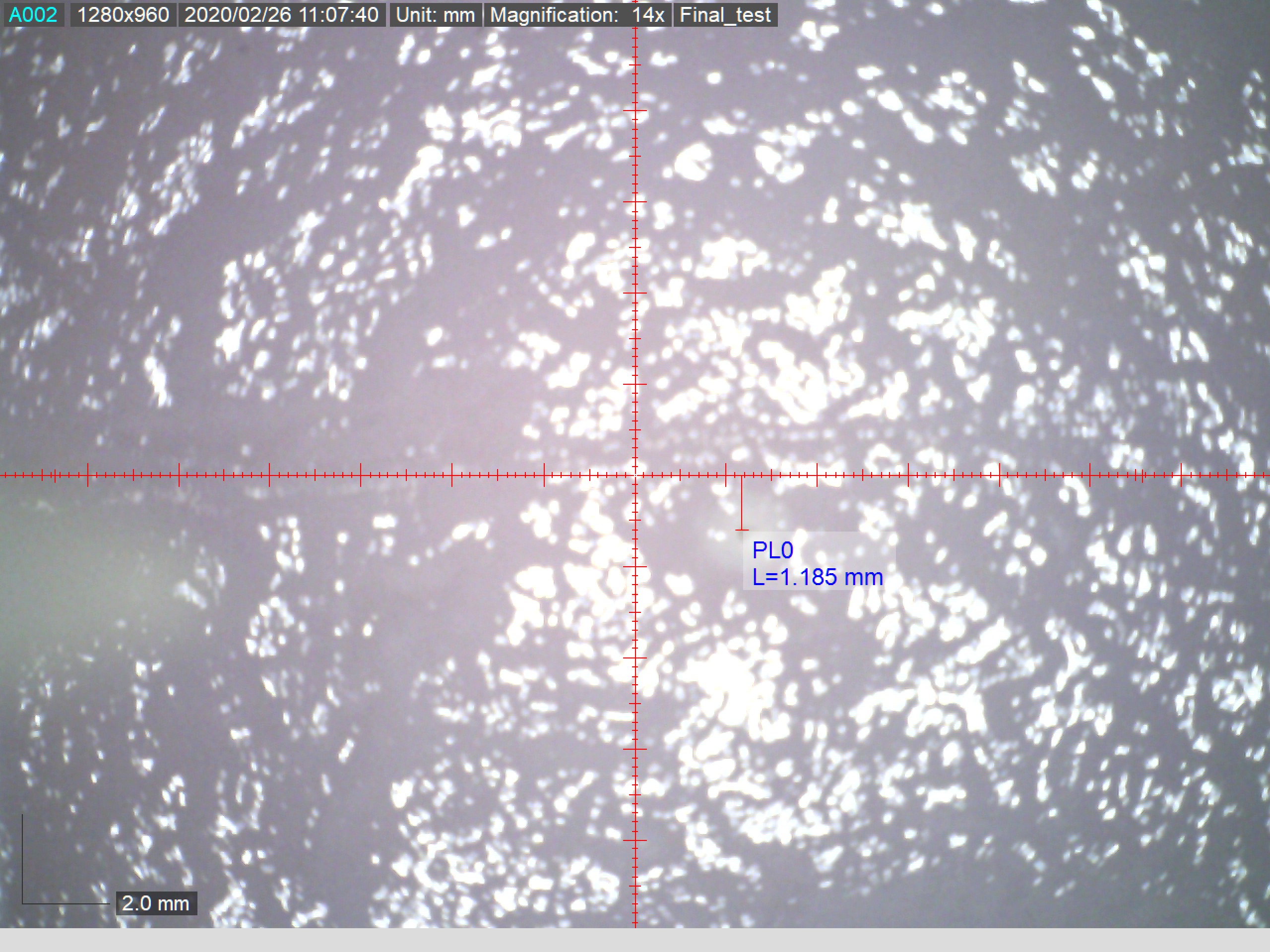

Supplement: S2 File — (ZIP) [file pone.0261089.s002.zip › Soft phantom/photos1.jpg]

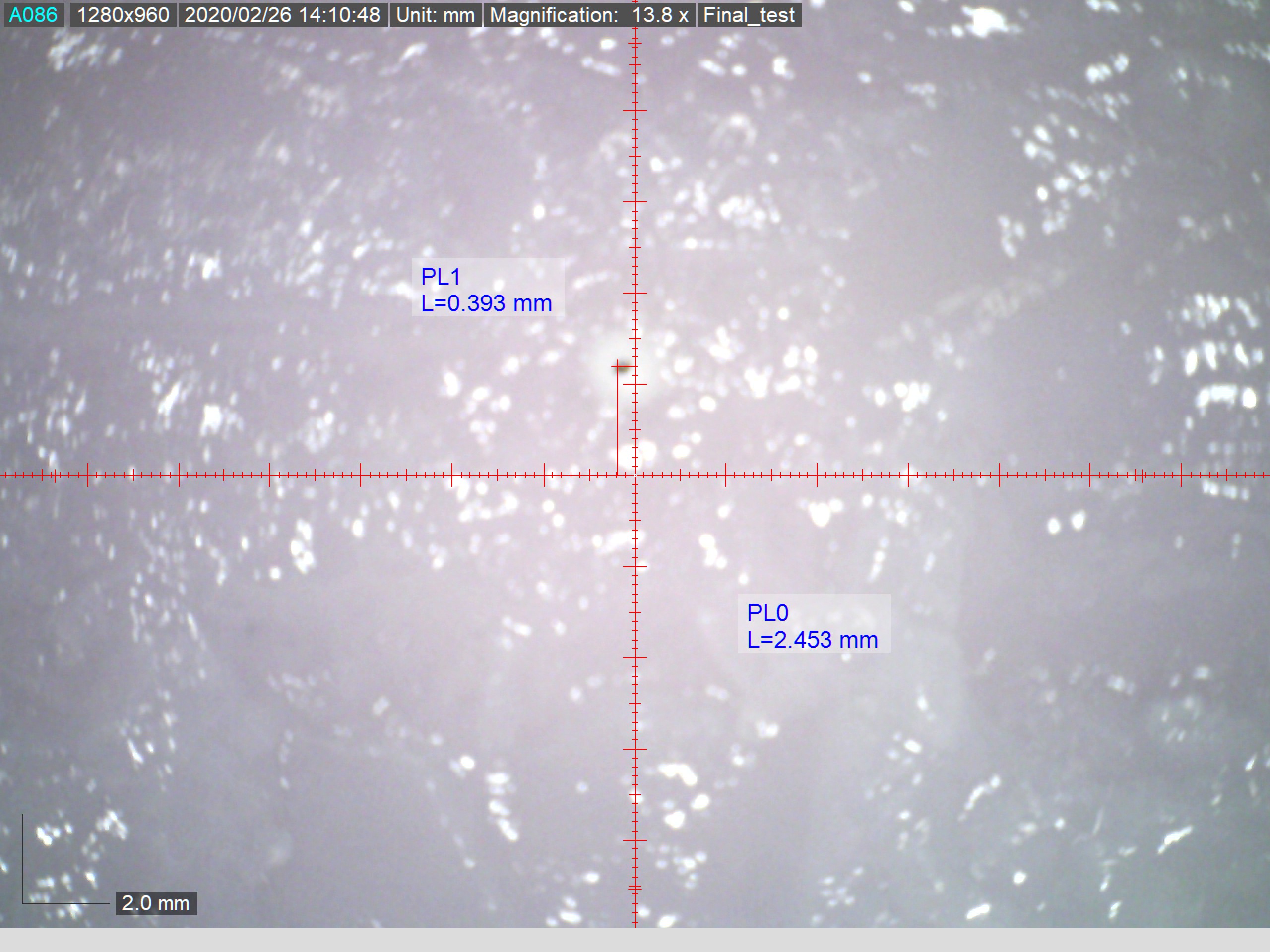

Supplement: S2 File — (ZIP) [file pone.0261089.s002.zip › Soft phantom/photos10.jpg]

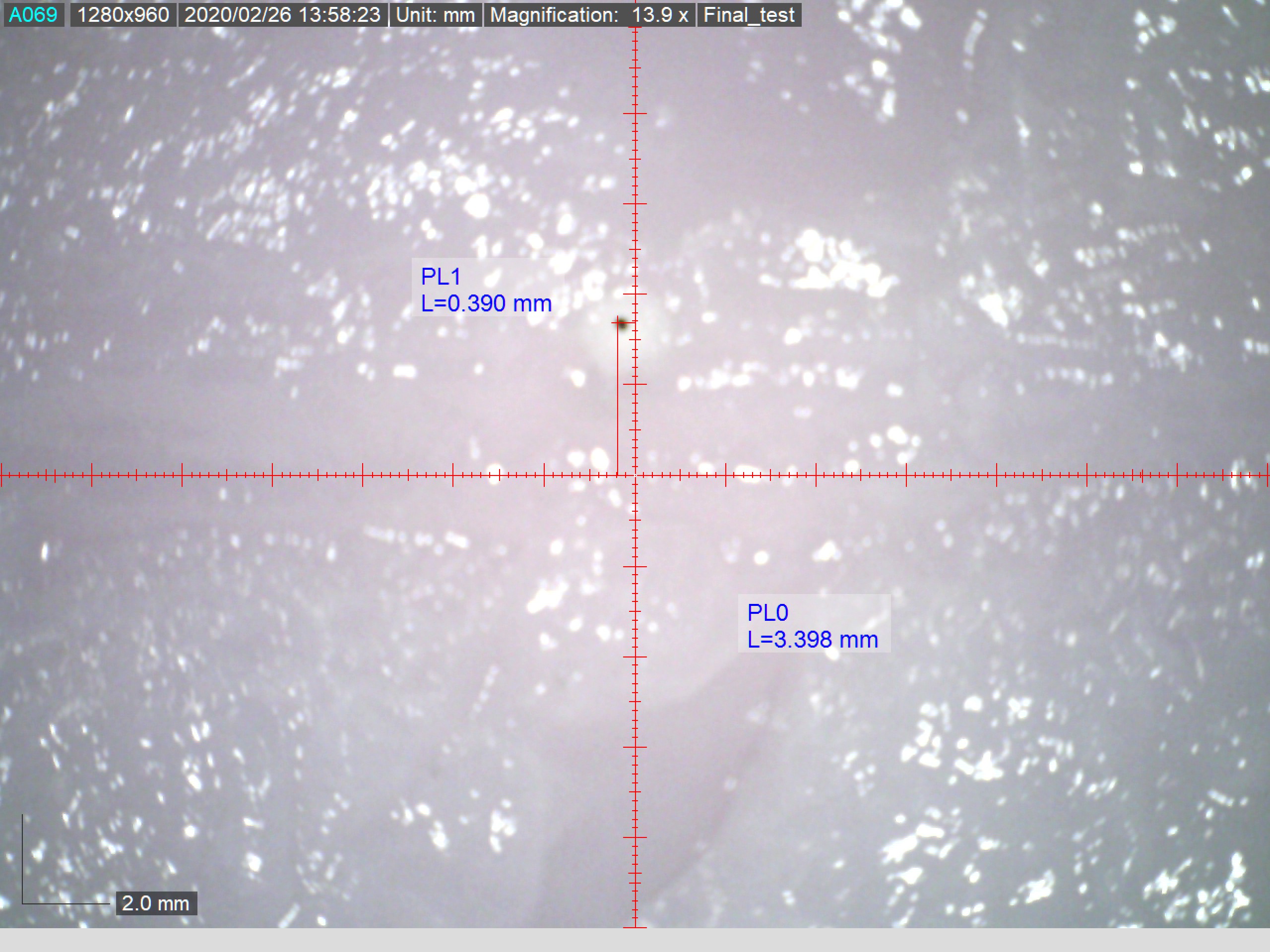

Supplement: S2 File — (ZIP) [file pone.0261089.s002.zip › Soft phantom/photos11.jpg]

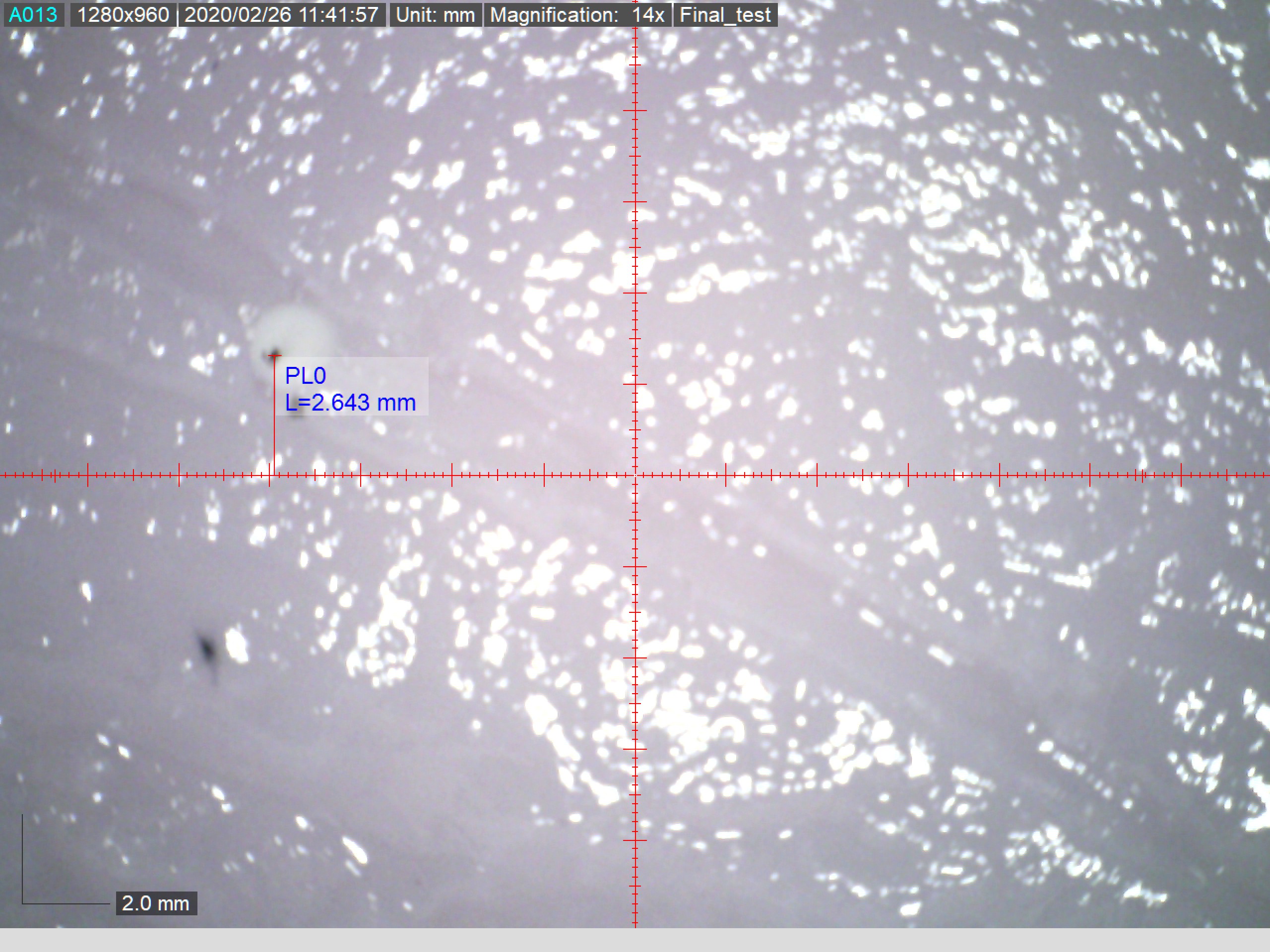

Supplement: S2 File — (ZIP) [file pone.0261089.s002.zip › Soft phantom/photos12.jpg]

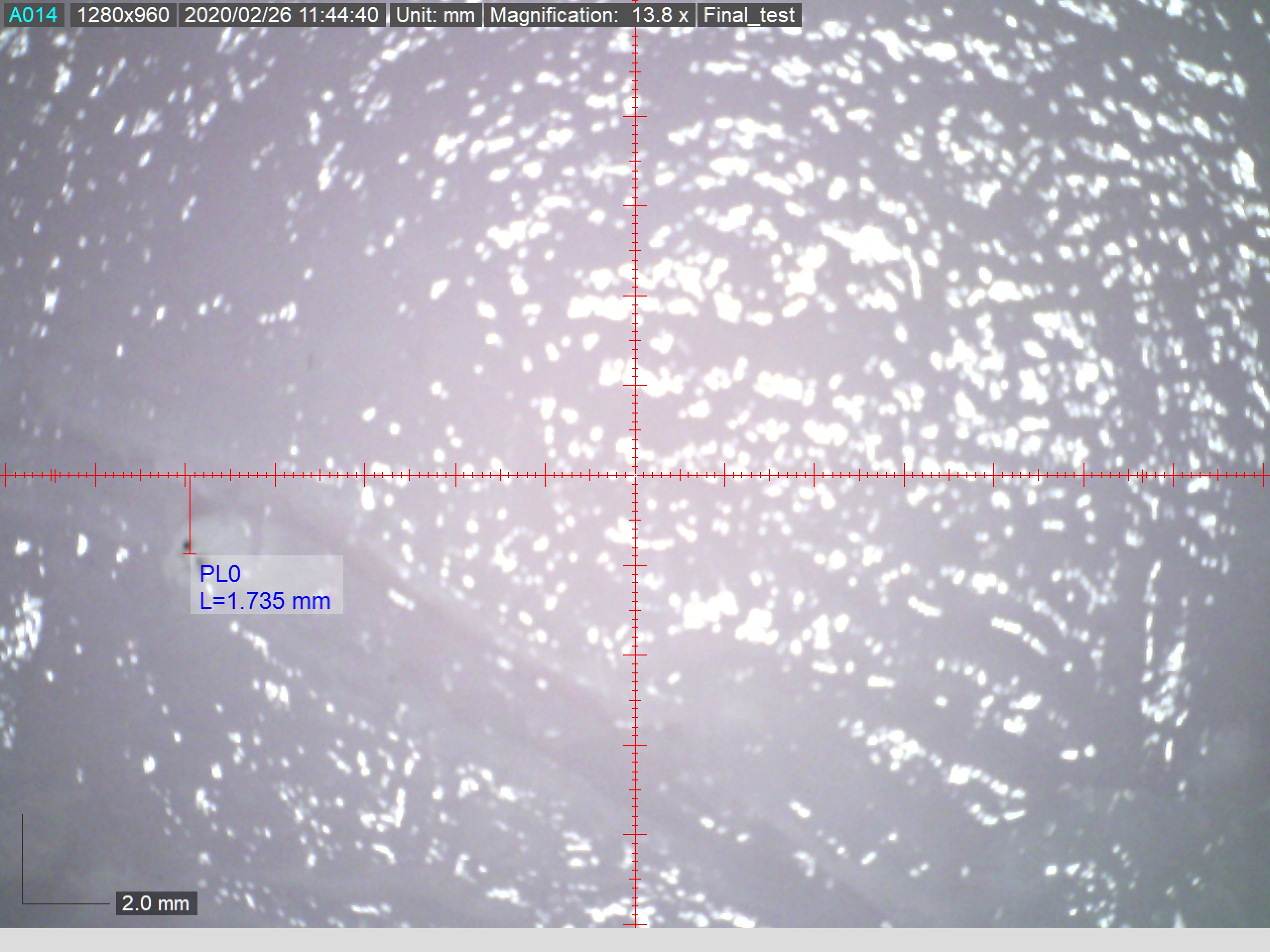

Supplement: S2 File — (ZIP) [file pone.0261089.s002.zip › Soft phantom/photos13.jpg]

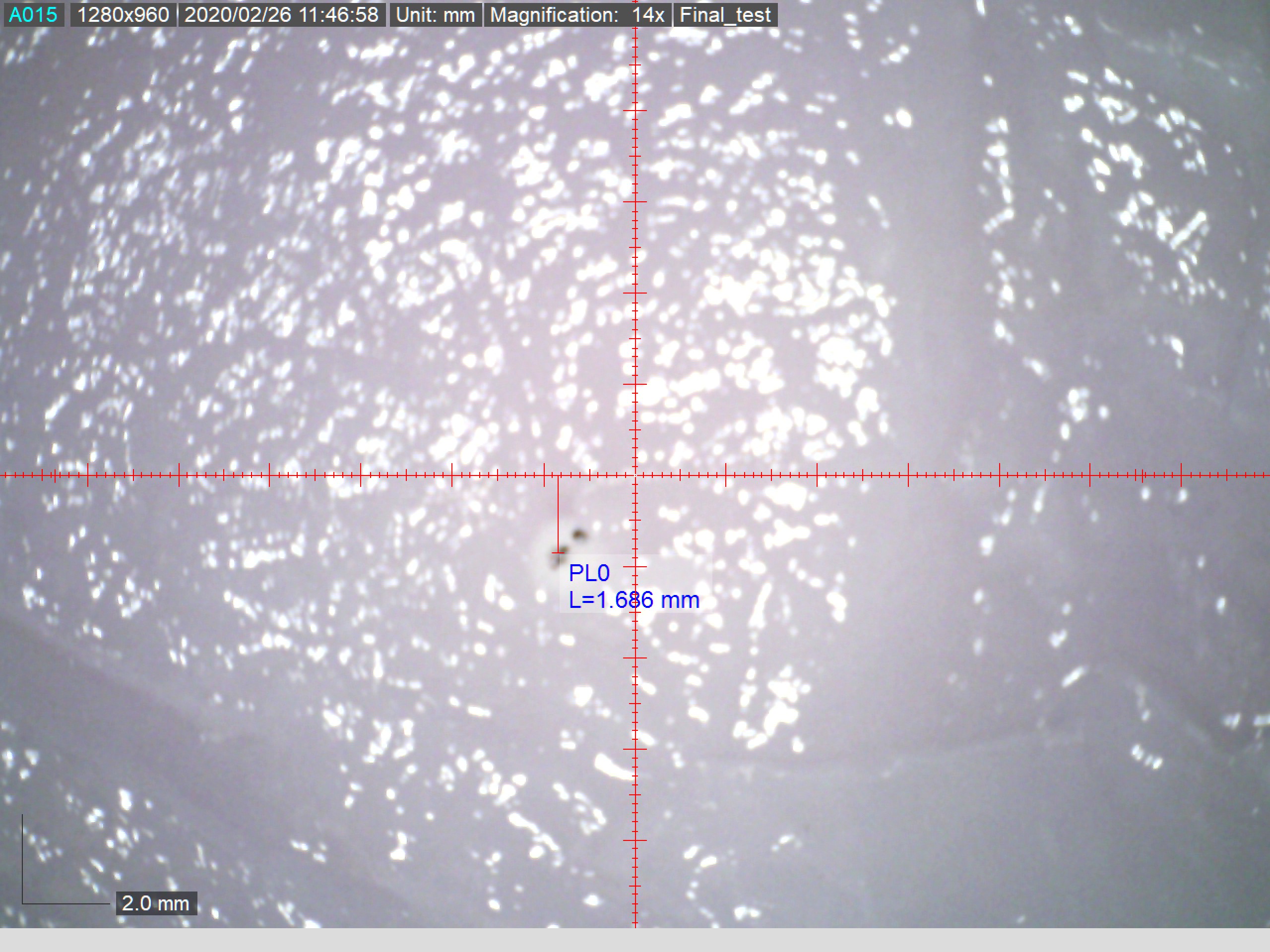

Supplement: S2 File — (ZIP) [file pone.0261089.s002.zip › Soft phantom/photos14.jpg]

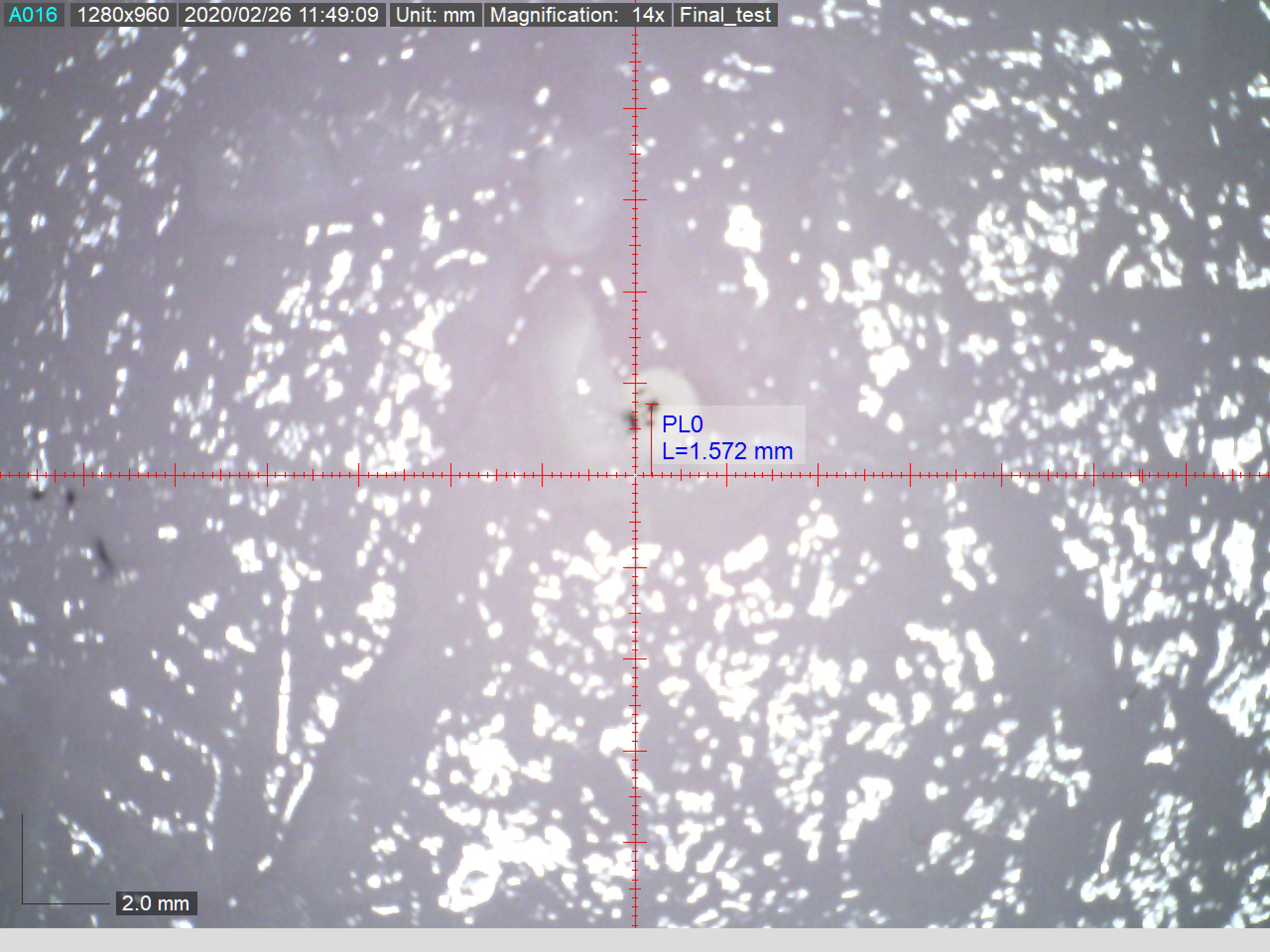

Supplement: S2 File — (ZIP) [file pone.0261089.s002.zip › Soft phantom/photos15.jpg]

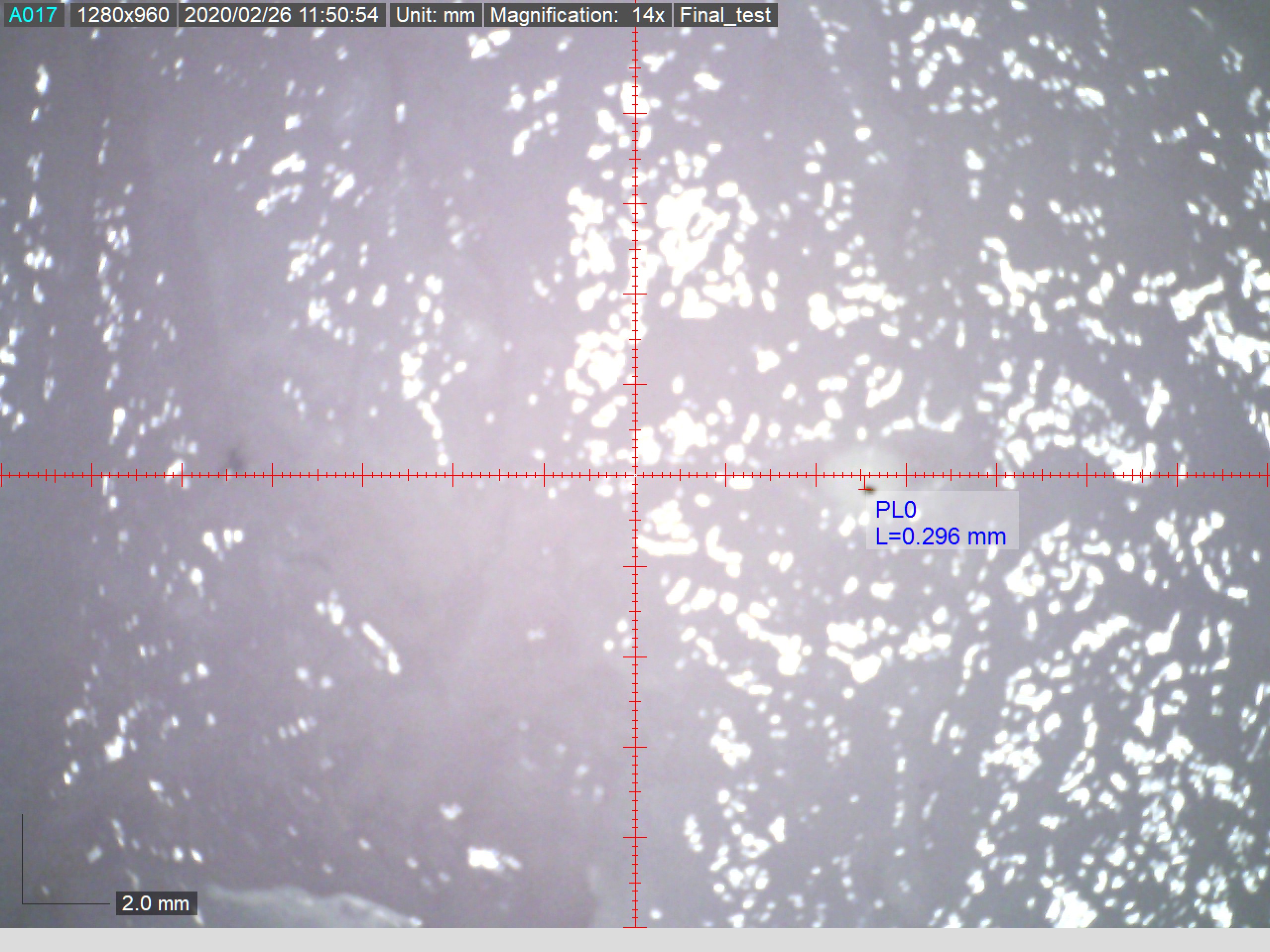

Supplement: S2 File — (ZIP) [file pone.0261089.s002.zip › Soft phantom/photos16.jpg]

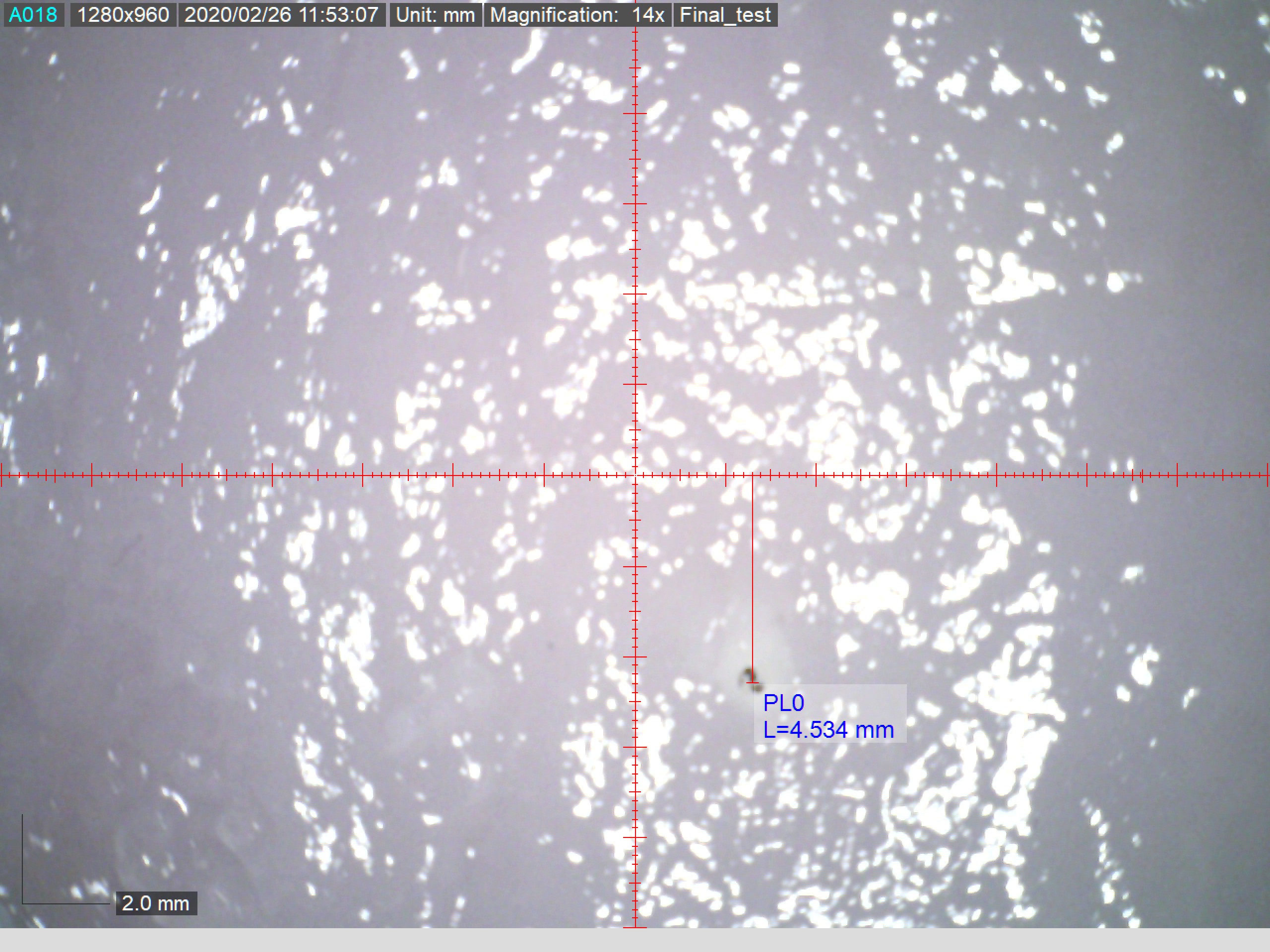

Supplement: S2 File — (ZIP) [file pone.0261089.s002.zip › Soft phantom/photos17.jpg]

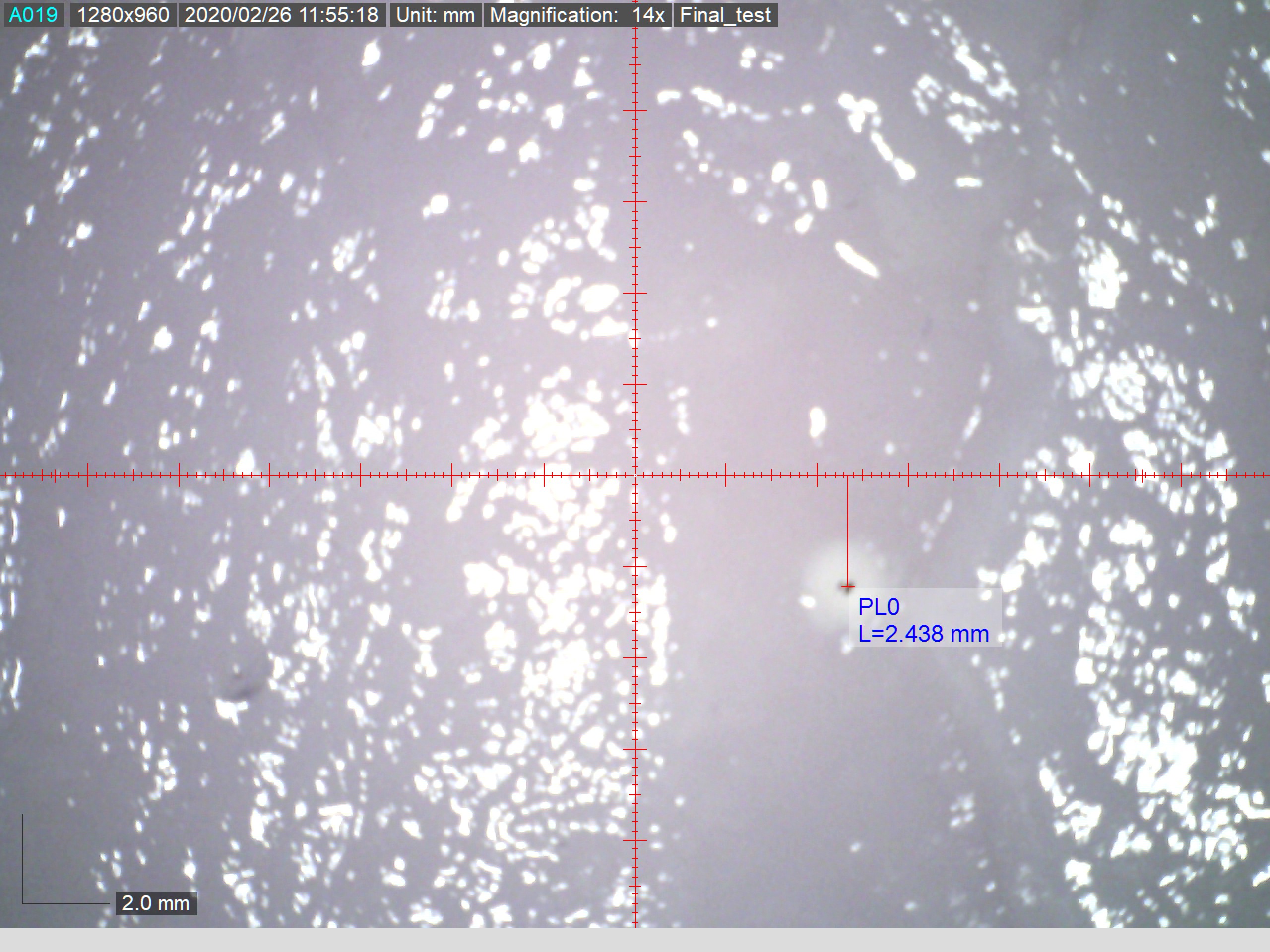

Supplement: S2 File — (ZIP) [file pone.0261089.s002.zip › Soft phantom/photos18.jpg]

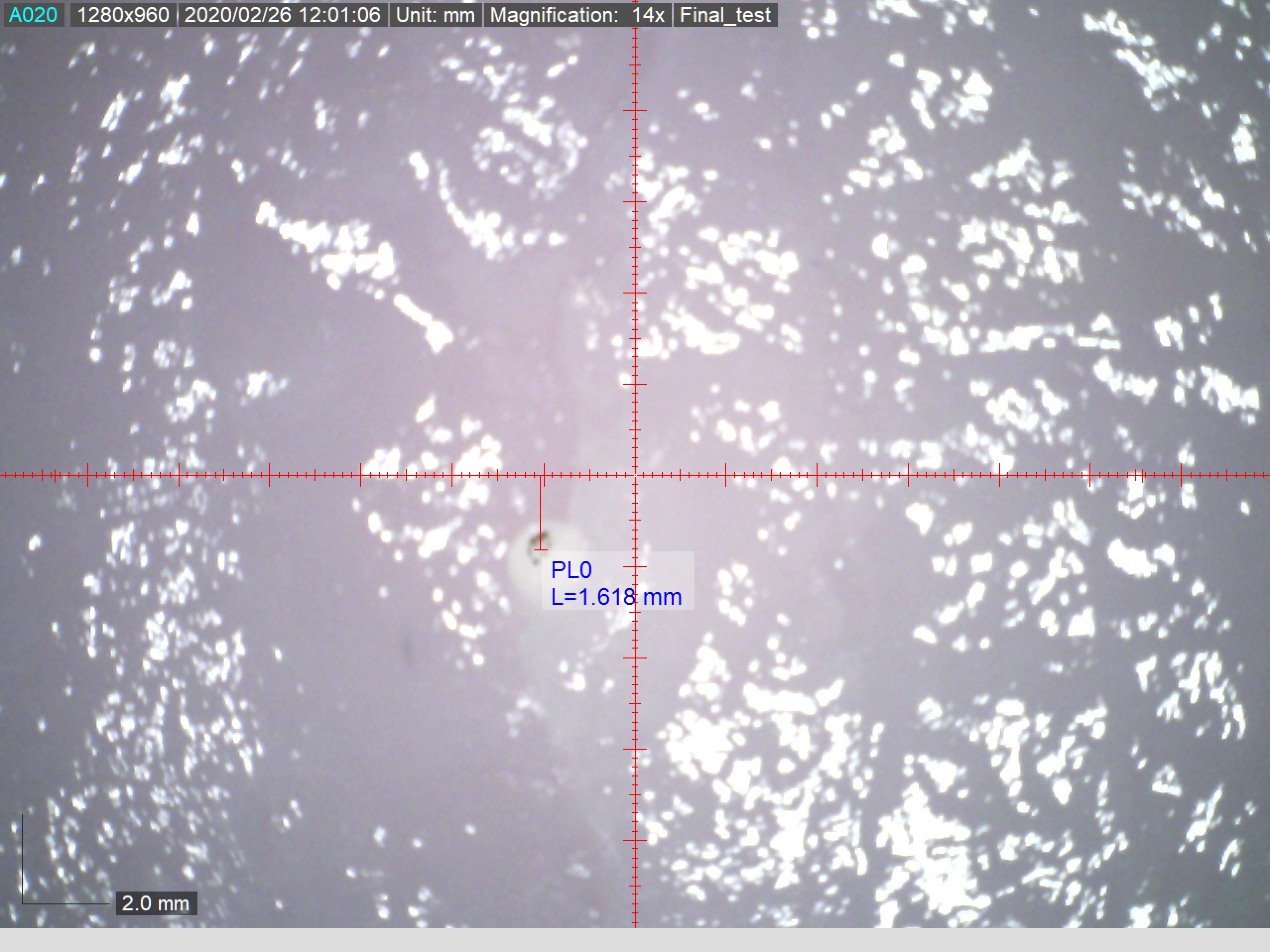

Supplement: S2 File — (ZIP) [file pone.0261089.s002.zip › Soft phantom/photos19.jpg]

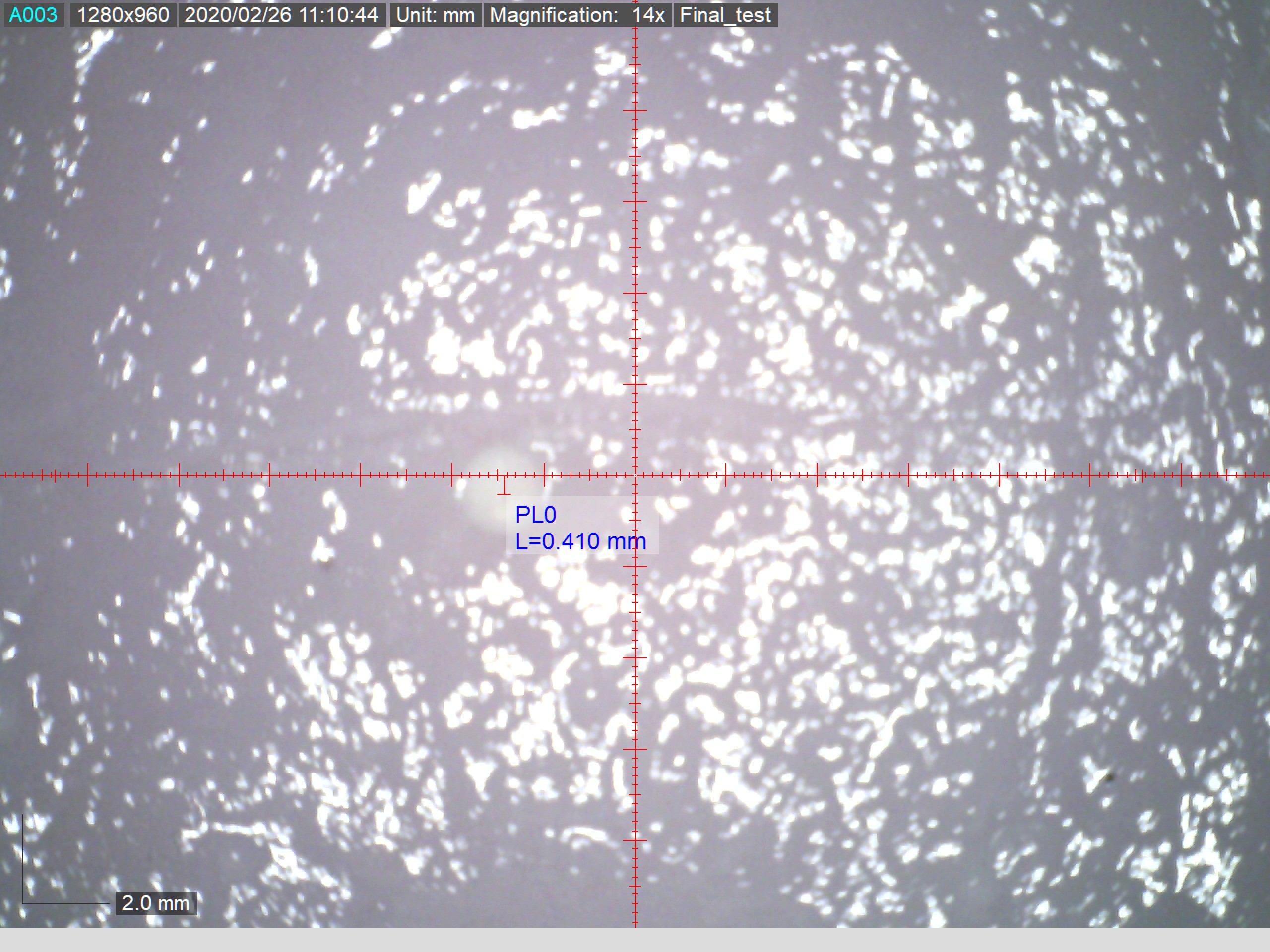

Supplement: S2 File — (ZIP) [file pone.0261089.s002.zip › Soft phantom/photos2.jpg]

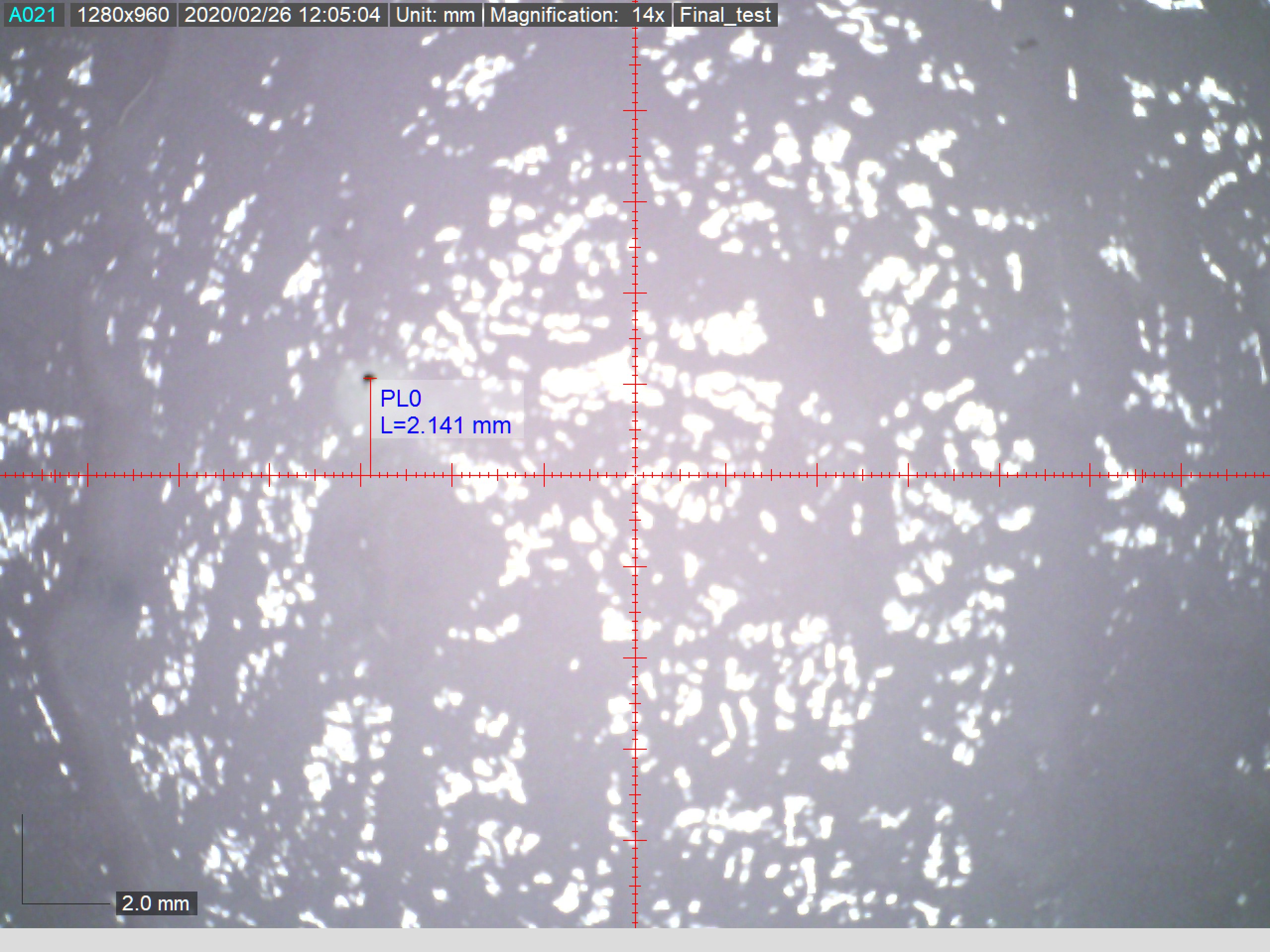

Supplement: S2 File — (ZIP) [file pone.0261089.s002.zip › Soft phantom/photos20.jpg]

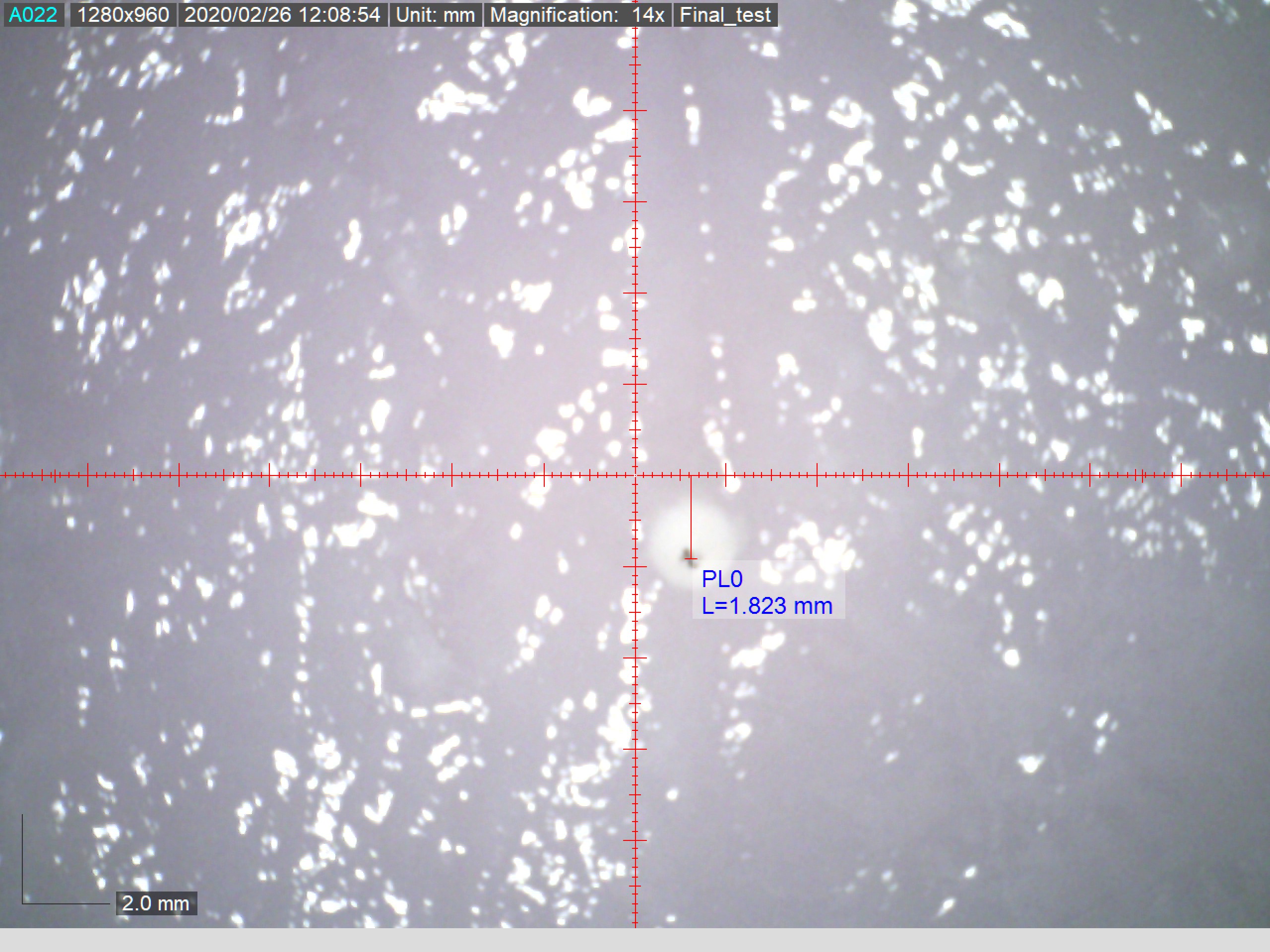

Supplement: S2 File — (ZIP) [file pone.0261089.s002.zip › Soft phantom/photos21.jpg]

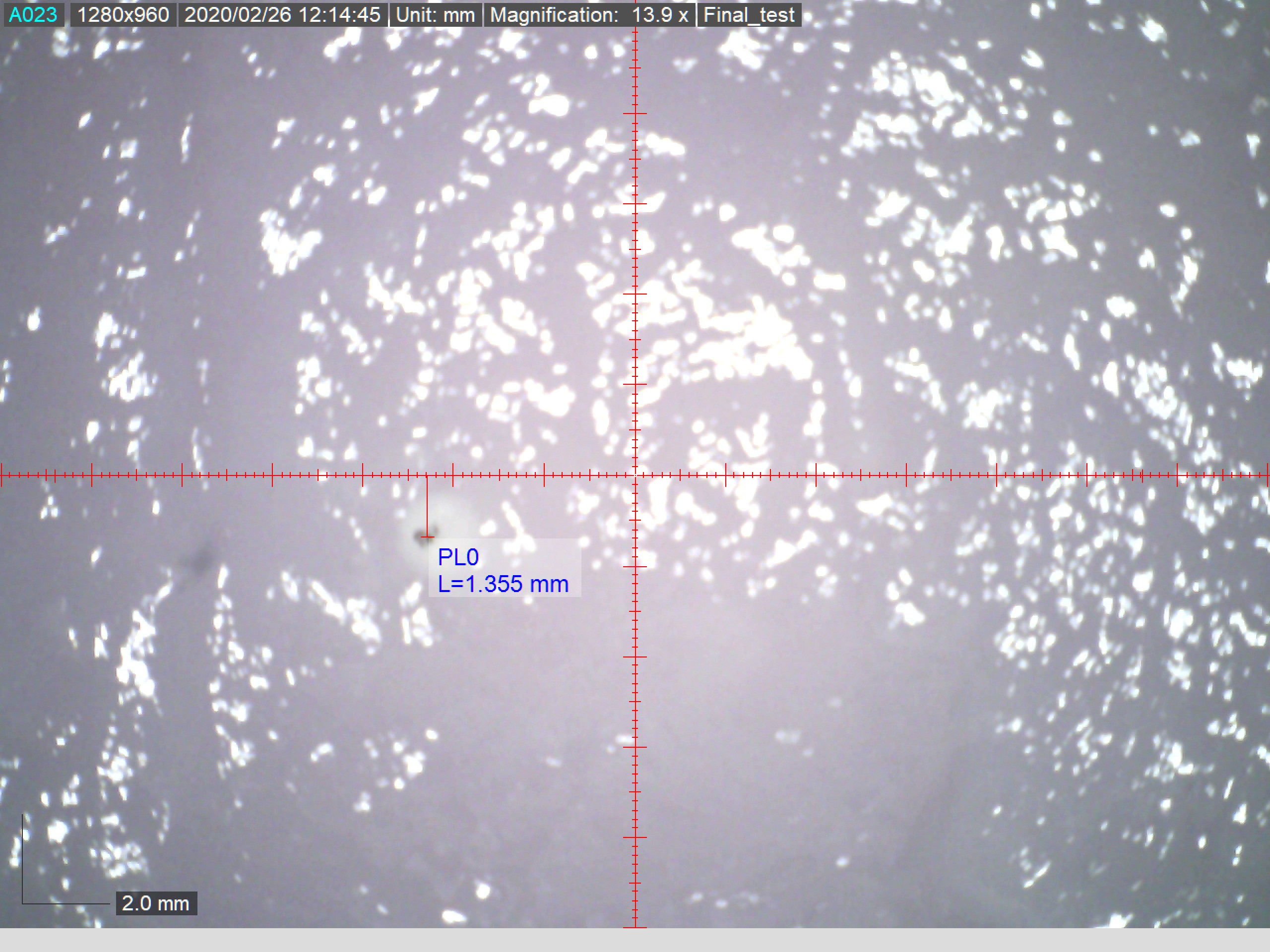

Supplement: S2 File — (ZIP) [file pone.0261089.s002.zip › Soft phantom/photos22.jpg]

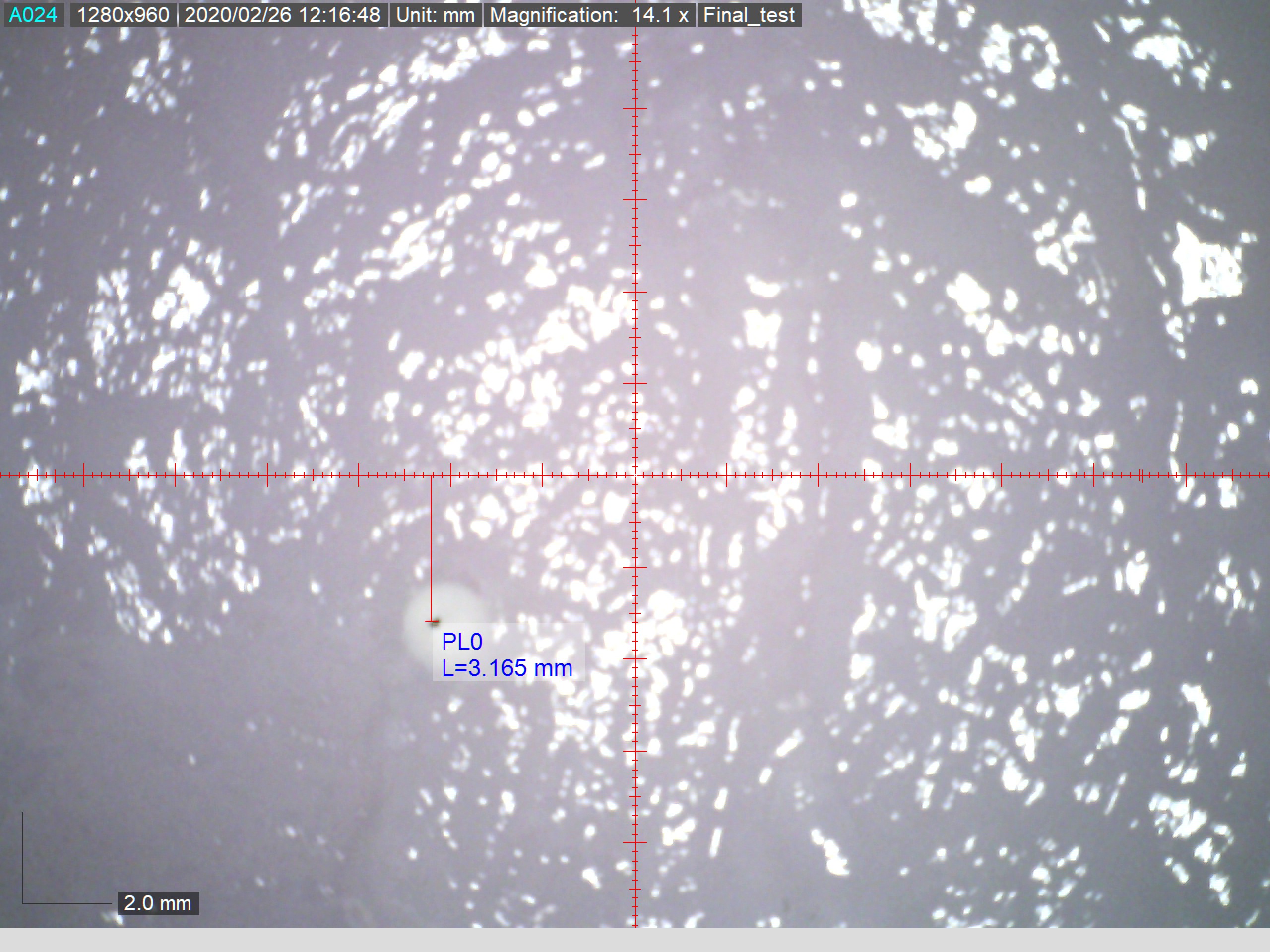

Supplement: S2 File — (ZIP) [file pone.0261089.s002.zip › Soft phantom/photos23.jpg]

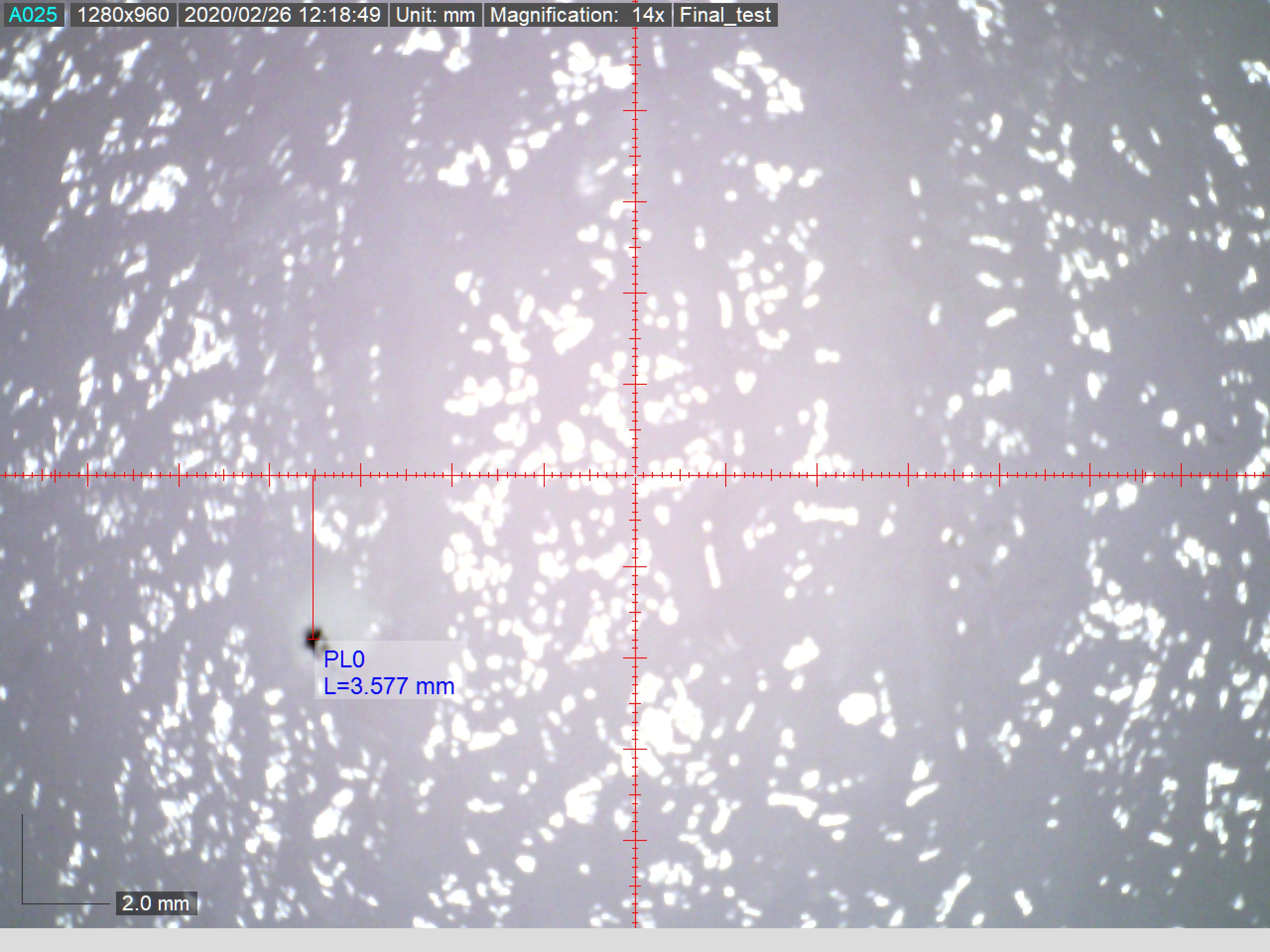

Supplement: S2 File — (ZIP) [file pone.0261089.s002.zip › Soft phantom/photos24.jpg]

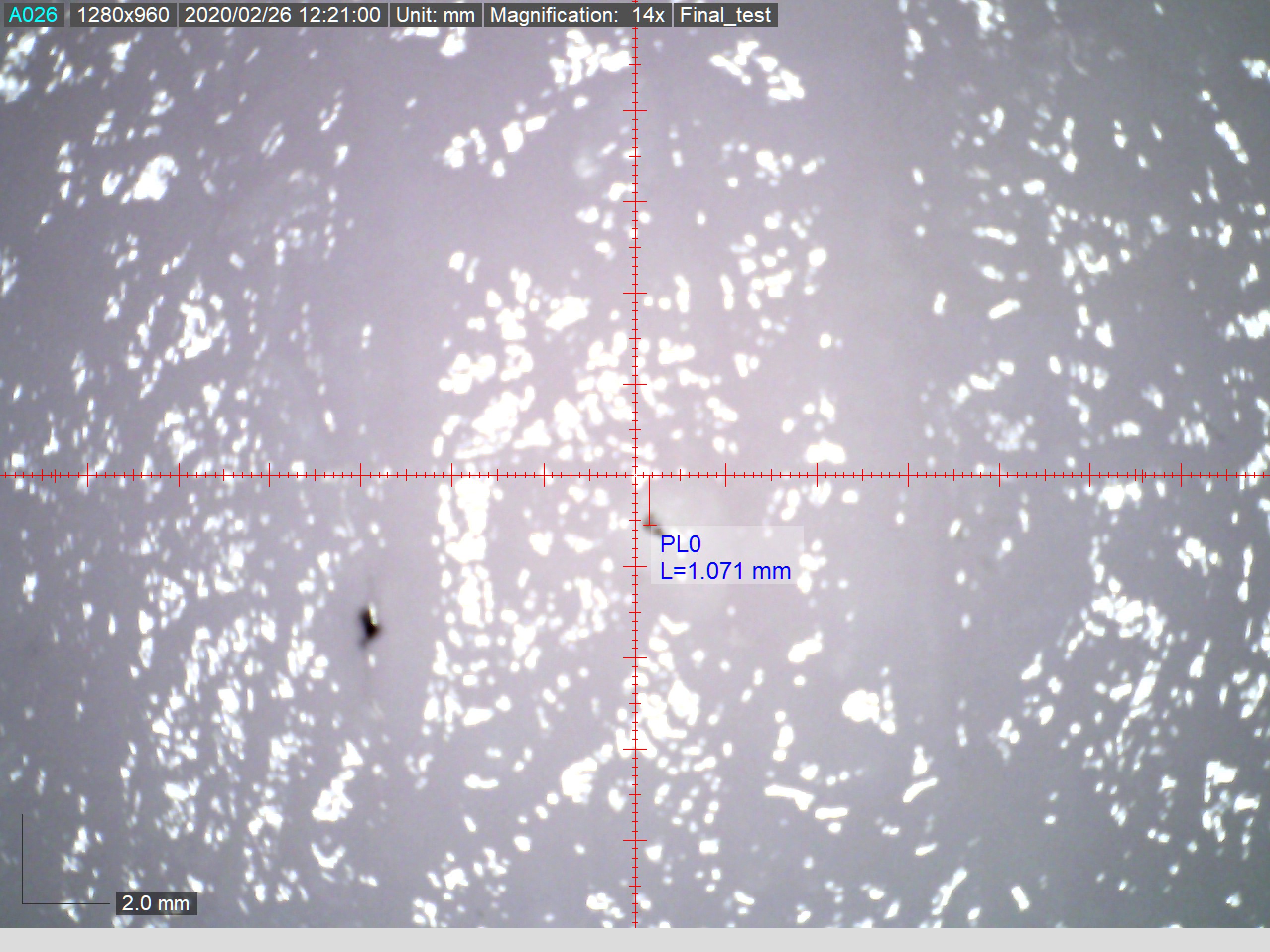

Supplement: S2 File — (ZIP) [file pone.0261089.s002.zip › Soft phantom/photos25.jpg]

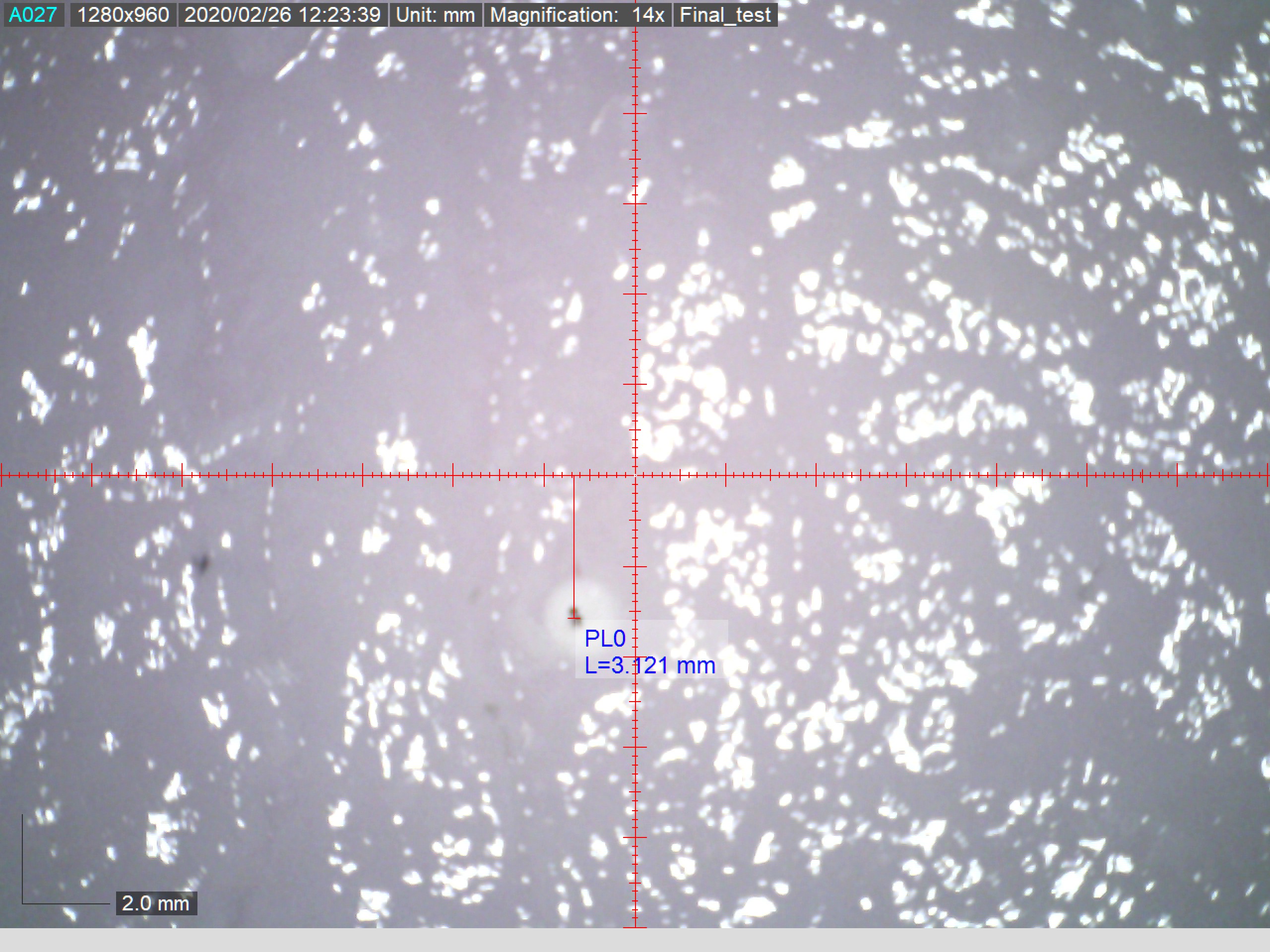

Supplement: S2 File — (ZIP) [file pone.0261089.s002.zip › Soft phantom/photos26.jpg]

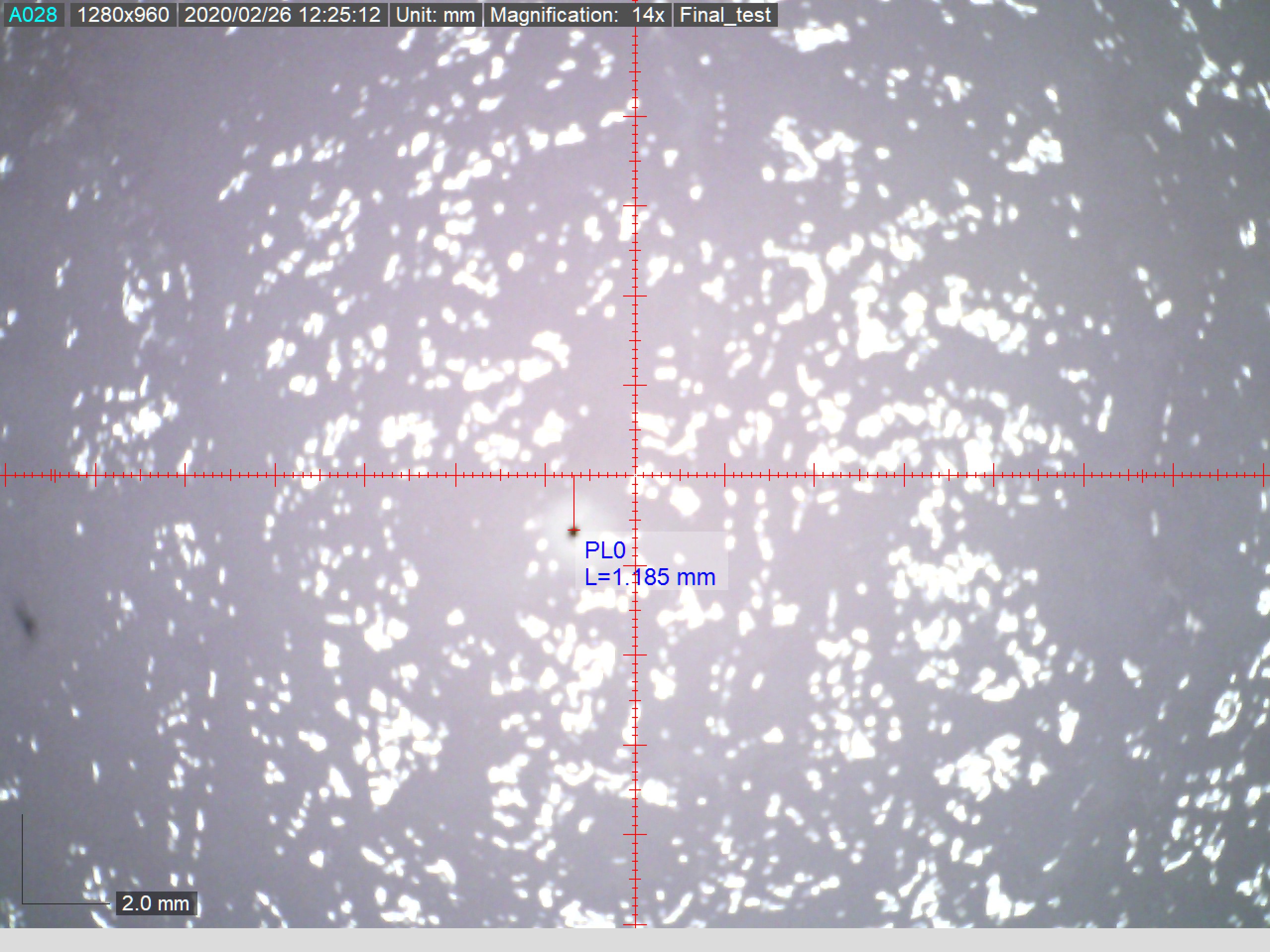

Supplement: S2 File — (ZIP) [file pone.0261089.s002.zip › Soft phantom/photos27.jpg]

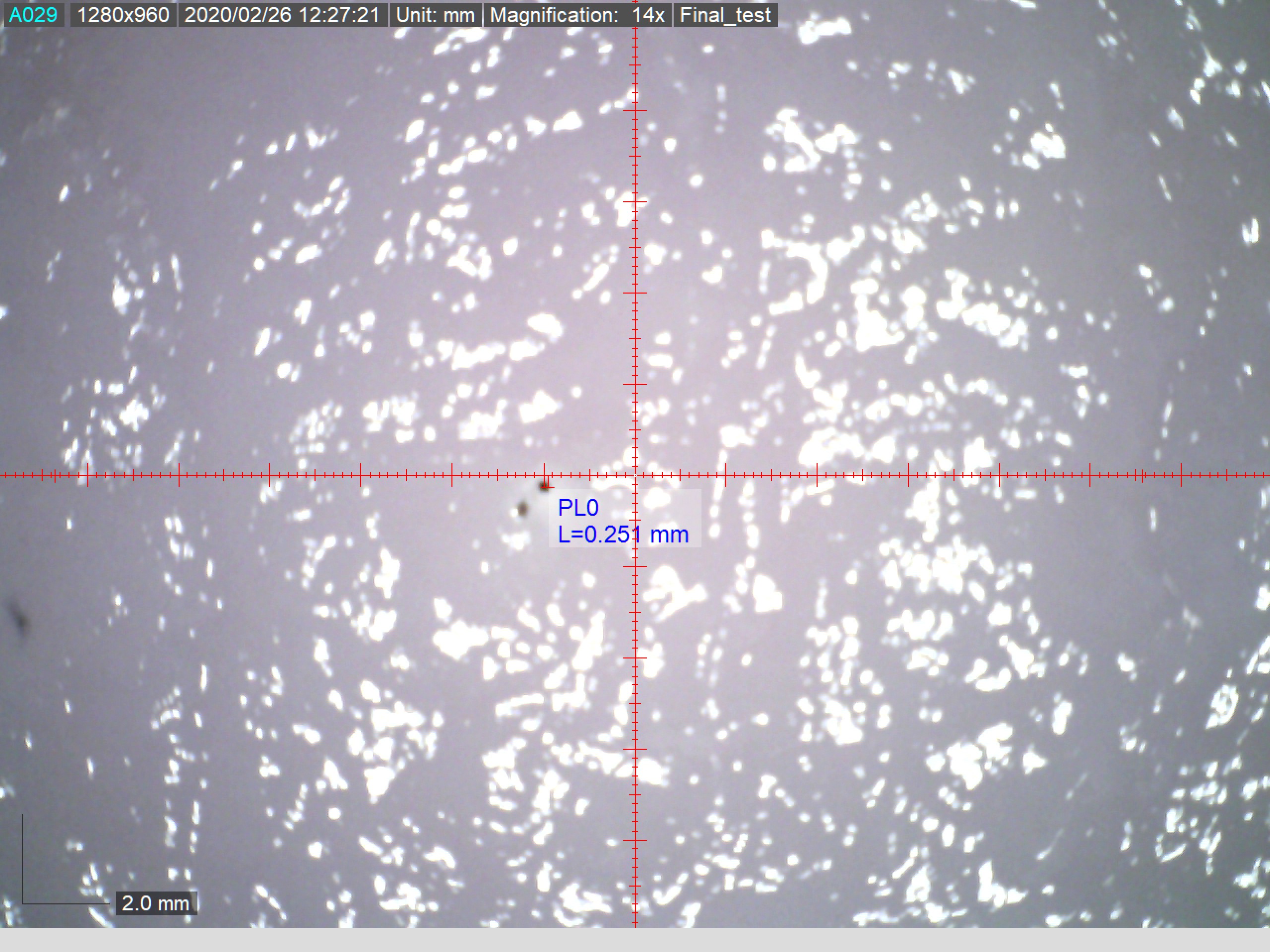

Supplement: S2 File — (ZIP) [file pone.0261089.s002.zip › Soft phantom/photos28.jpg]

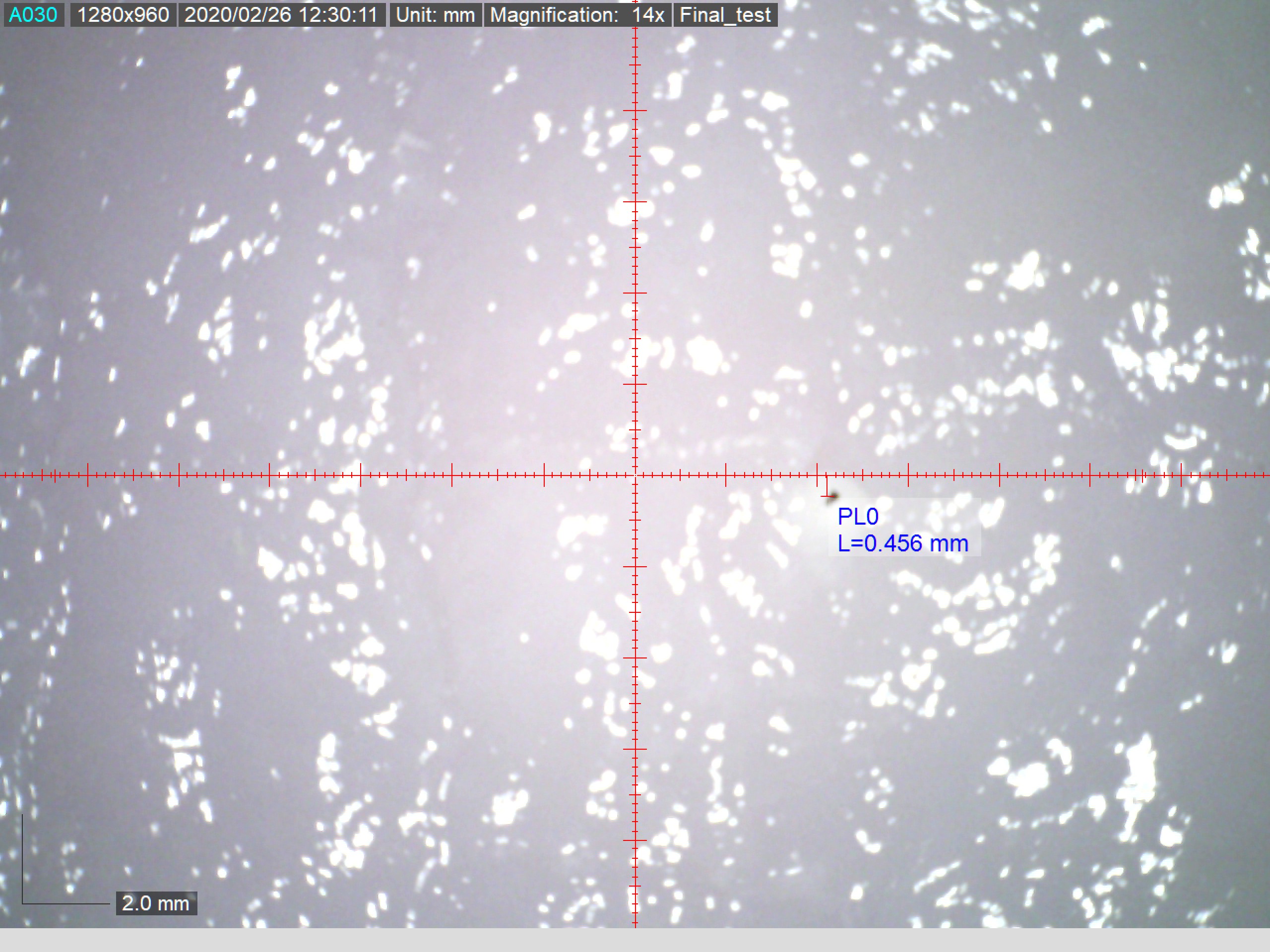

Supplement: S2 File — (ZIP) [file pone.0261089.s002.zip › Soft phantom/photos29.jpg]

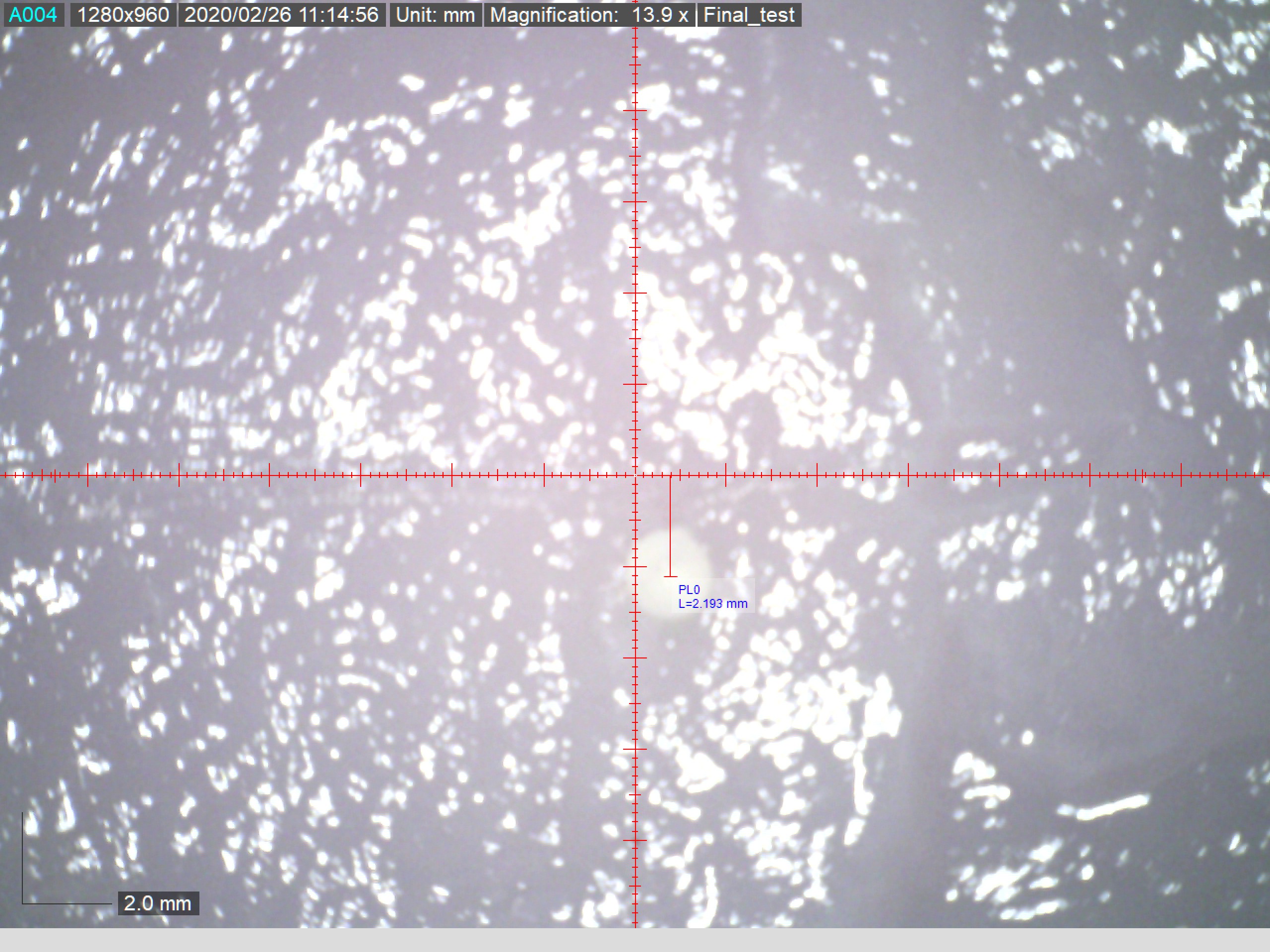

Supplement: S2 File — (ZIP) [file pone.0261089.s002.zip › Soft phantom/photos3.jpg]

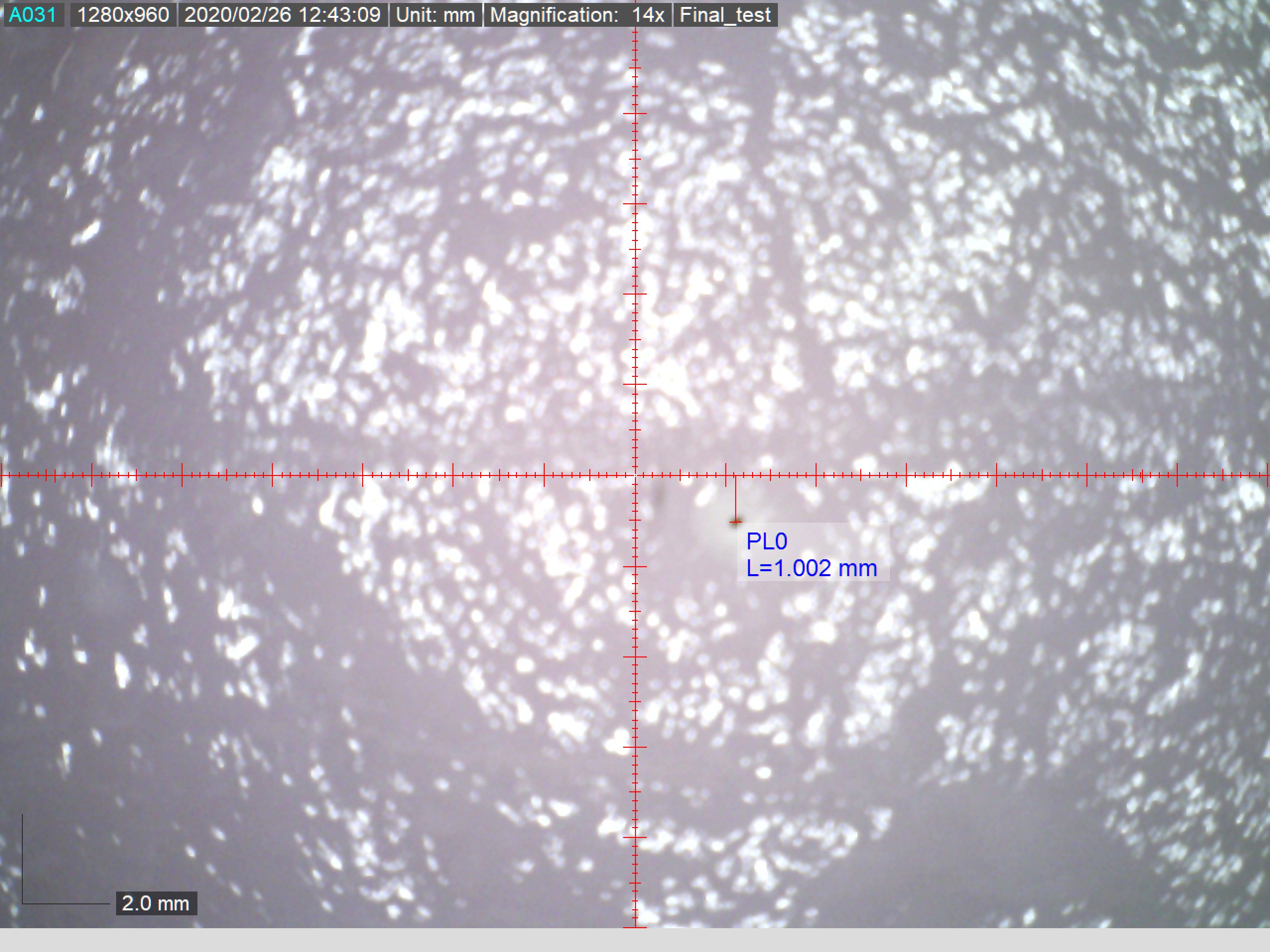

Supplement: S2 File — (ZIP) [file pone.0261089.s002.zip › Soft phantom/photos30.jpg]

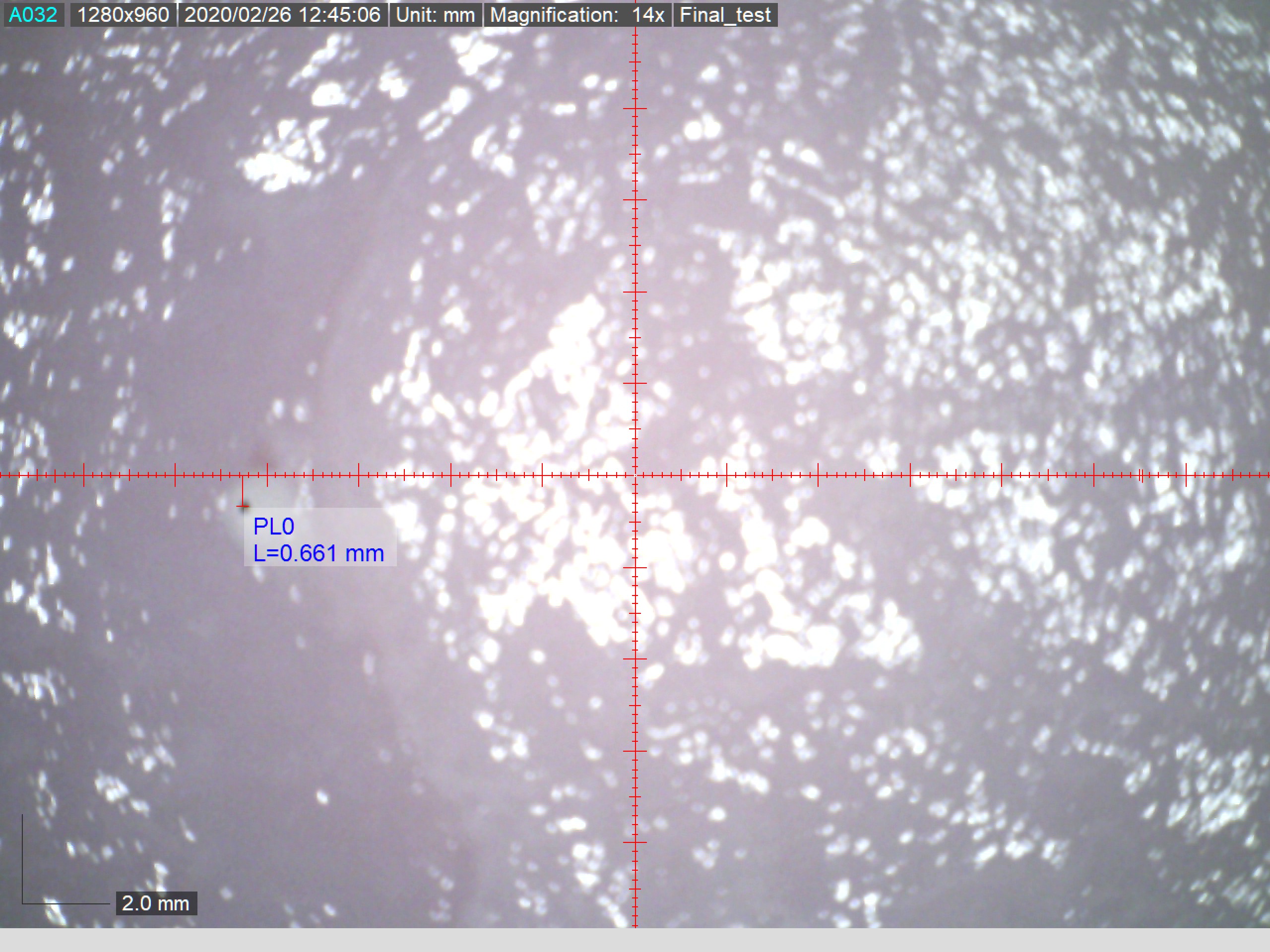

Supplement: S2 File — (ZIP) [file pone.0261089.s002.zip › Soft phantom/photos31.jpg]

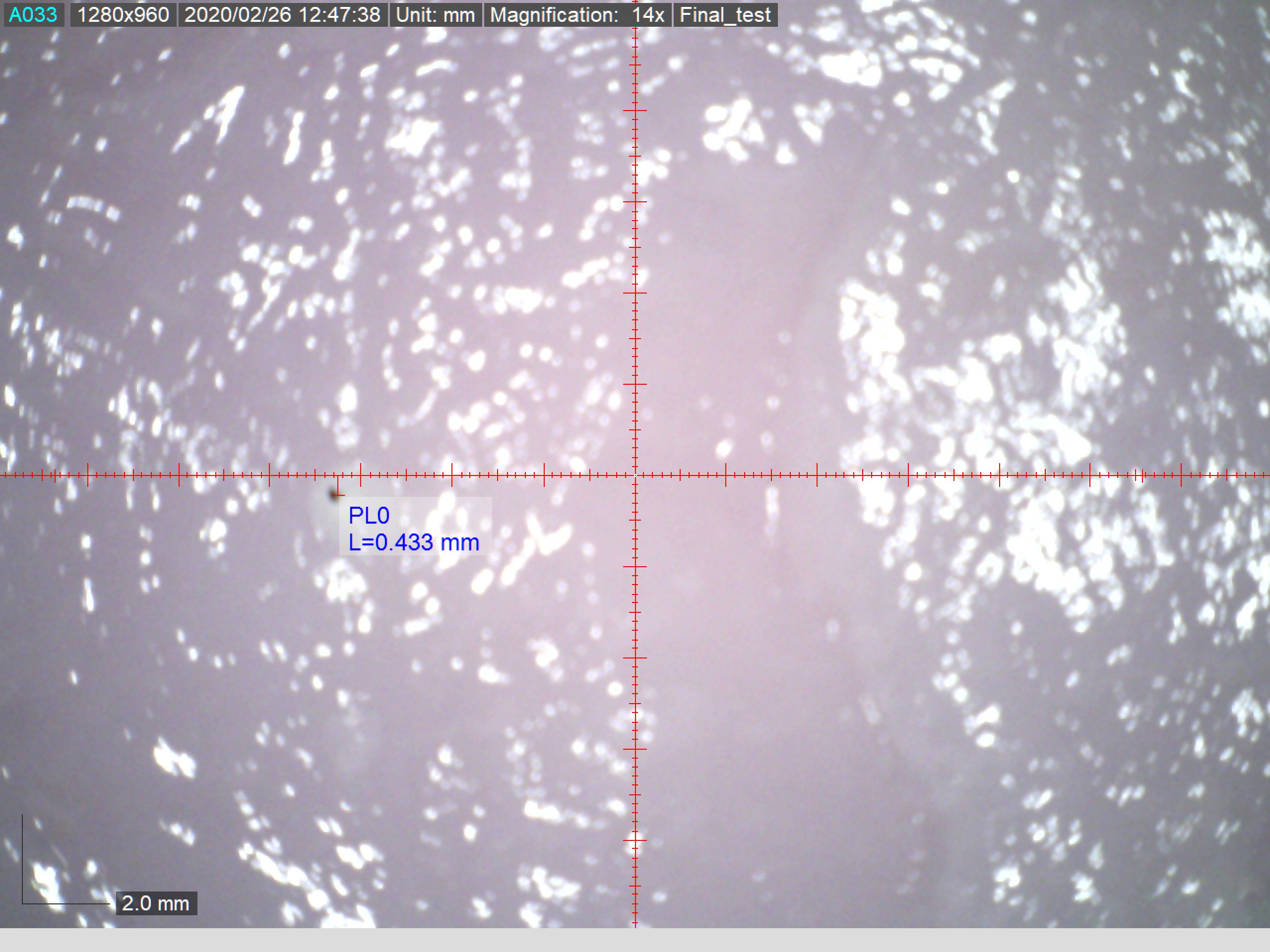

Supplement: S2 File — (ZIP) [file pone.0261089.s002.zip › Soft phantom/photos32.jpg]

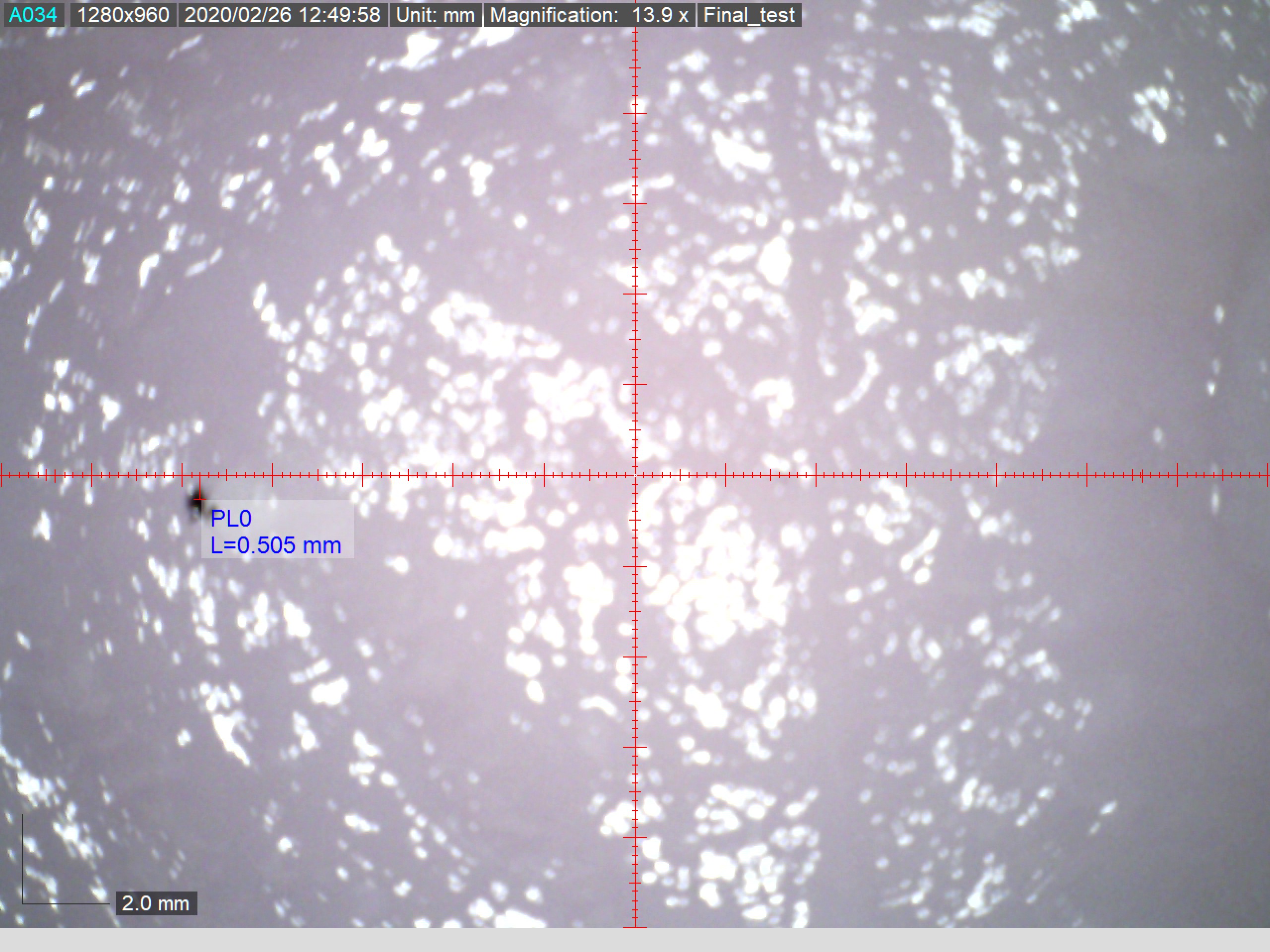

Supplement: S2 File — (ZIP) [file pone.0261089.s002.zip › Soft phantom/photos33.jpg]

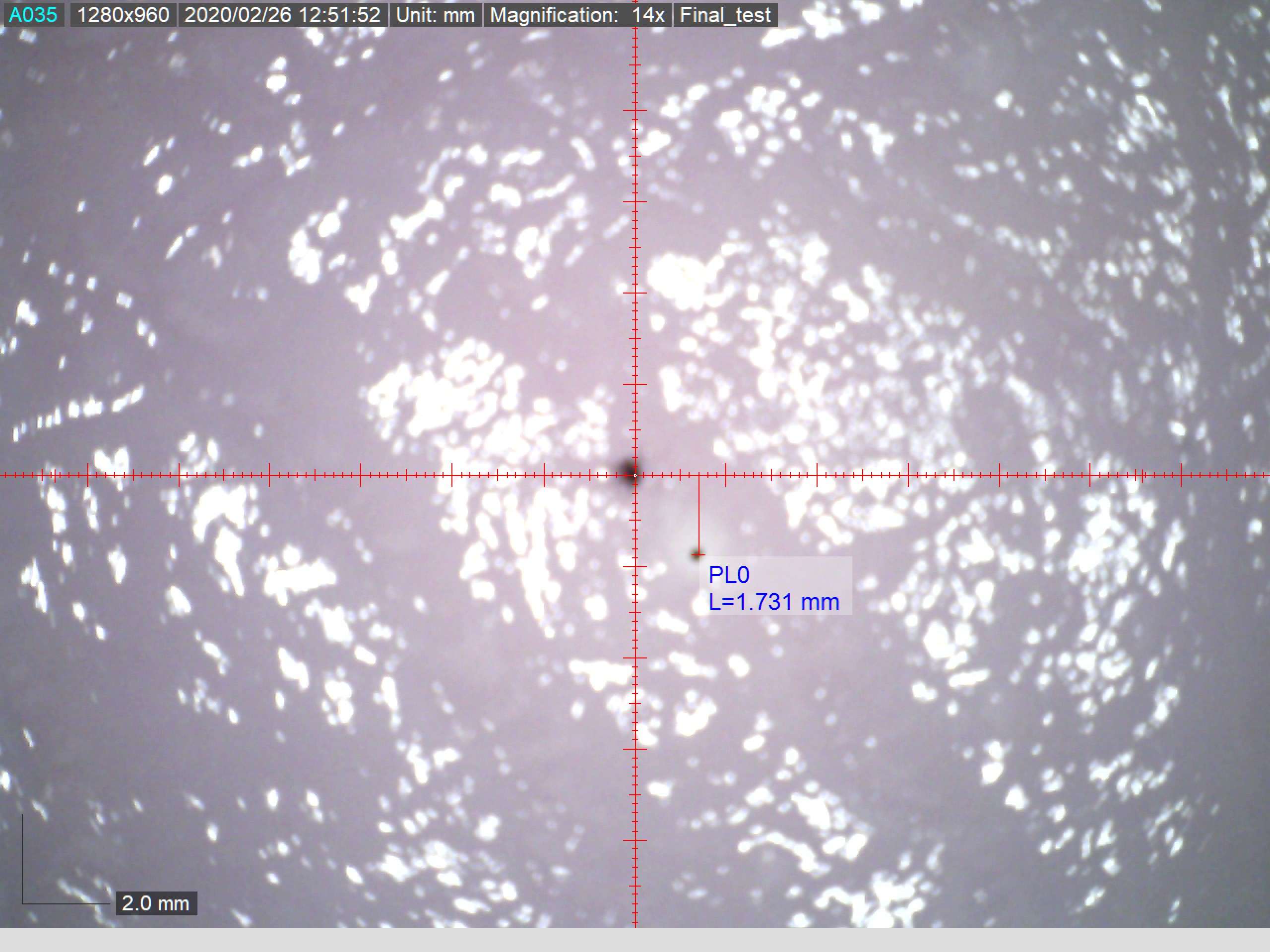

Supplement: S2 File — (ZIP) [file pone.0261089.s002.zip › Soft phantom/photos34.jpg]

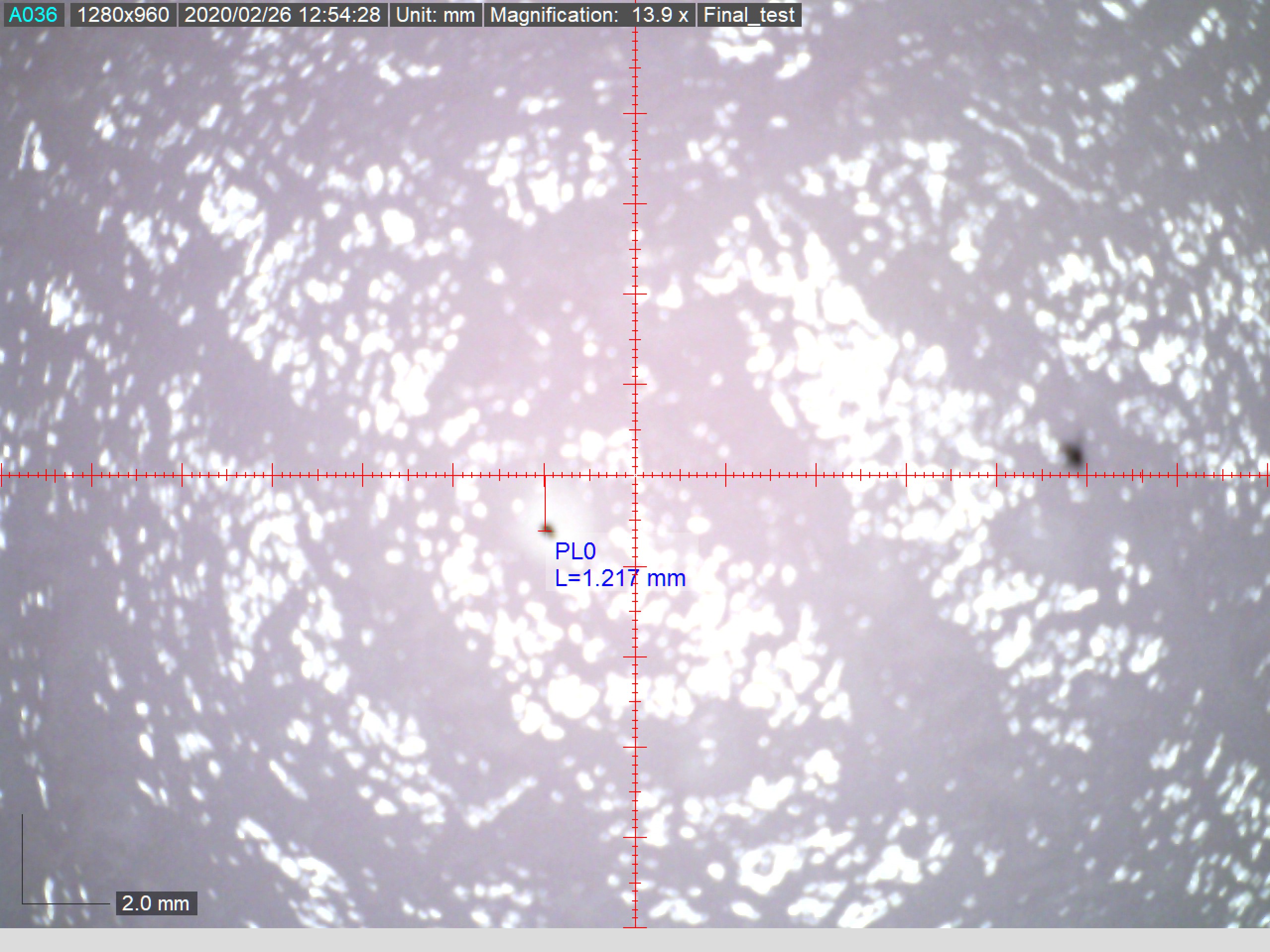

Supplement: S2 File — (ZIP) [file pone.0261089.s002.zip › Soft phantom/photos35.jpg]

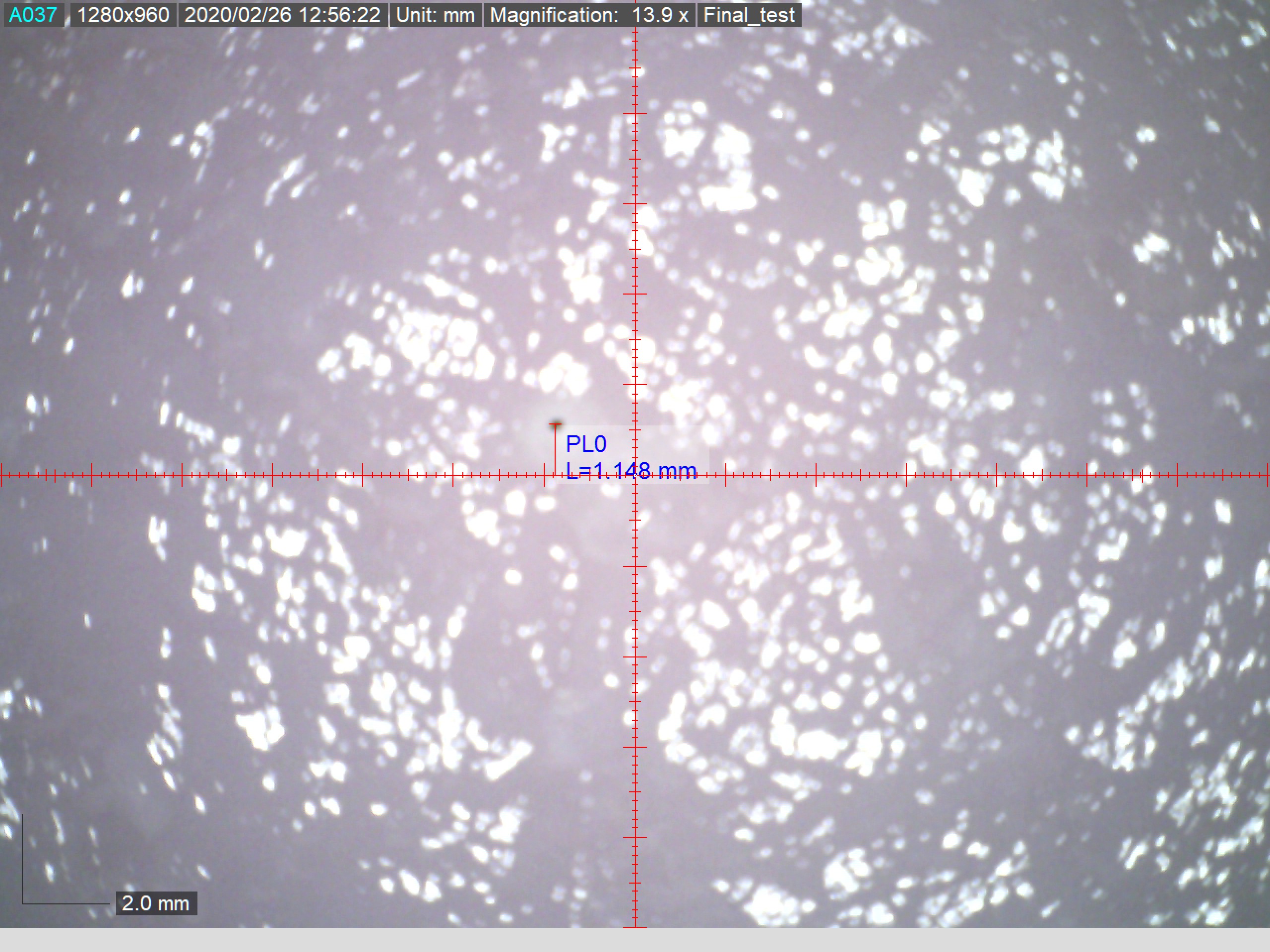

Supplement: S2 File — (ZIP) [file pone.0261089.s002.zip › Soft phantom/photos36.jpg]

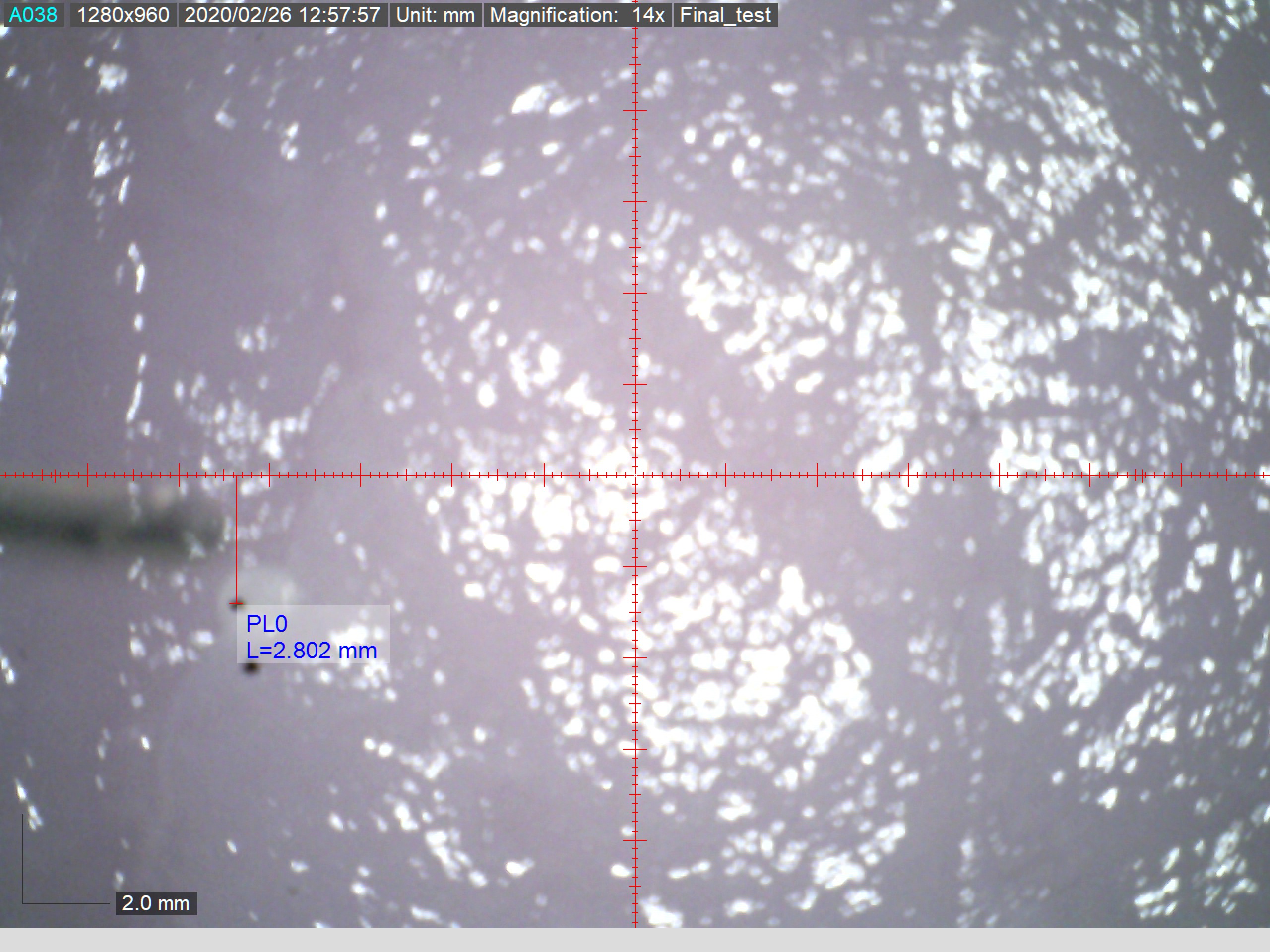

Supplement: S2 File — (ZIP) [file pone.0261089.s002.zip › Soft phantom/photos37.jpg]

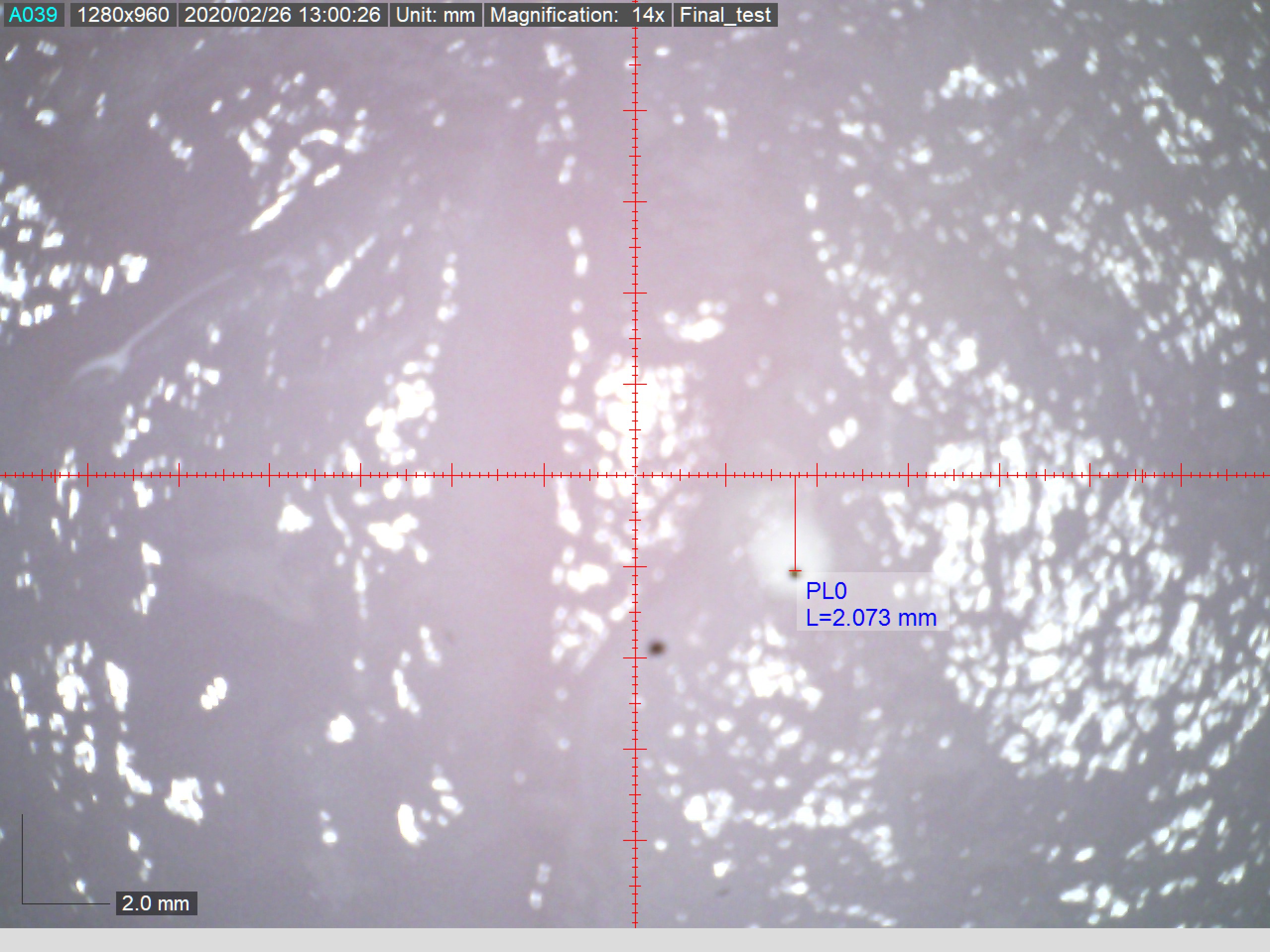

Supplement: S2 File — (ZIP) [file pone.0261089.s002.zip › Soft phantom/photos38.jpg]

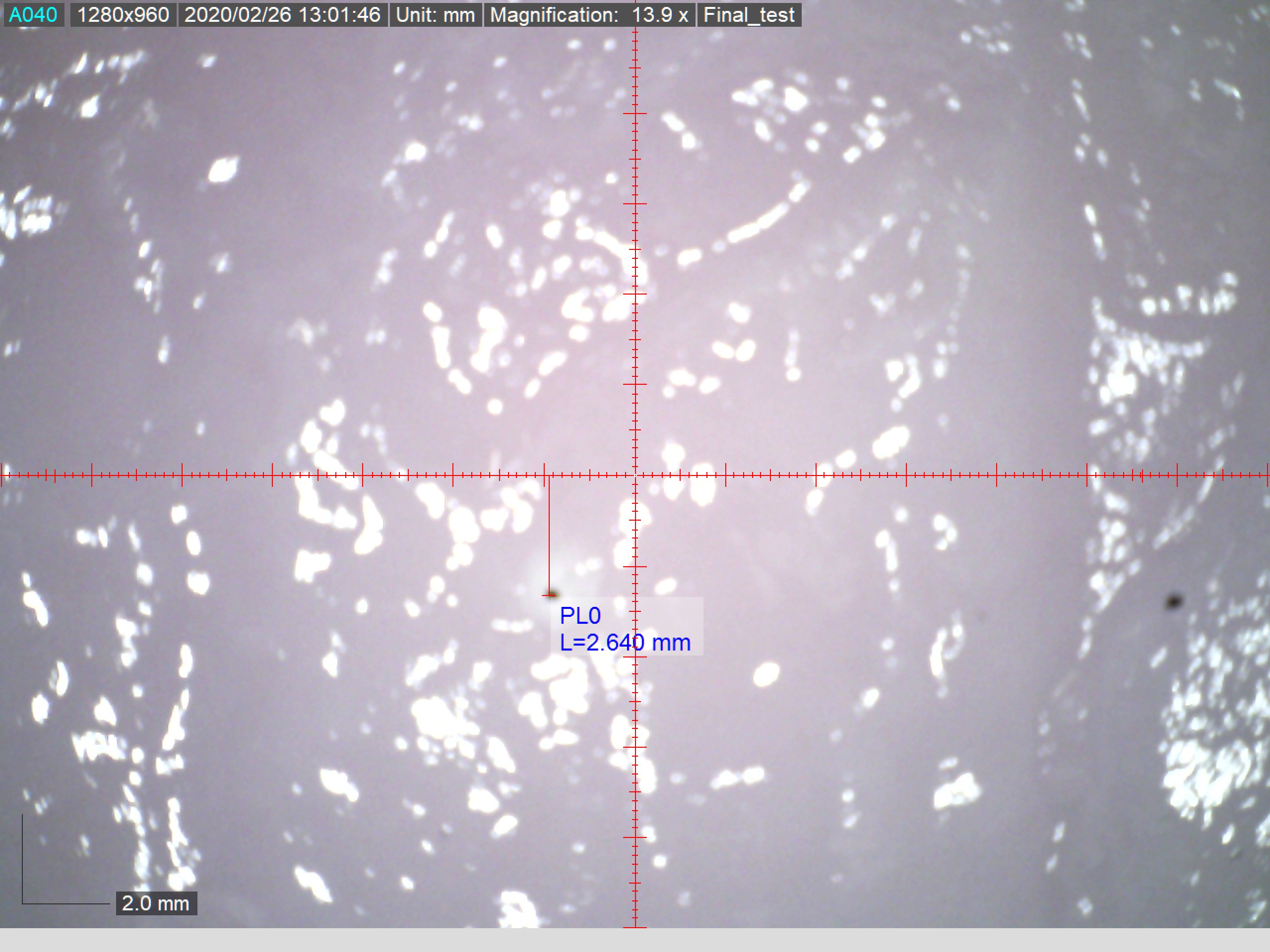

Supplement: S2 File — (ZIP) [file pone.0261089.s002.zip › Soft phantom/photos39.jpg]

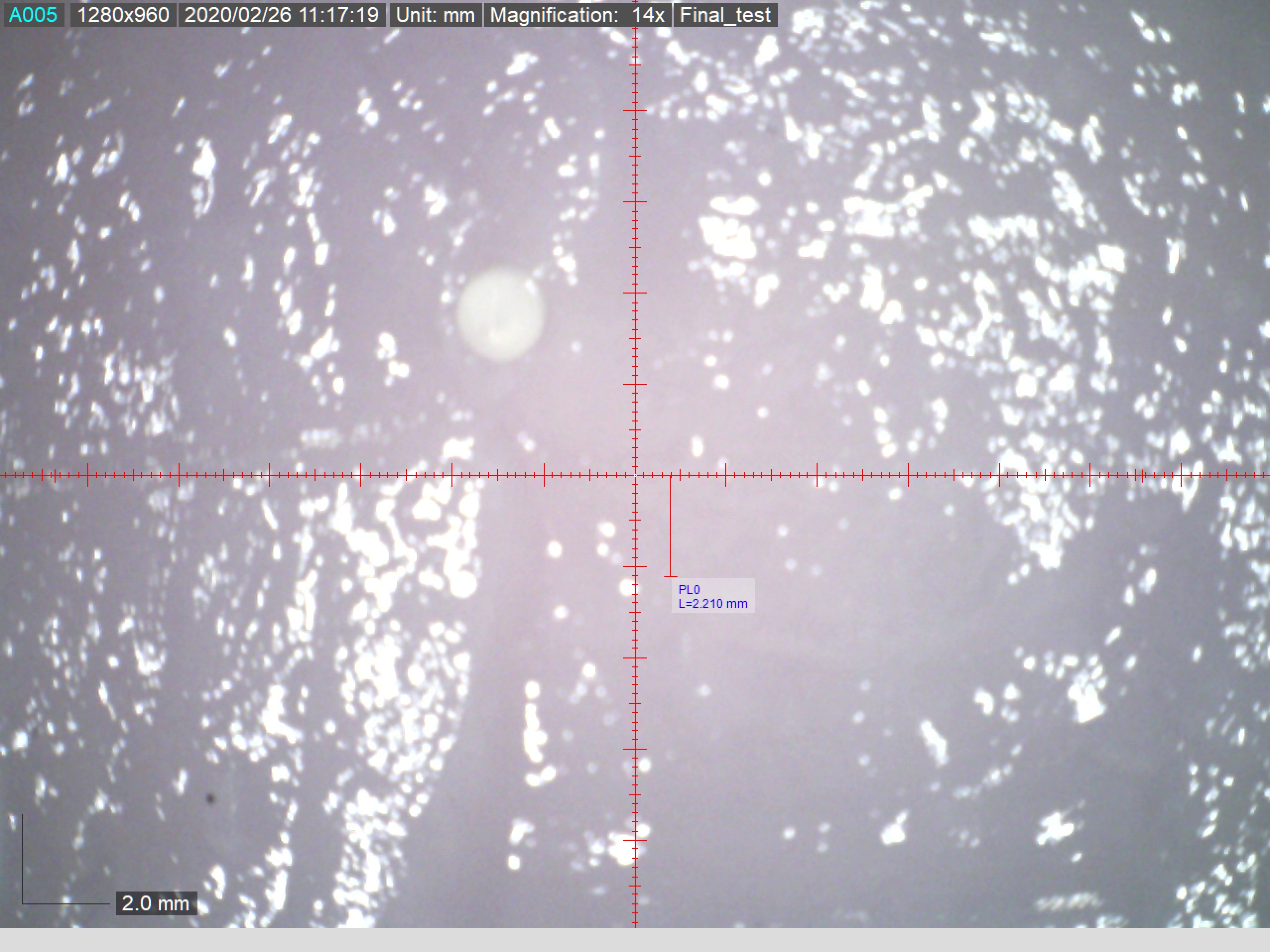

Supplement: S2 File — (ZIP) [file pone.0261089.s002.zip › Soft phantom/photos4.jpg]

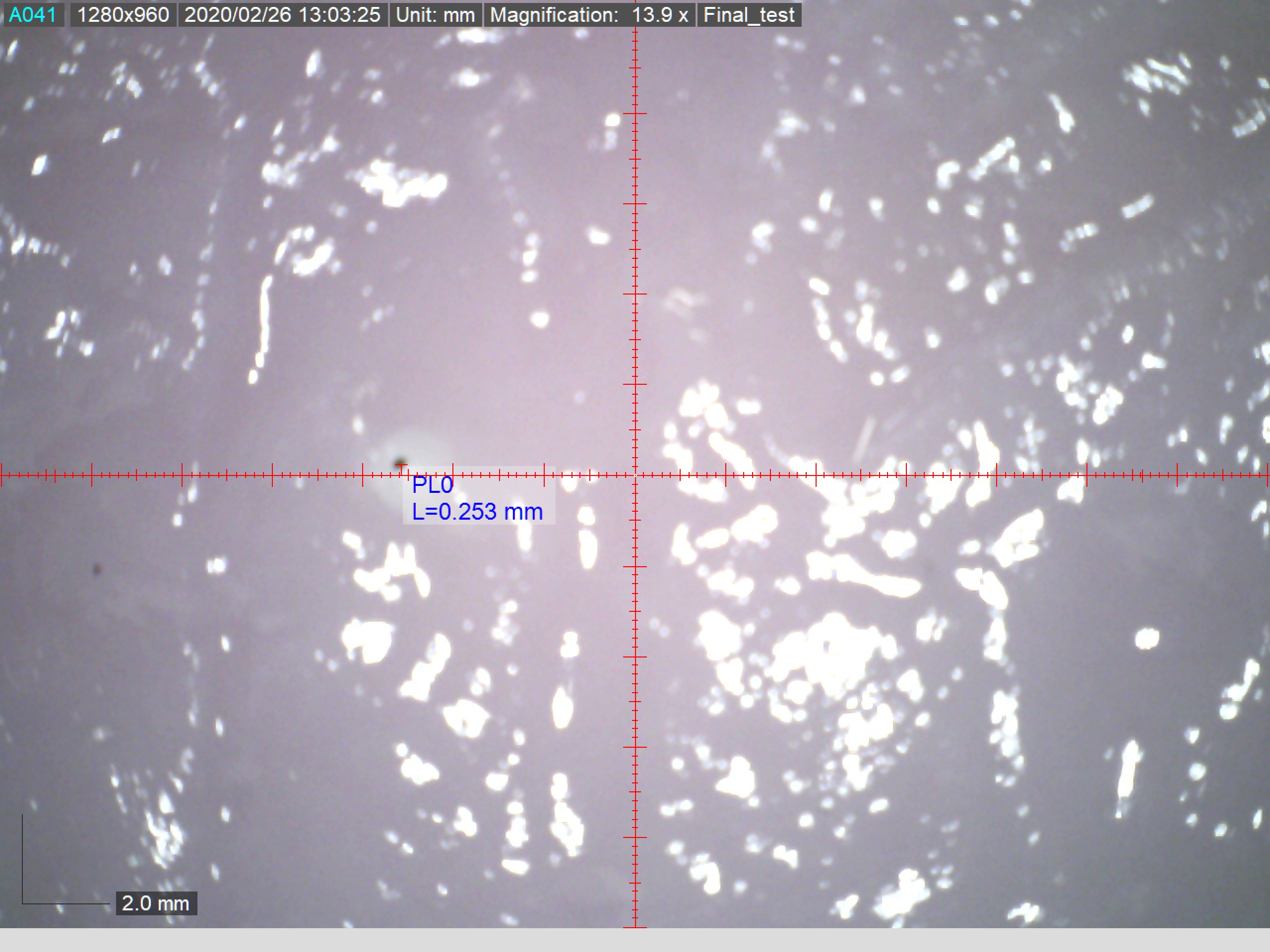

Supplement: S2 File — (ZIP) [file pone.0261089.s002.zip › Soft phantom/photos40.jpg]

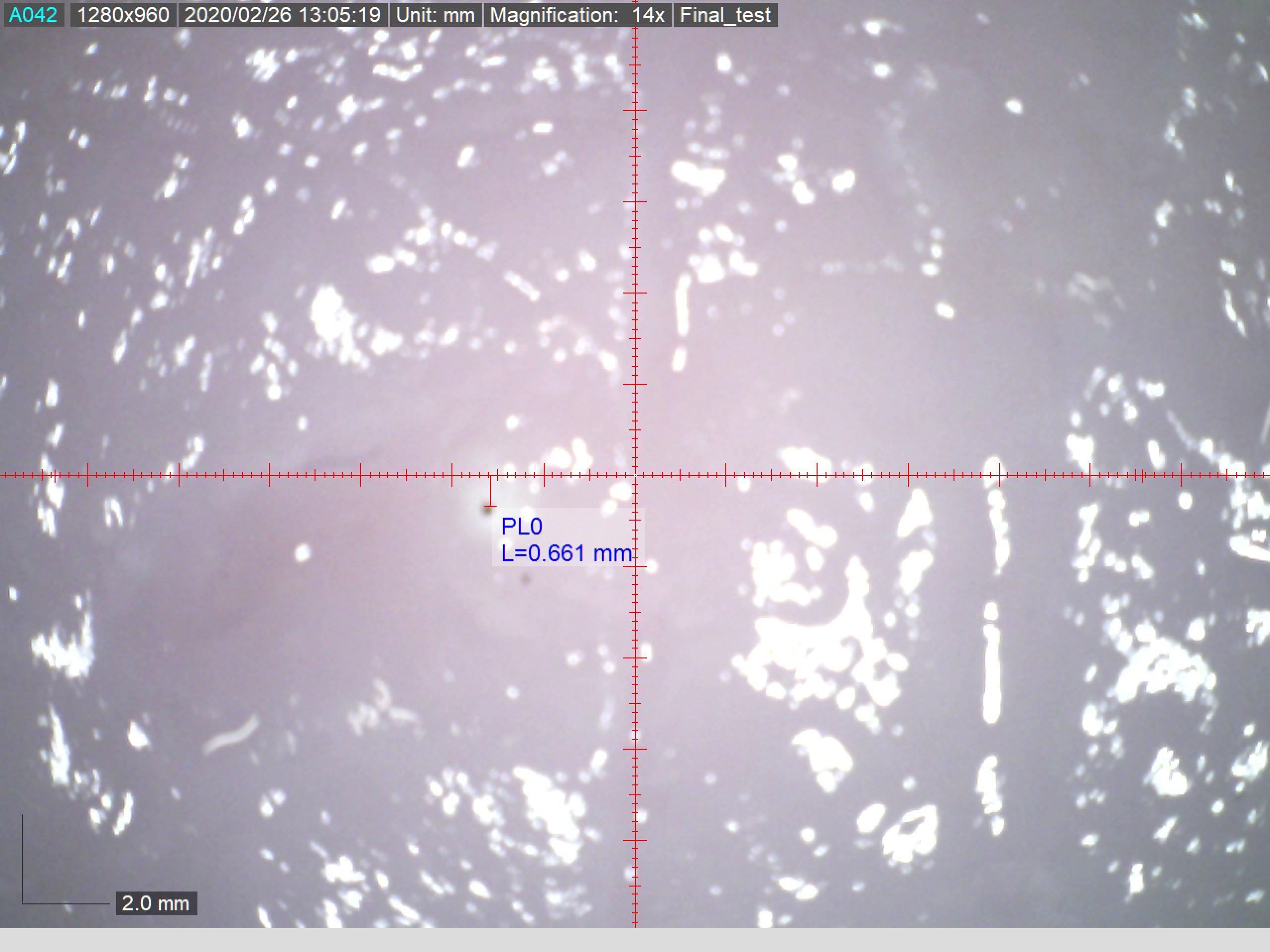

Supplement: S2 File — (ZIP) [file pone.0261089.s002.zip › Soft phantom/photos41.jpg]

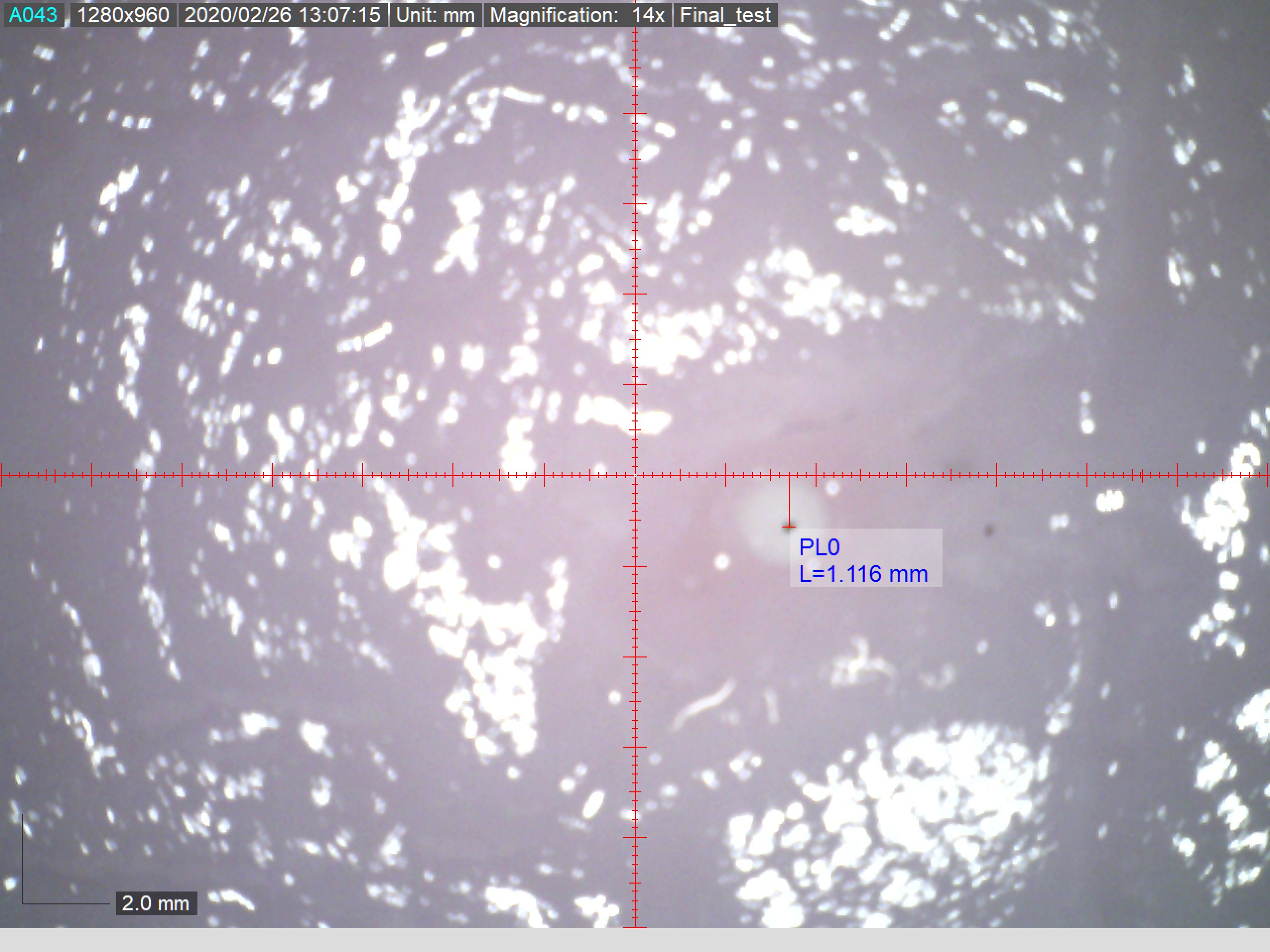

Supplement: S2 File — (ZIP) [file pone.0261089.s002.zip › Soft phantom/photos42.jpg]

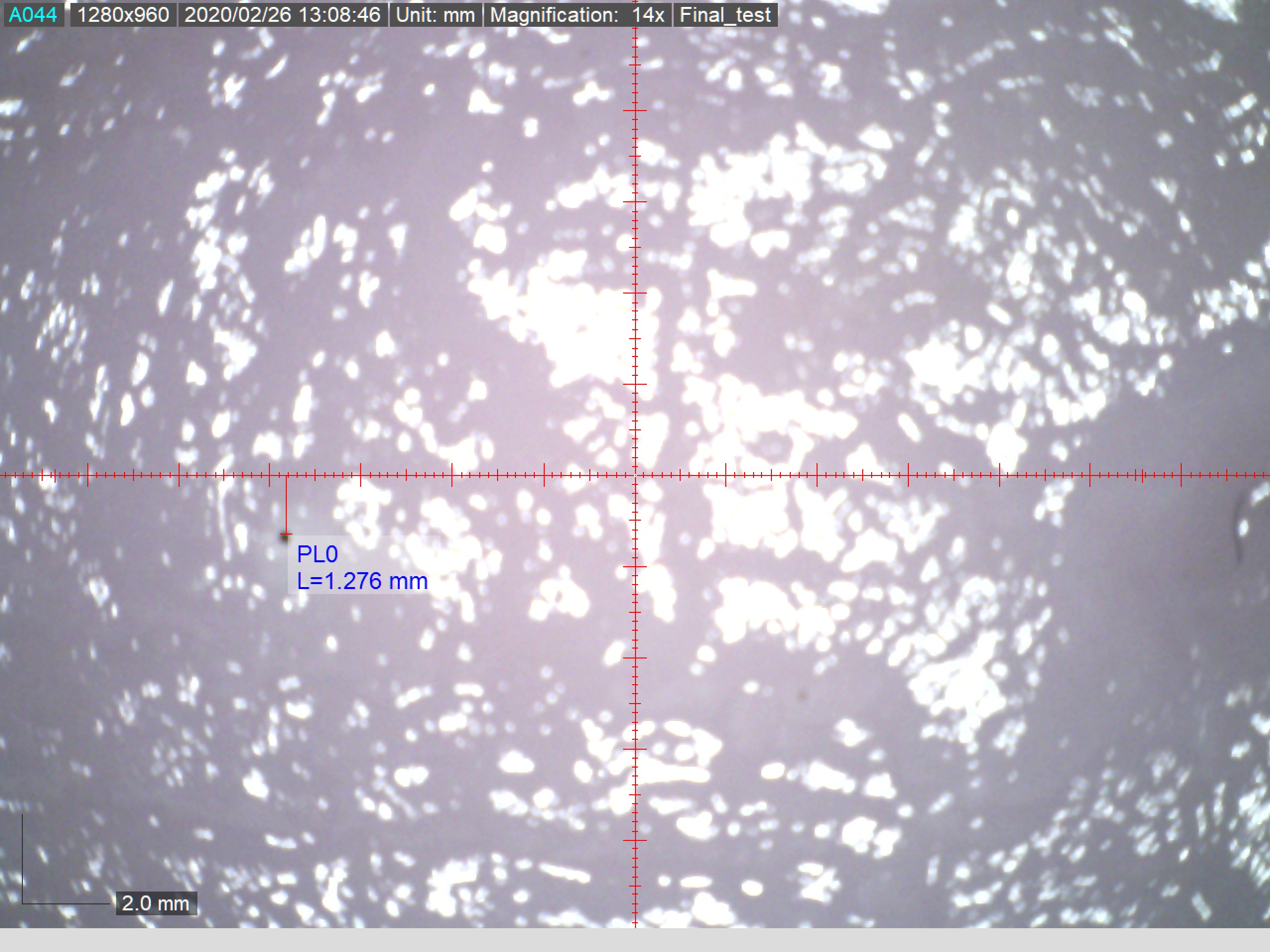

Supplement: S2 File — (ZIP) [file pone.0261089.s002.zip › Soft phantom/photos43.jpg]

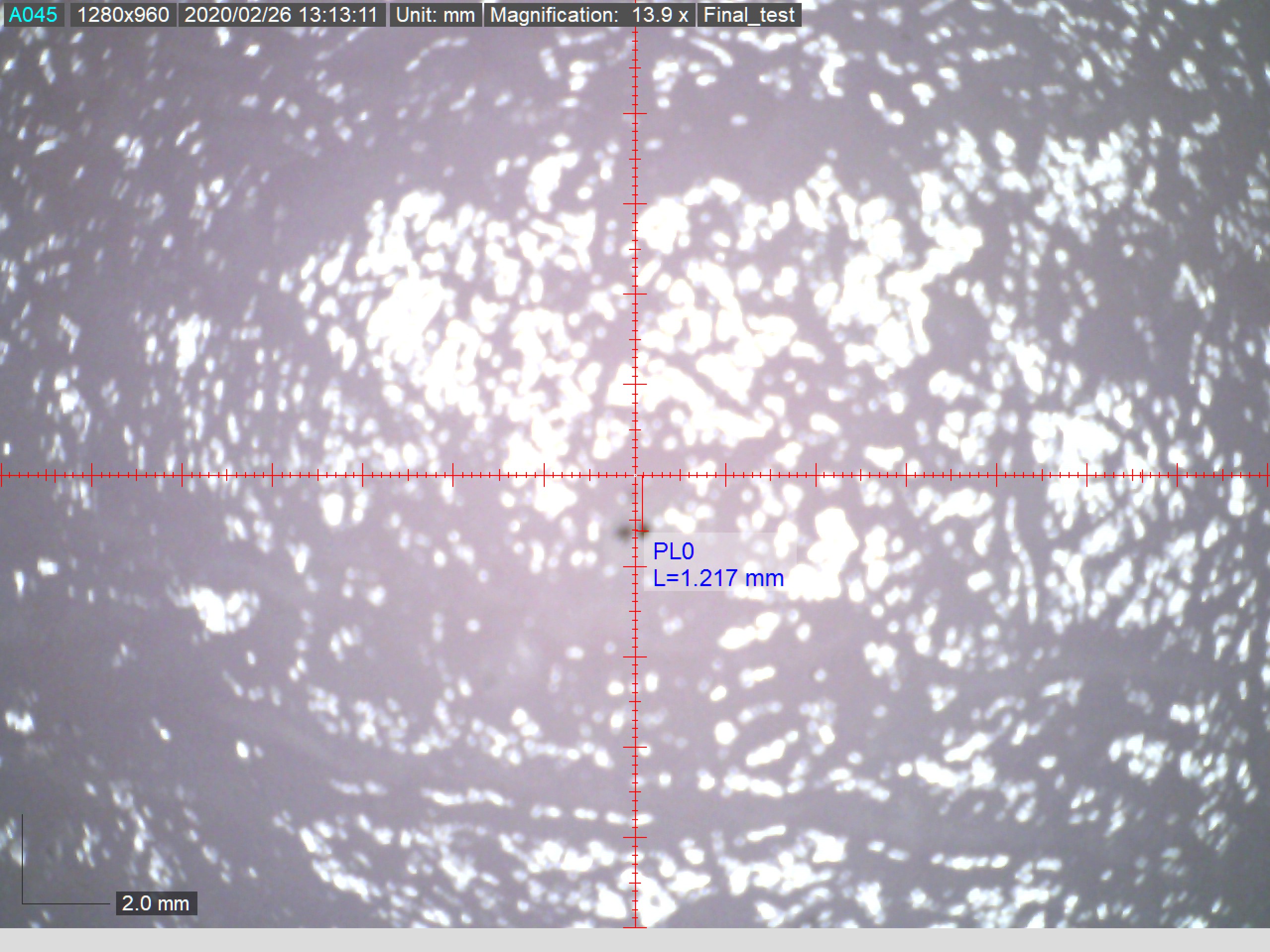

Supplement: S2 File — (ZIP) [file pone.0261089.s002.zip › Soft phantom/photos44.jpg]

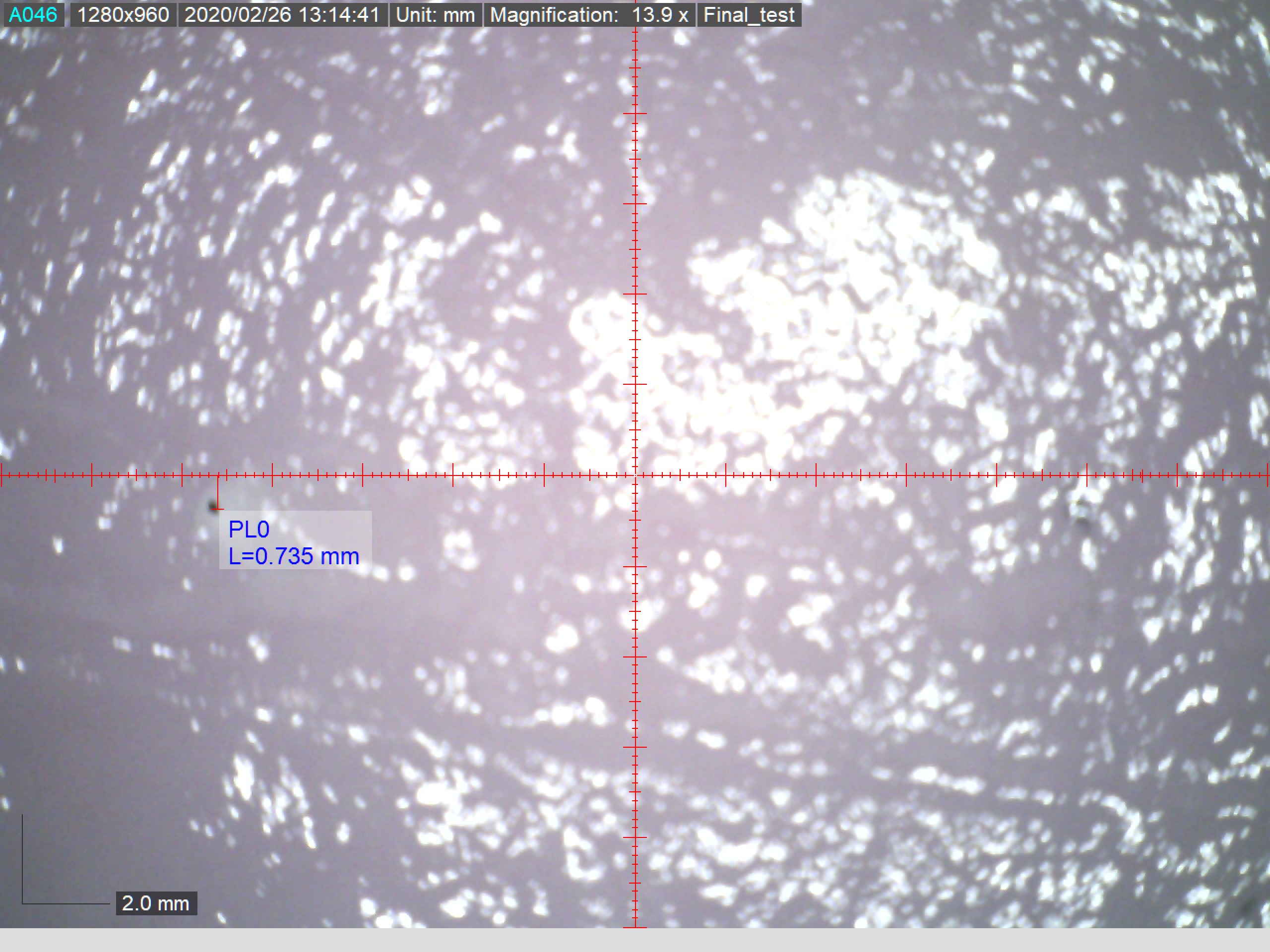

Supplement: S2 File — (ZIP) [file pone.0261089.s002.zip › Soft phantom/photos45.jpg]

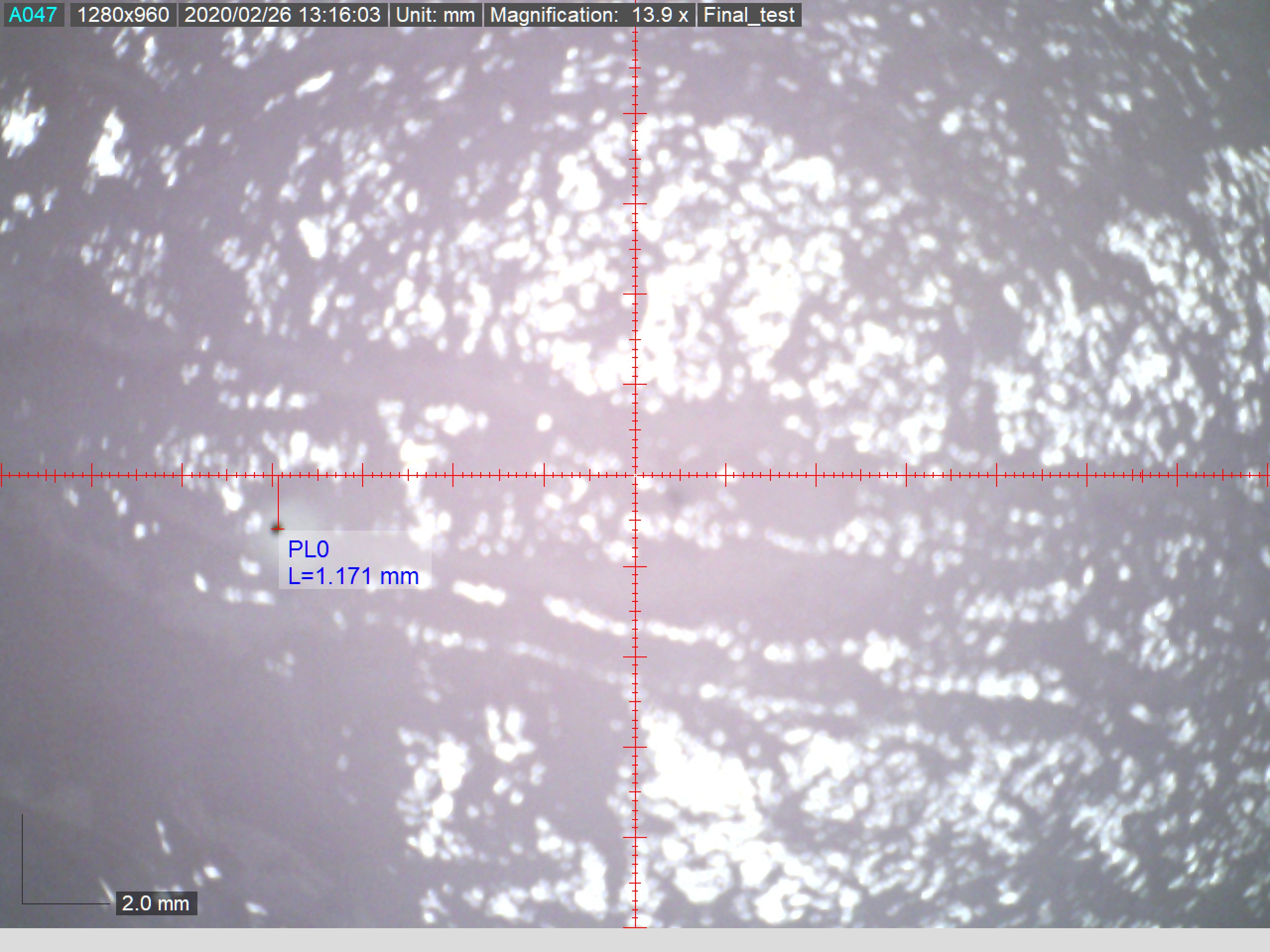

Supplement: S2 File — (ZIP) [file pone.0261089.s002.zip › Soft phantom/photos46.jpg]

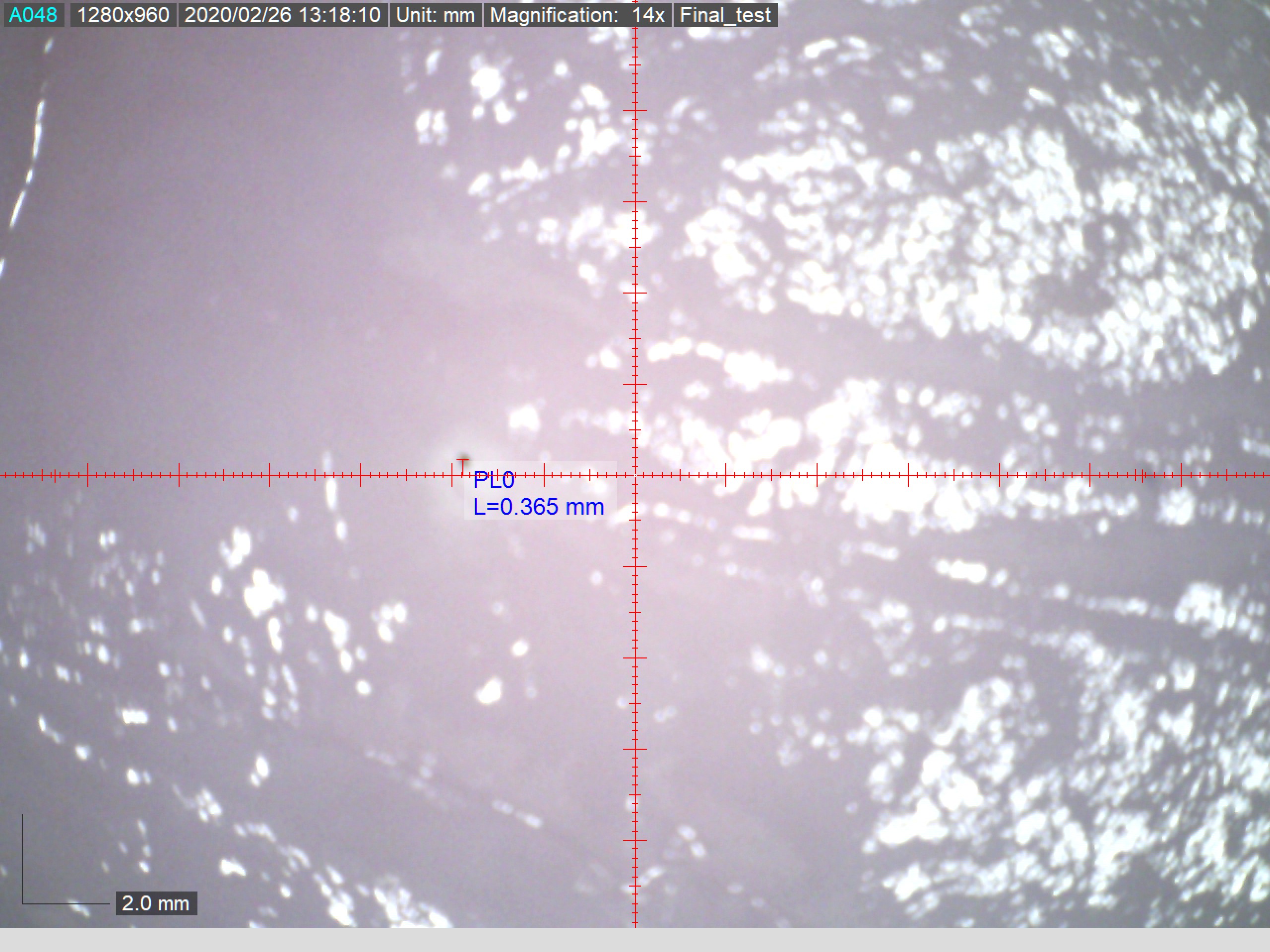

Supplement: S2 File — (ZIP) [file pone.0261089.s002.zip › Soft phantom/photos47.jpg]

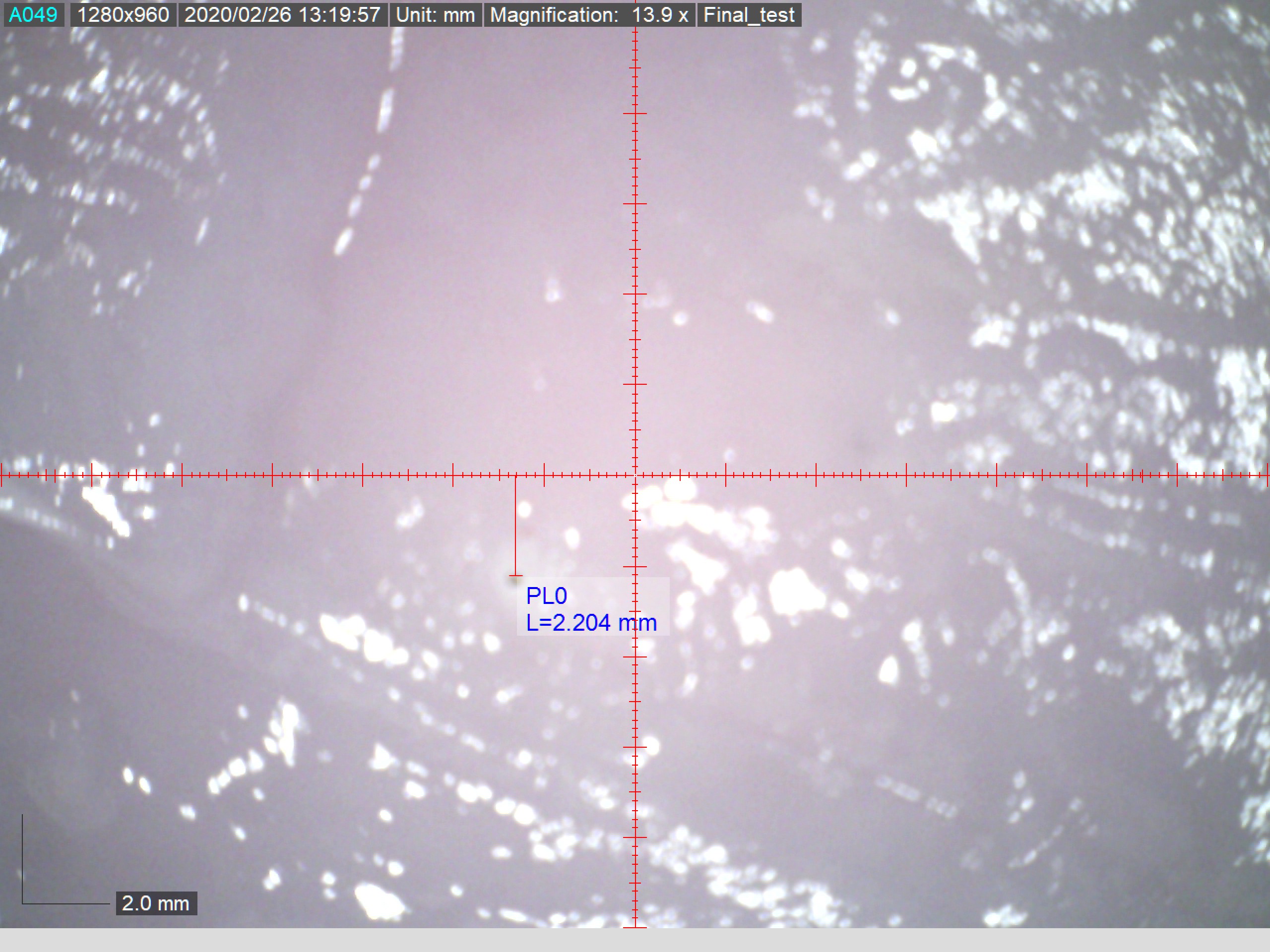

Supplement: S2 File — (ZIP) [file pone.0261089.s002.zip › Soft phantom/photos48.jpg]

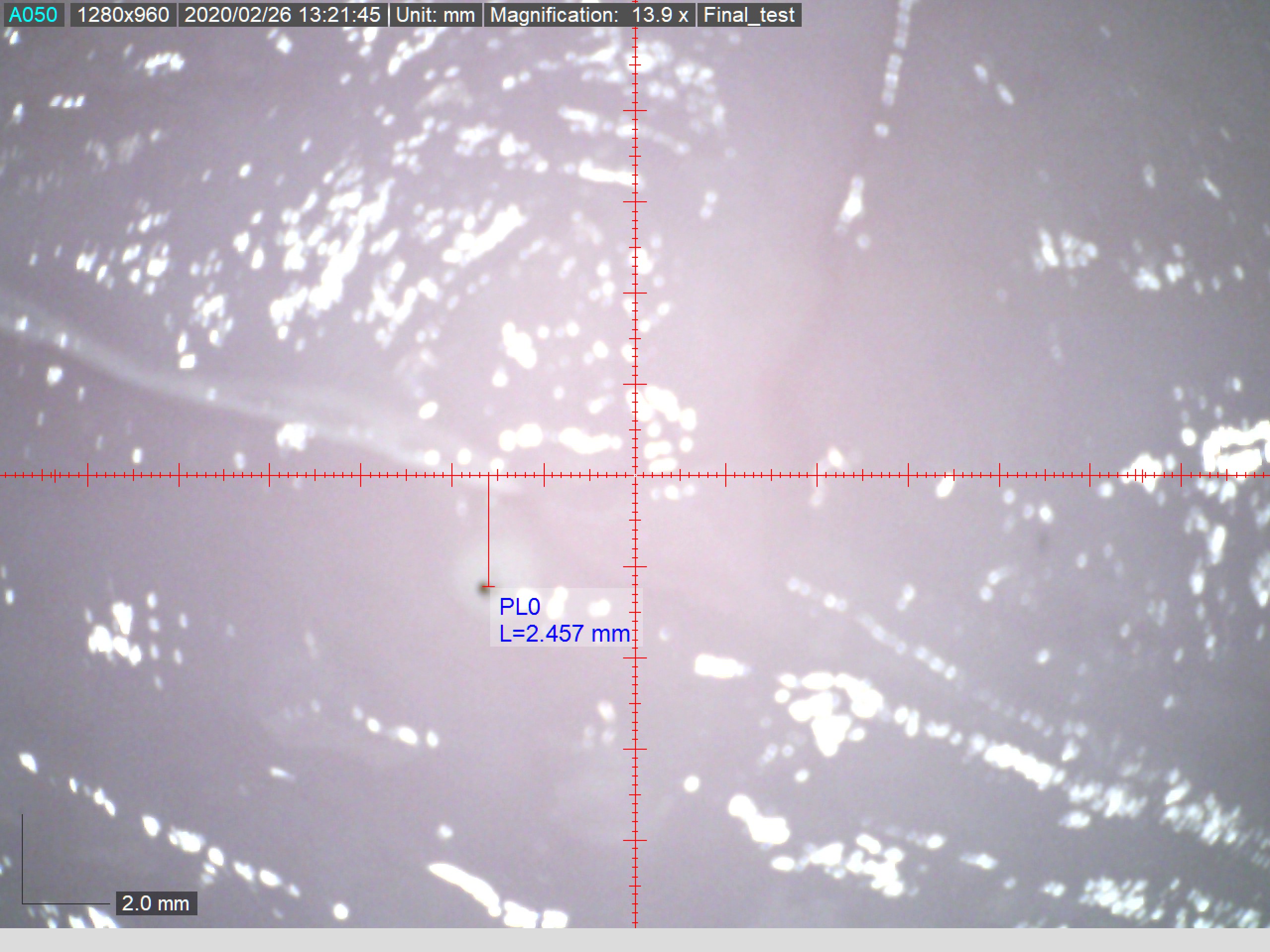

Supplement: S2 File — (ZIP) [file pone.0261089.s002.zip › Soft phantom/photos49.jpg]

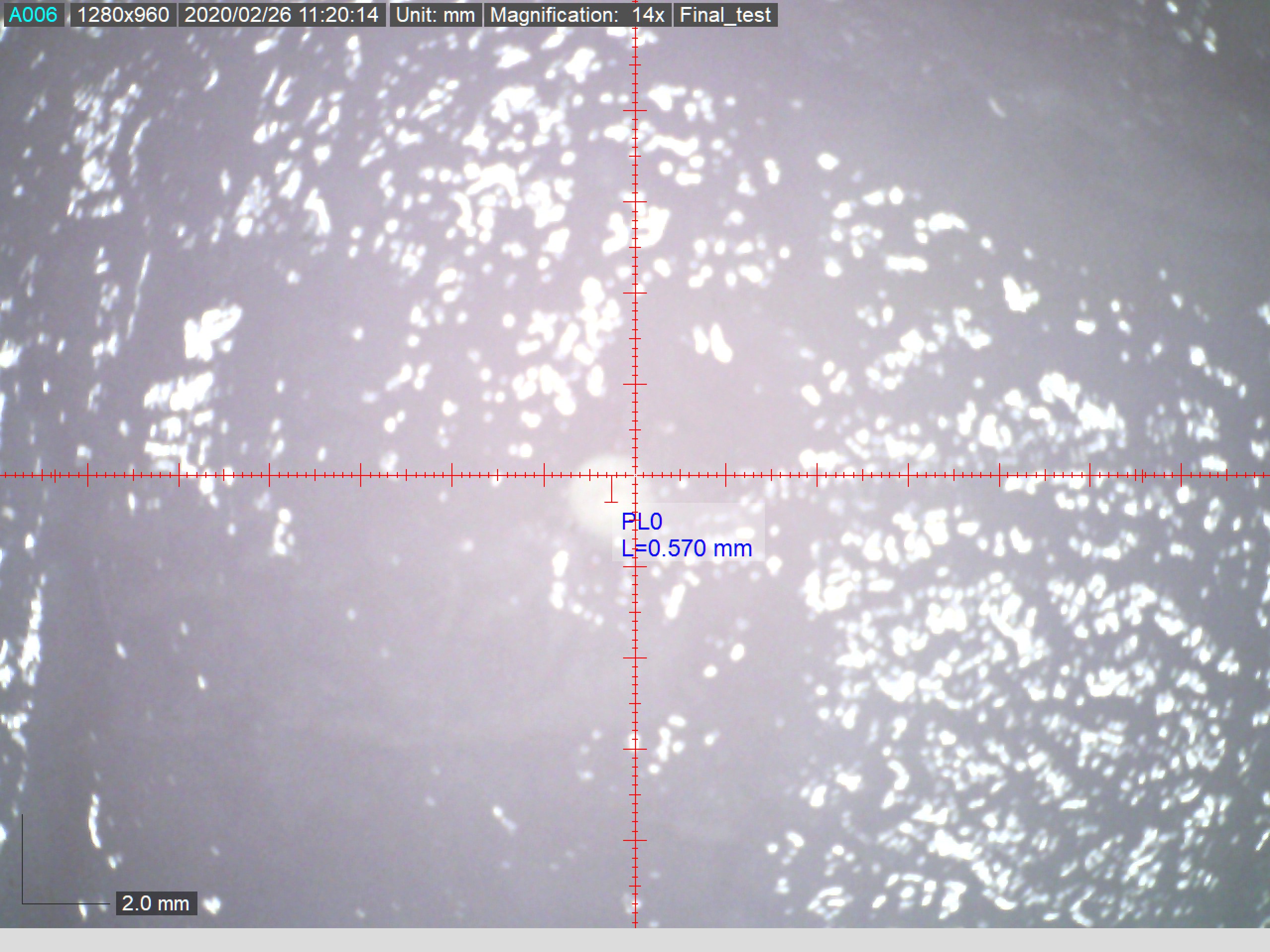

Supplement: S2 File — (ZIP) [file pone.0261089.s002.zip › Soft phantom/photos5.jpg]

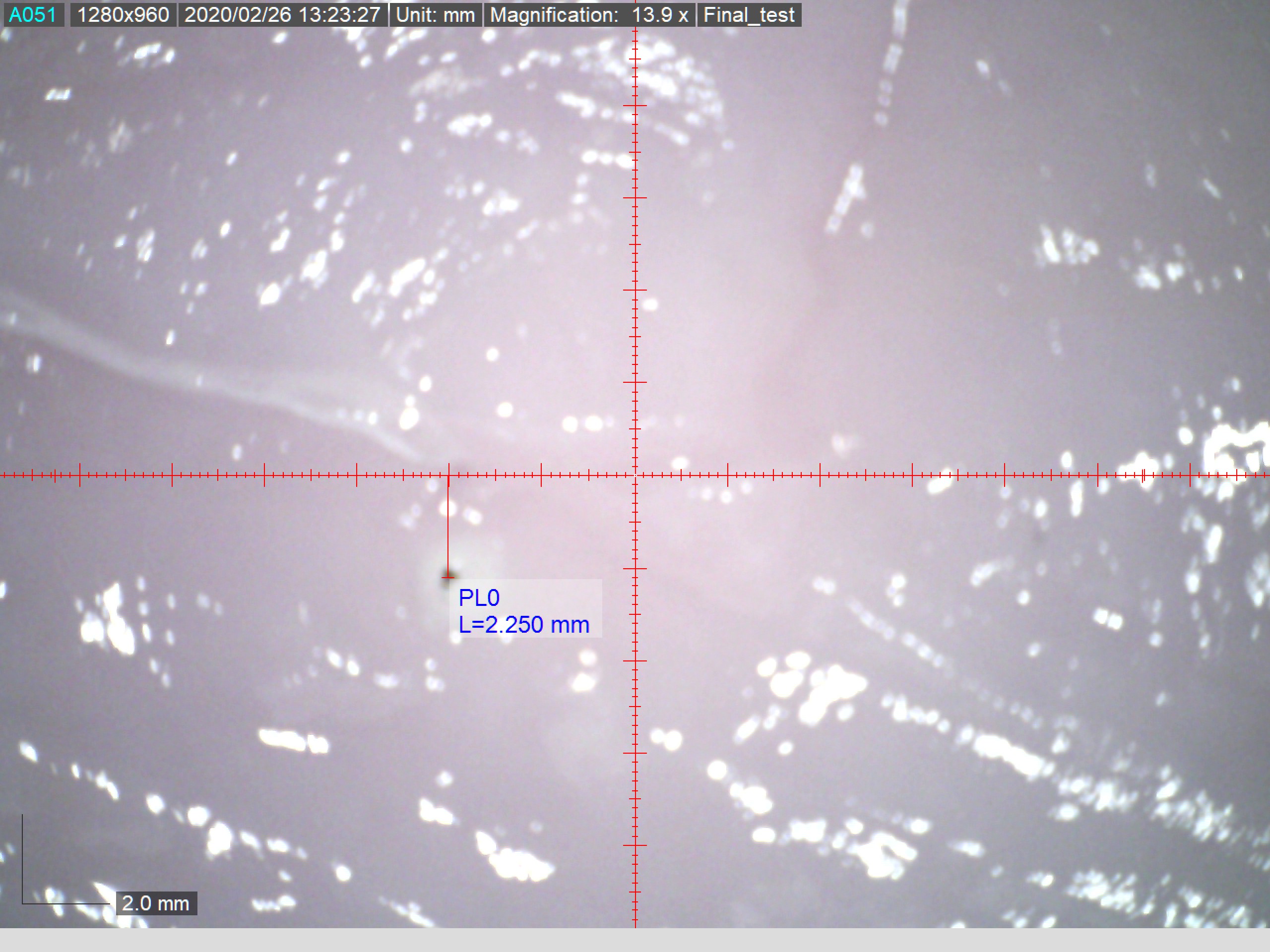

Supplement: S2 File — (ZIP) [file pone.0261089.s002.zip › Soft phantom/photos50.jpg]

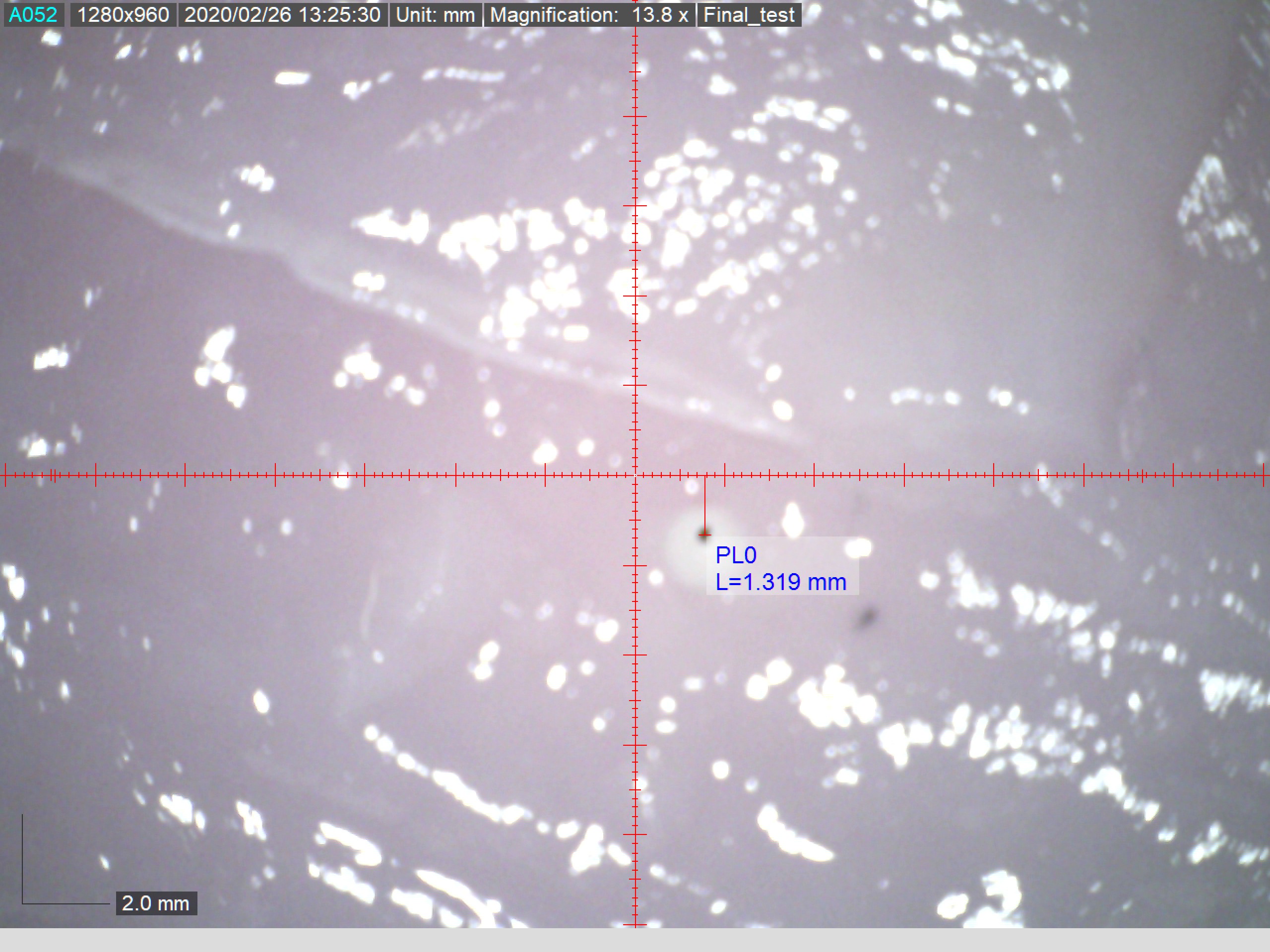

Supplement: S2 File — (ZIP) [file pone.0261089.s002.zip › Soft phantom/photos51.jpg]

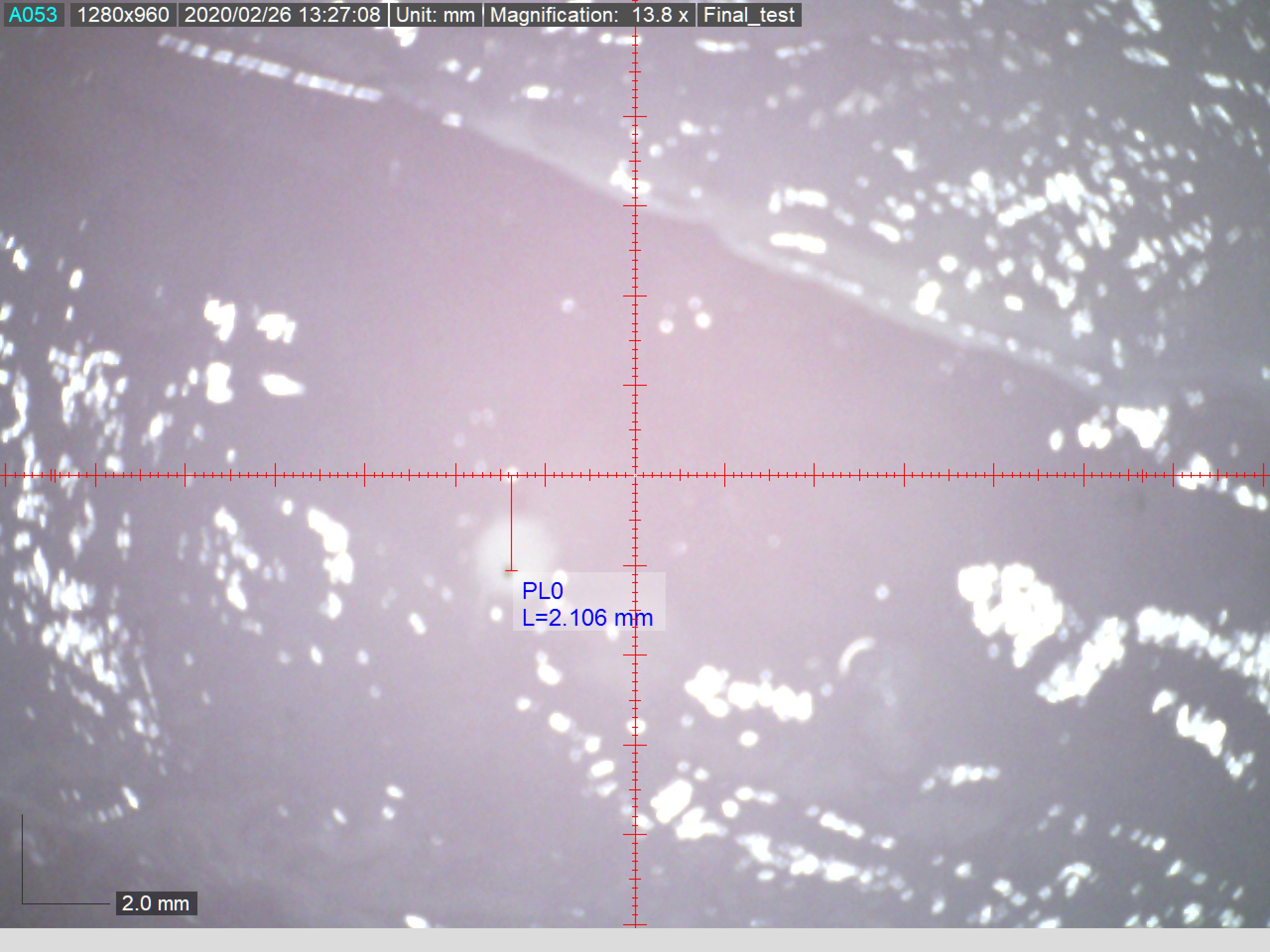

Supplement: S2 File — (ZIP) [file pone.0261089.s002.zip › Soft phantom/photos52.jpg]

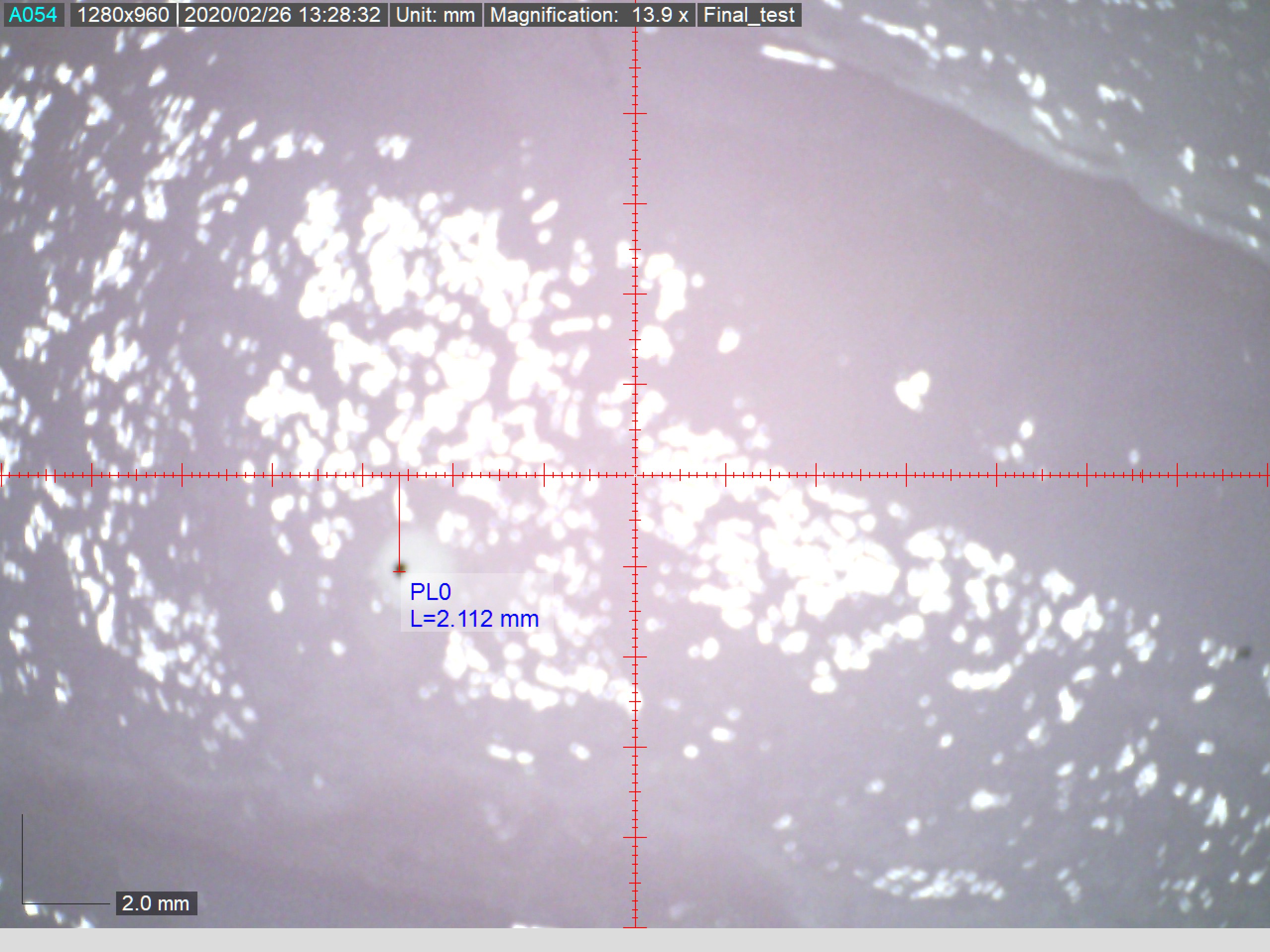

Supplement: S2 File — (ZIP) [file pone.0261089.s002.zip › Soft phantom/photos53.jpg]

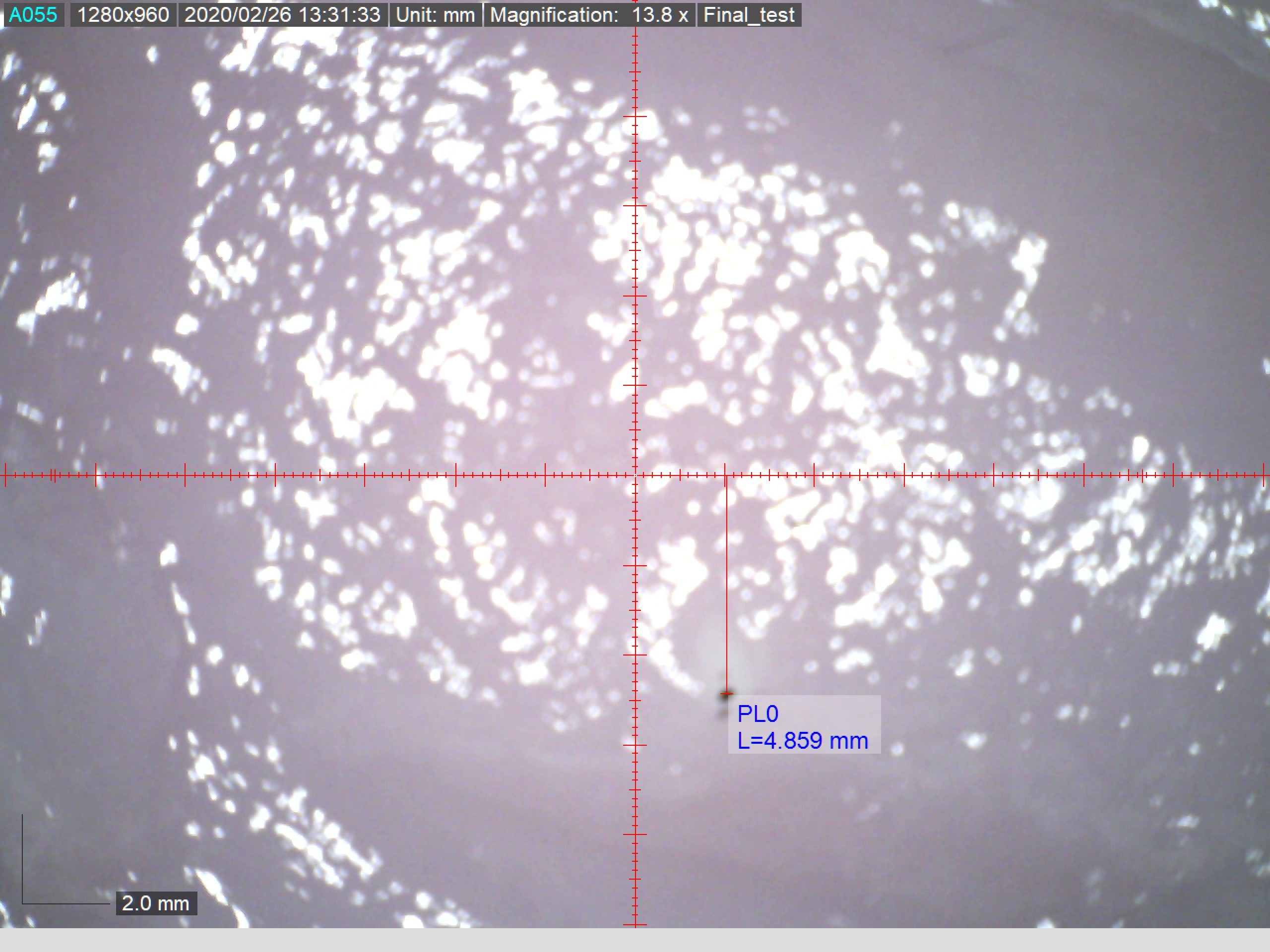

Supplement: S2 File — (ZIP) [file pone.0261089.s002.zip › Soft phantom/photos54.jpg]

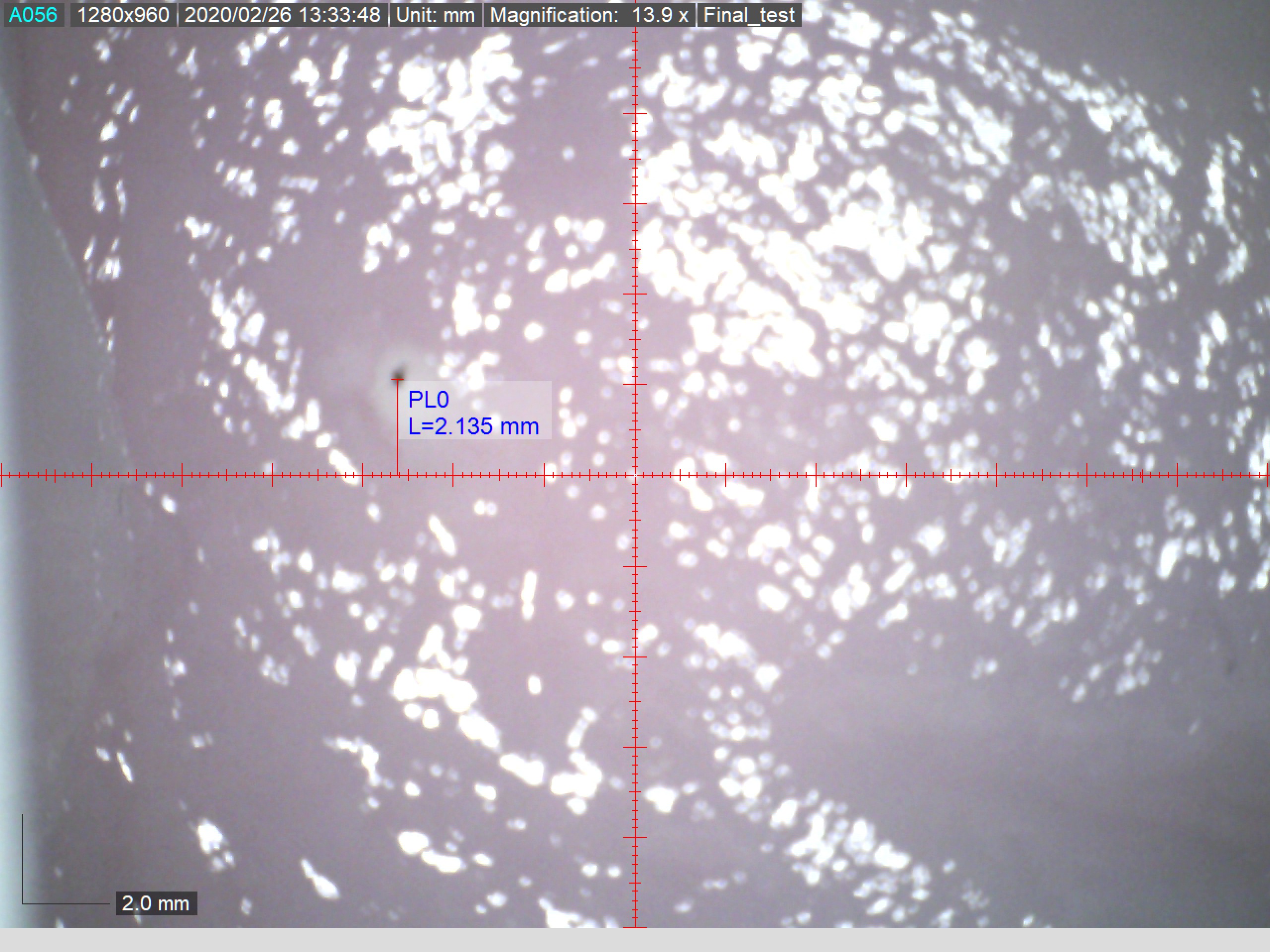

Supplement: S2 File — (ZIP) [file pone.0261089.s002.zip › Soft phantom/photos55.jpg]

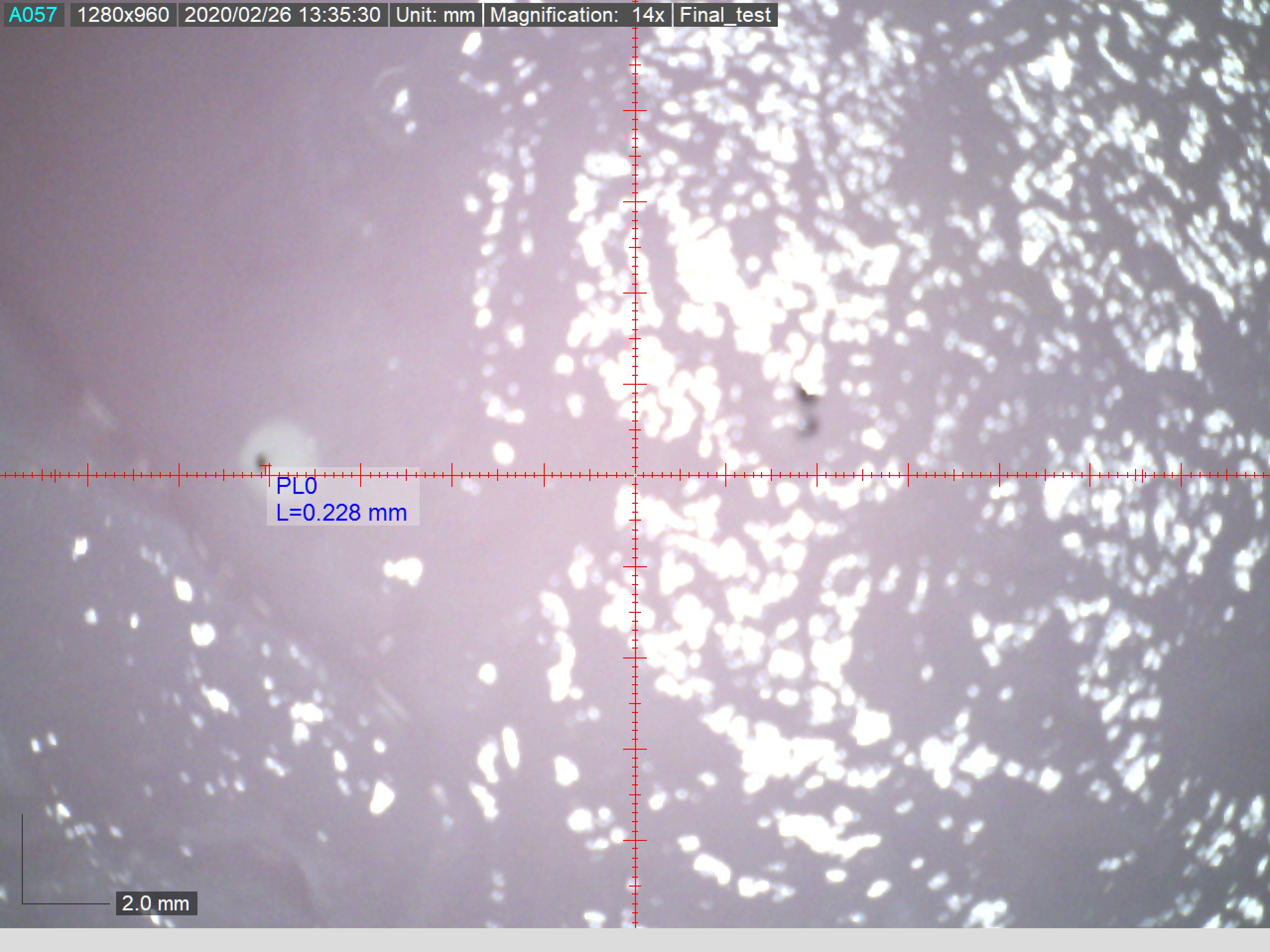

Supplement: S2 File — (ZIP) [file pone.0261089.s002.zip › Soft phantom/photos56.jpg]

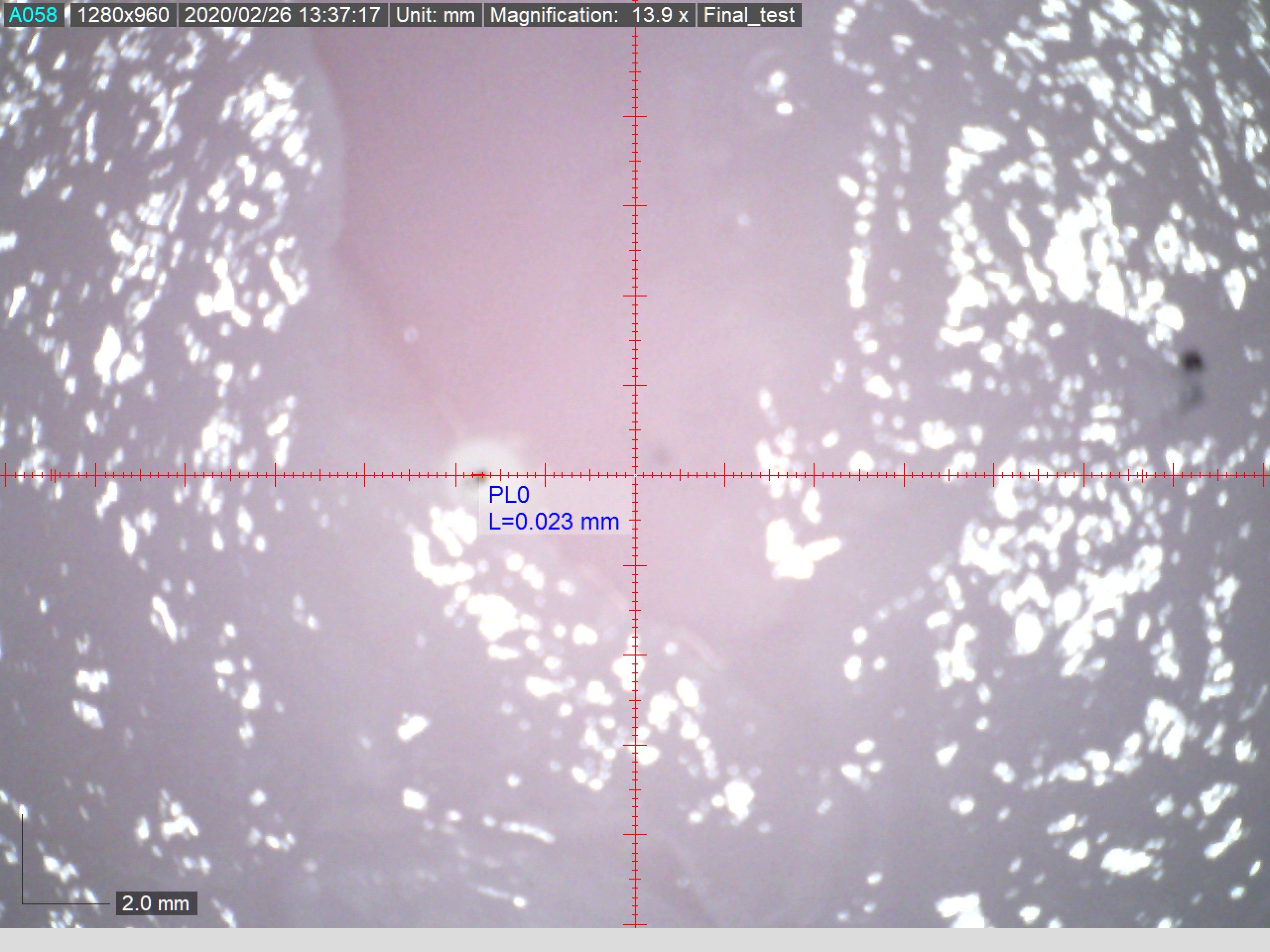

Supplement: S2 File — (ZIP) [file pone.0261089.s002.zip › Soft phantom/photos57.jpg]

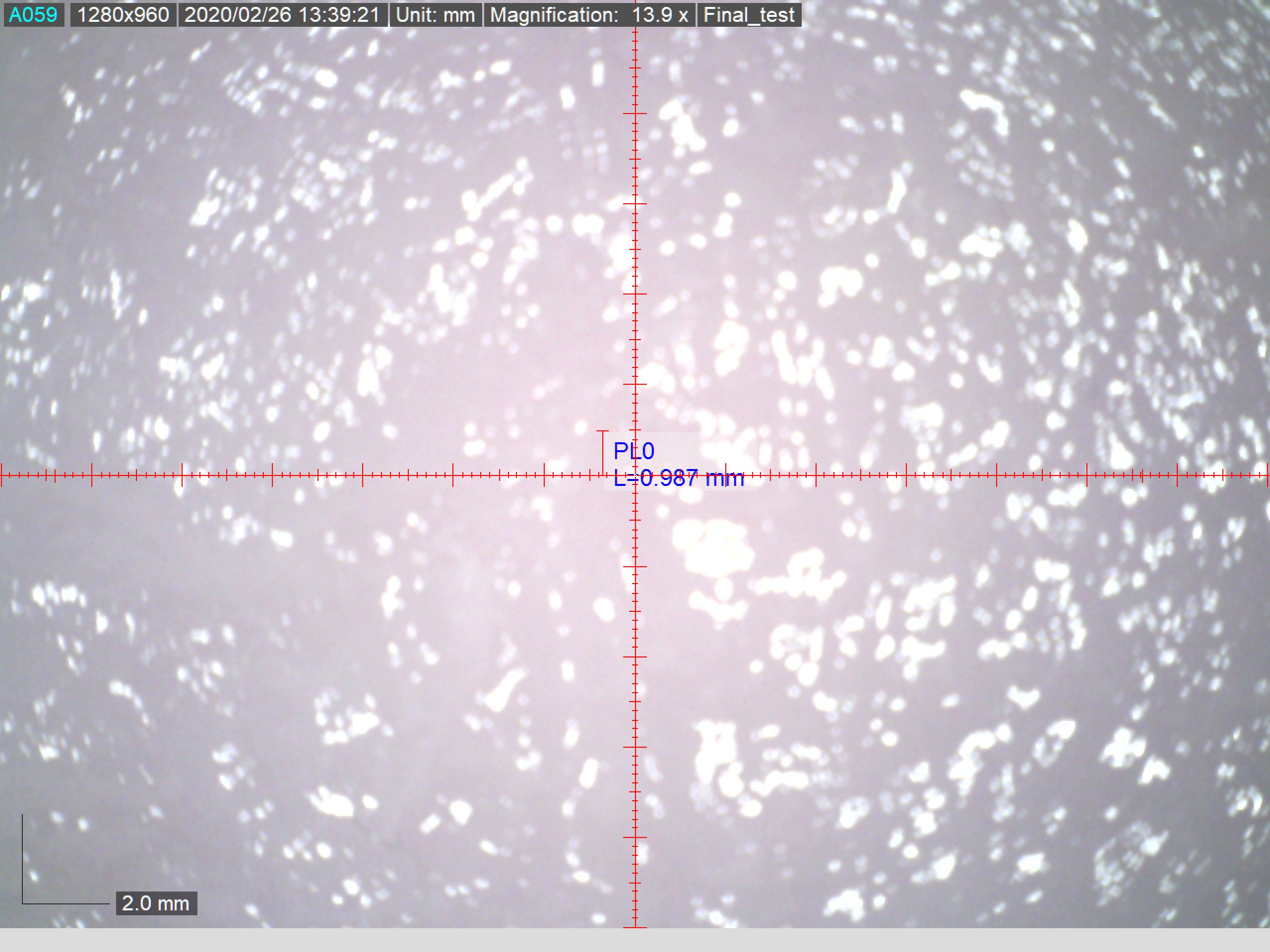

Supplement: S2 File — (ZIP) [file pone.0261089.s002.zip › Soft phantom/photos58.jpg]

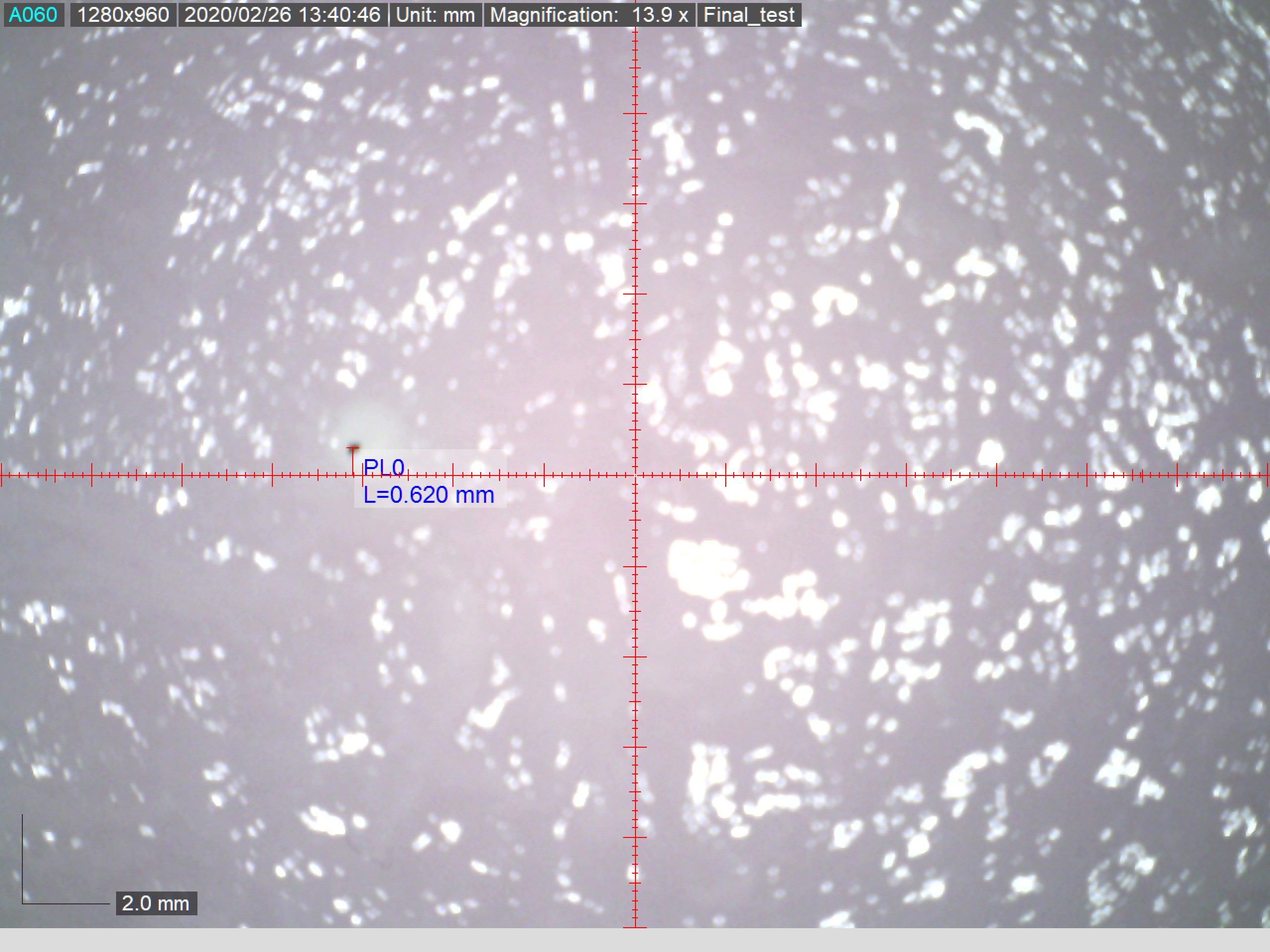

Supplement: S2 File — (ZIP) [file pone.0261089.s002.zip › Soft phantom/photos59.jpg]

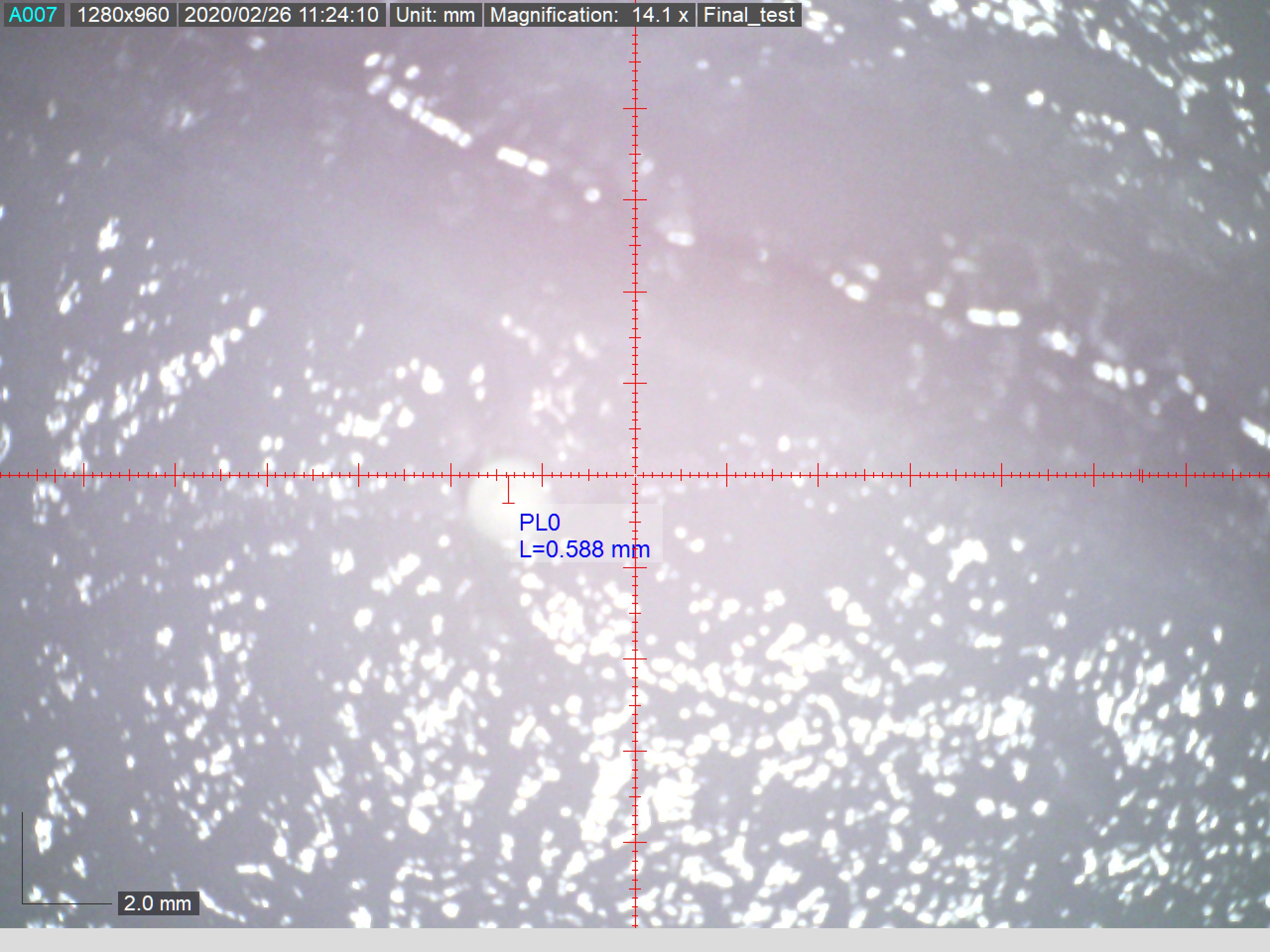

Supplement: S2 File — (ZIP) [file pone.0261089.s002.zip › Soft phantom/photos6.jpg]

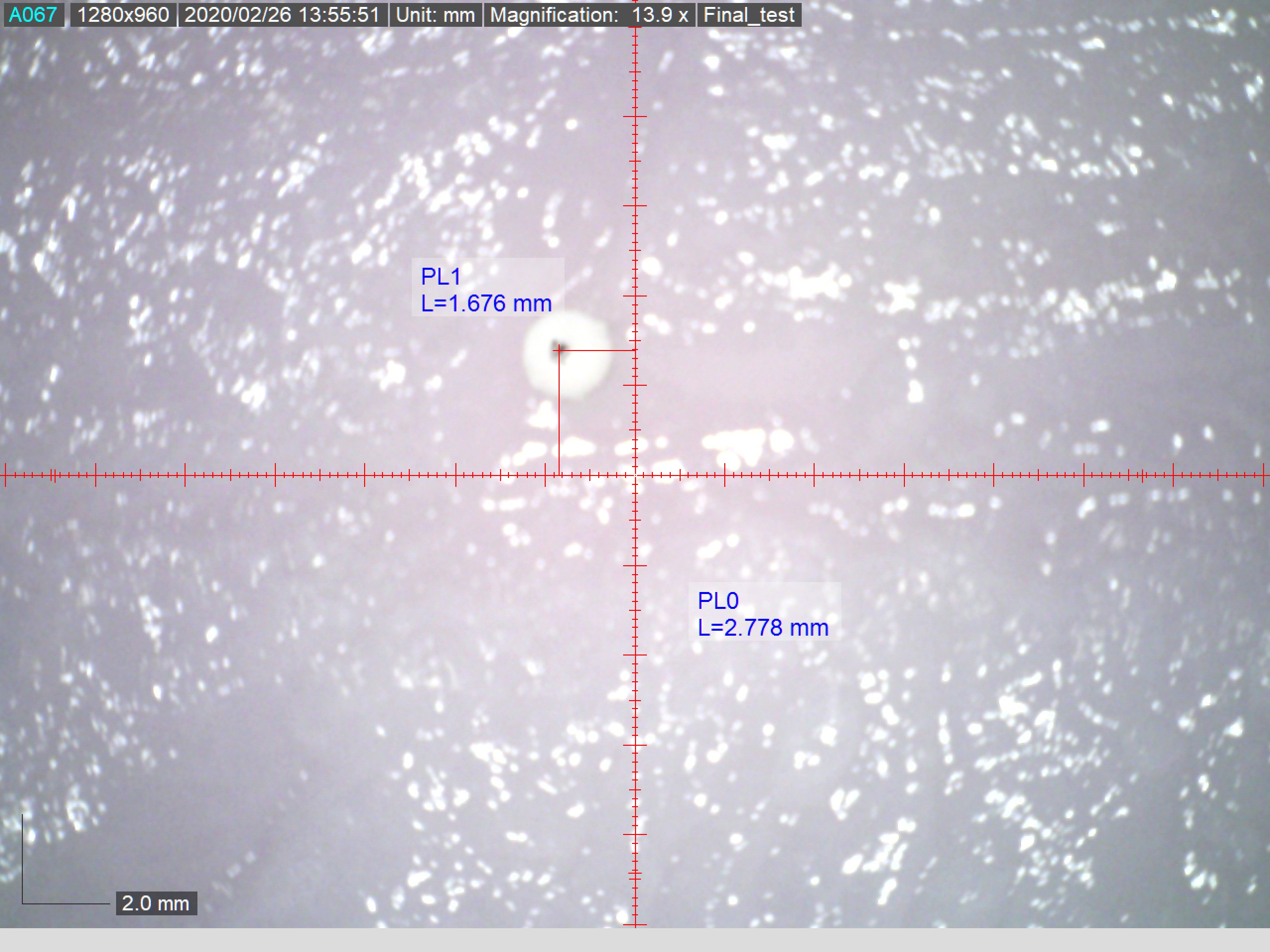

Supplement: S2 File — (ZIP) [file pone.0261089.s002.zip › Soft phantom/photos60.jpg]

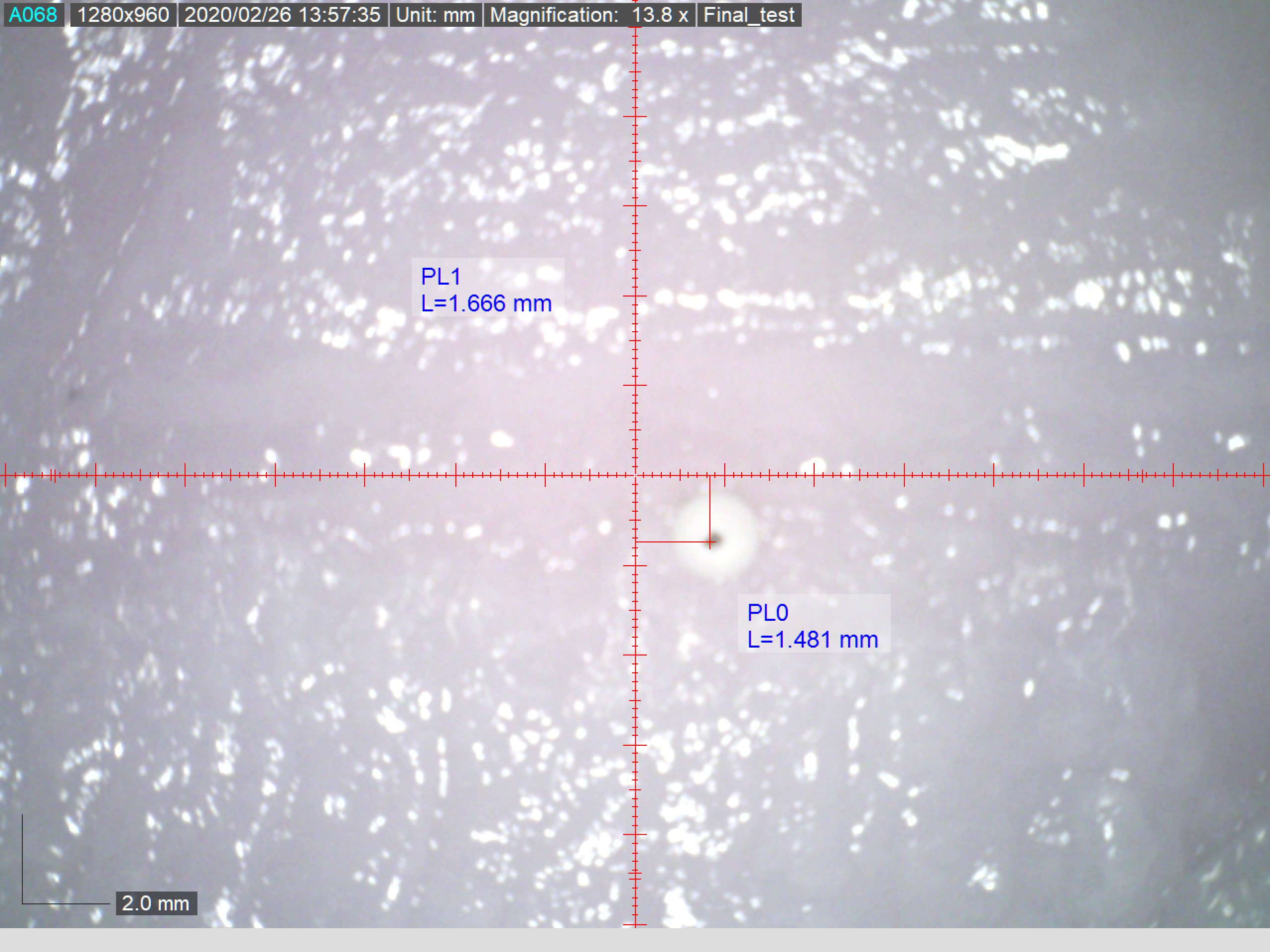

Supplement: S2 File — (ZIP) [file pone.0261089.s002.zip › Soft phantom/photos61.jpg]

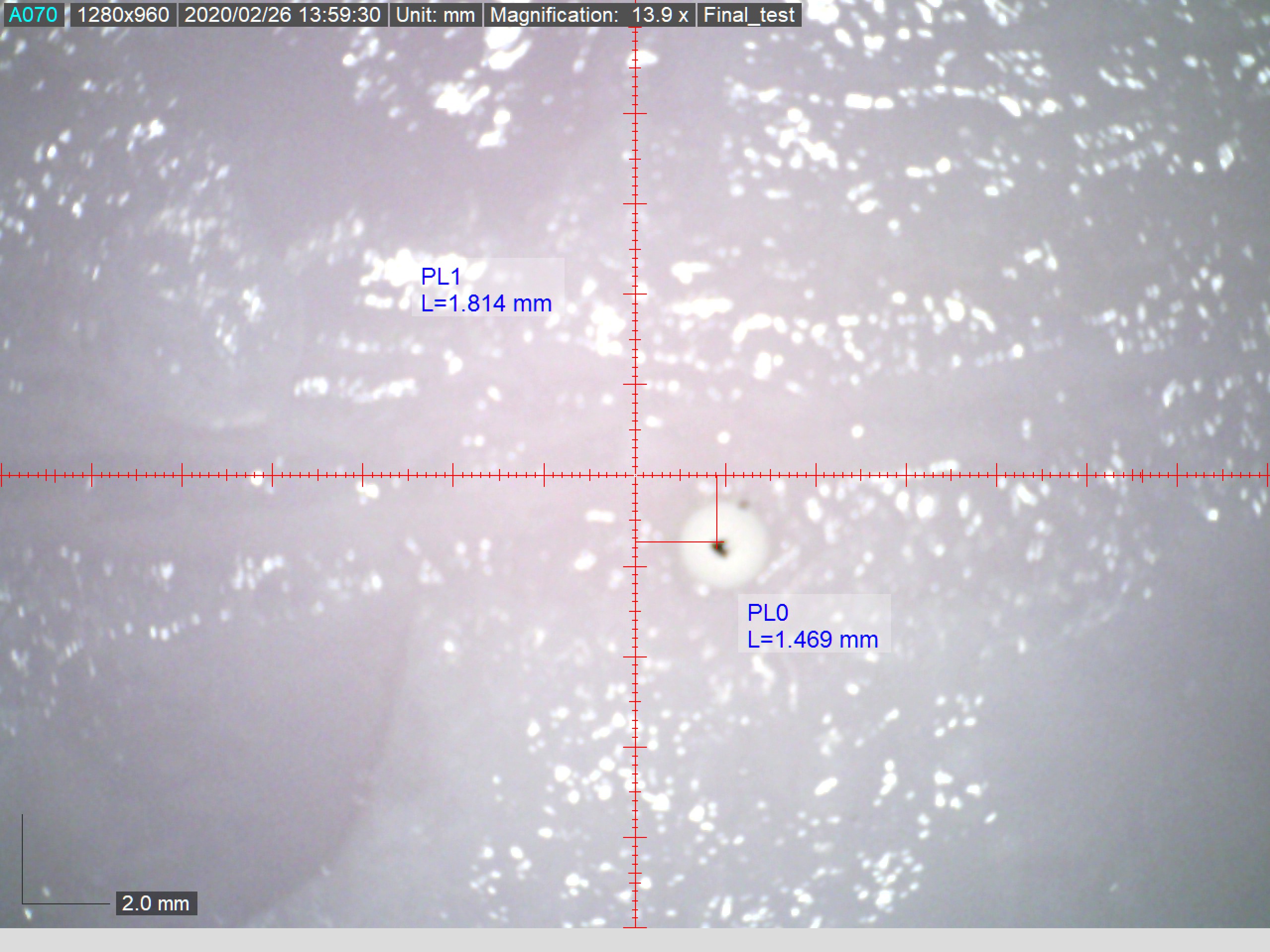

Supplement: S2 File — (ZIP) [file pone.0261089.s002.zip › Soft phantom/photos63.jpg]

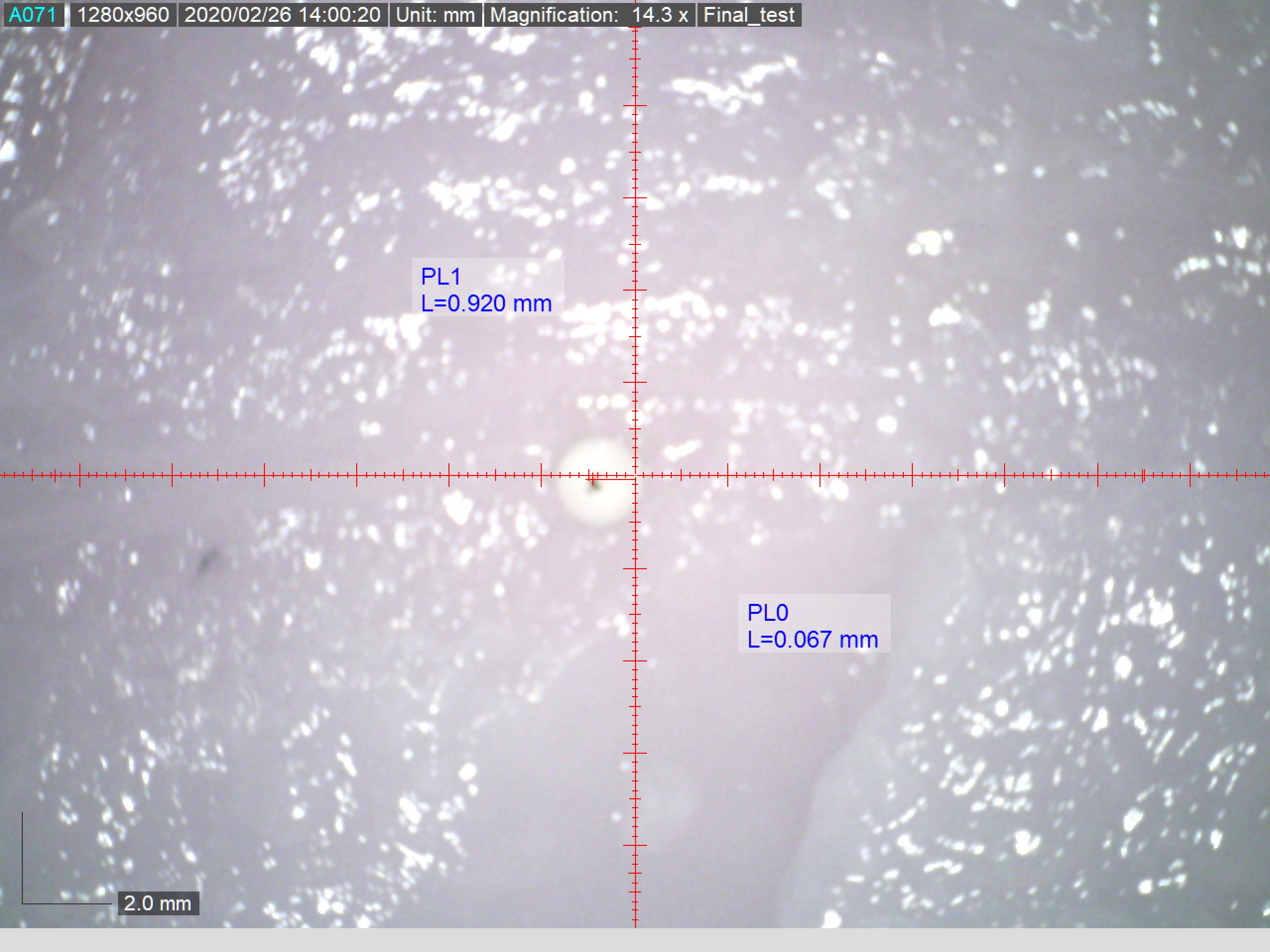

Supplement: S2 File — (ZIP) [file pone.0261089.s002.zip › Soft phantom/photos64.jpg]

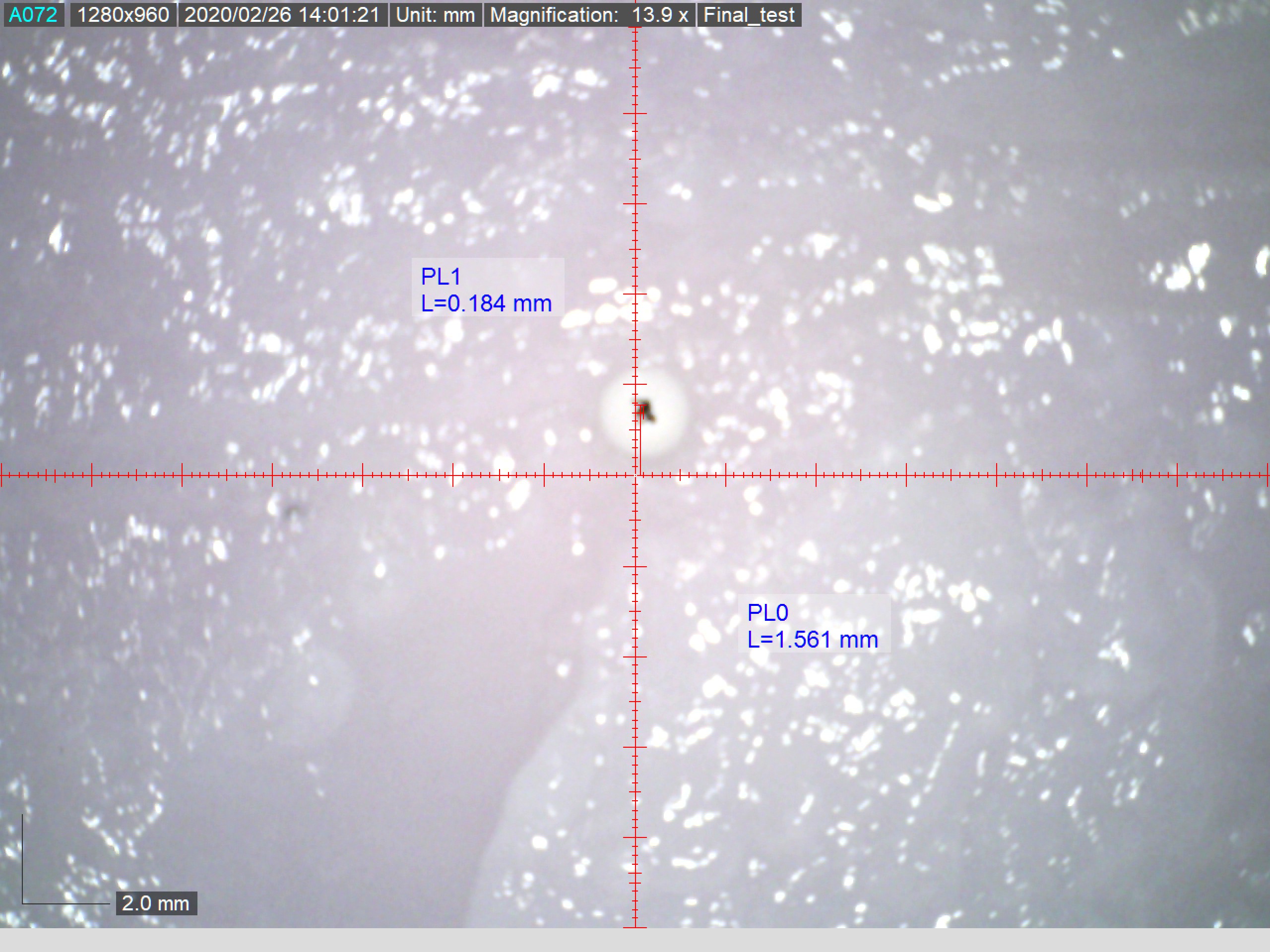

Supplement: S2 File — (ZIP) [file pone.0261089.s002.zip › Soft phantom/photos65.jpg]

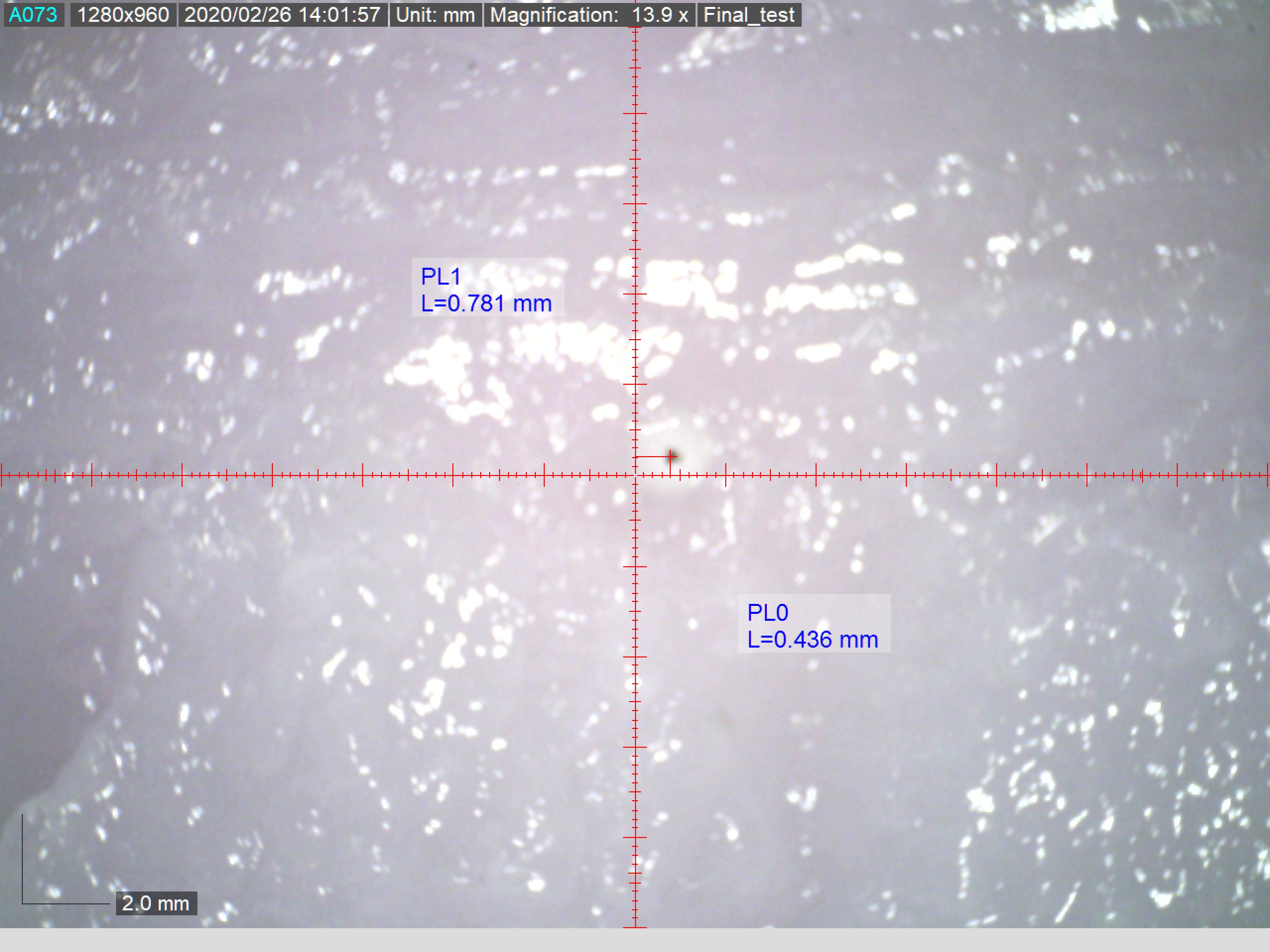

Supplement: S2 File — (ZIP) [file pone.0261089.s002.zip › Soft phantom/photos66.jpg]

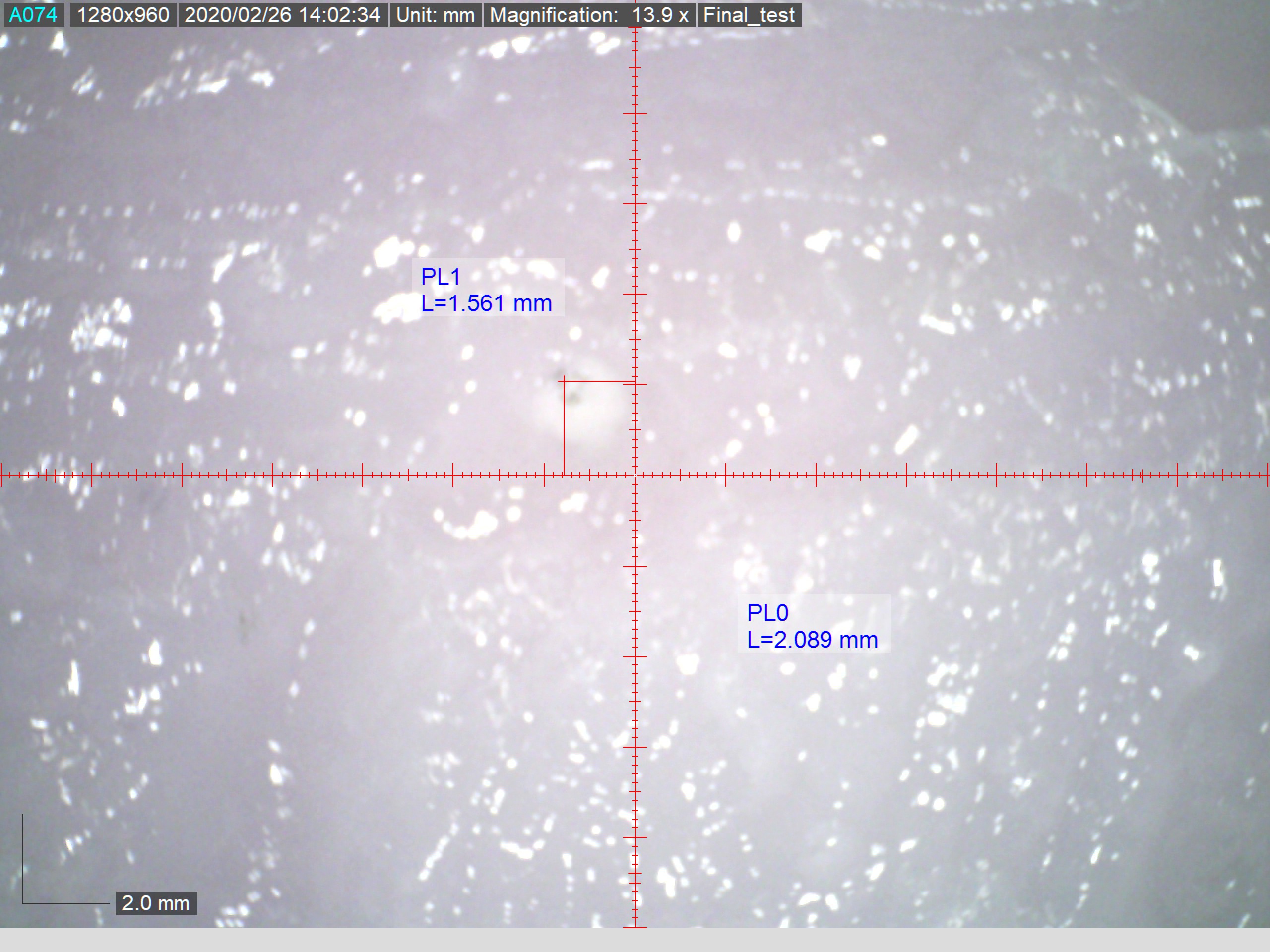

Supplement: S2 File — (ZIP) [file pone.0261089.s002.zip › Soft phantom/photos67.jpg]

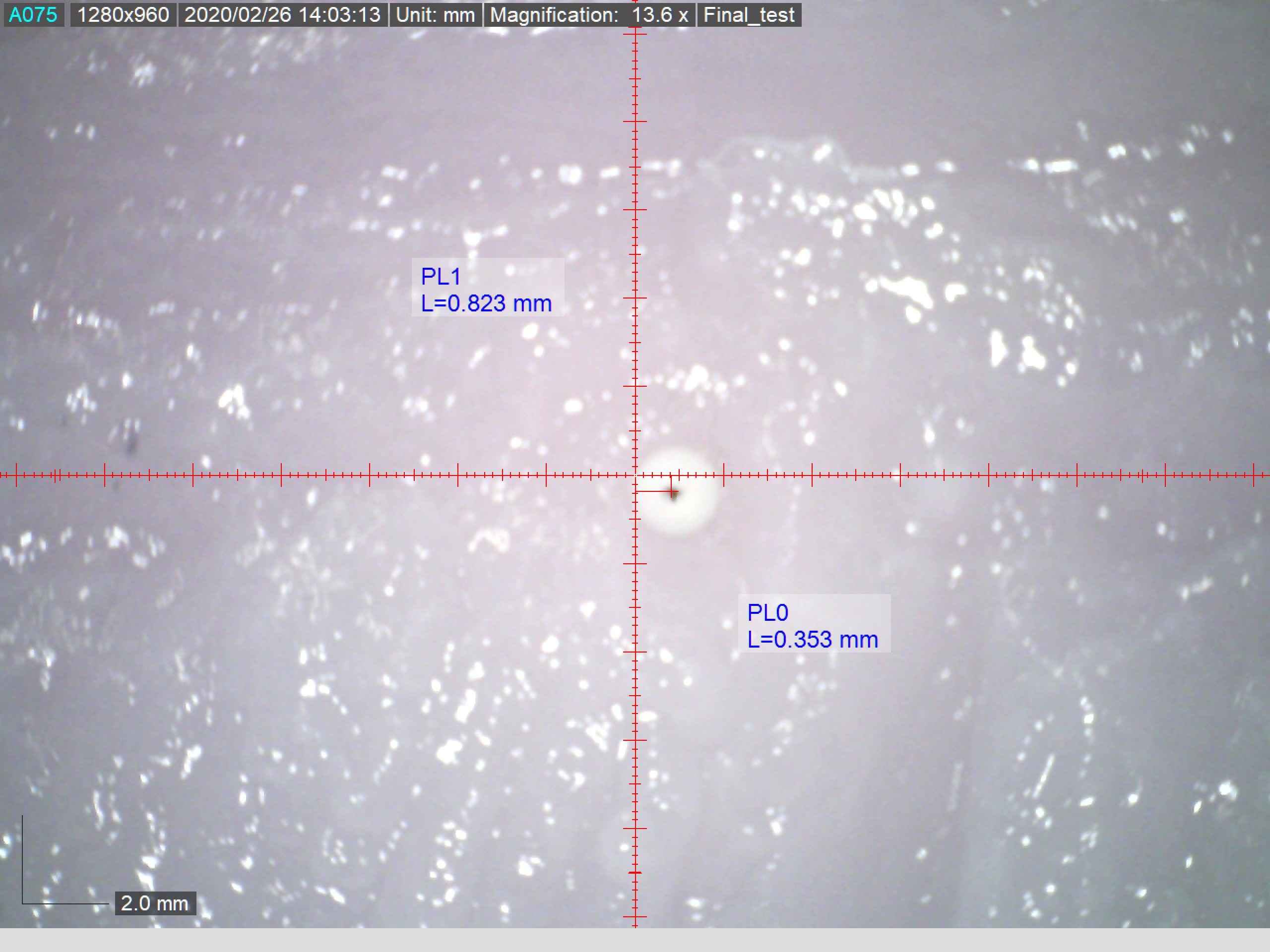

Supplement: S2 File — (ZIP) [file pone.0261089.s002.zip › Soft phantom/photos68.jpg]

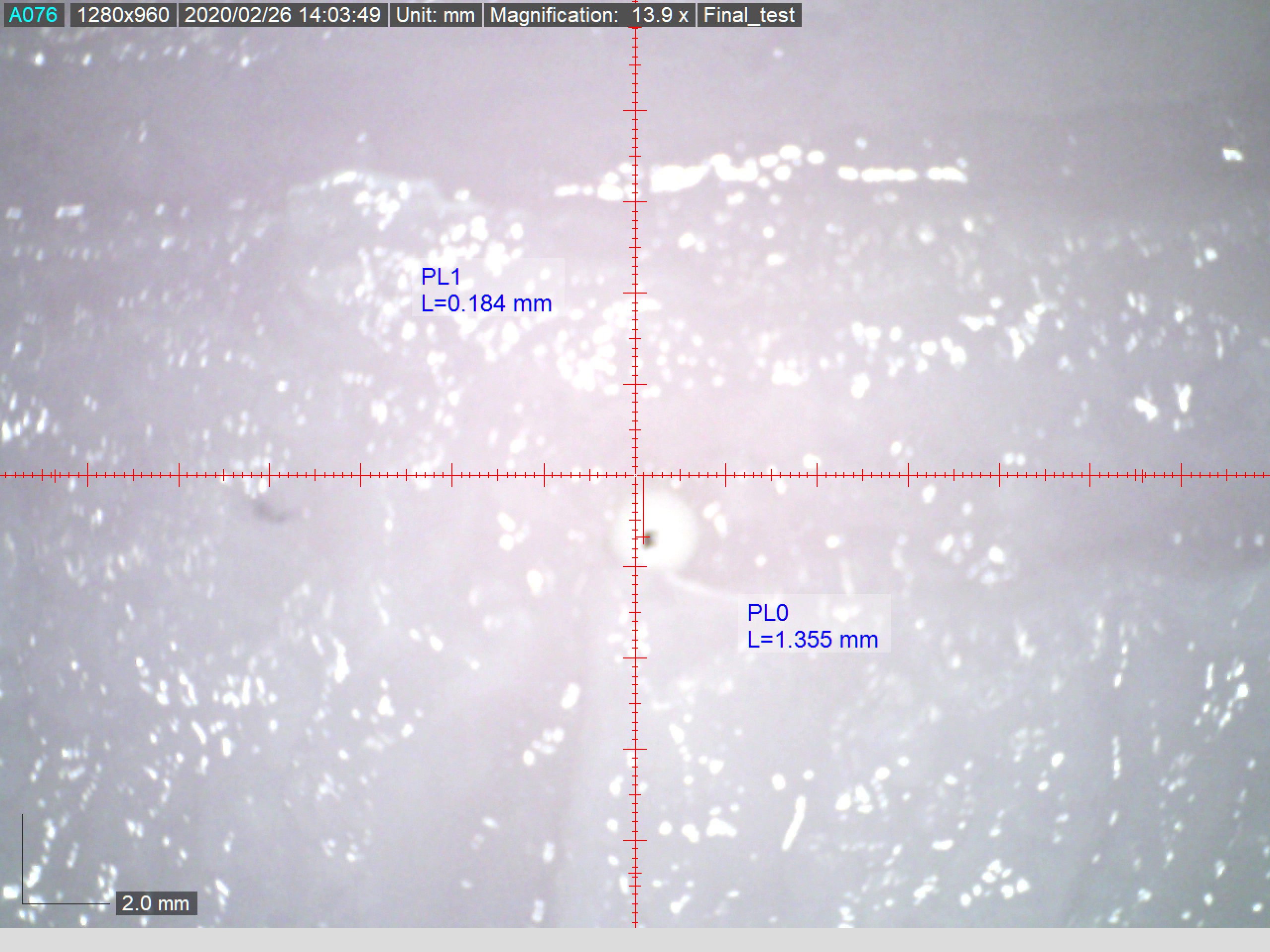

Supplement: S2 File — (ZIP) [file pone.0261089.s002.zip › Soft phantom/photos69.jpg]

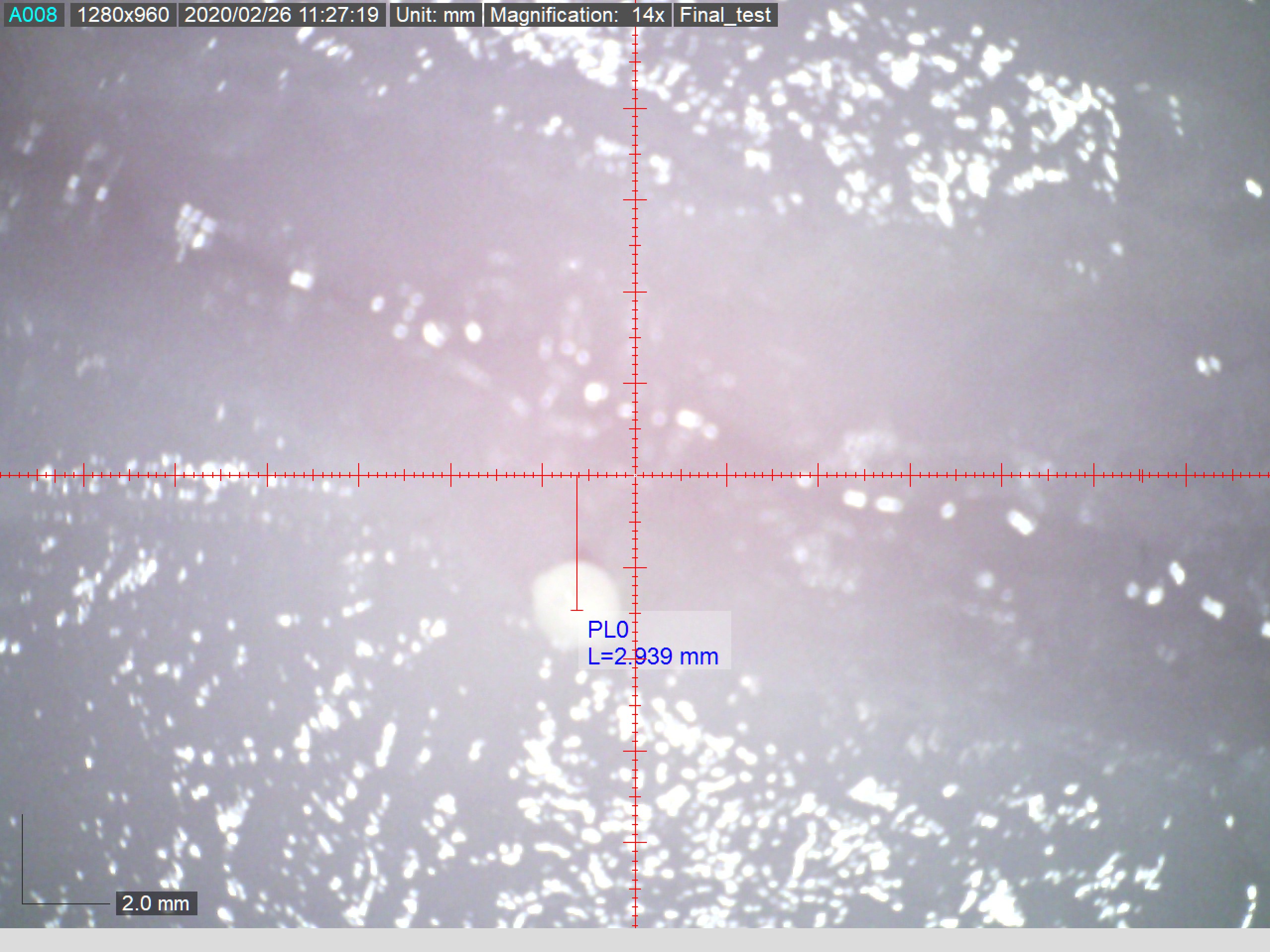

Supplement: S2 File — (ZIP) [file pone.0261089.s002.zip › Soft phantom/photos7.jpg]

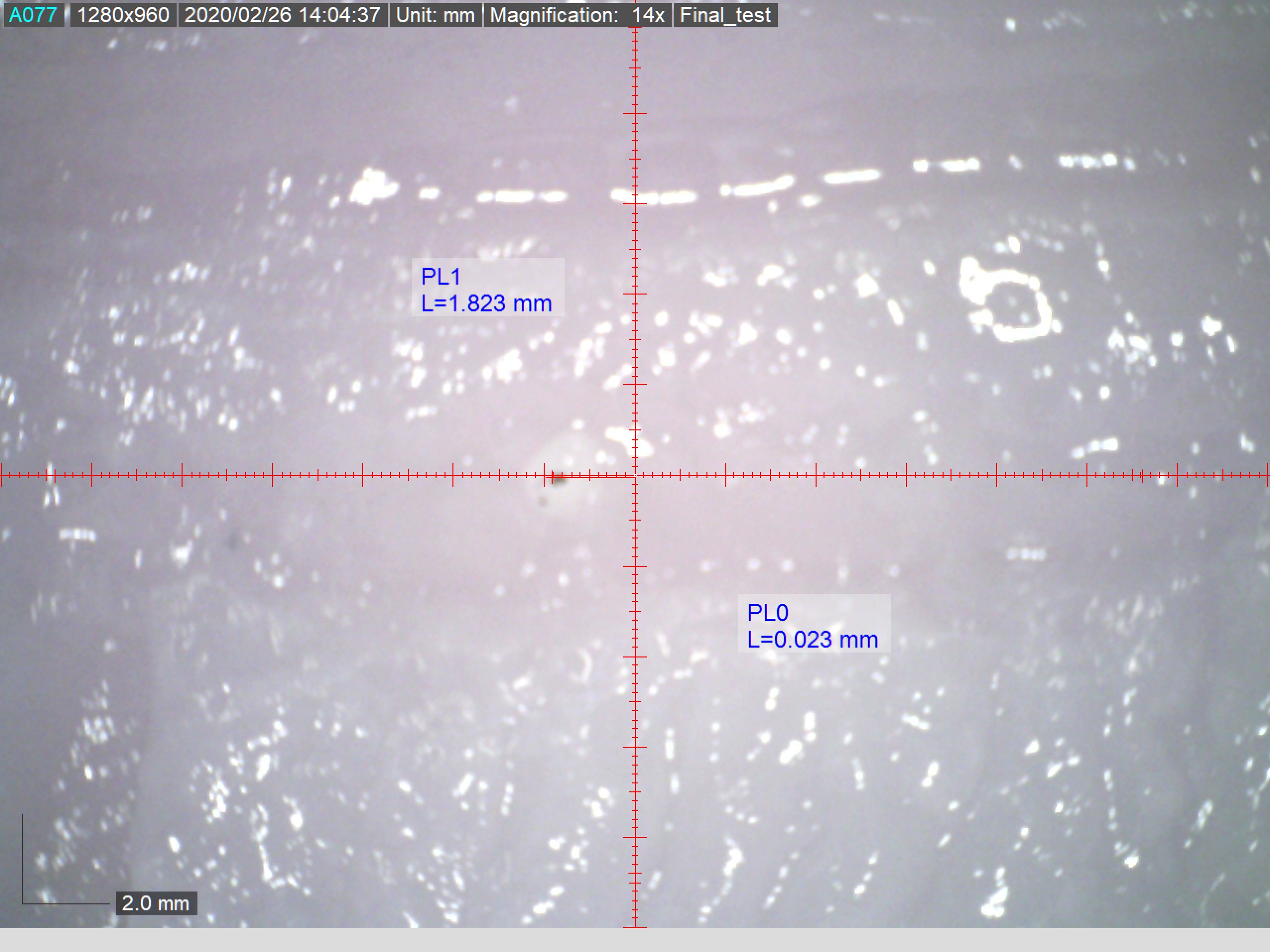

Supplement: S2 File — (ZIP) [file pone.0261089.s002.zip › Soft phantom/photos70.jpg]

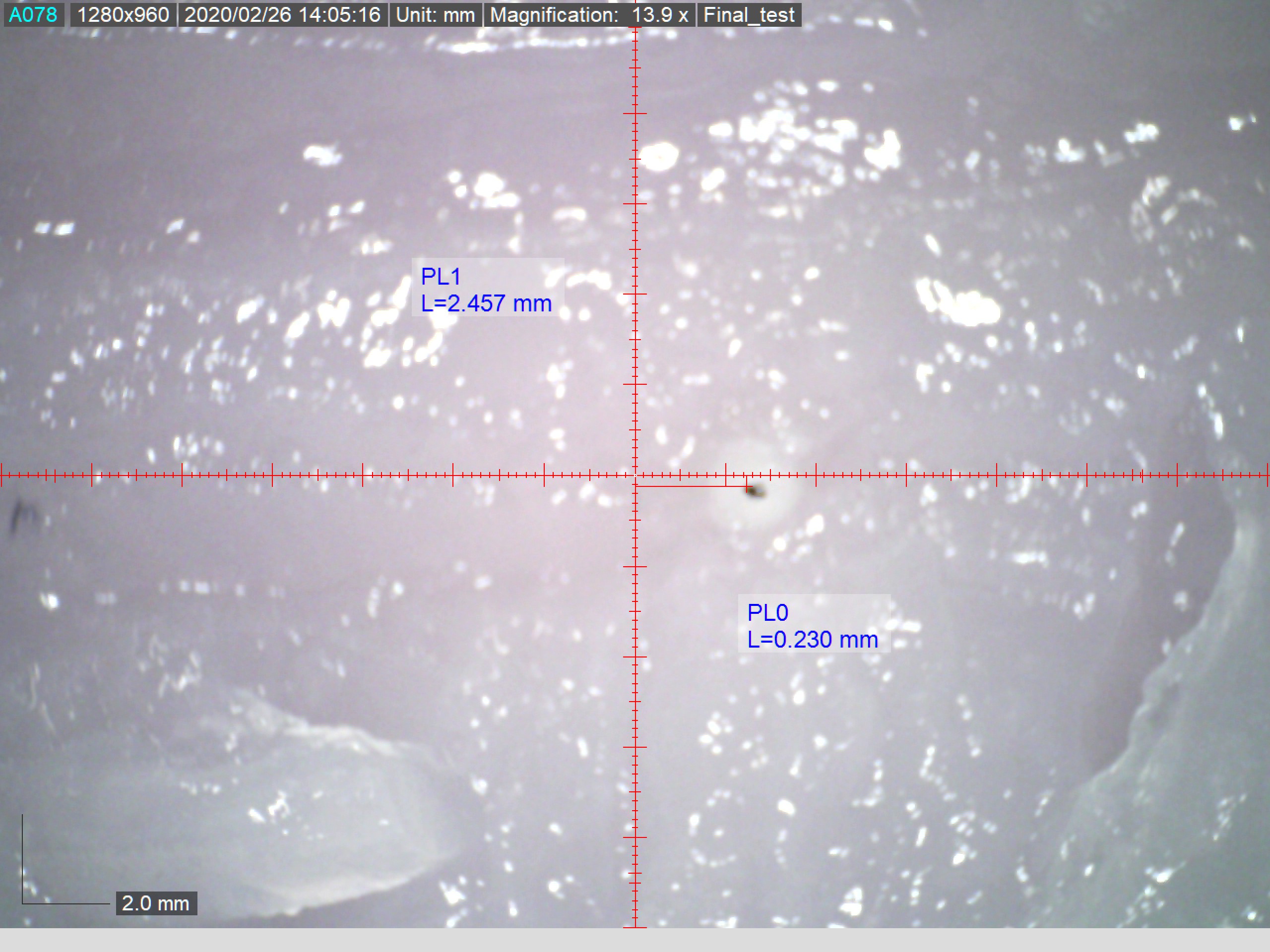

Supplement: S2 File — (ZIP) [file pone.0261089.s002.zip › Soft phantom/photos71.jpg]

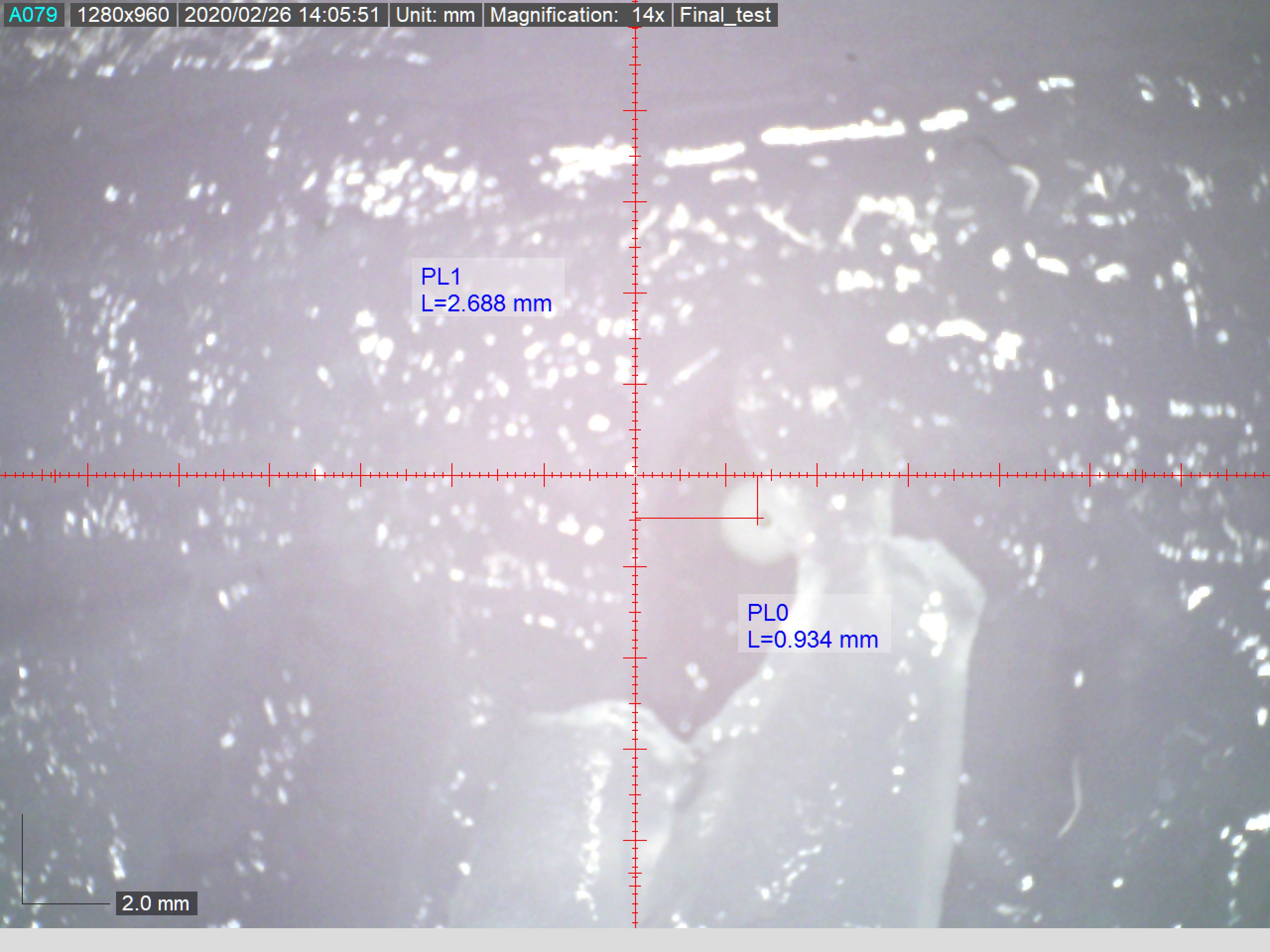

Supplement: S2 File — (ZIP) [file pone.0261089.s002.zip › Soft phantom/photos72.jpg]

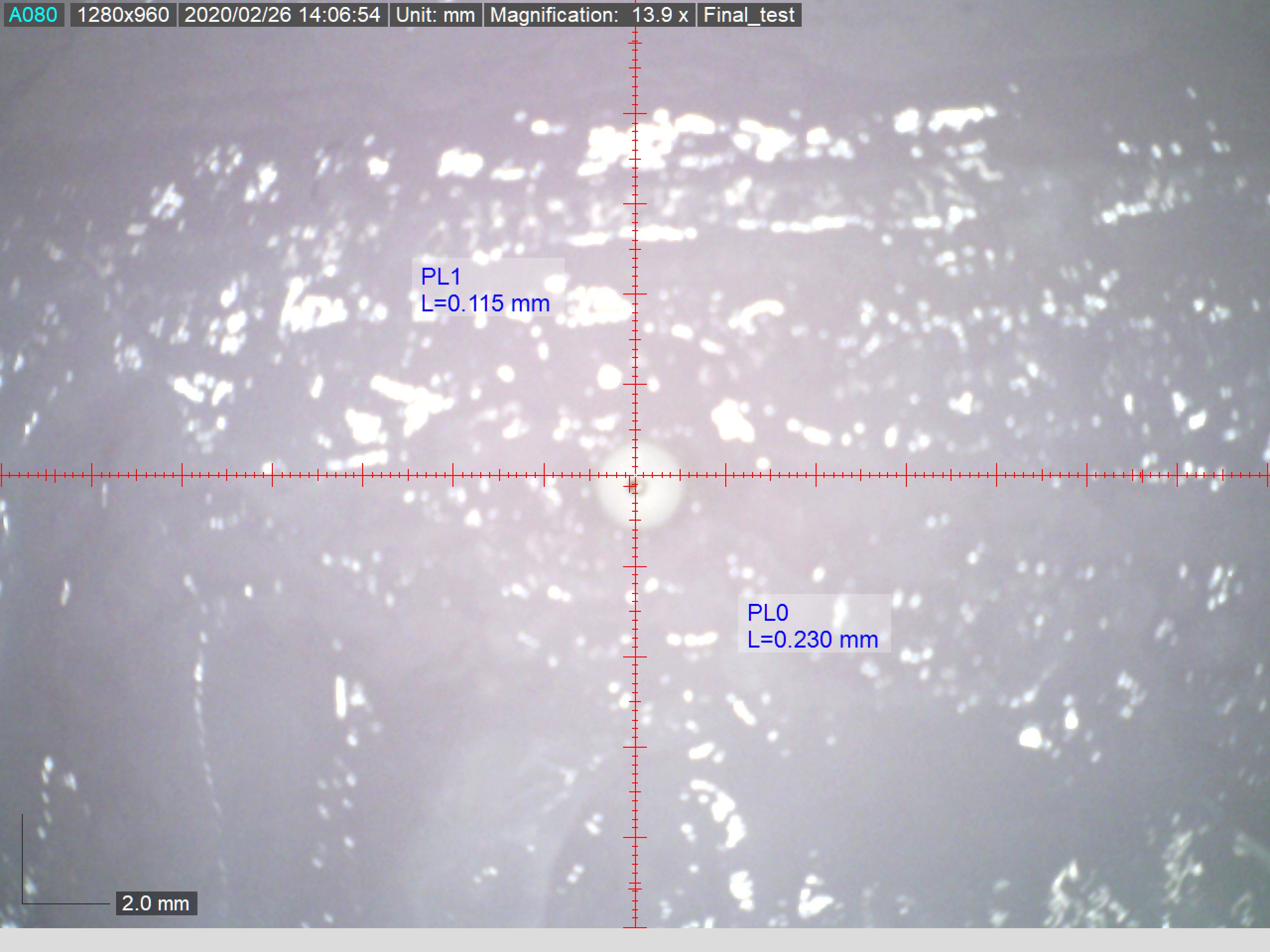

Supplement: S2 File — (ZIP) [file pone.0261089.s002.zip › Soft phantom/photos73.jpg]

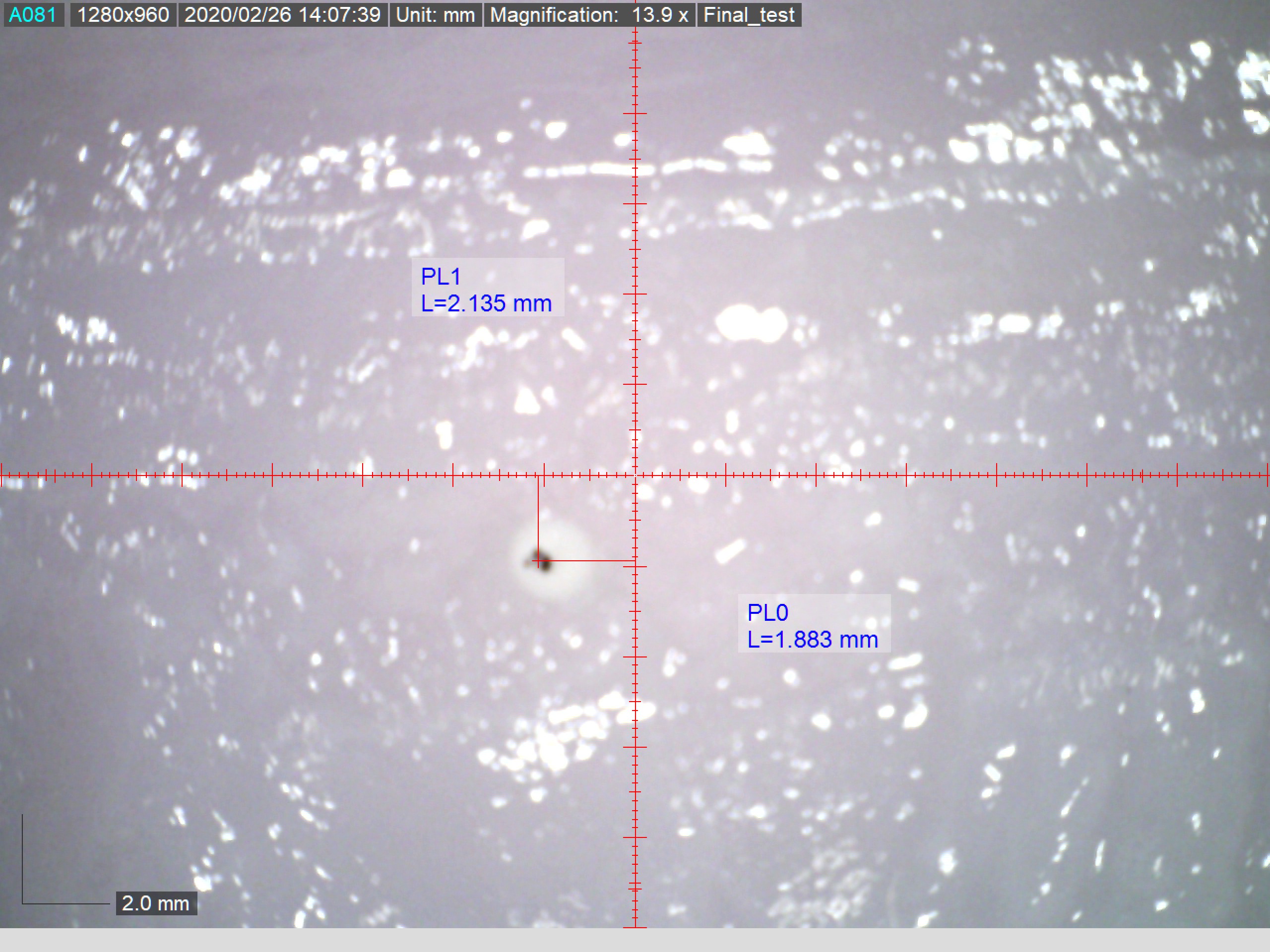

Supplement: S2 File — (ZIP) [file pone.0261089.s002.zip › Soft phantom/photos74.jpg]

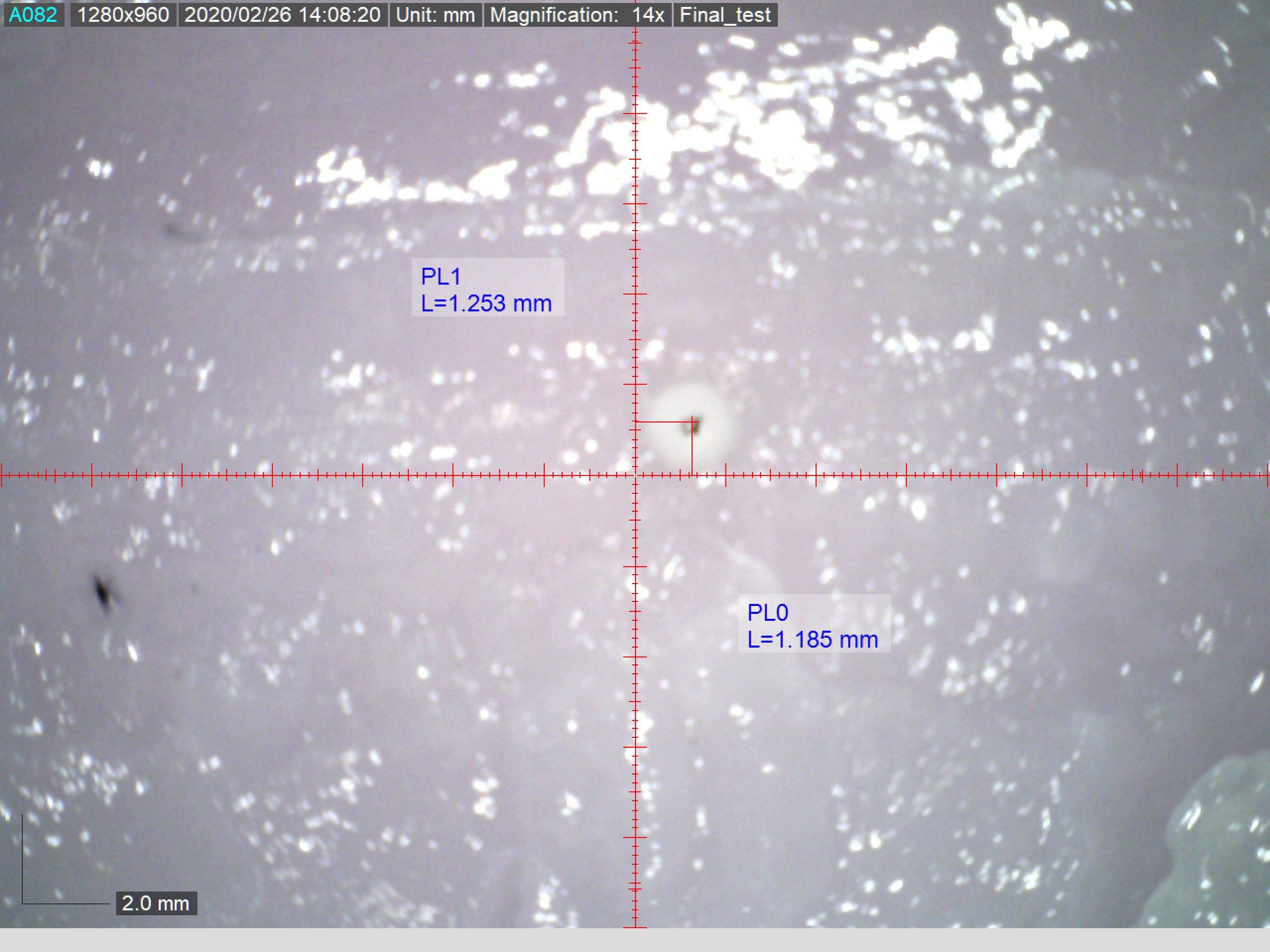

Supplement: S2 File — (ZIP) [file pone.0261089.s002.zip › Soft phantom/photos75.jpg]

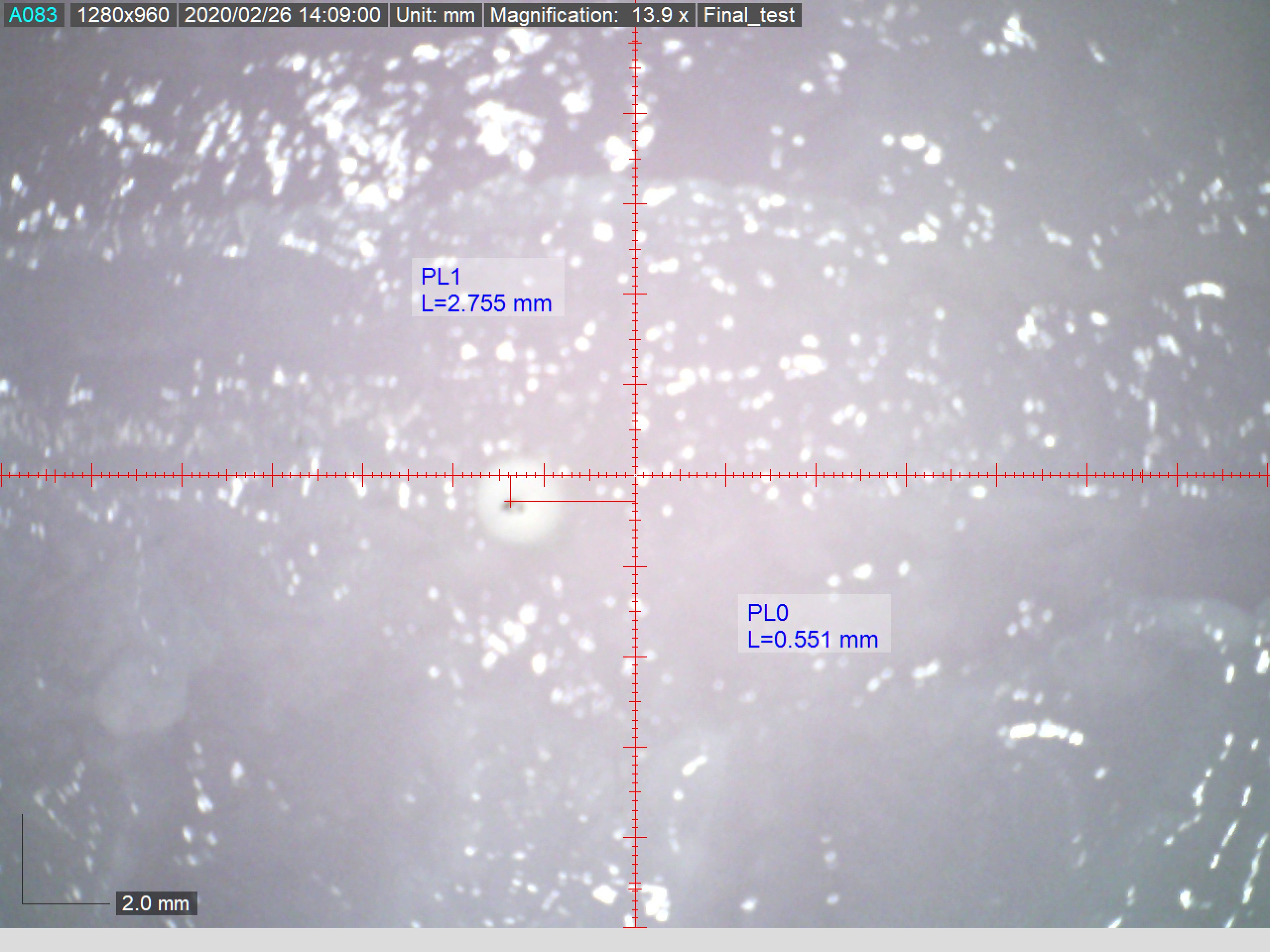

Supplement: S2 File — (ZIP) [file pone.0261089.s002.zip › Soft phantom/photos76.jpg]

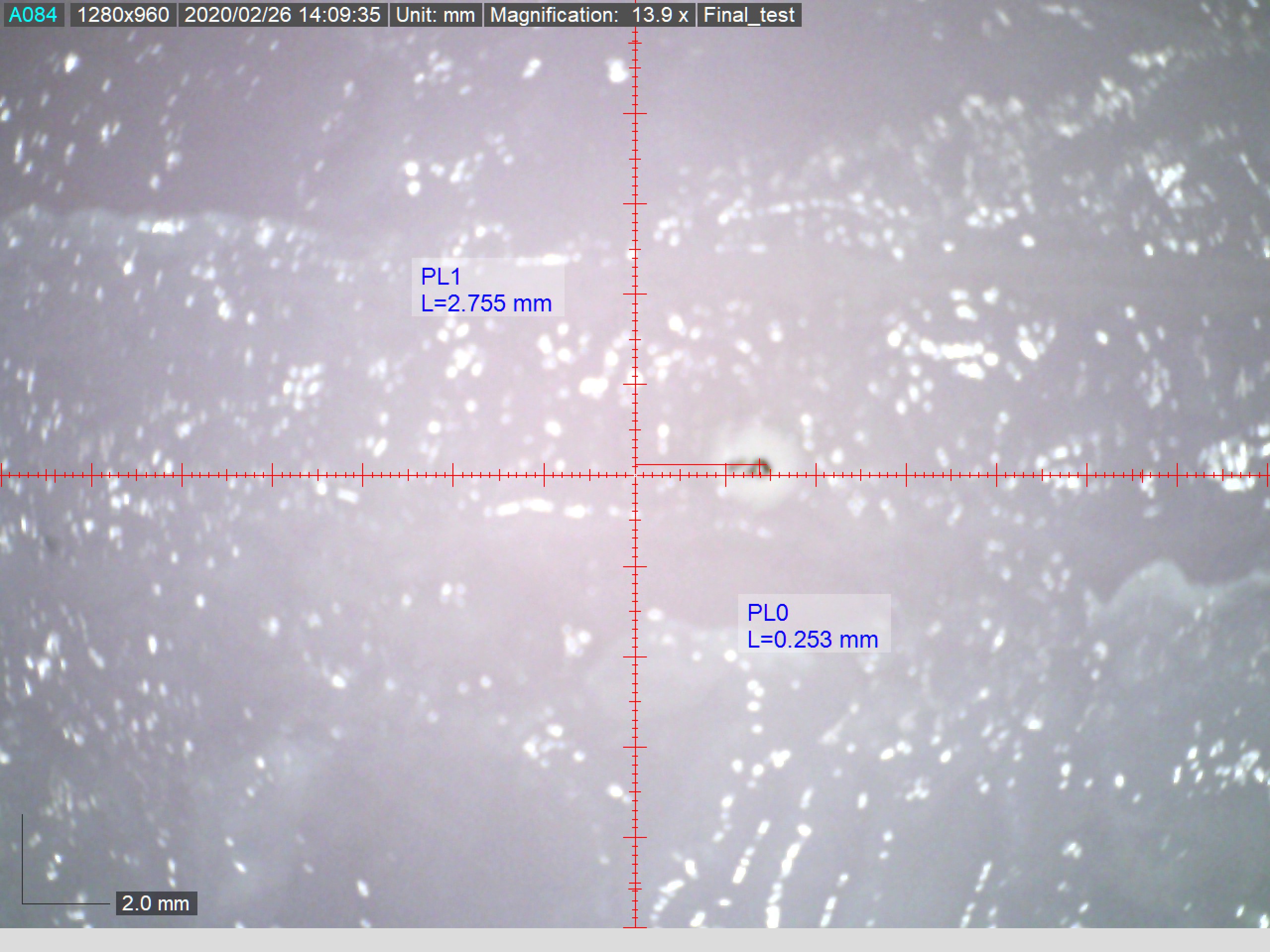

Supplement: S2 File — (ZIP) [file pone.0261089.s002.zip › Soft phantom/photos77.jpg]

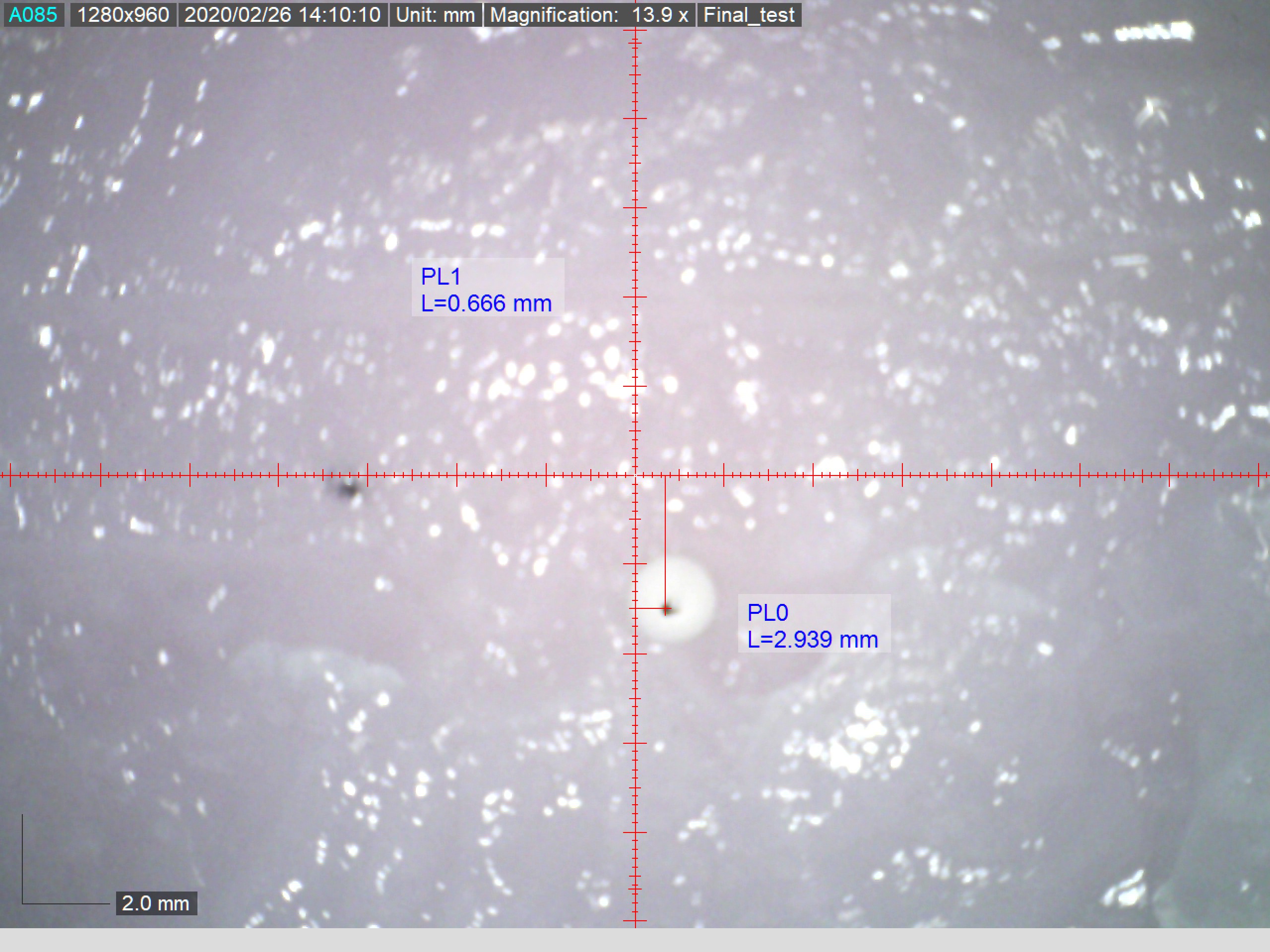

Supplement: S2 File — (ZIP) [file pone.0261089.s002.zip › Soft phantom/photos78.jpg]

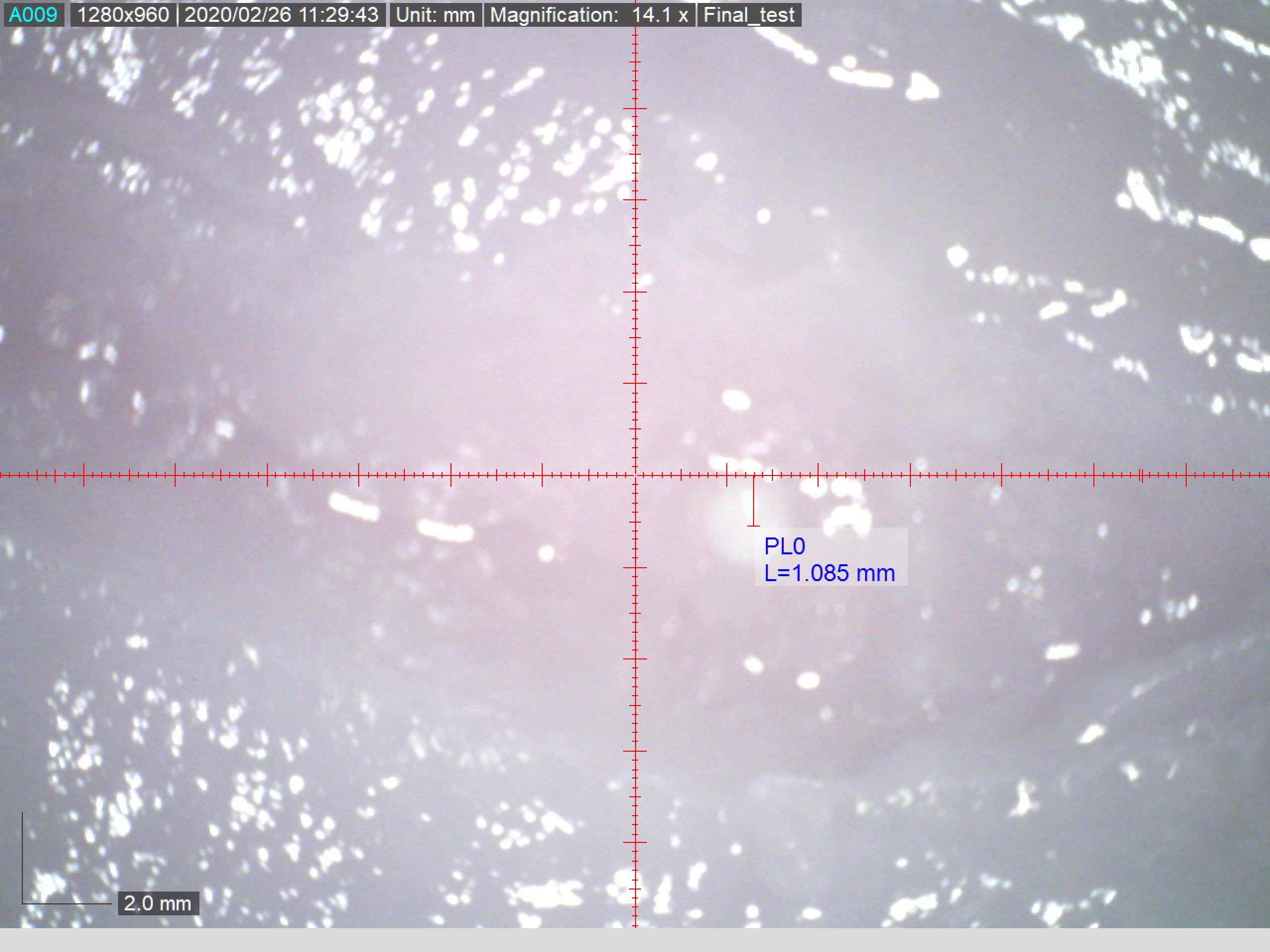

Supplement: S2 File — (ZIP) [file pone.0261089.s002.zip › Soft phantom/photos8.jpg]

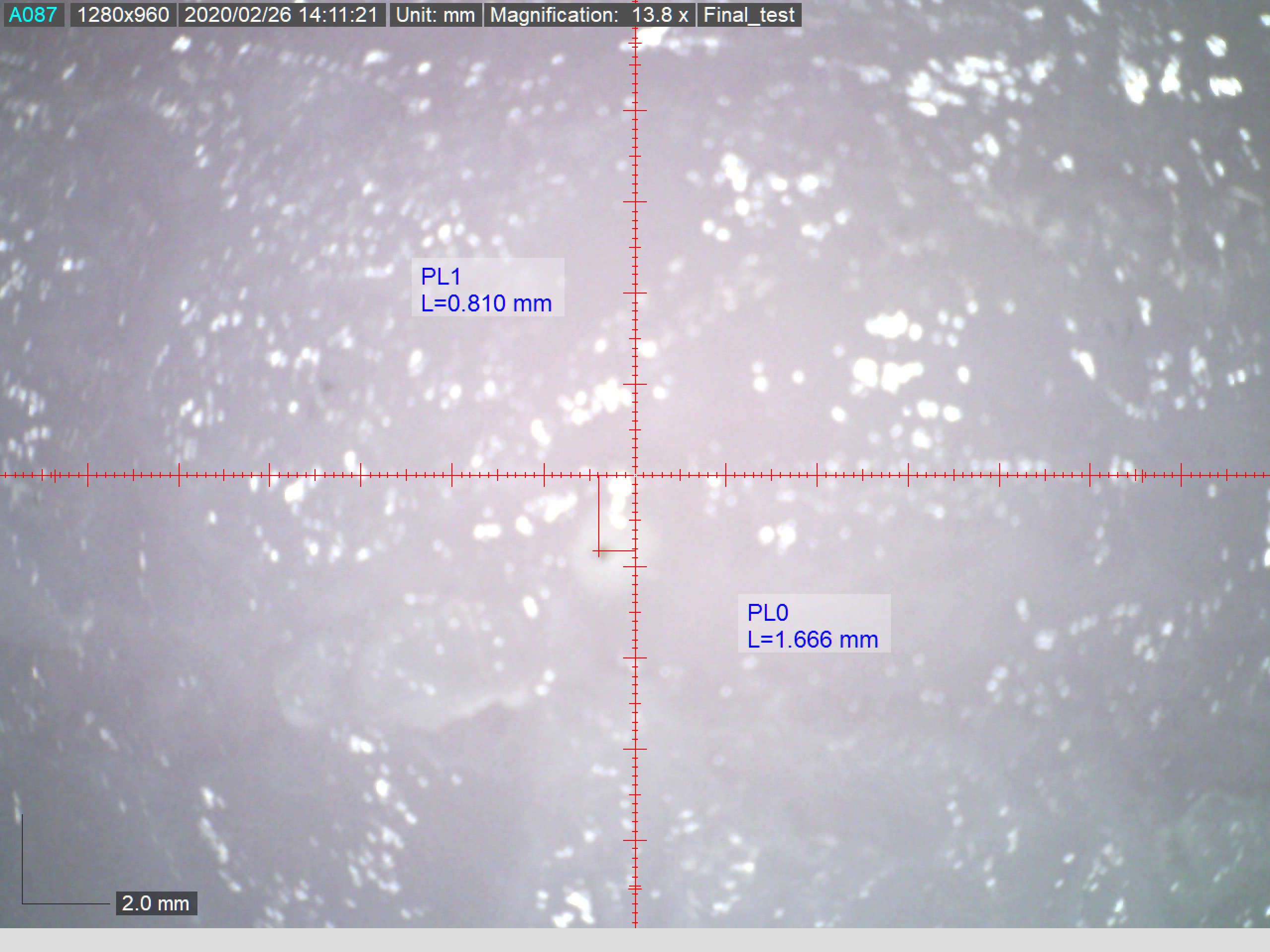

Supplement: S2 File — (ZIP) [file pone.0261089.s002.zip › Soft phantom/photos80.jpg]

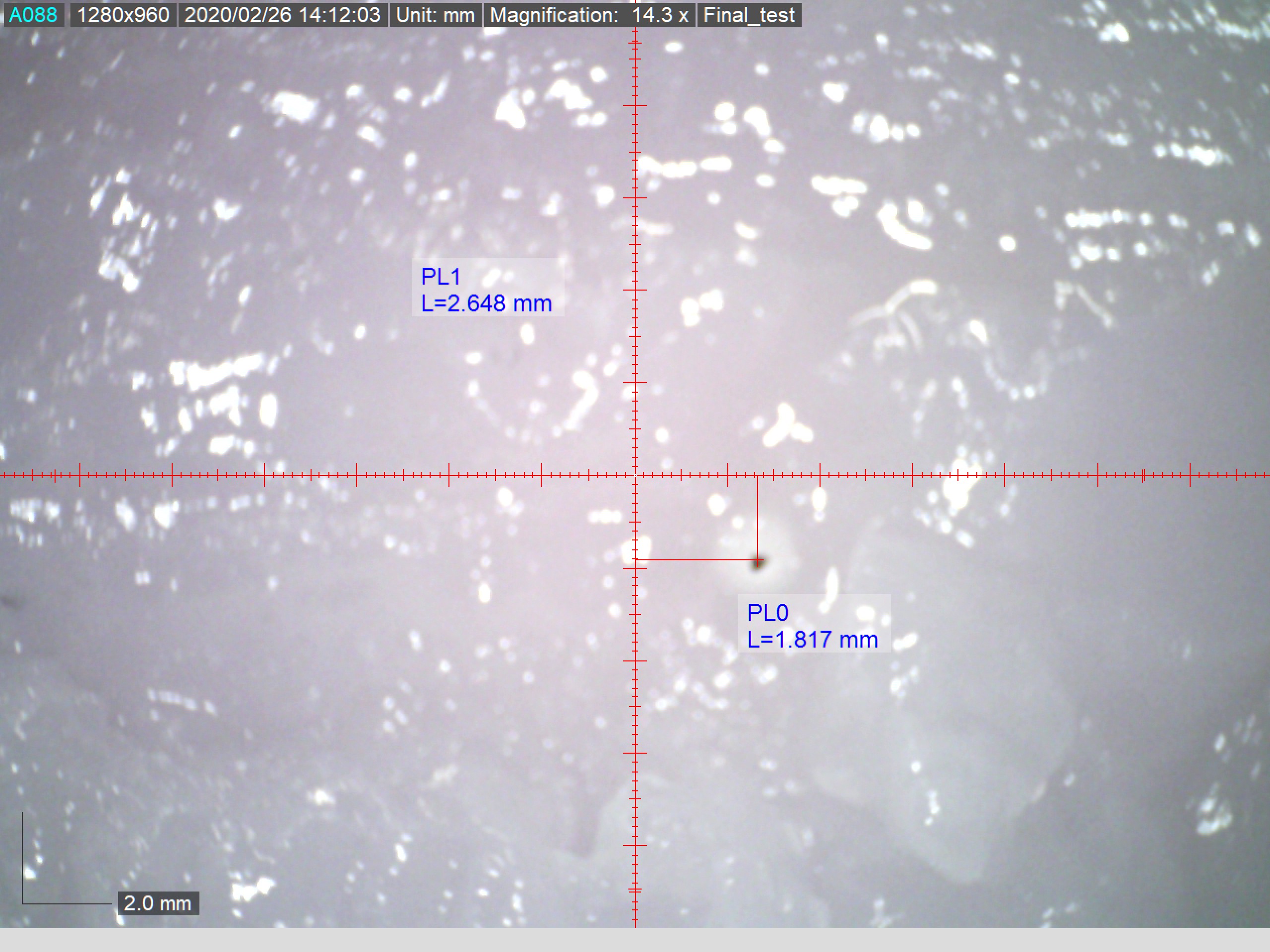

Supplement: S2 File — (ZIP) [file pone.0261089.s002.zip › Soft phantom/photos81.jpg]

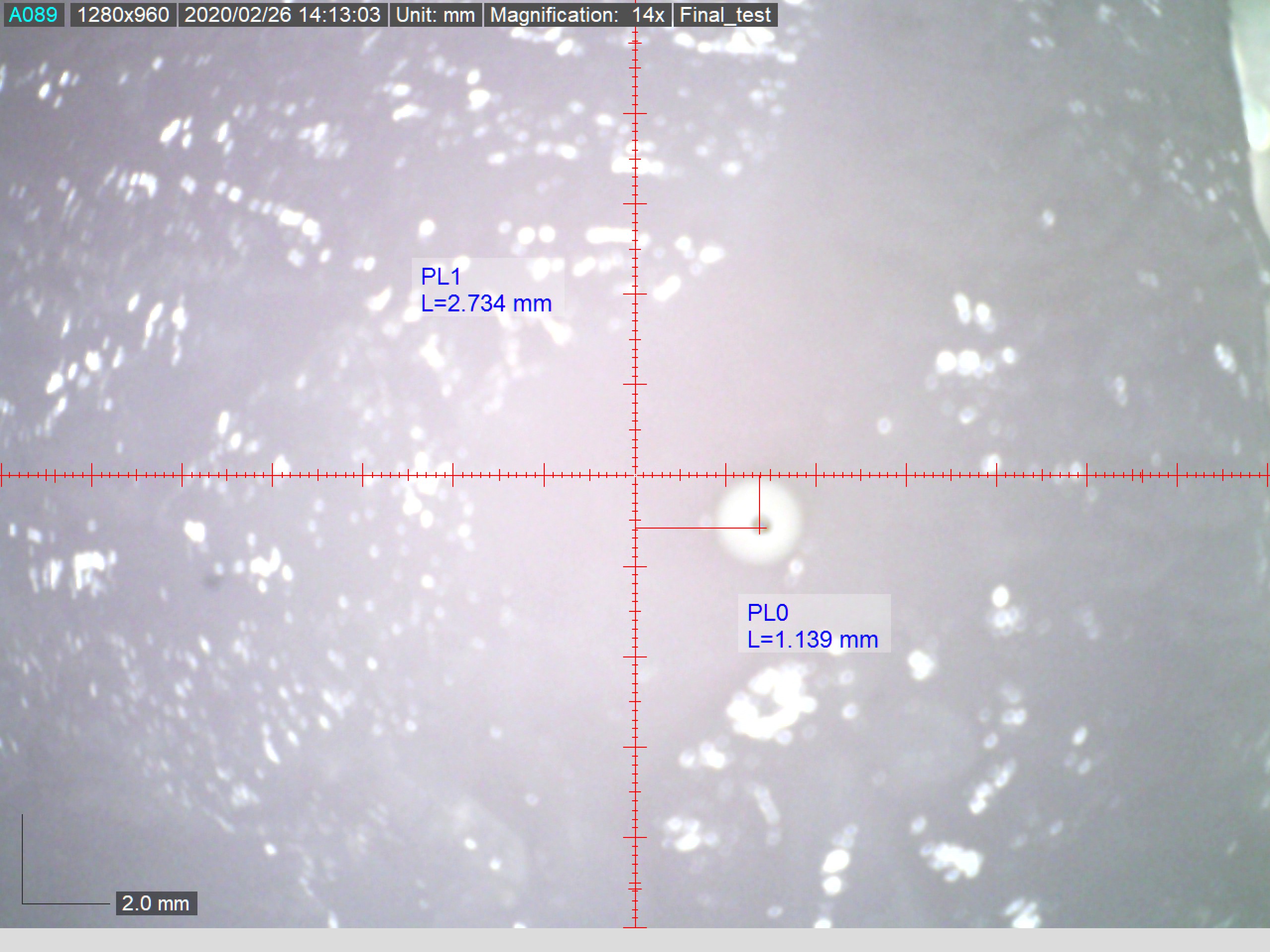

Supplement: S2 File — (ZIP) [file pone.0261089.s002.zip › Soft phantom/photos82.jpg]

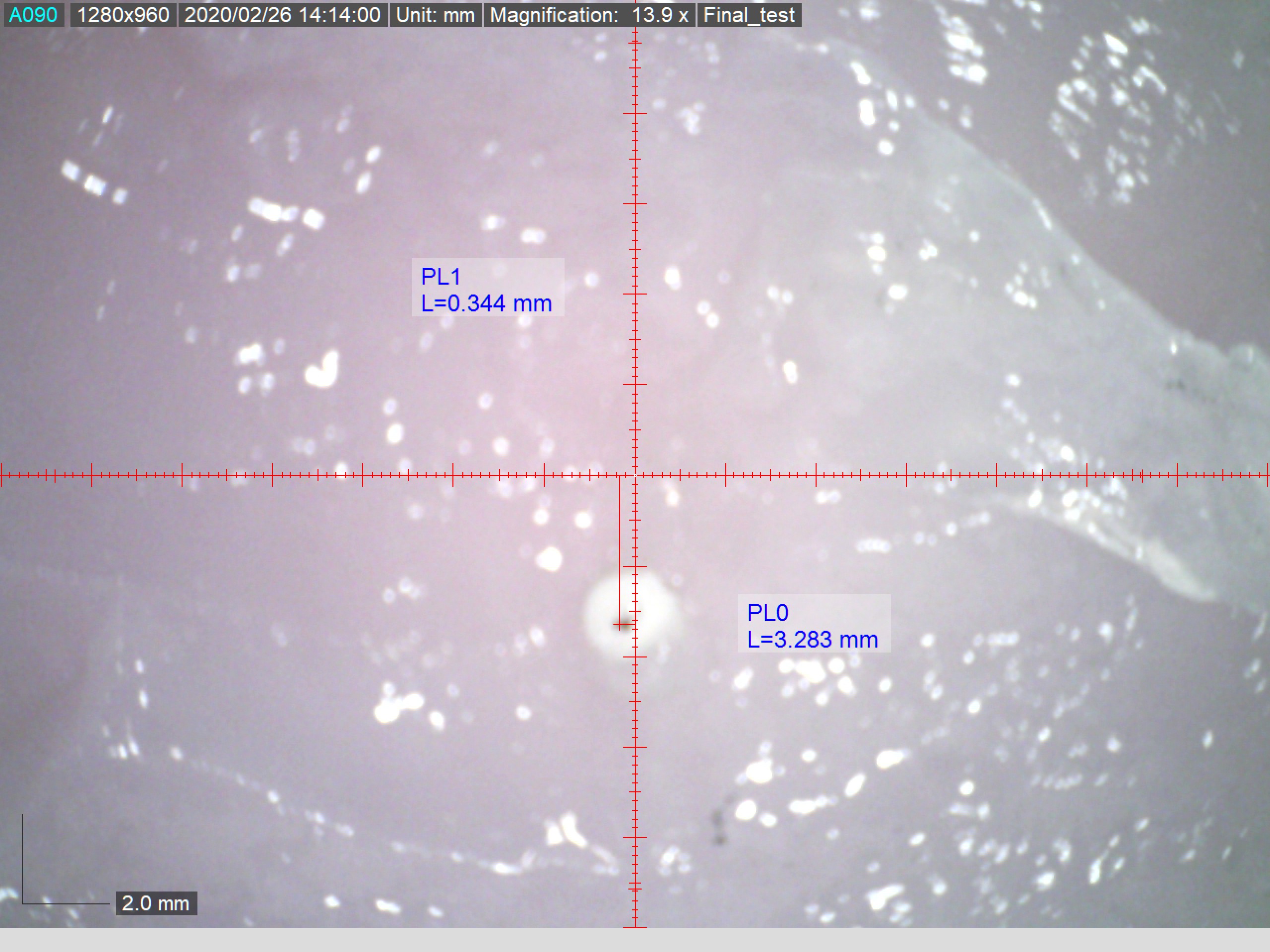

Supplement: S2 File — (ZIP) [file pone.0261089.s002.zip › Soft phantom/photos83.jpg]

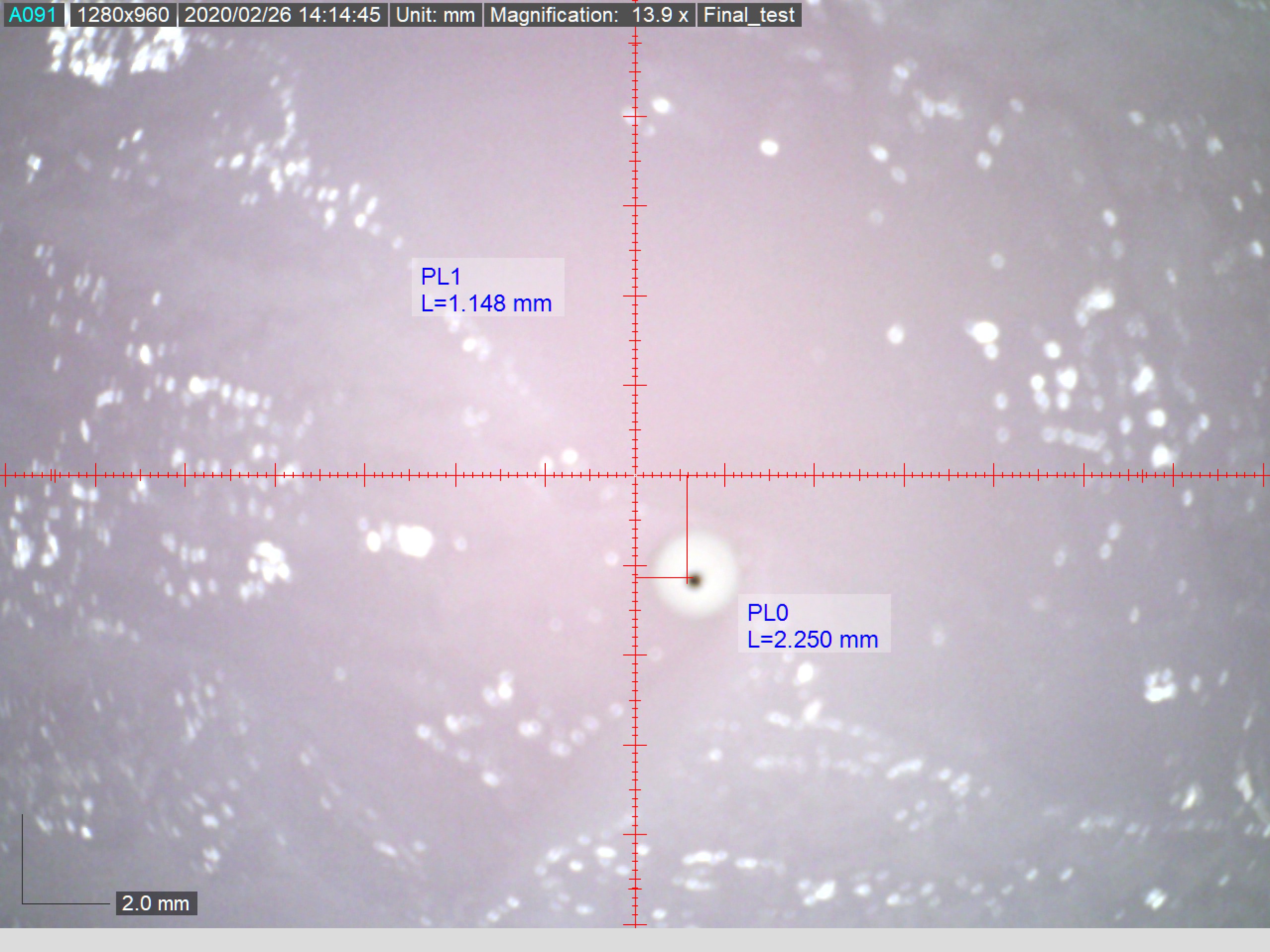

Supplement: S2 File — (ZIP) [file pone.0261089.s002.zip › Soft phantom/photos84.jpg]

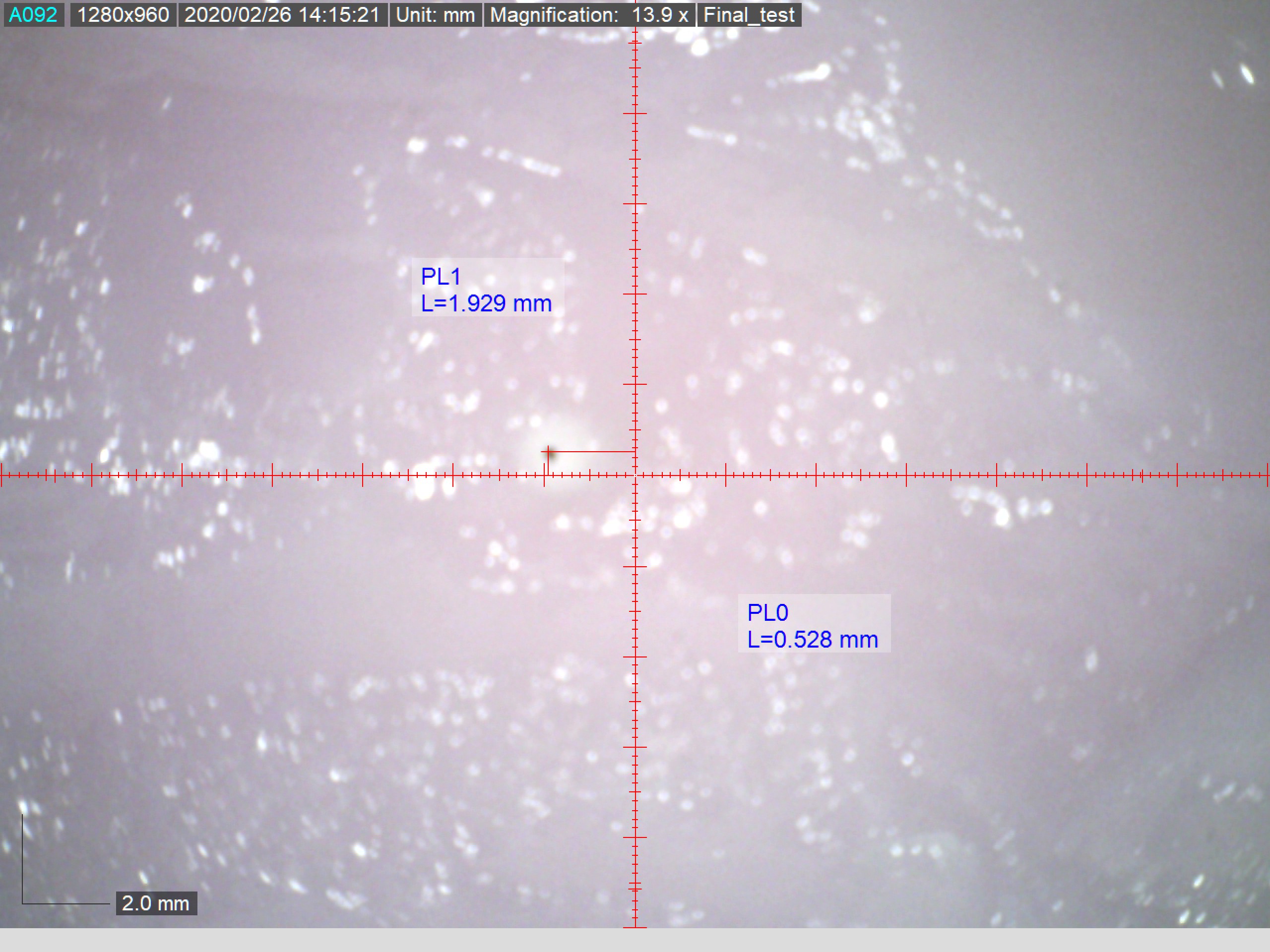

Supplement: S2 File — (ZIP) [file pone.0261089.s002.zip › Soft phantom/photos85.jpg]

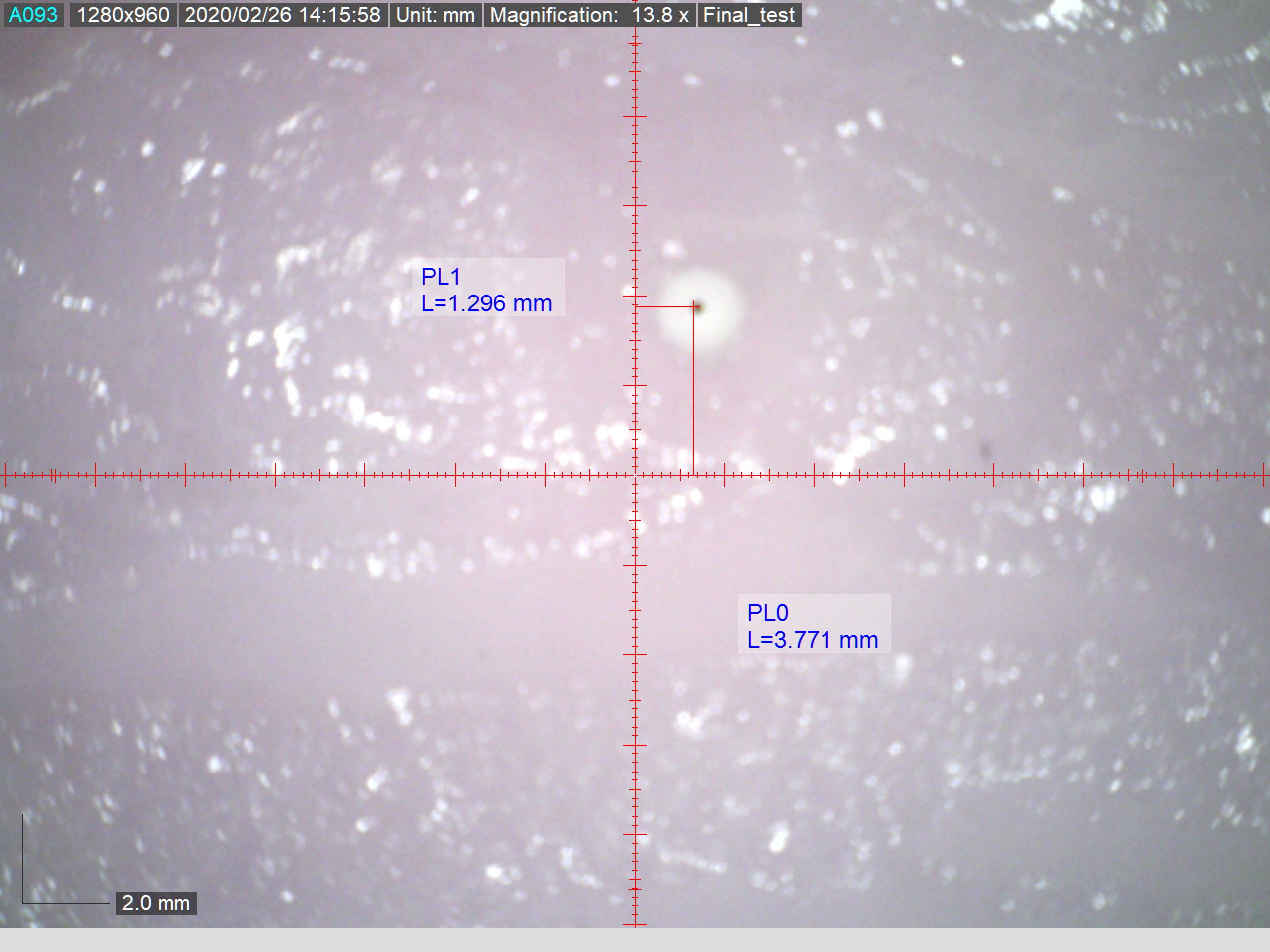

Supplement: S2 File — (ZIP) [file pone.0261089.s002.zip › Soft phantom/photos86.jpg]

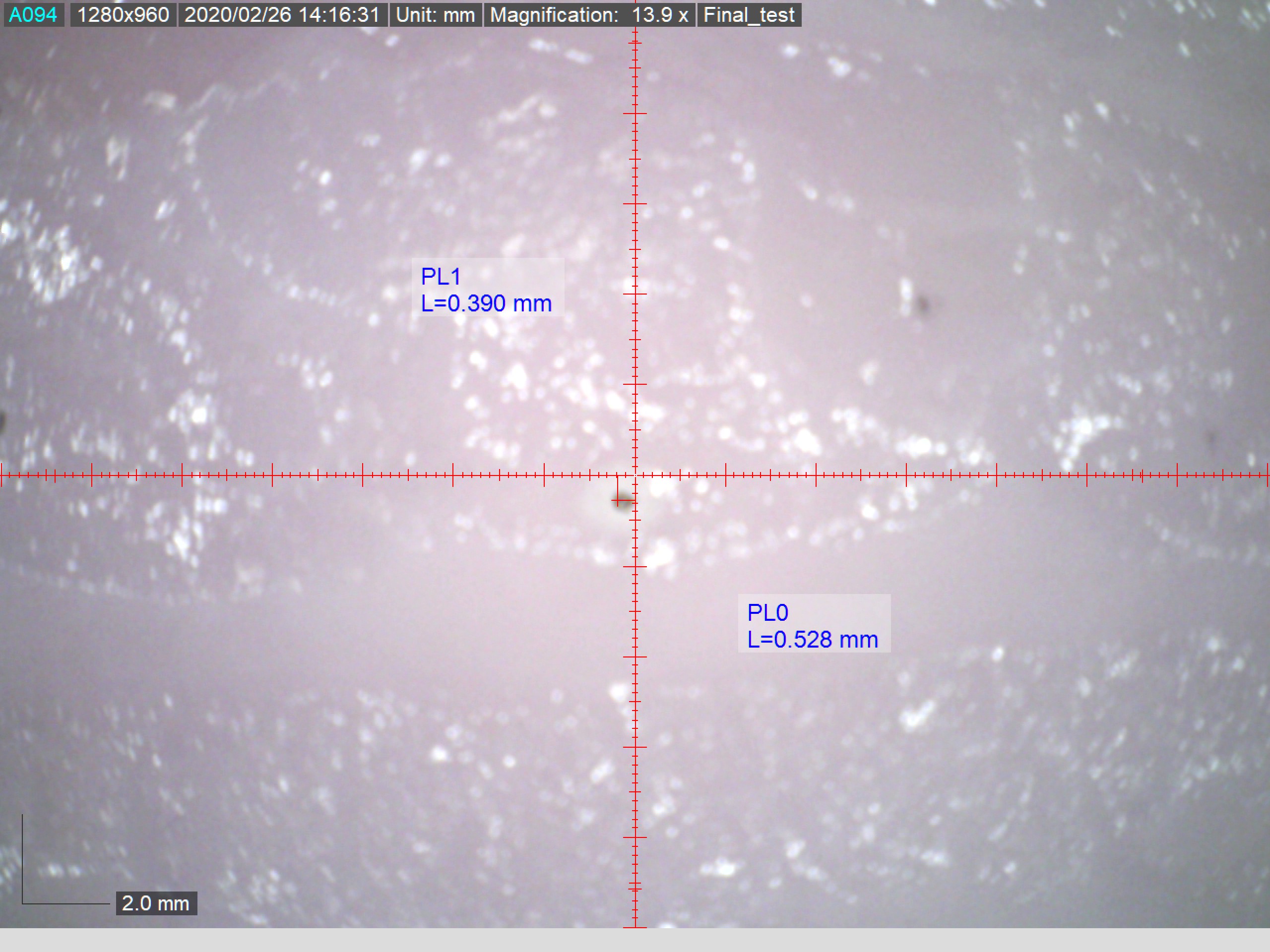

Supplement: S2 File — (ZIP) [file pone.0261089.s002.zip › Soft phantom/photos87.jpg]

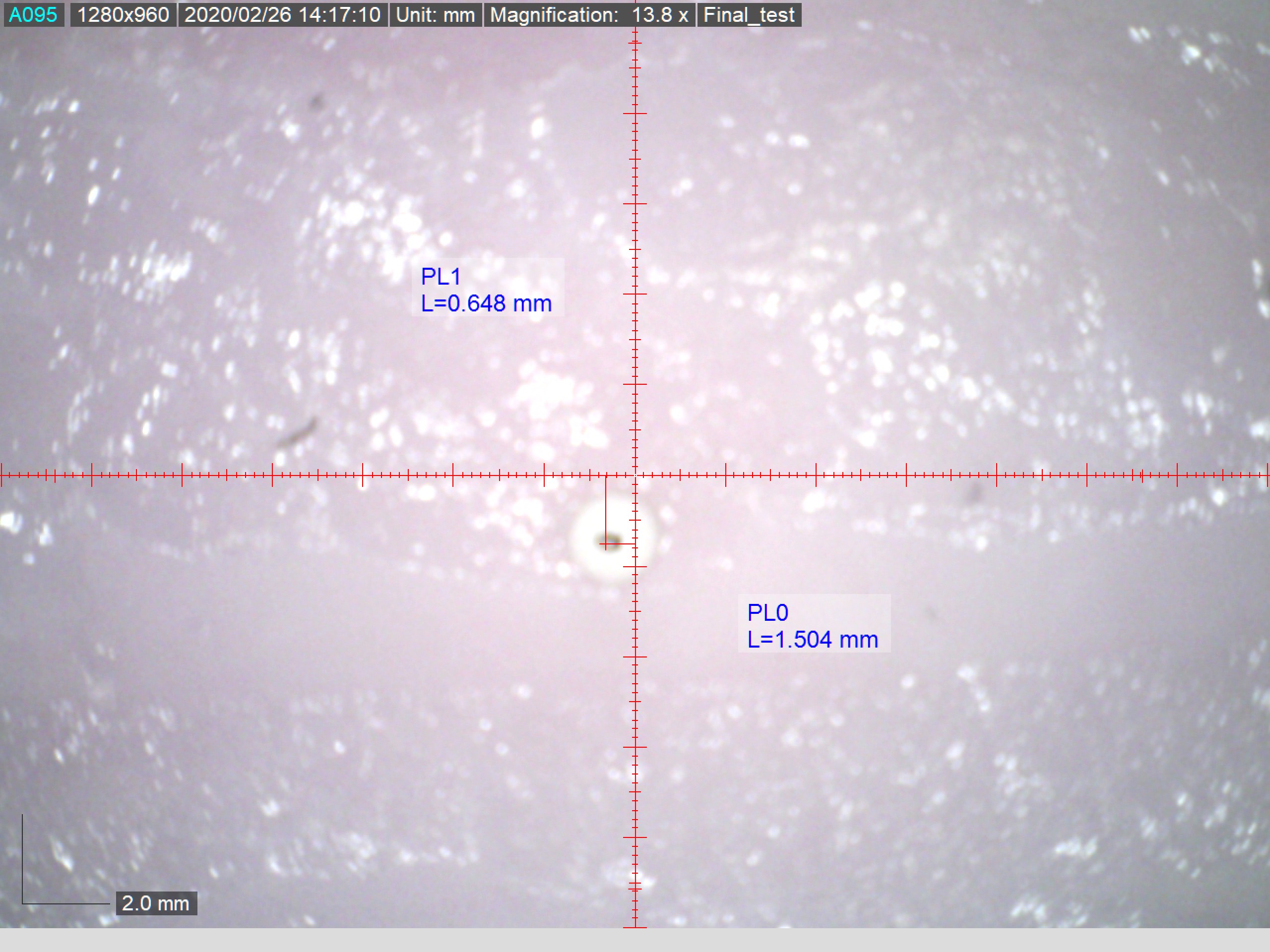

Supplement: S2 File — (ZIP) [file pone.0261089.s002.zip › Soft phantom/photos88.jpg]

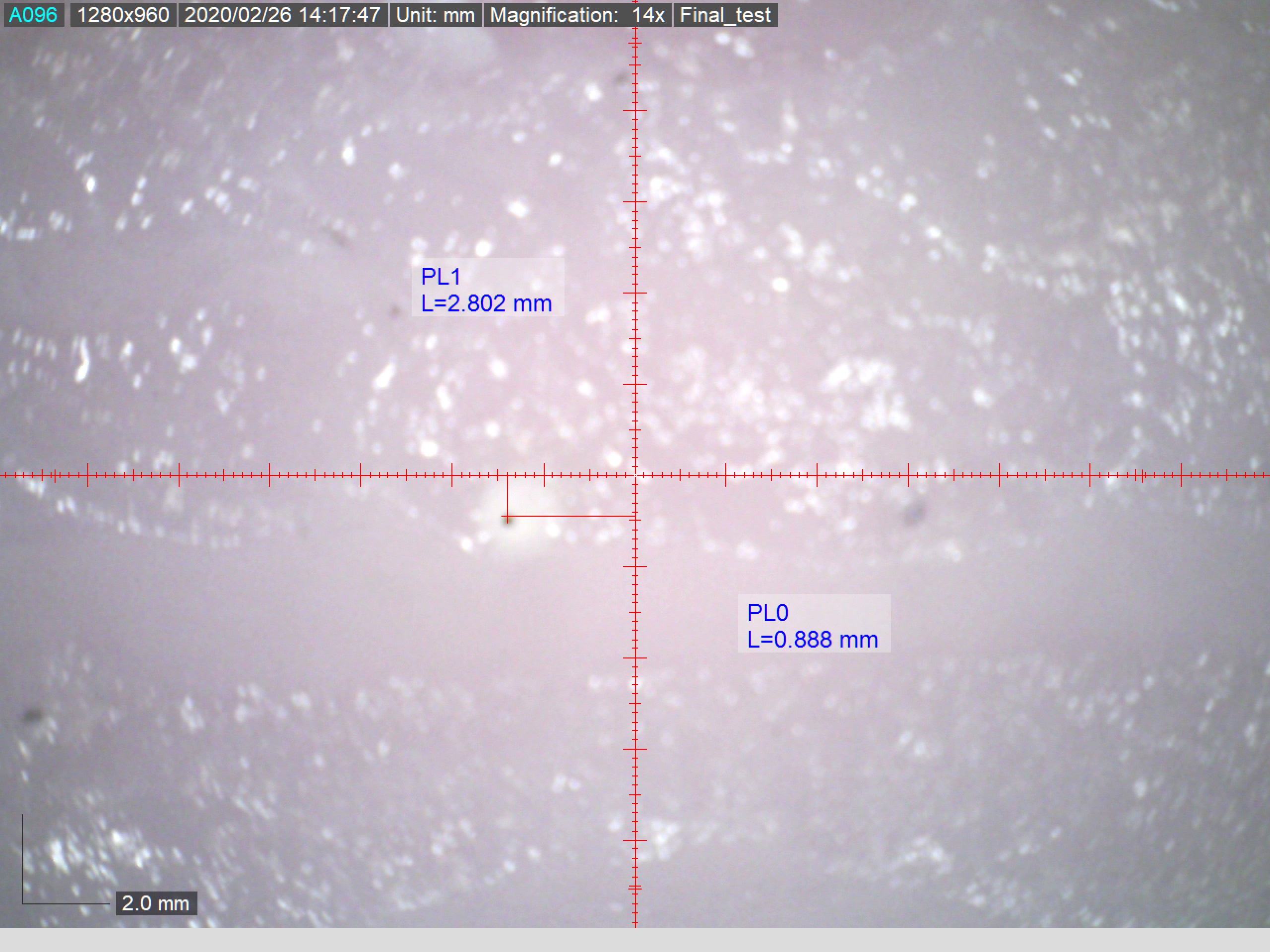

Supplement: S2 File — (ZIP) [file pone.0261089.s002.zip › Soft phantom/photos89.jpg]

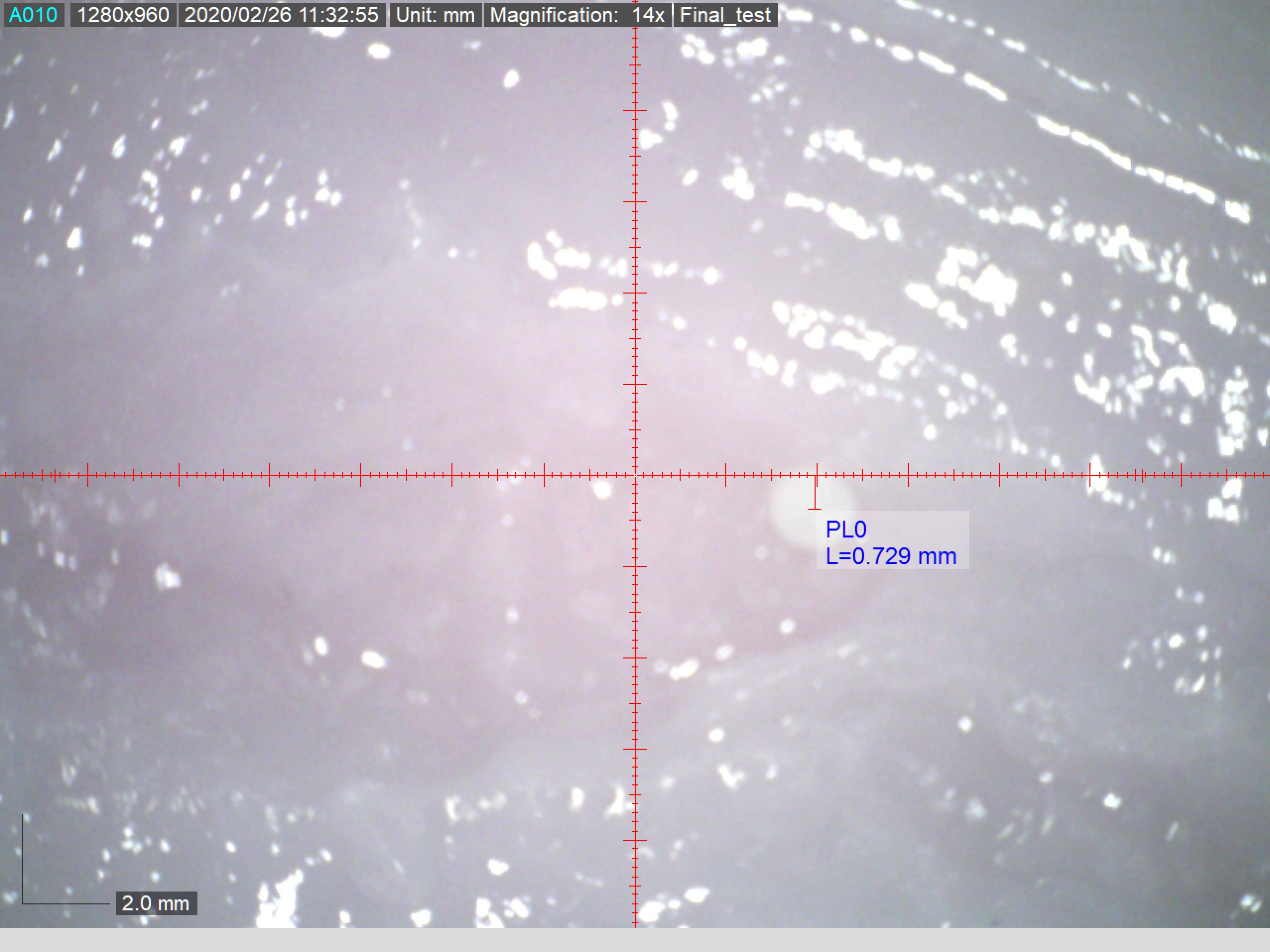

Supplement: S2 File — (ZIP) [file pone.0261089.s002.zip › Soft phantom/photos9.jpg]

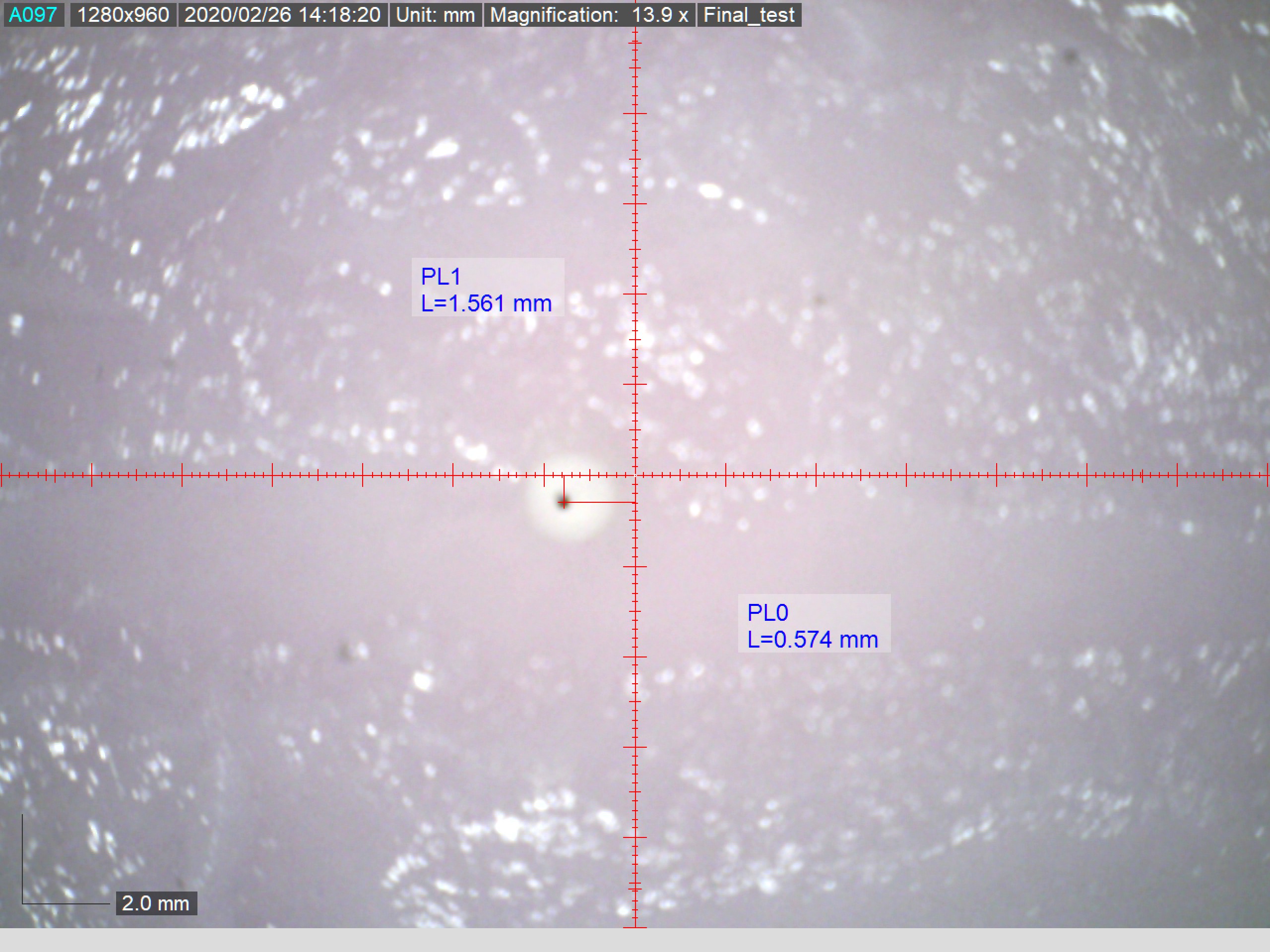

Supplement: S2 File — (ZIP) [file pone.0261089.s002.zip › Soft phantom/photos90.jpg]

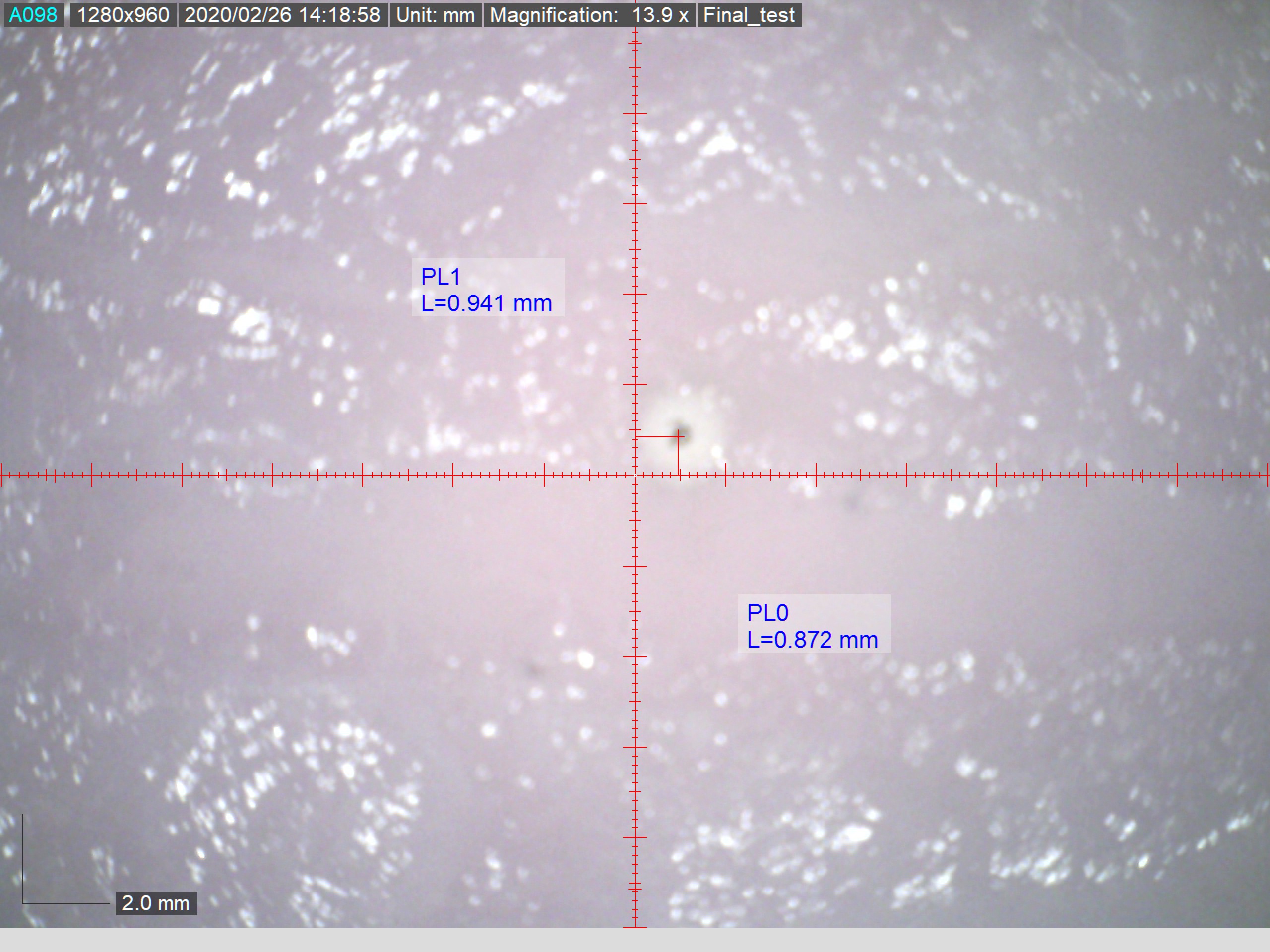

Supplement: S2 File — (ZIP) [file pone.0261089.s002.zip › Soft phantom/photos91.jpg]

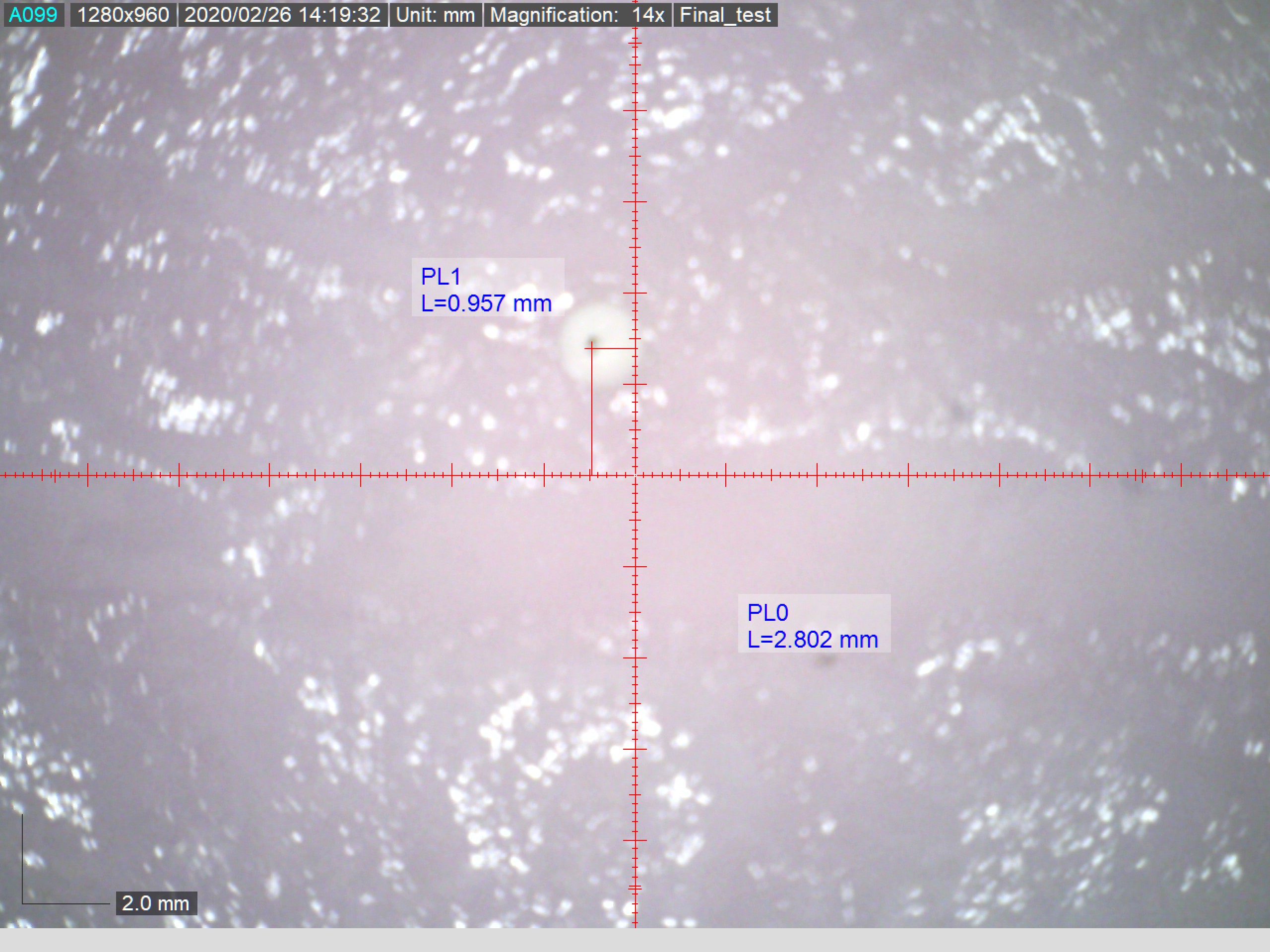

Supplement: S2 File — (ZIP) [file pone.0261089.s002.zip › Soft phantom/photos92.jpg]

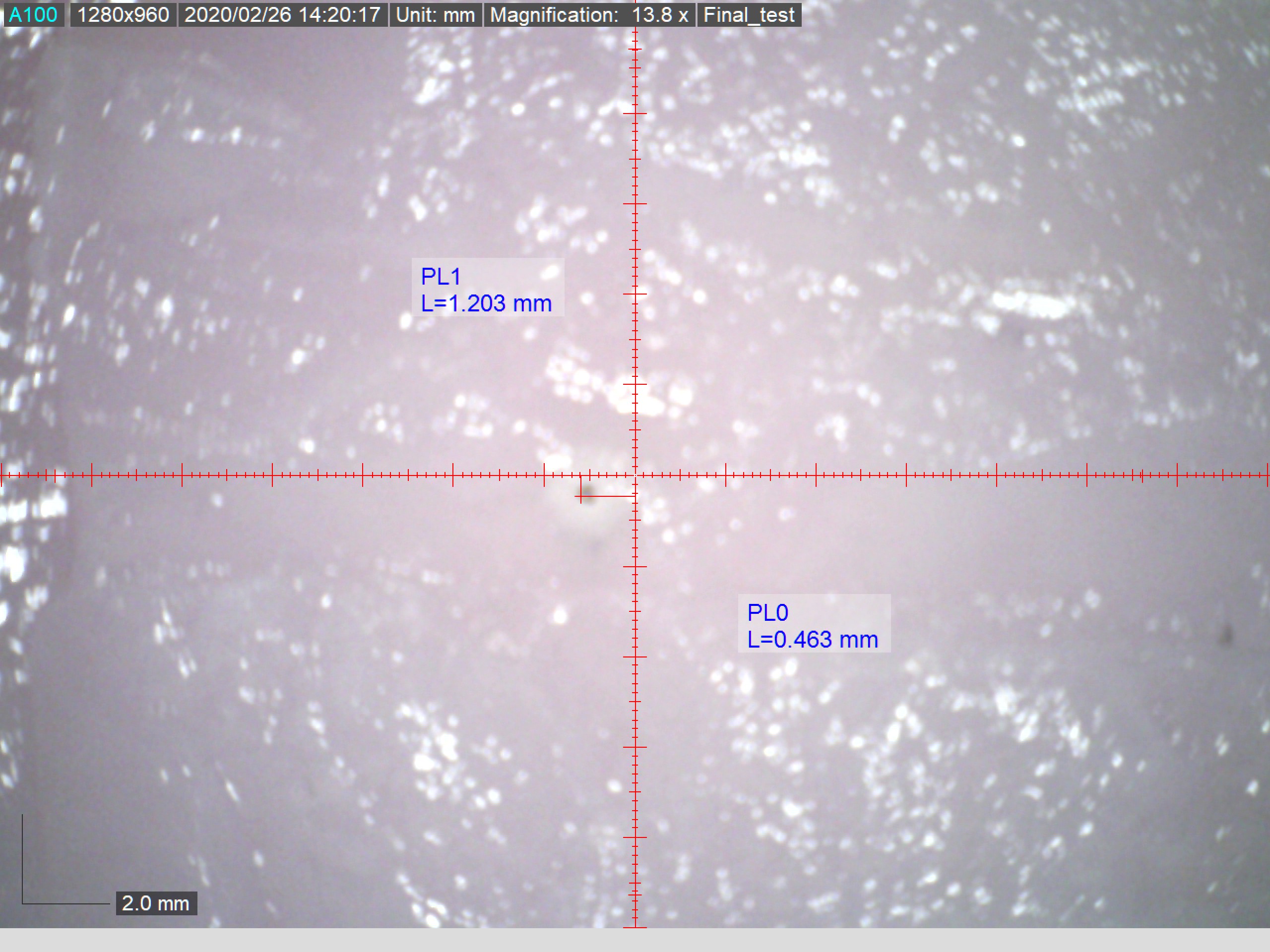

Supplement: S2 File — (ZIP) [file pone.0261089.s002.zip › Soft phantom/photos93.jpg]

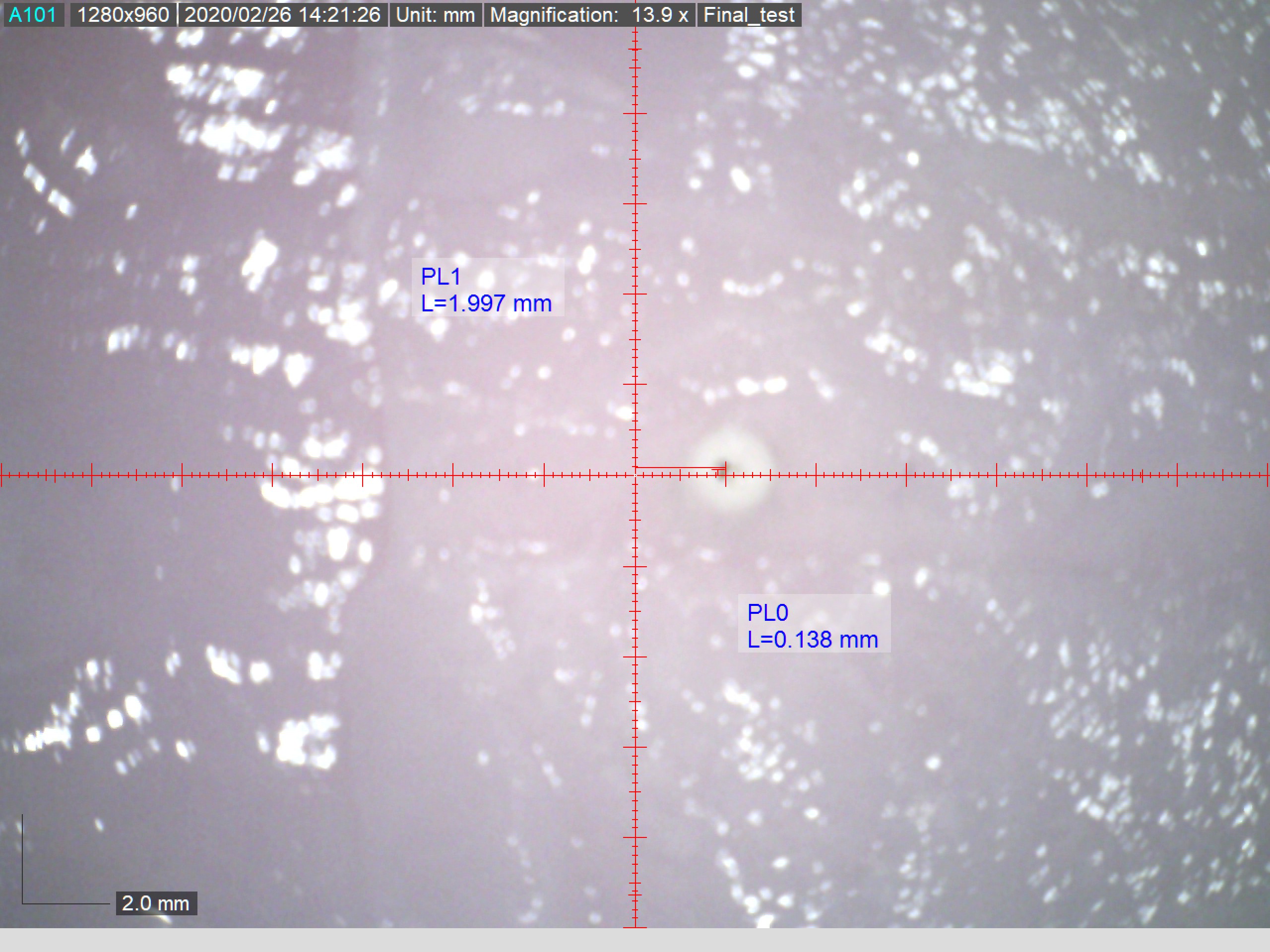

Supplement: S2 File — (ZIP) [file pone.0261089.s002.zip › Soft phantom/photos94.jpg]

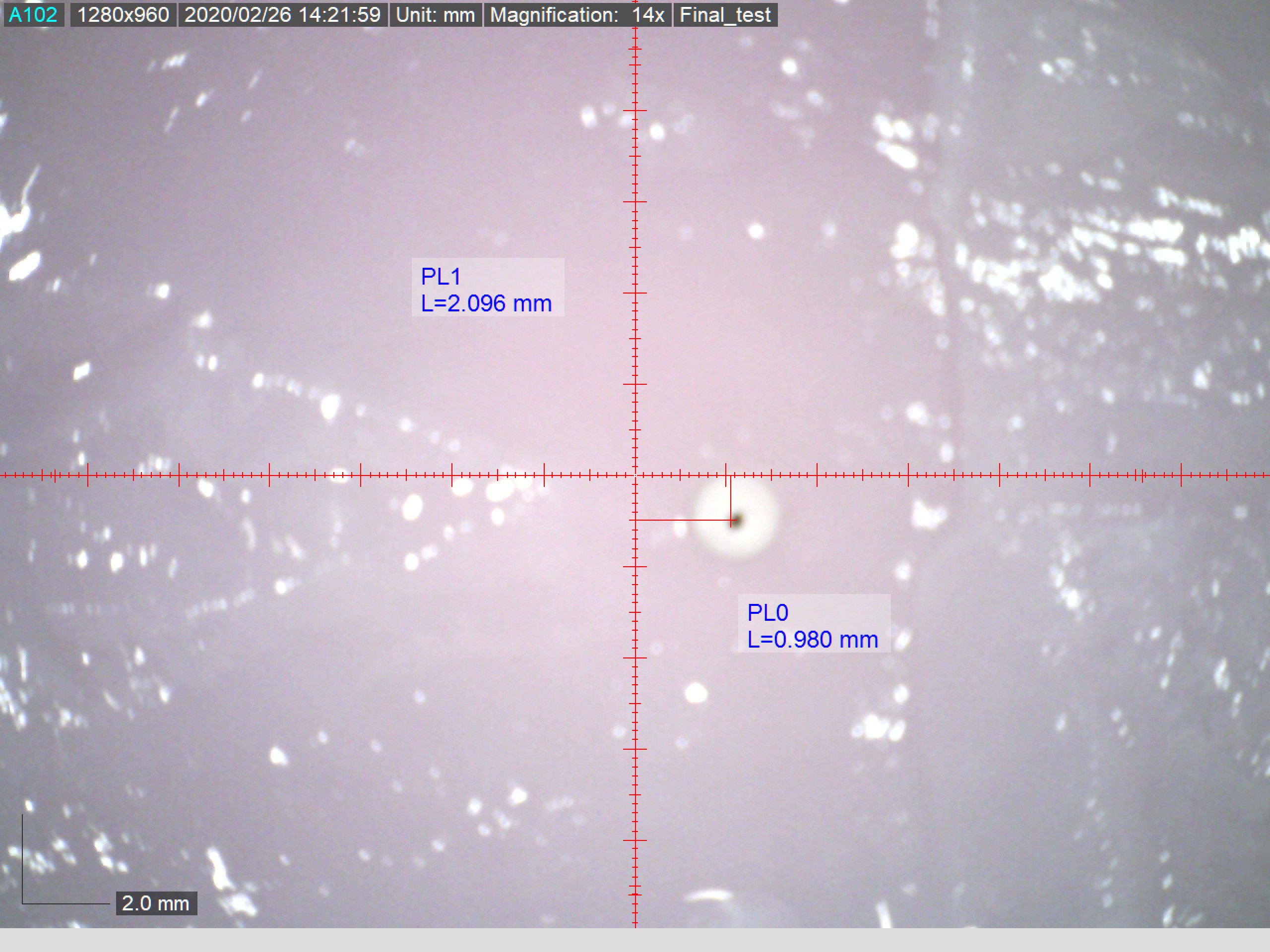

Supplement: S2 File — (ZIP) [file pone.0261089.s002.zip › Soft phantom/photos95.jpg]

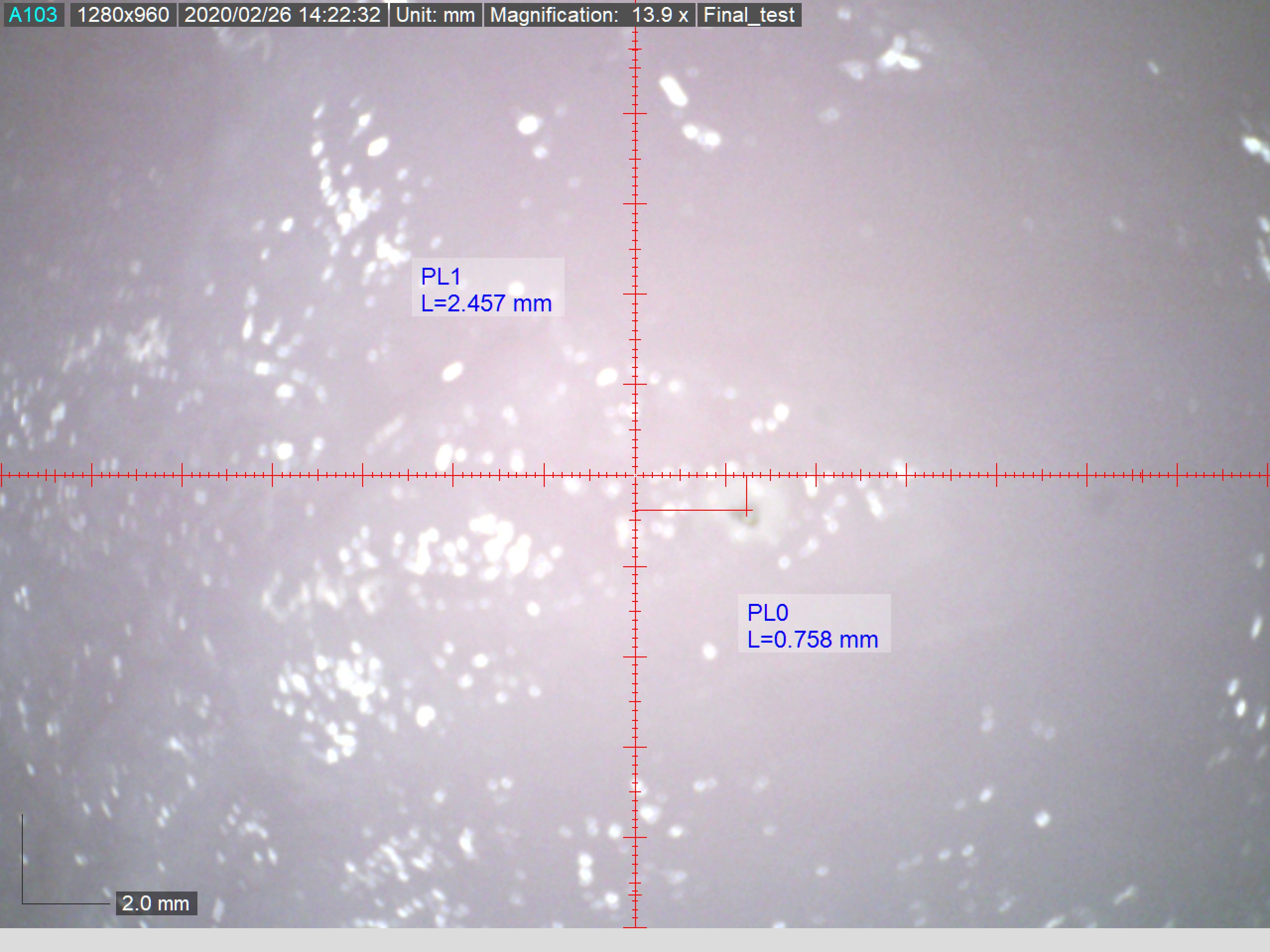

Supplement: S2 File — (ZIP) [file pone.0261089.s002.zip › Soft phantom/photos96.jpg]

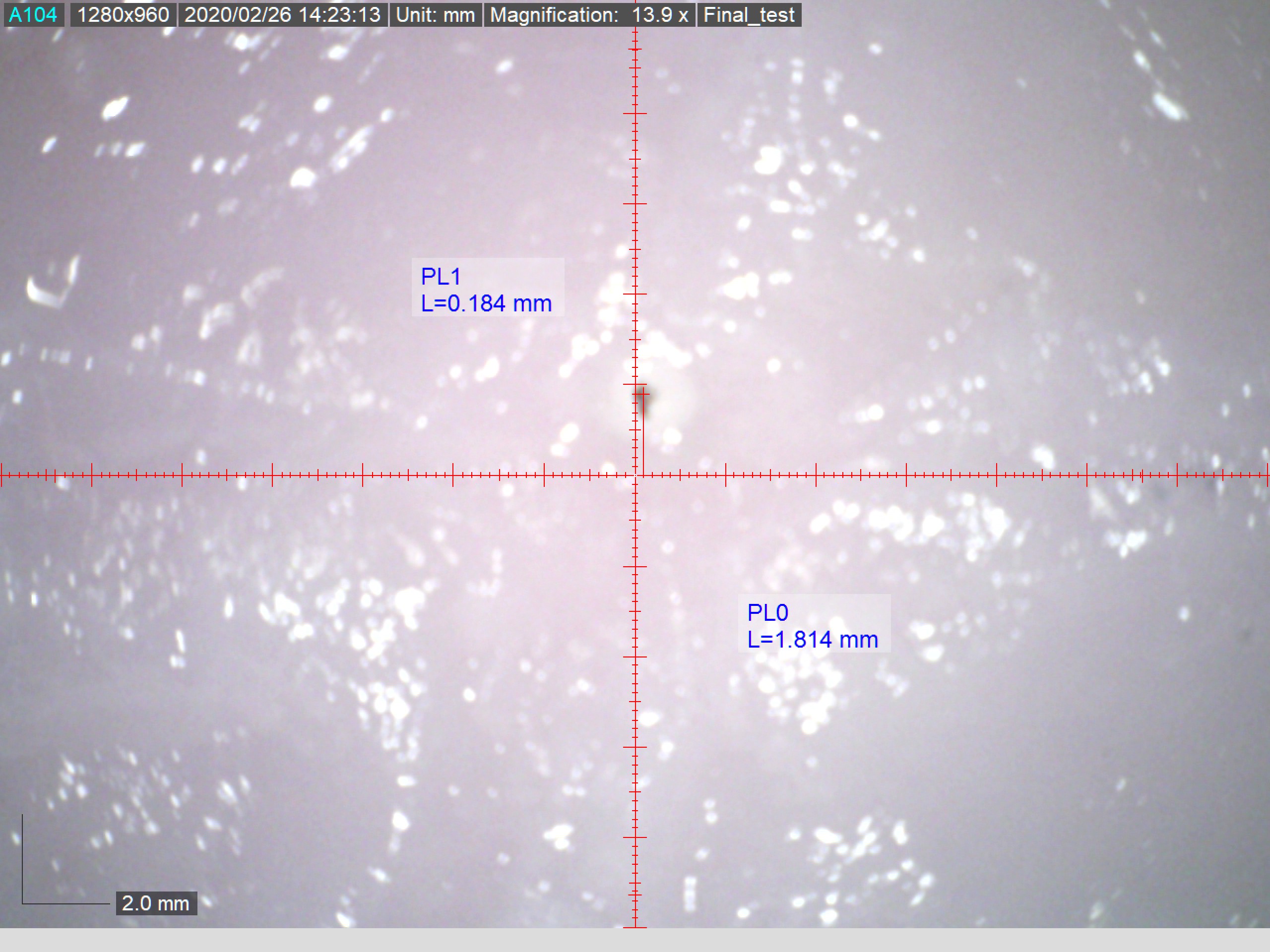

Supplement: S2 File — (ZIP) [file pone.0261089.s002.zip › Soft phantom/photos97.jpg]

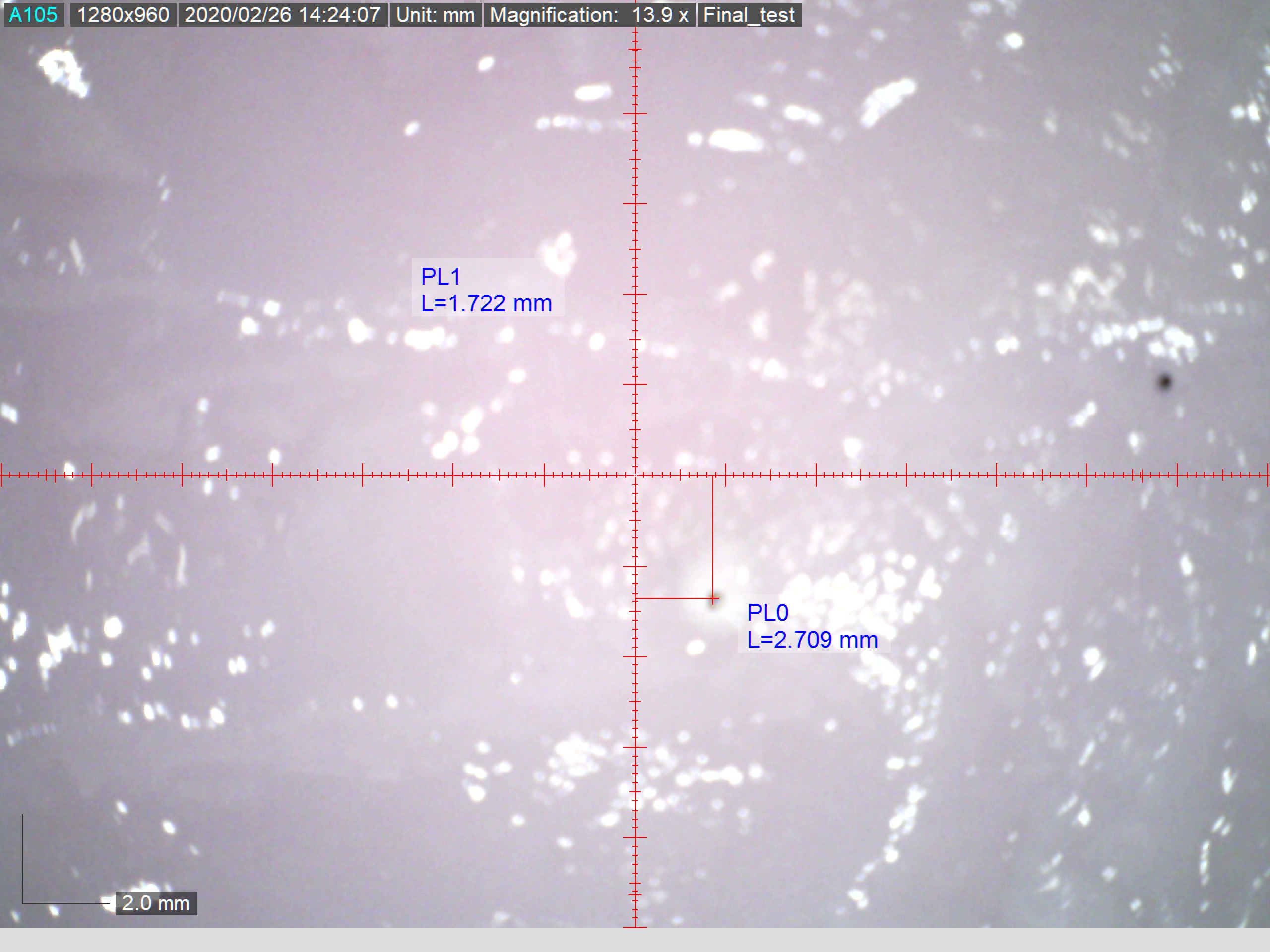

Supplement: S2 File — (ZIP) [file pone.0261089.s002.zip › Soft phantom/photos98.jpg]

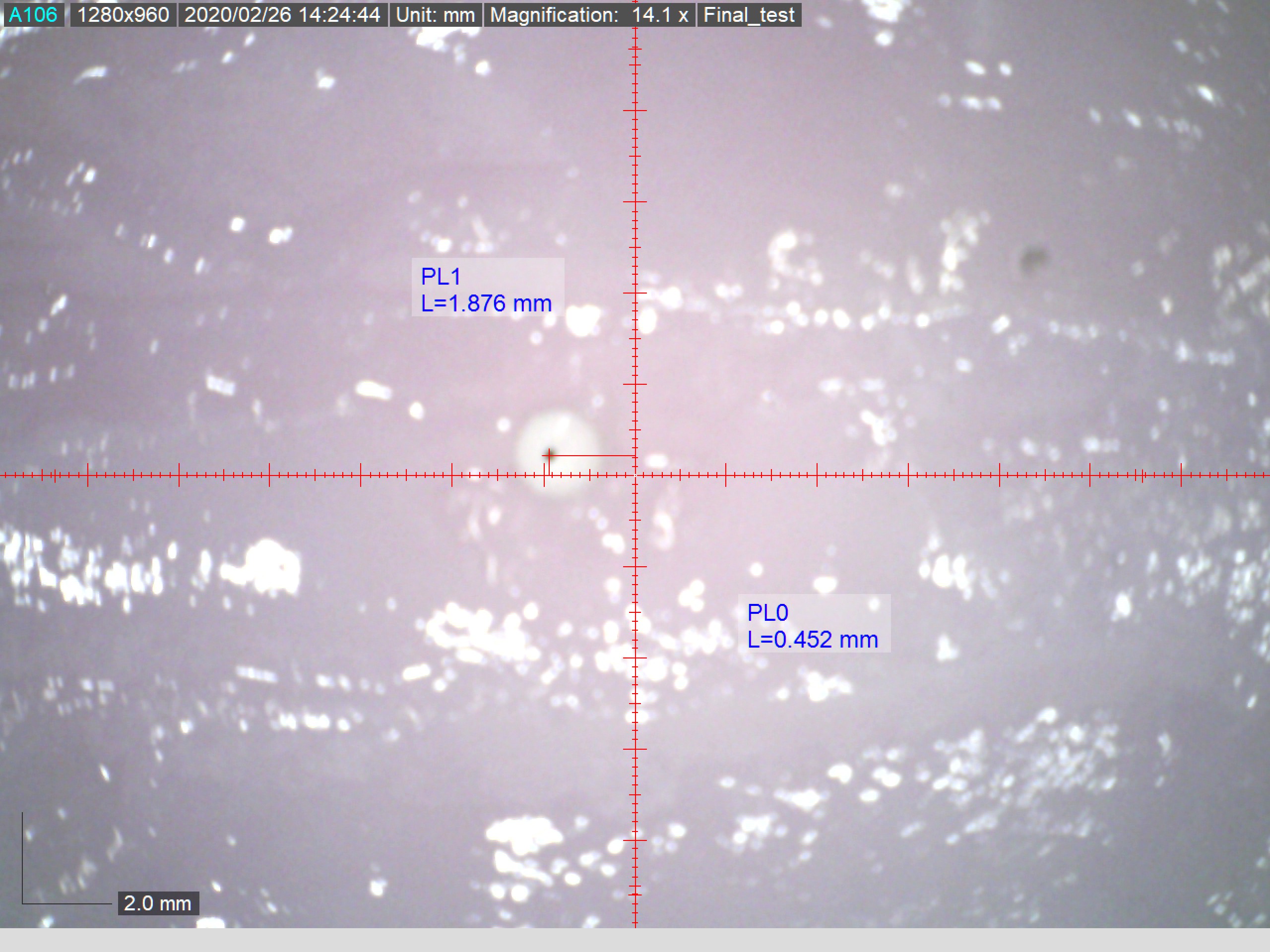

Supplement: S2 File — (ZIP) [file pone.0261089.s002.zip › Soft phantom/photos99.jpg]

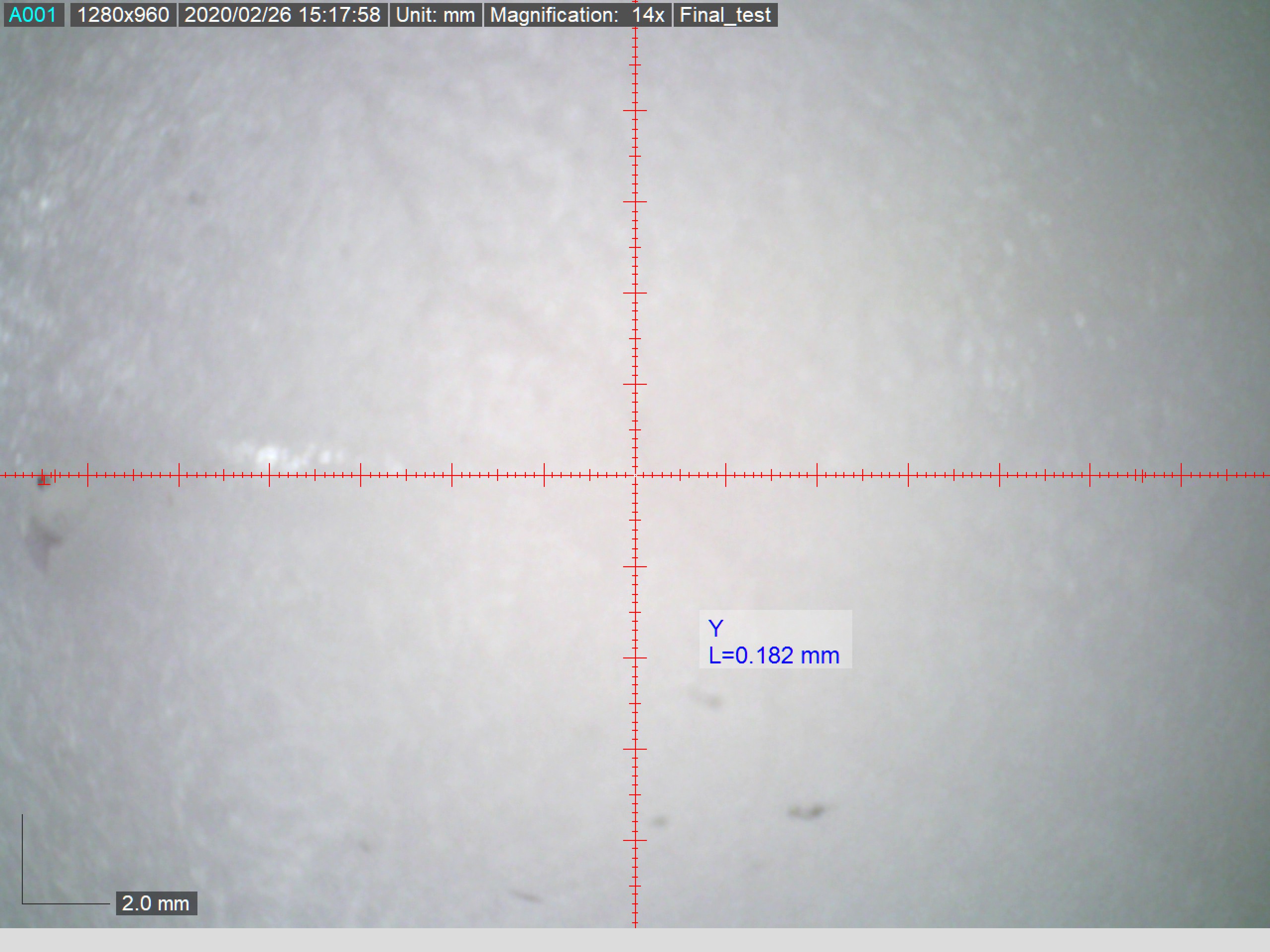

Supplement: S3 File — (ZIP) [file pone.0261089.s003.zip › Stiff phantom/fotos0.jpg]

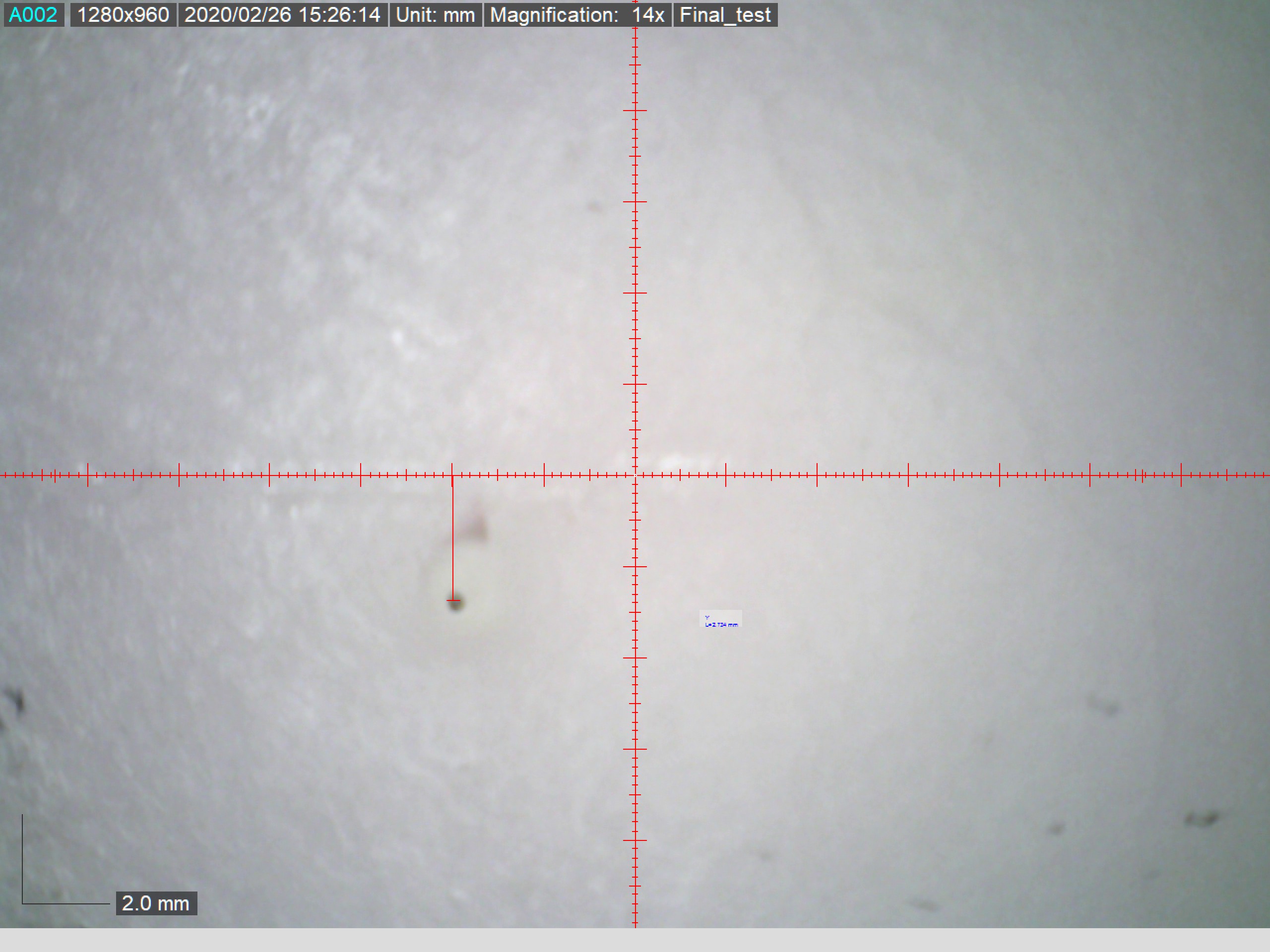

Supplement: S3 File — (ZIP) [file pone.0261089.s003.zip › Stiff phantom/fotos1.jpg]
