# Supplementary figures and images for: Axially rigid steerable needle with compliant active tip control (part 2 of 2)
Source: PLoS One. 2021 Dec 16;16(12):e0261089. doi: 10.1371/journal.pone.0261089 (PMC8675730; doi:10.1371/journal.pone.0261089)

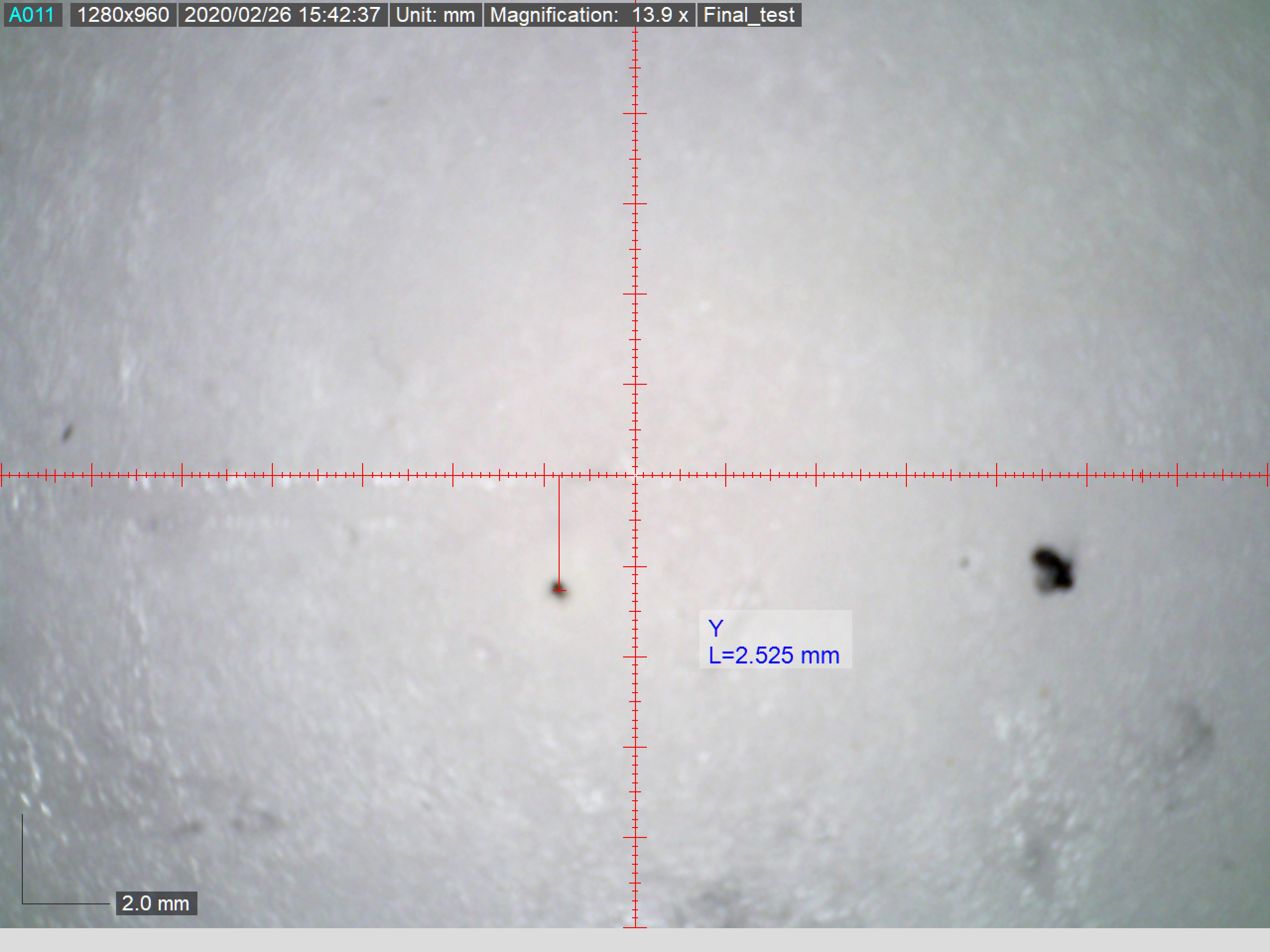

Supplement: S3 File — (ZIP) [file pone.0261089.s003.zip › Stiff phantom/fotos10.jpg]

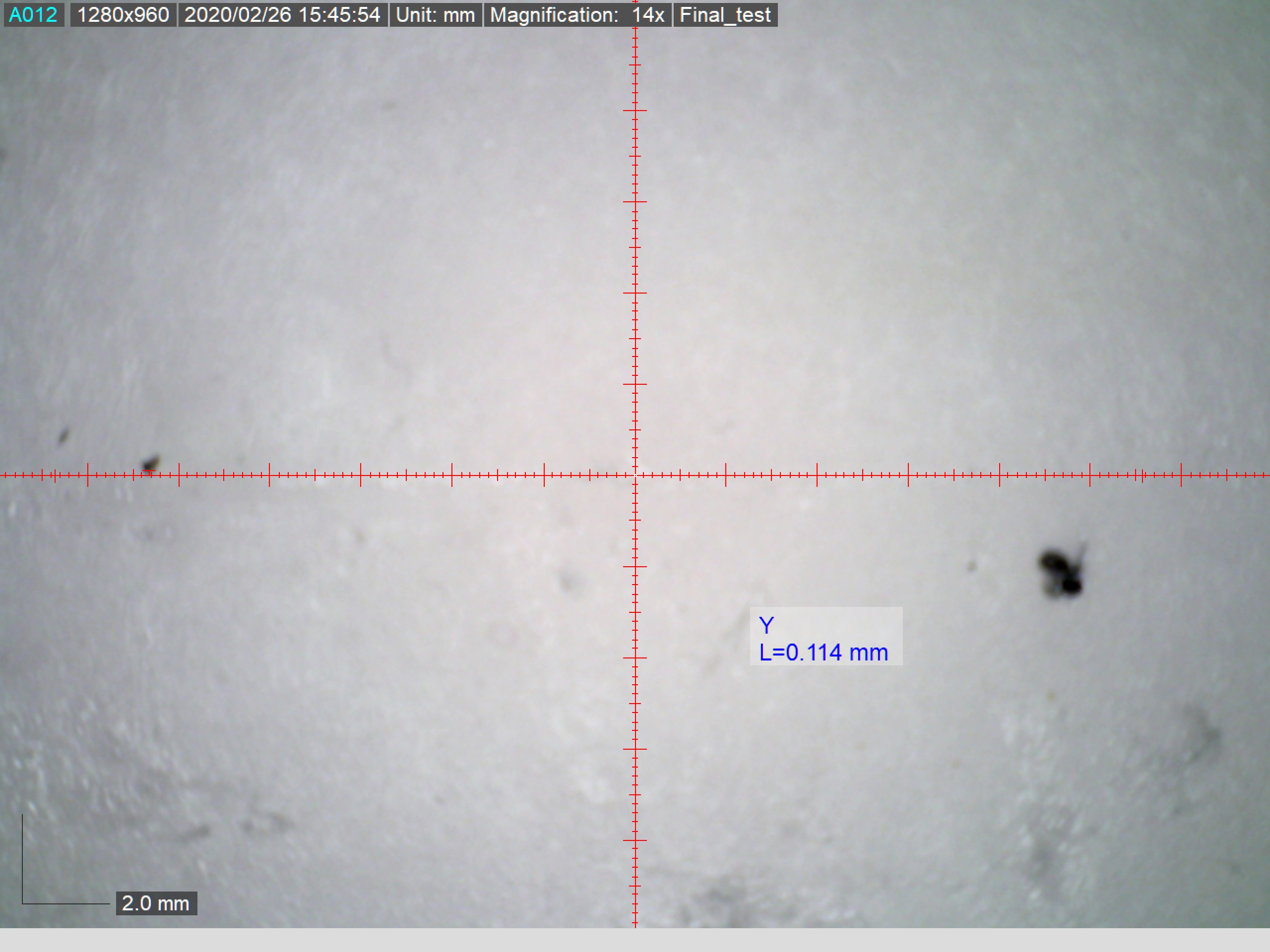

Supplement: S3 File — (ZIP) [file pone.0261089.s003.zip › Stiff phantom/fotos11.jpg]

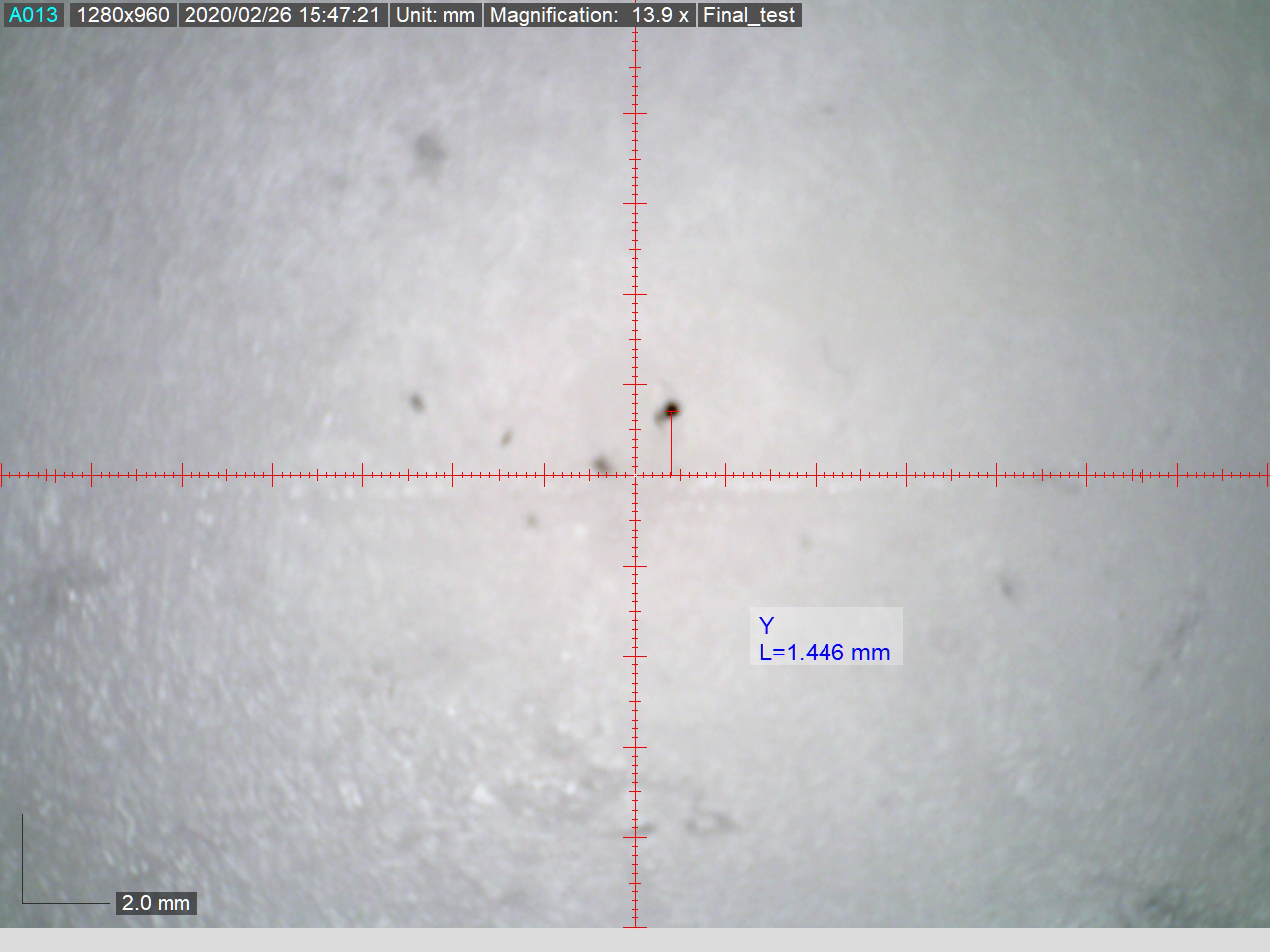

Supplement: S3 File — (ZIP) [file pone.0261089.s003.zip › Stiff phantom/fotos12.jpg]

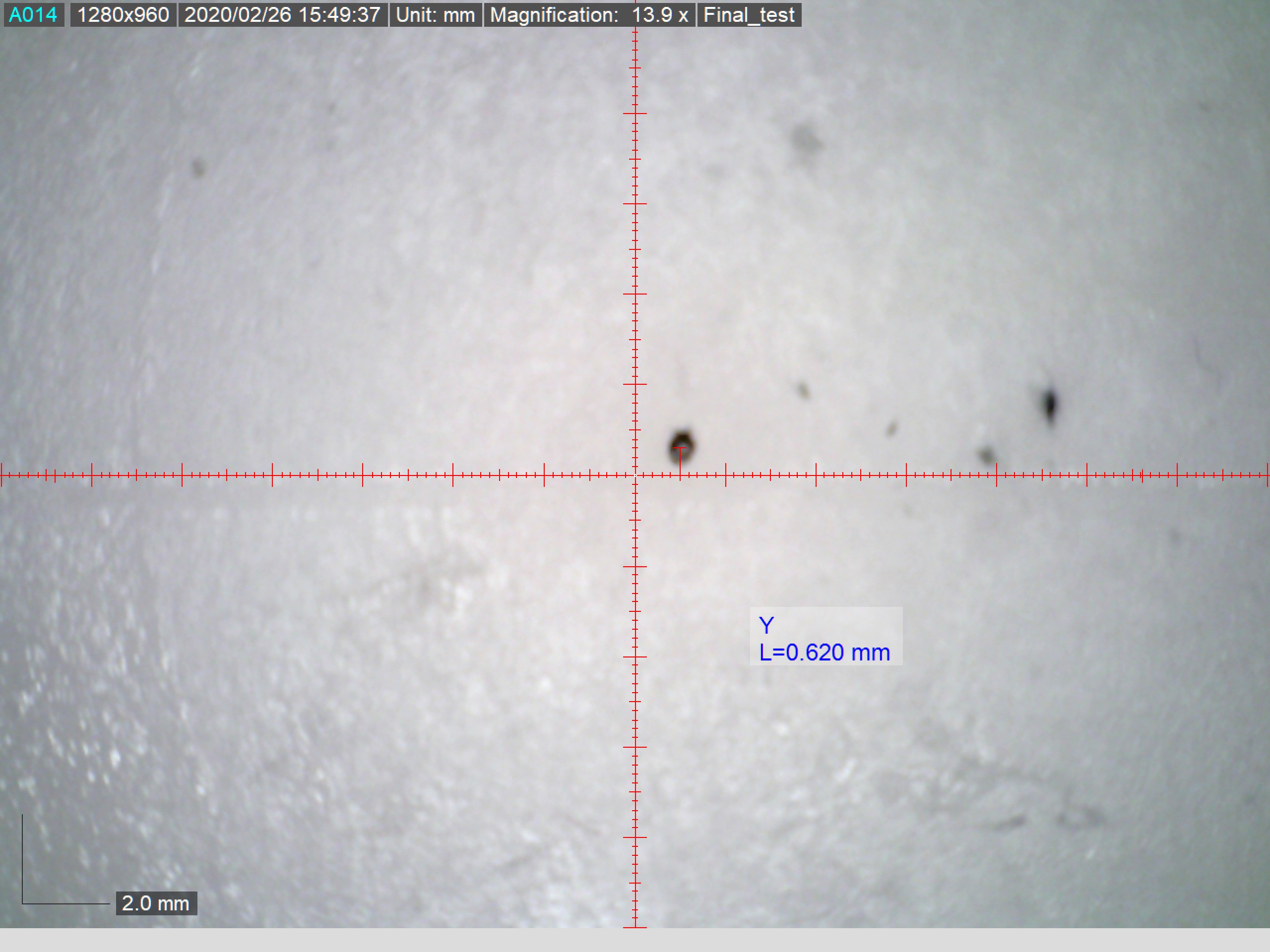

Supplement: S3 File — (ZIP) [file pone.0261089.s003.zip › Stiff phantom/fotos13.jpg]

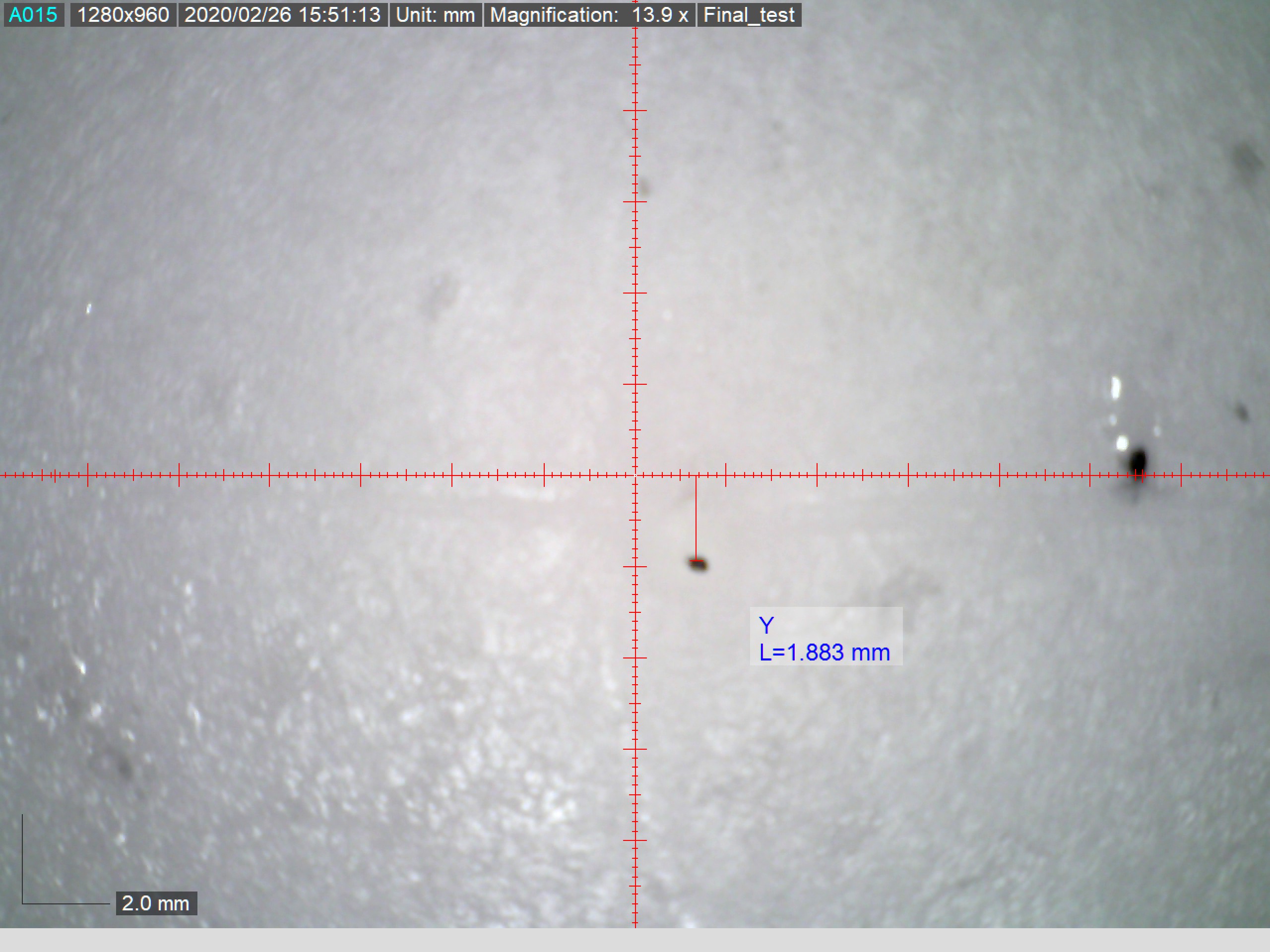

Supplement: S3 File — (ZIP) [file pone.0261089.s003.zip › Stiff phantom/fotos14.jpg]

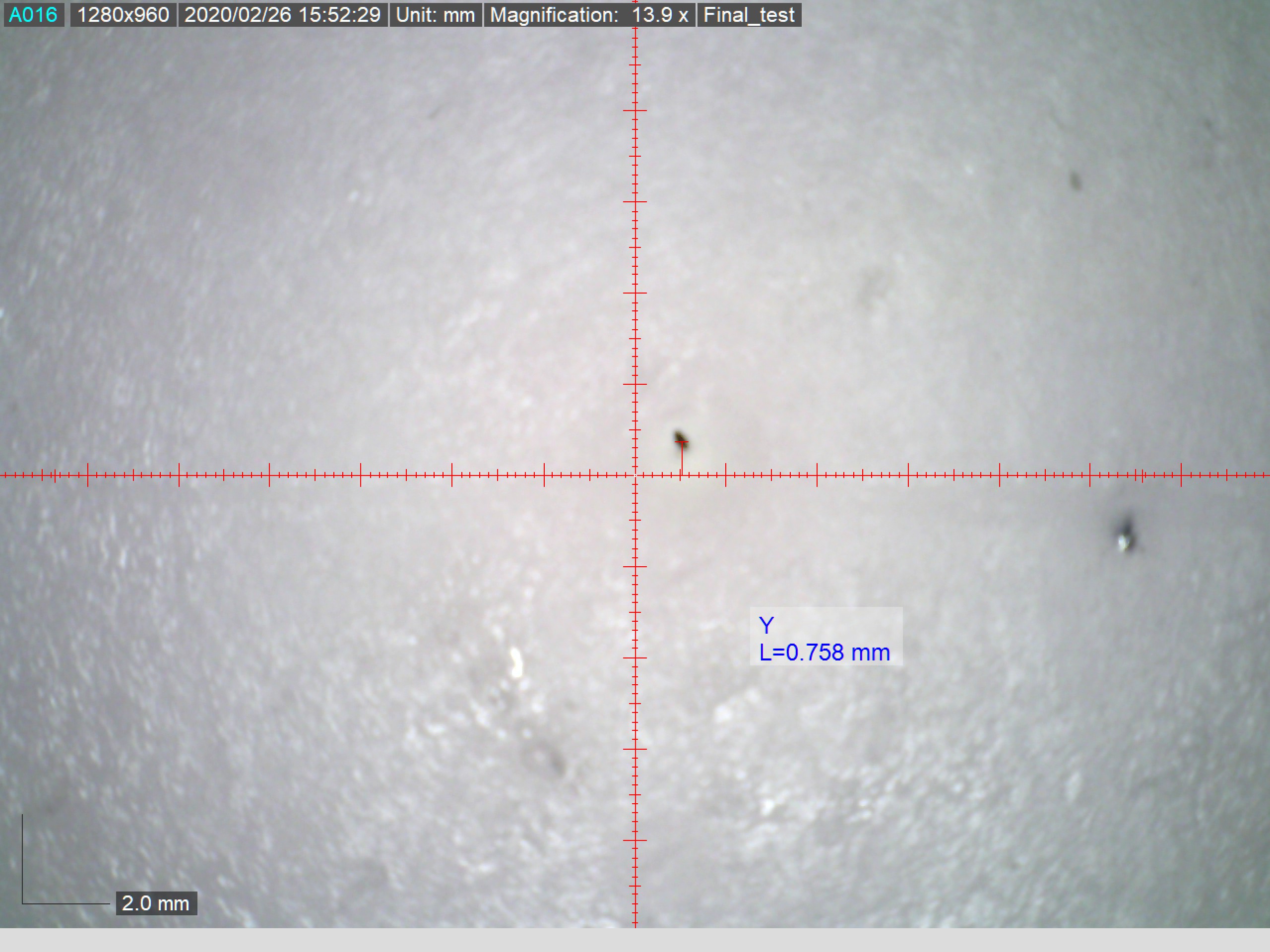

Supplement: S3 File — (ZIP) [file pone.0261089.s003.zip › Stiff phantom/fotos15.jpg]

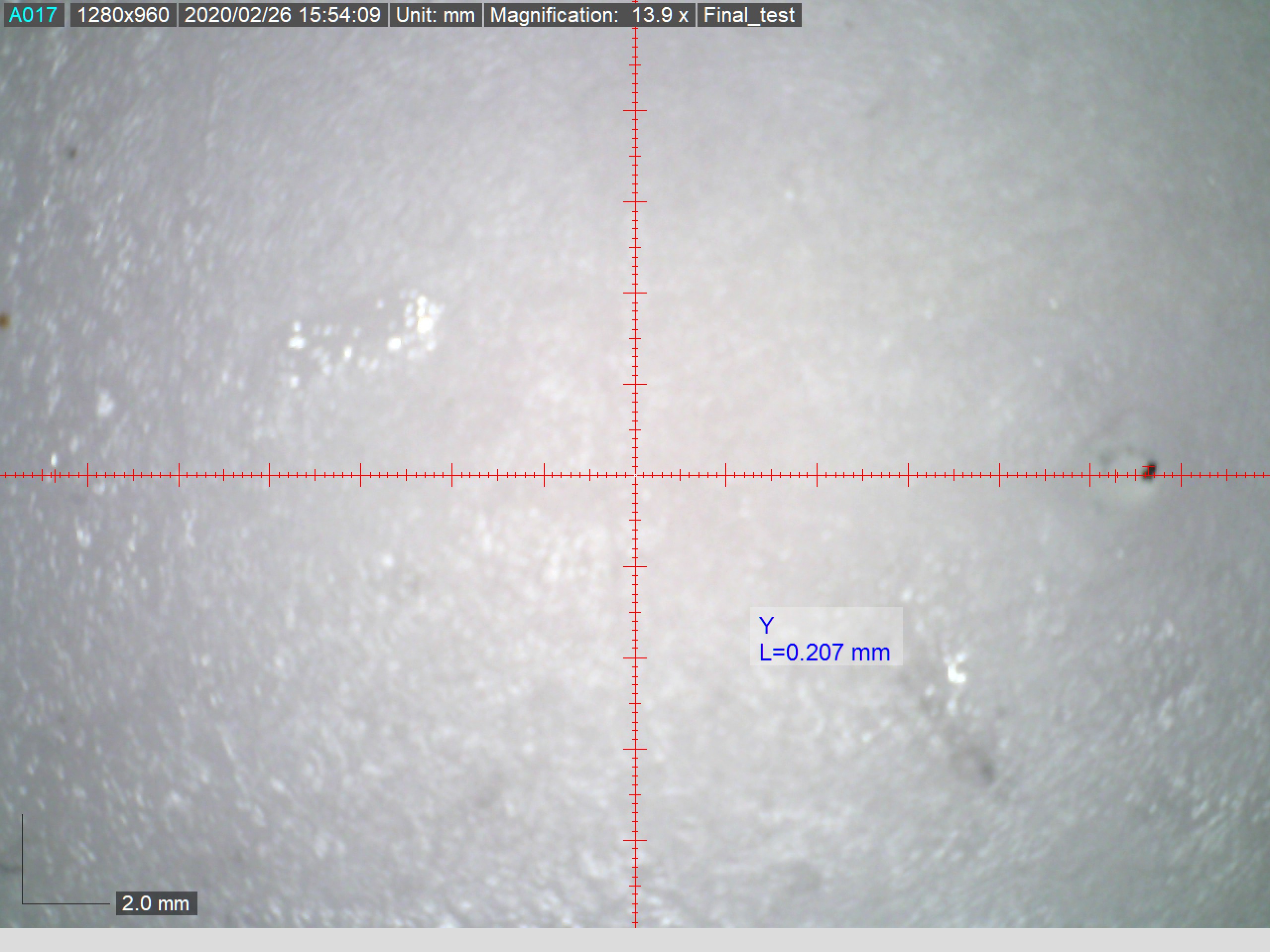

Supplement: S3 File — (ZIP) [file pone.0261089.s003.zip › Stiff phantom/fotos16.jpg]

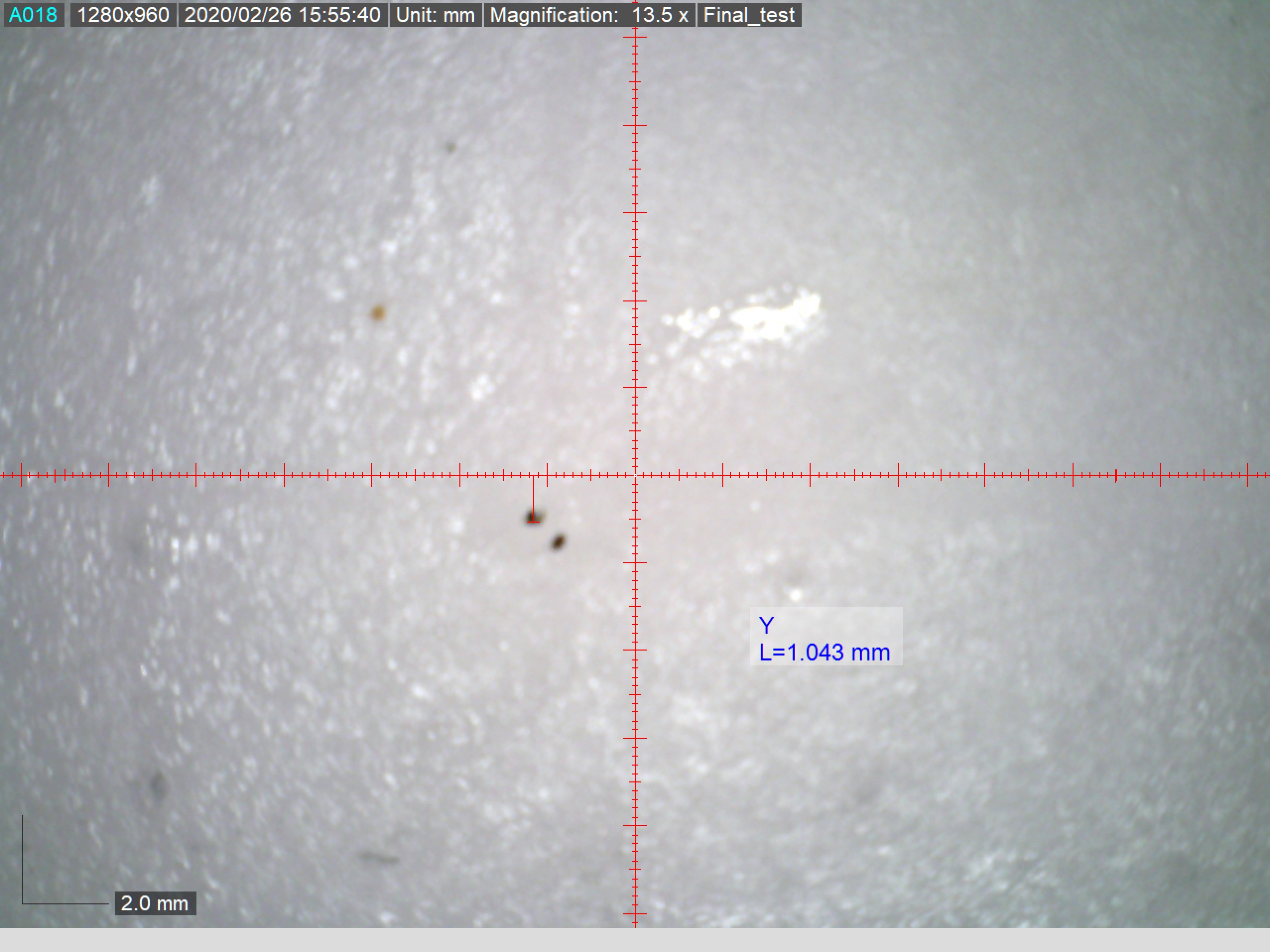

Supplement: S3 File — (ZIP) [file pone.0261089.s003.zip › Stiff phantom/fotos17.jpg]

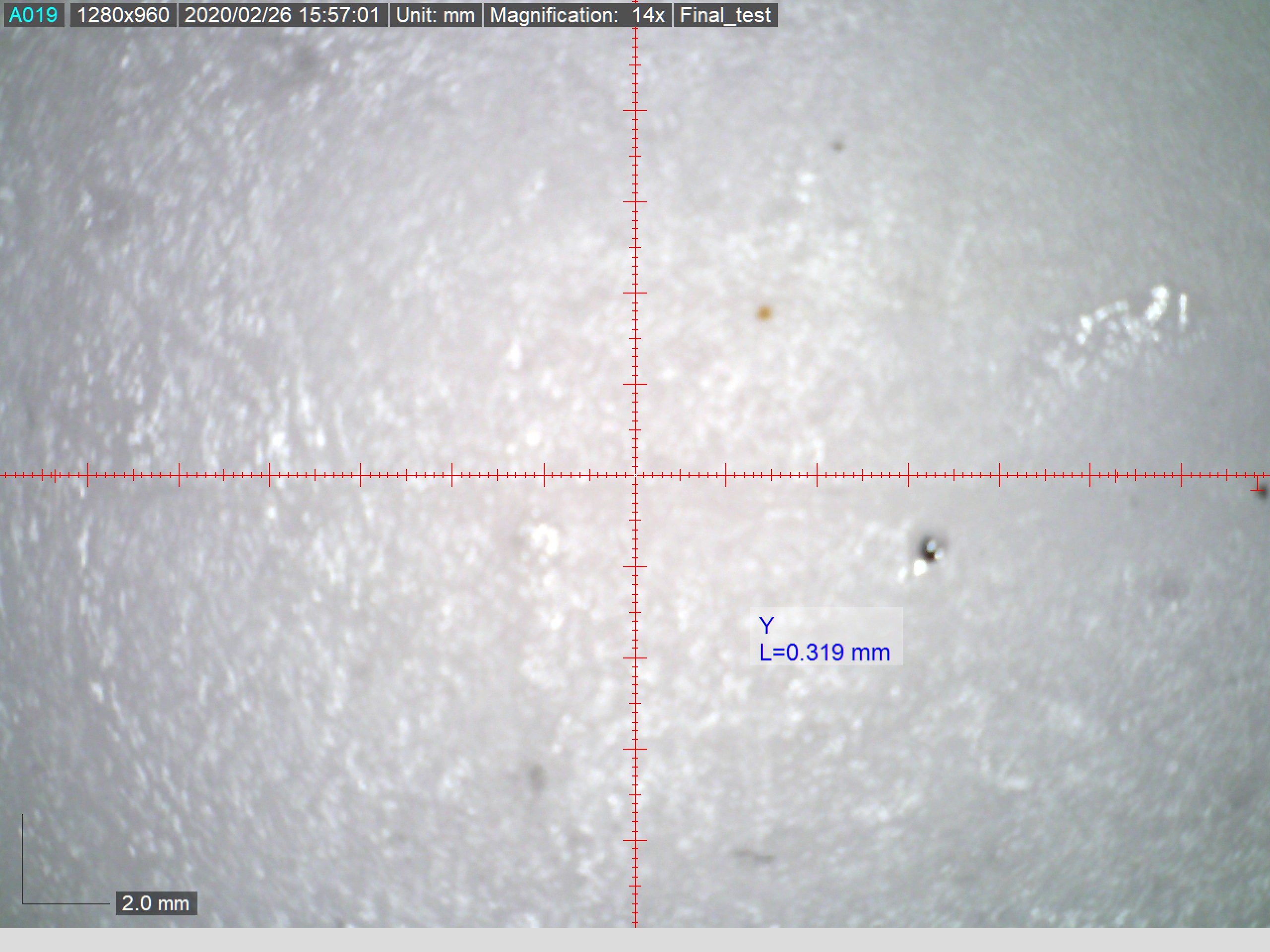

Supplement: S3 File — (ZIP) [file pone.0261089.s003.zip › Stiff phantom/fotos18.jpg]

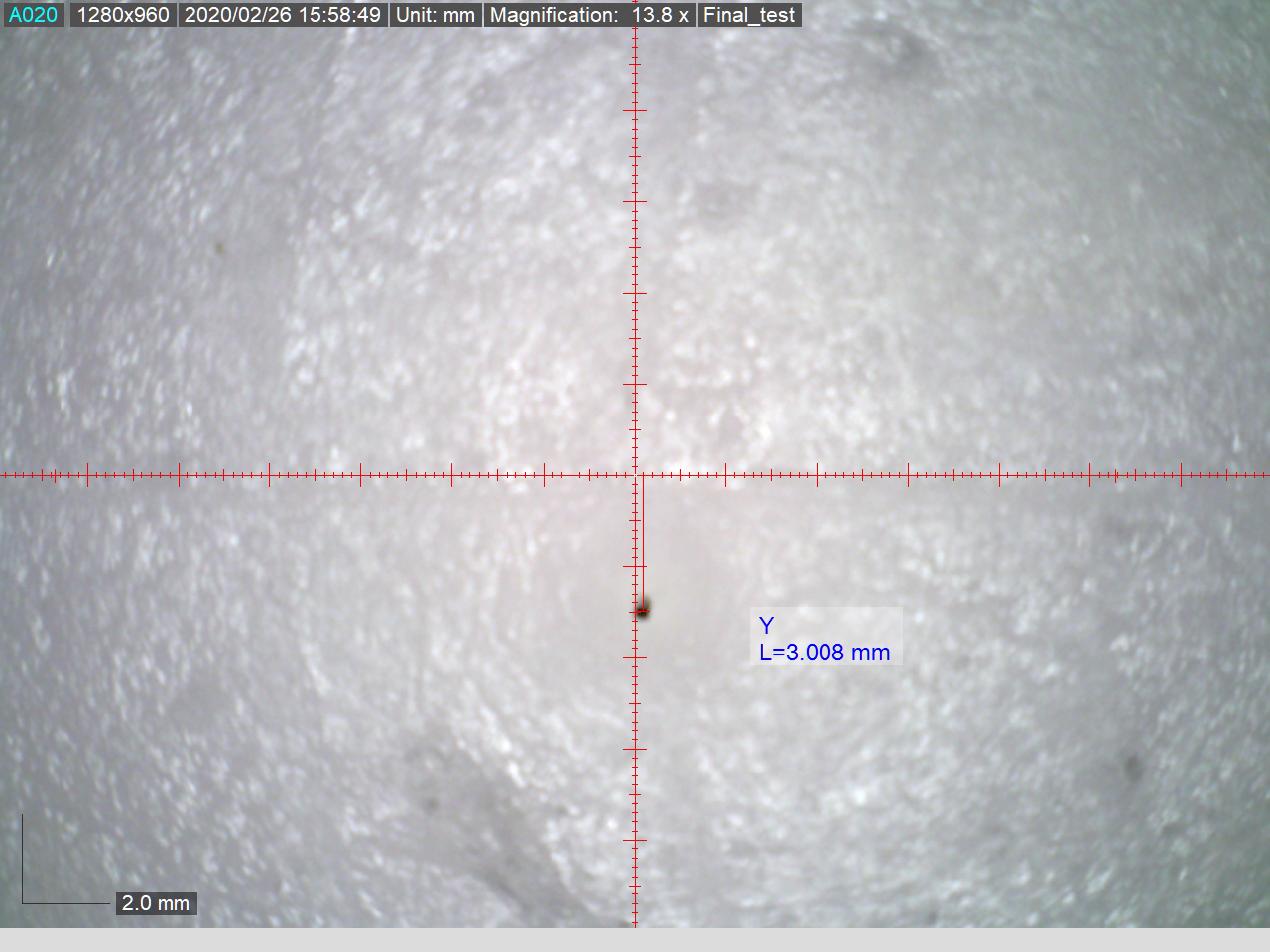

Supplement: S3 File — (ZIP) [file pone.0261089.s003.zip › Stiff phantom/fotos19.jpg]

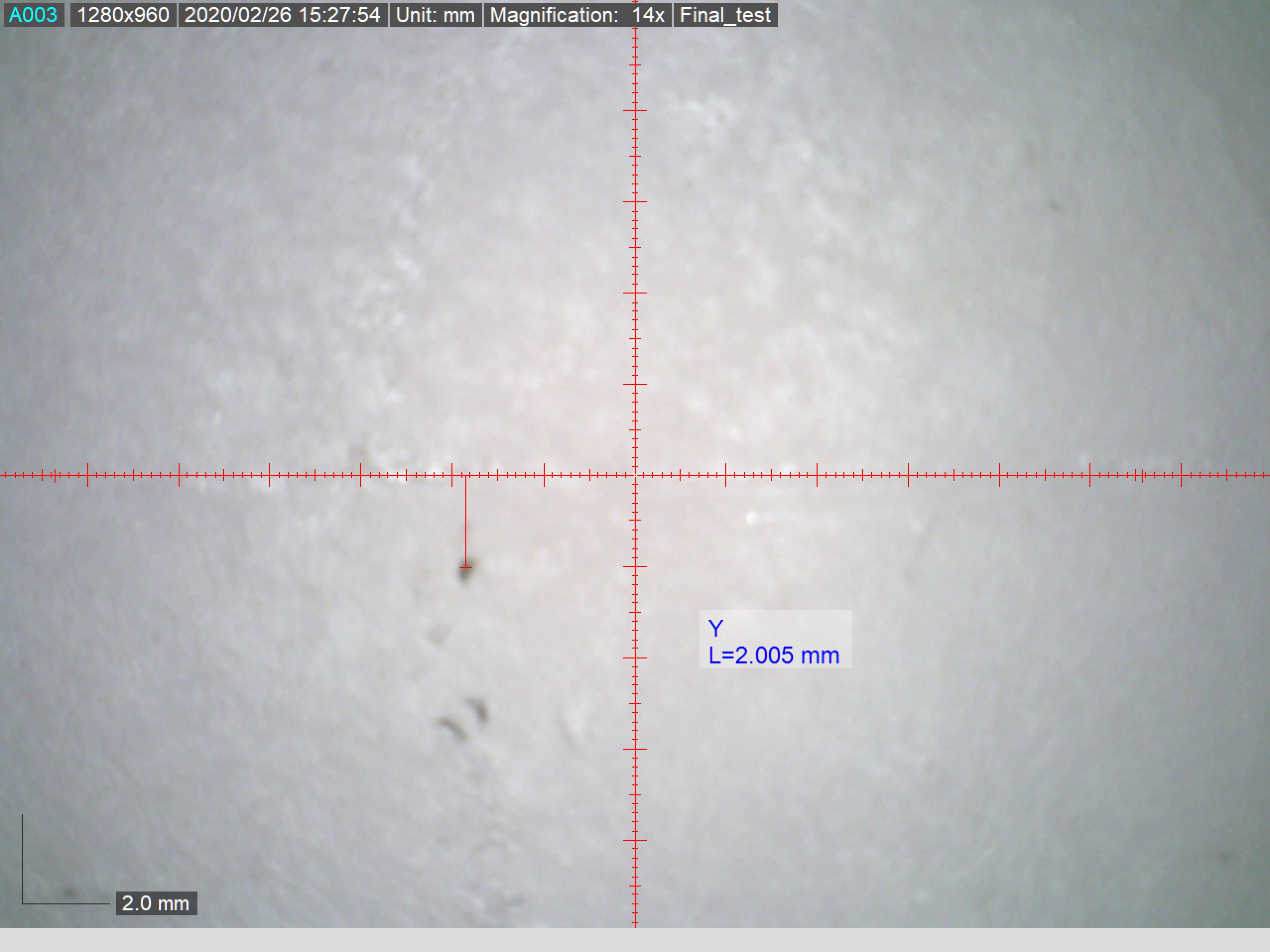

Supplement: S3 File — (ZIP) [file pone.0261089.s003.zip › Stiff phantom/fotos2.jpg]

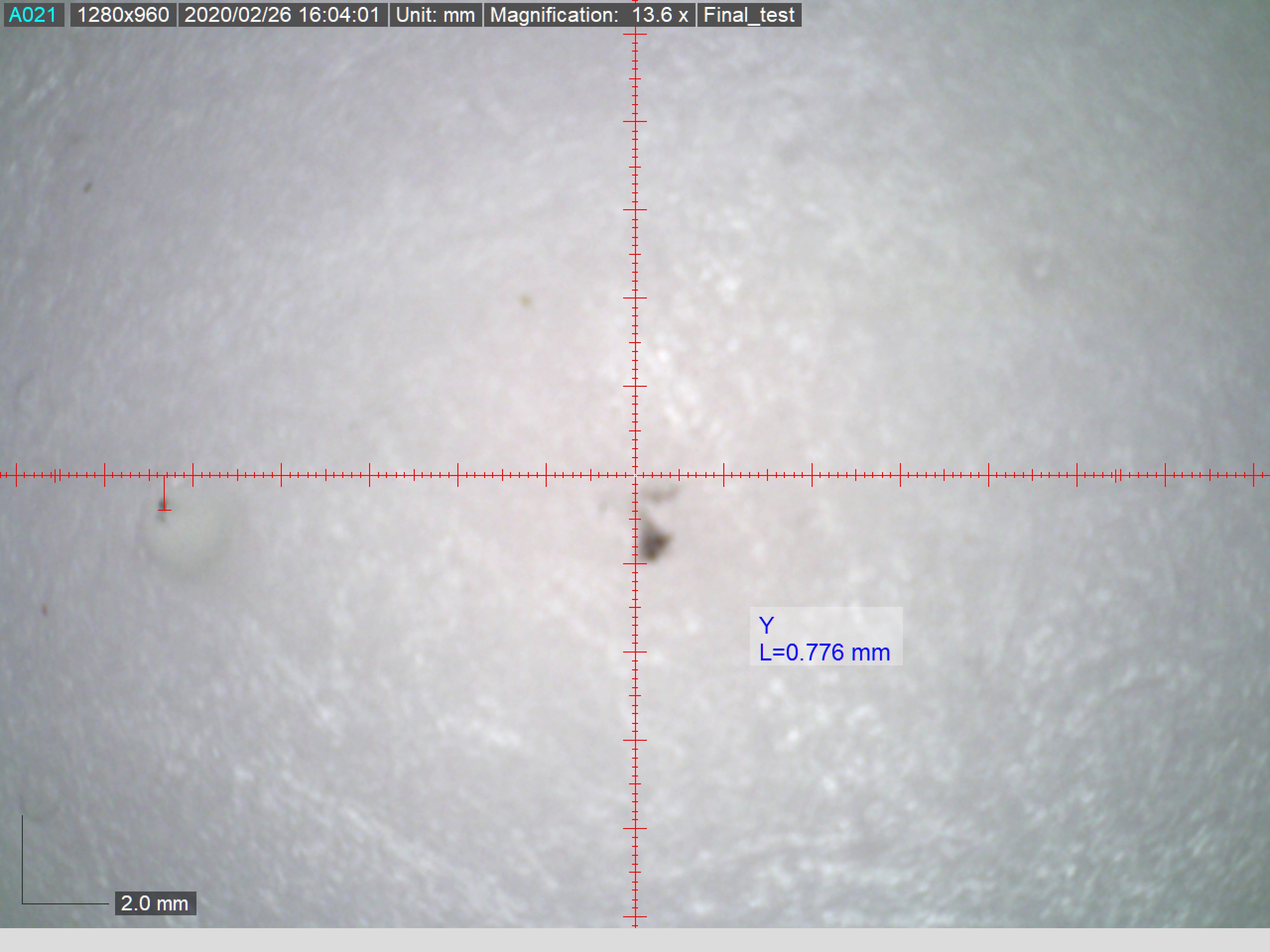

Supplement: S3 File — (ZIP) [file pone.0261089.s003.zip › Stiff phantom/fotos20.jpg]

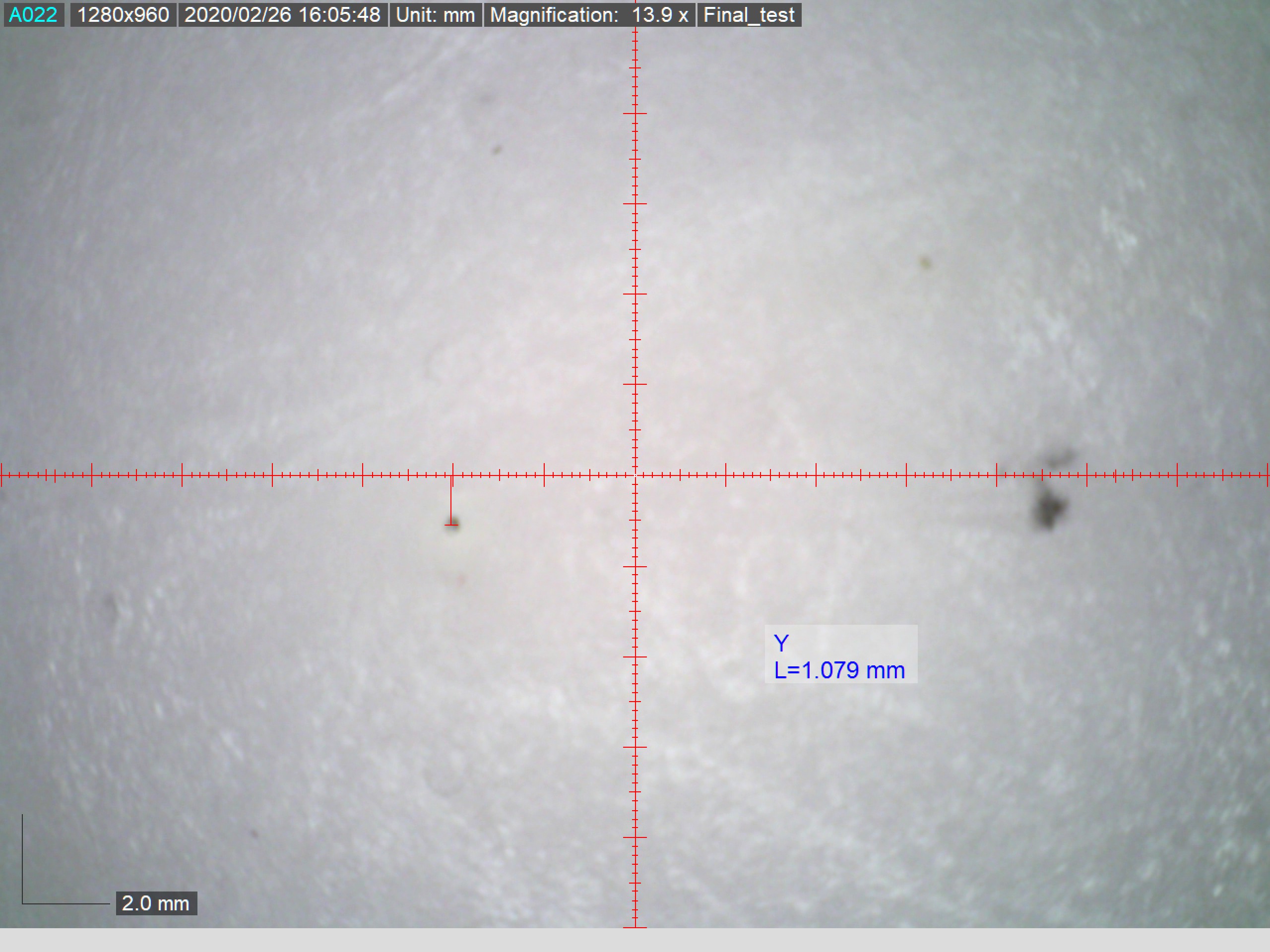

Supplement: S3 File — (ZIP) [file pone.0261089.s003.zip › Stiff phantom/fotos21.jpg]

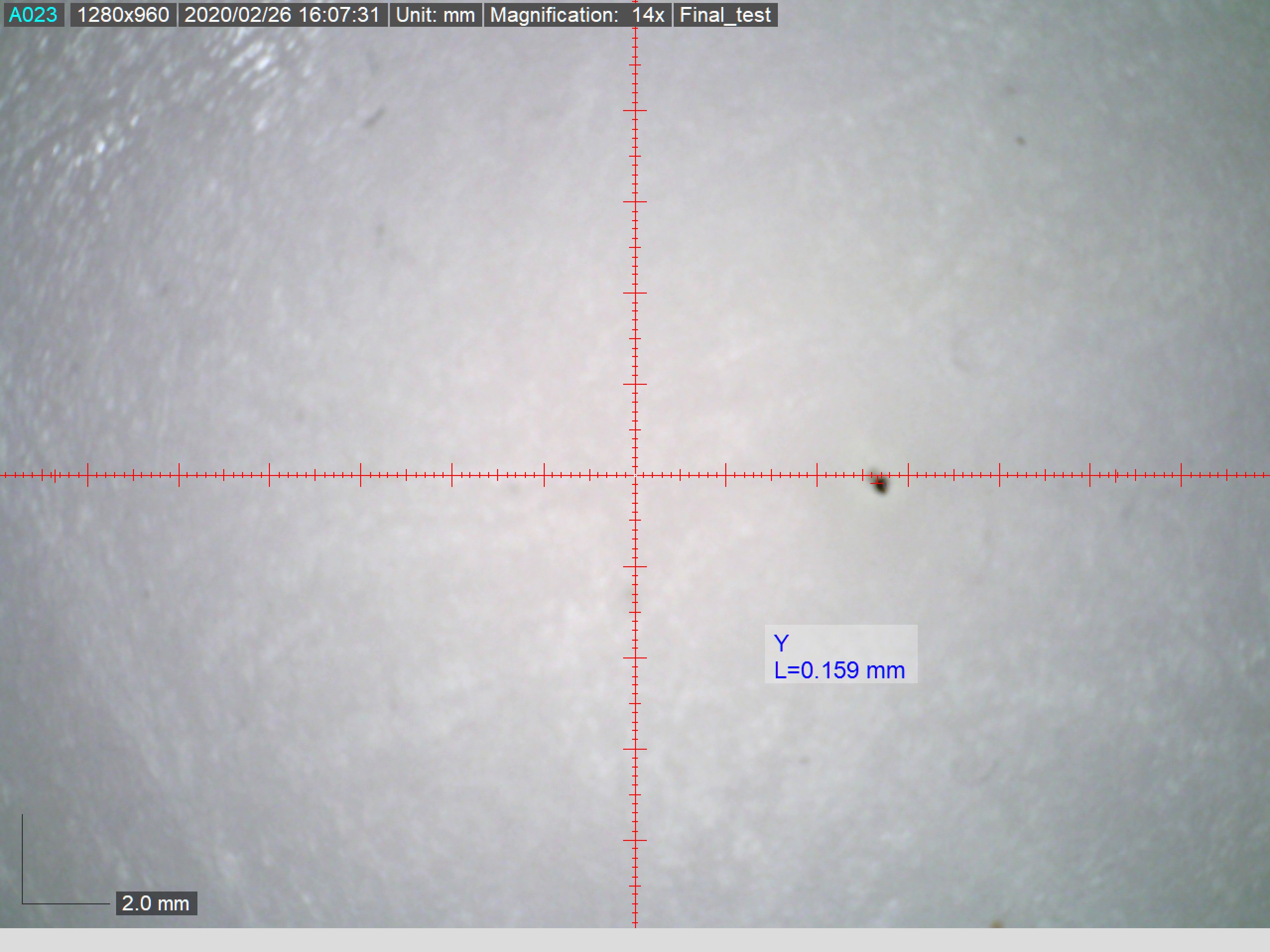

Supplement: S3 File — (ZIP) [file pone.0261089.s003.zip › Stiff phantom/fotos22.jpg]

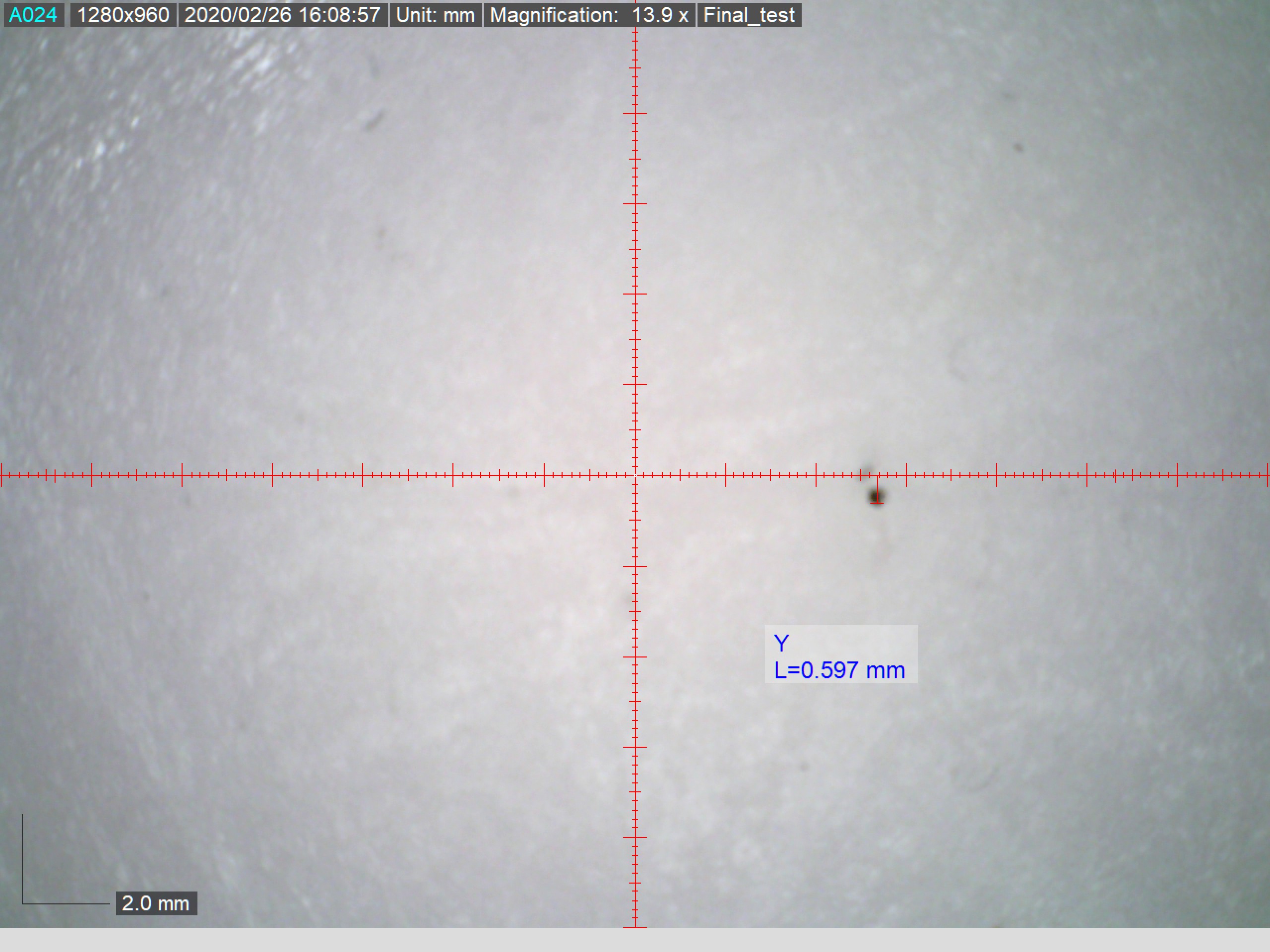

Supplement: S3 File — (ZIP) [file pone.0261089.s003.zip › Stiff phantom/fotos23.jpg]

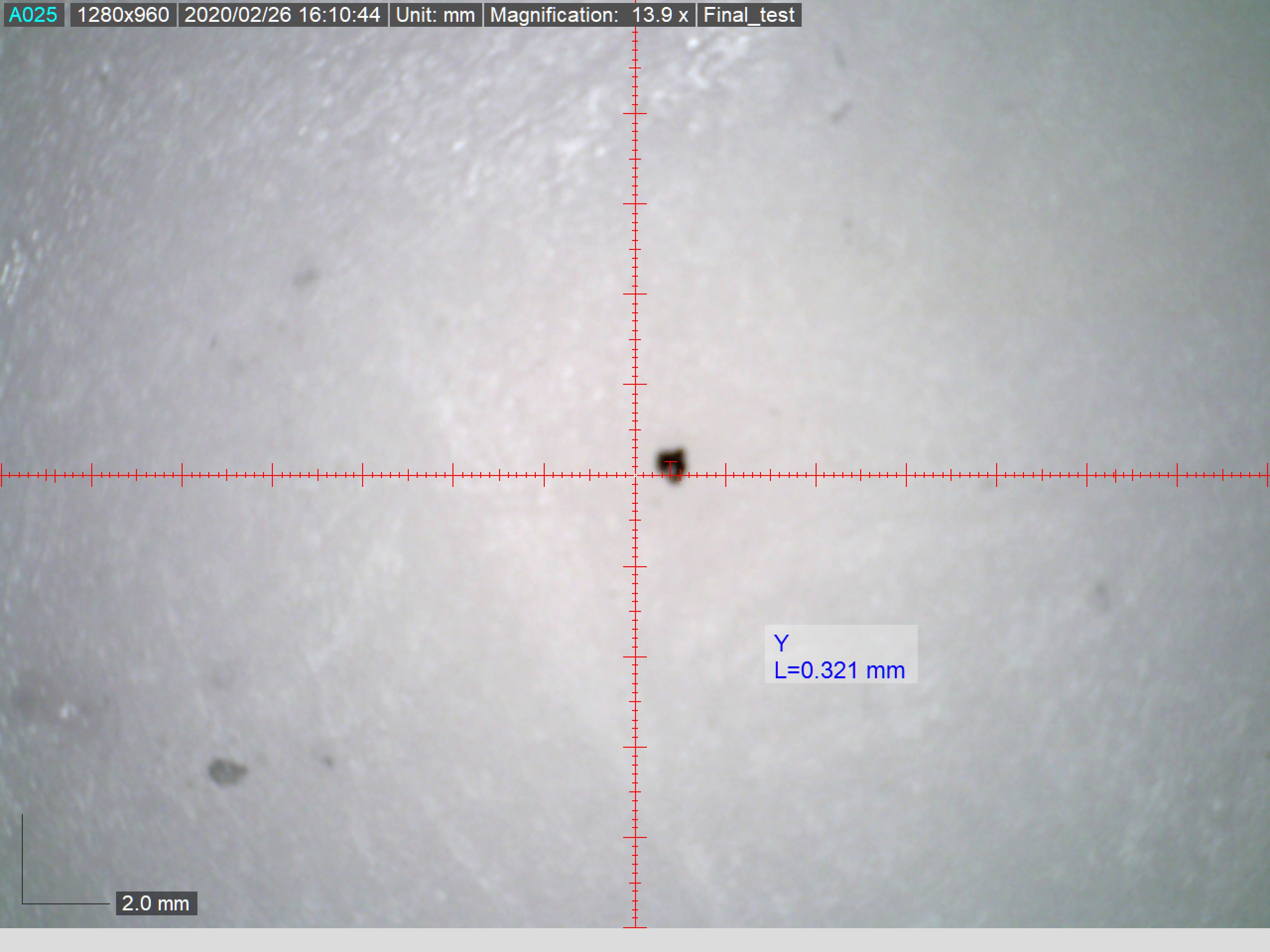

Supplement: S3 File — (ZIP) [file pone.0261089.s003.zip › Stiff phantom/fotos24.jpg]

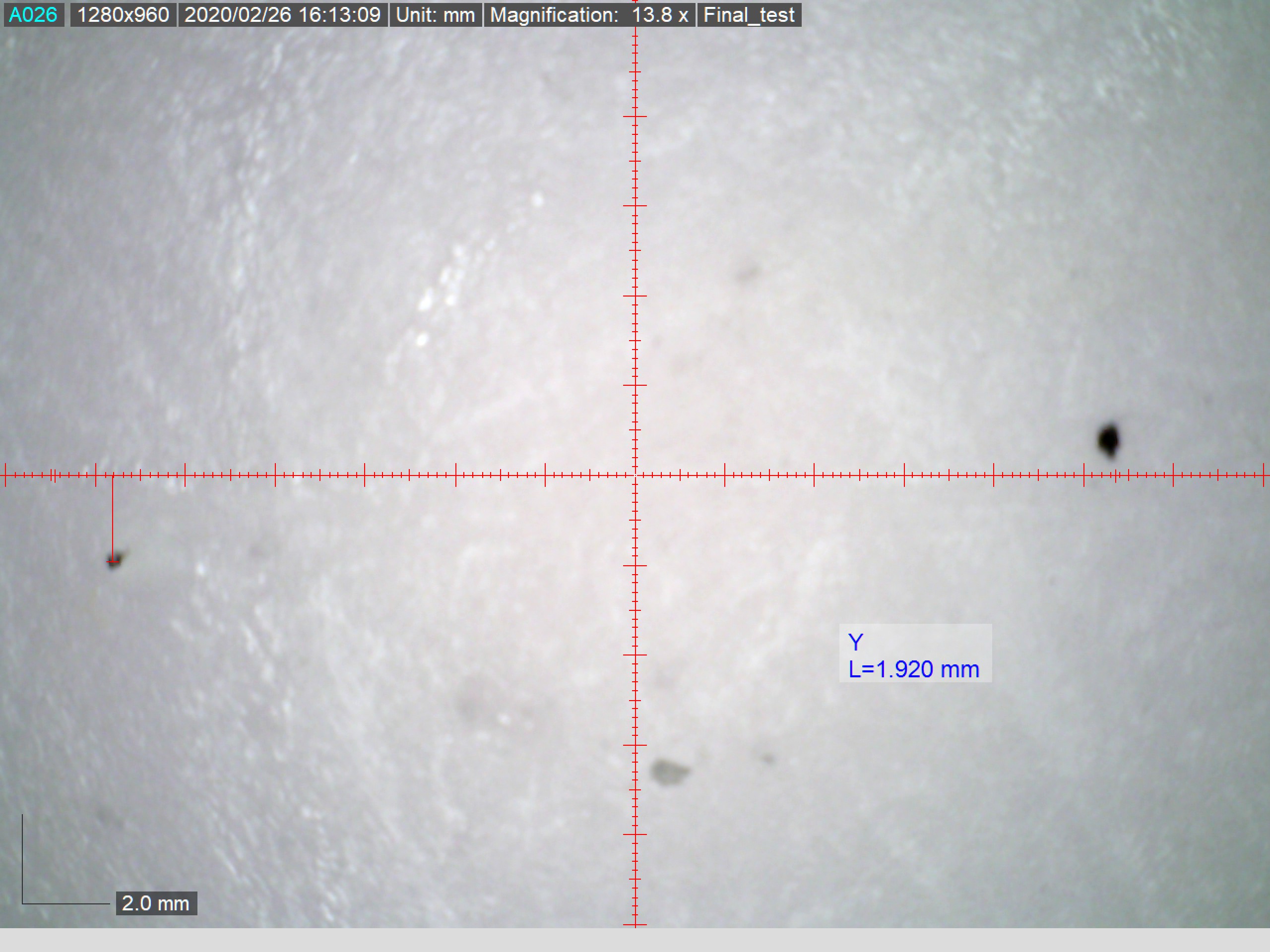

Supplement: S3 File — (ZIP) [file pone.0261089.s003.zip › Stiff phantom/fotos25.jpg]

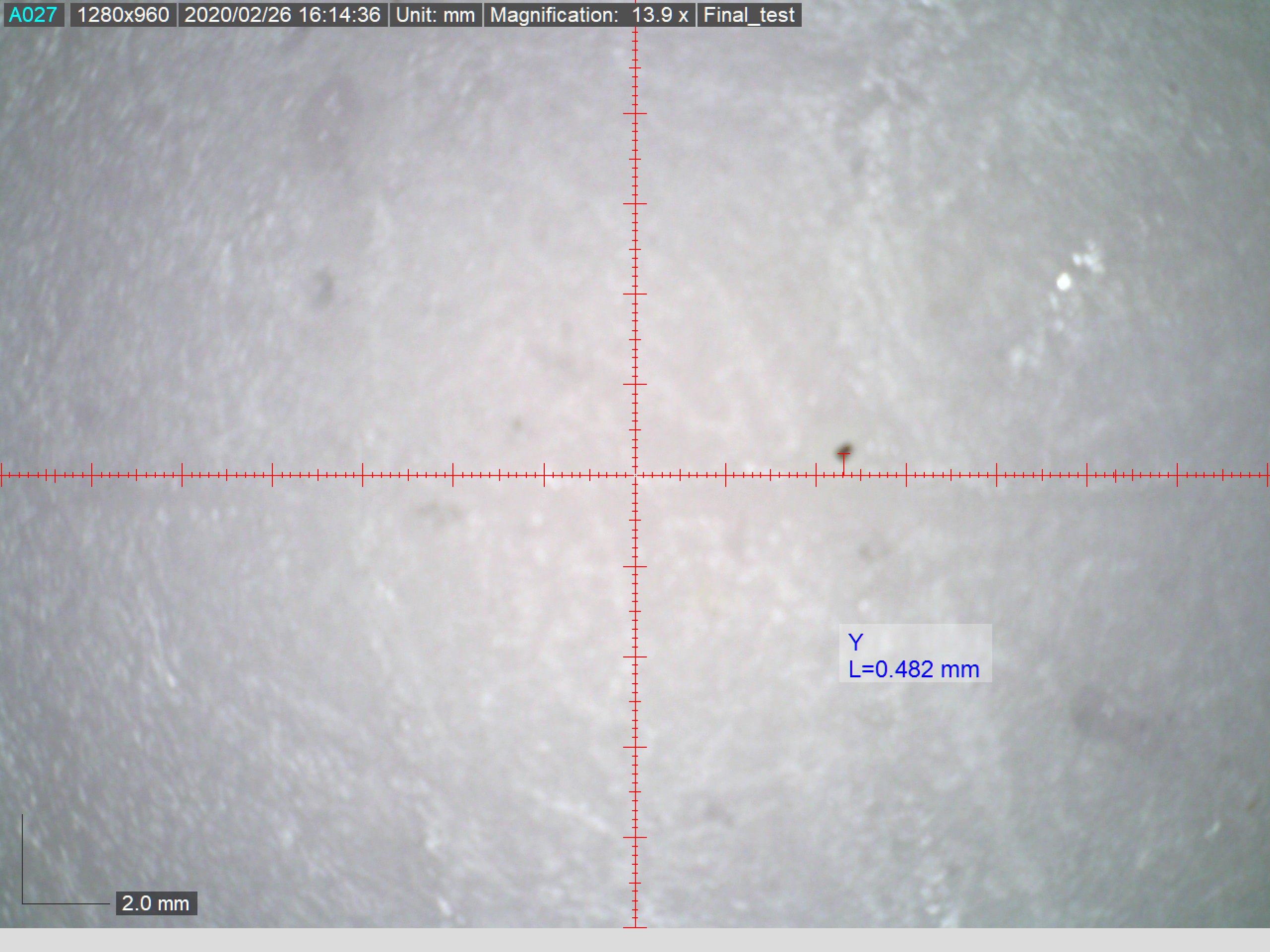

Supplement: S3 File — (ZIP) [file pone.0261089.s003.zip › Stiff phantom/fotos26.jpg]

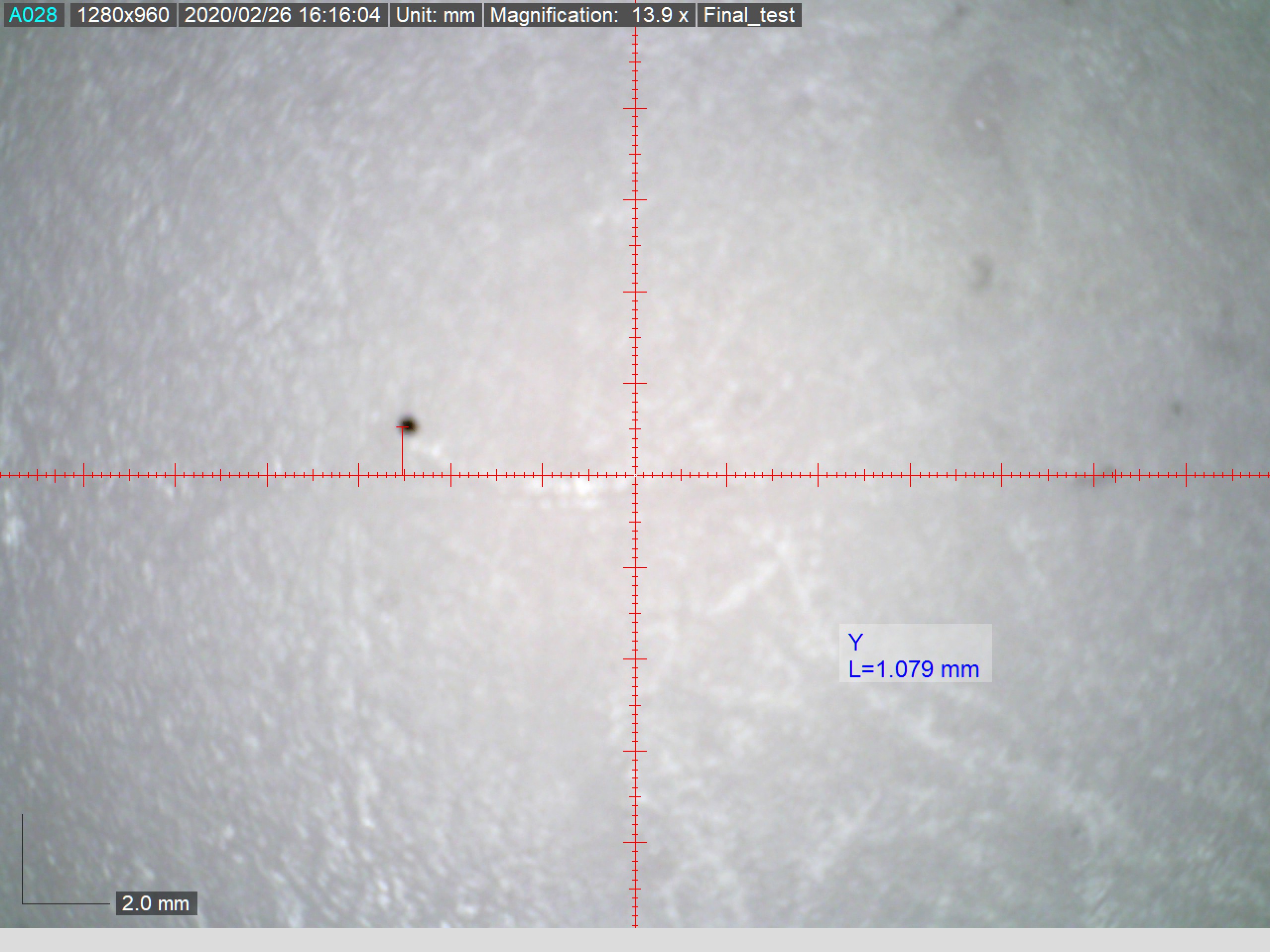

Supplement: S3 File — (ZIP) [file pone.0261089.s003.zip › Stiff phantom/fotos27.jpg]

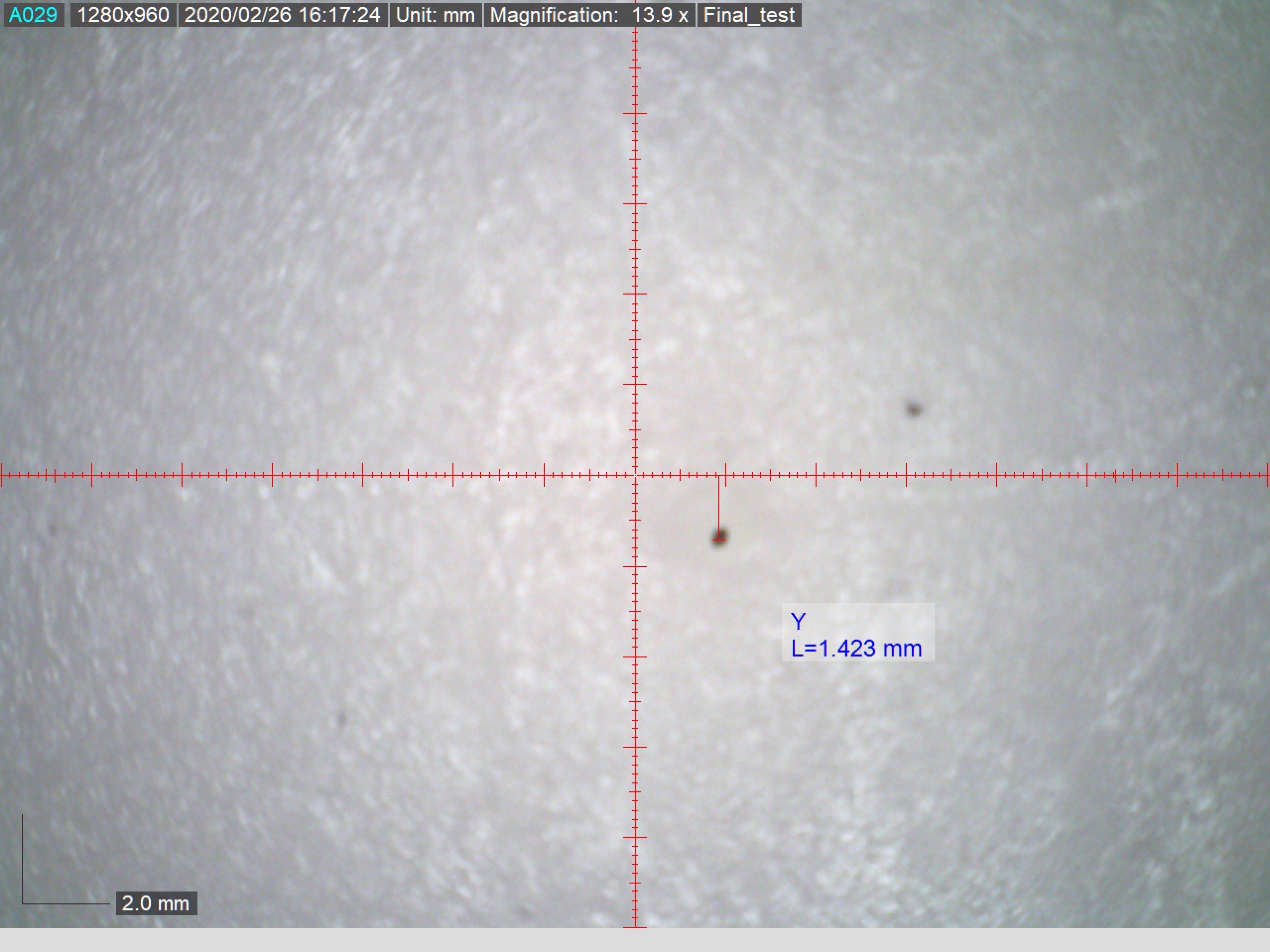

Supplement: S3 File — (ZIP) [file pone.0261089.s003.zip › Stiff phantom/fotos28.jpg]

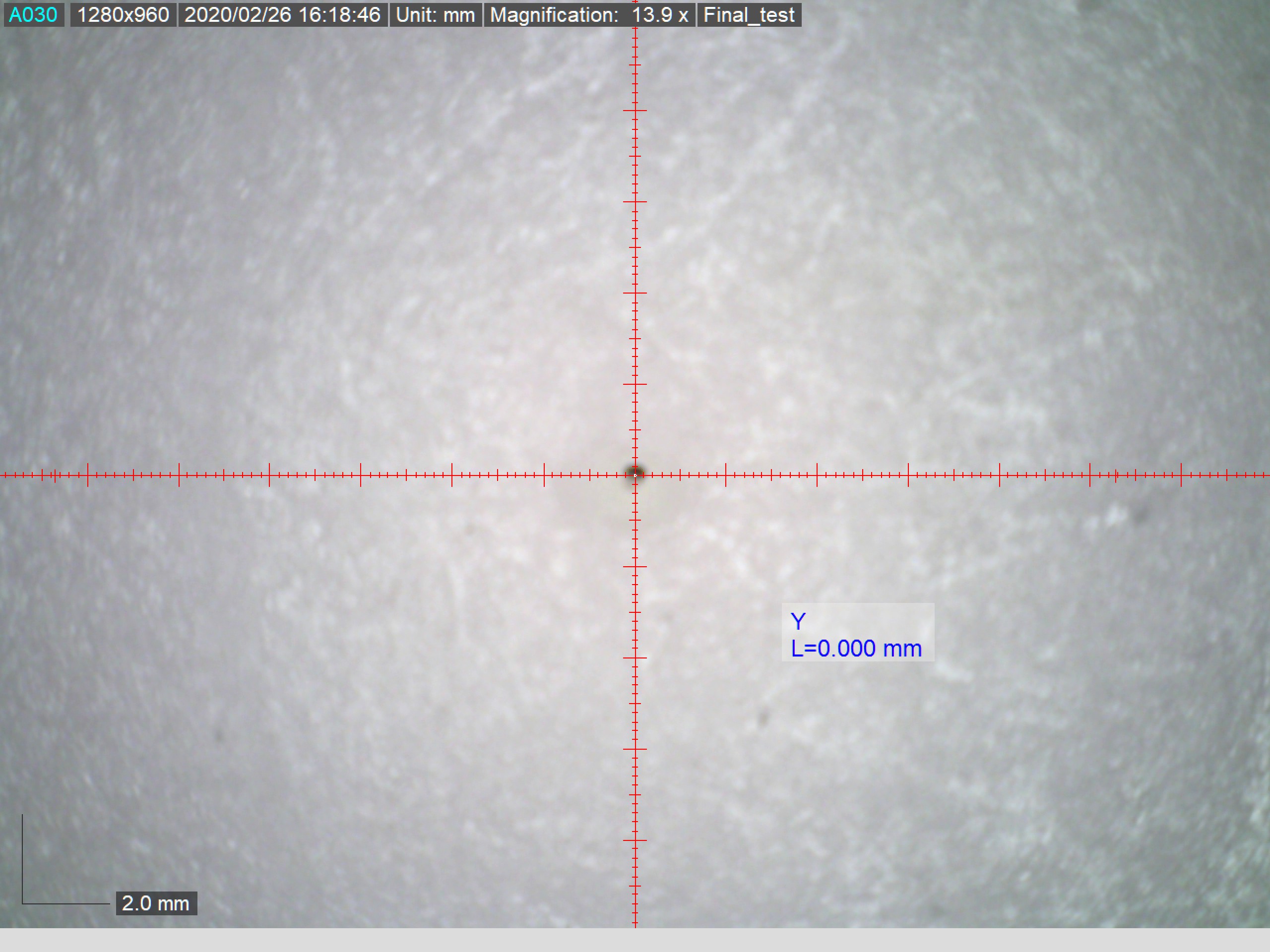

Supplement: S3 File — (ZIP) [file pone.0261089.s003.zip › Stiff phantom/fotos29.jpg]

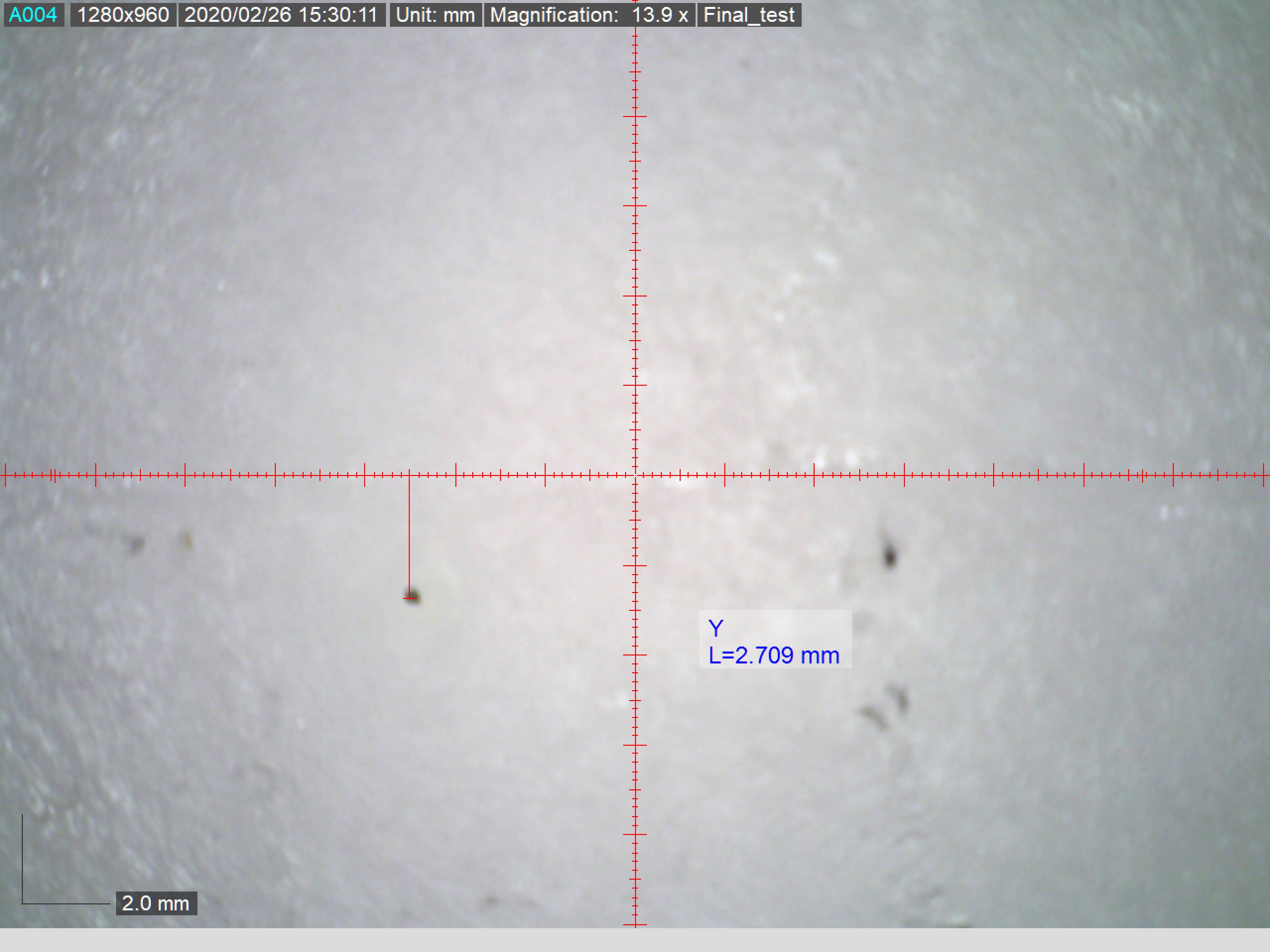

Supplement: S3 File — (ZIP) [file pone.0261089.s003.zip › Stiff phantom/fotos3.jpg]

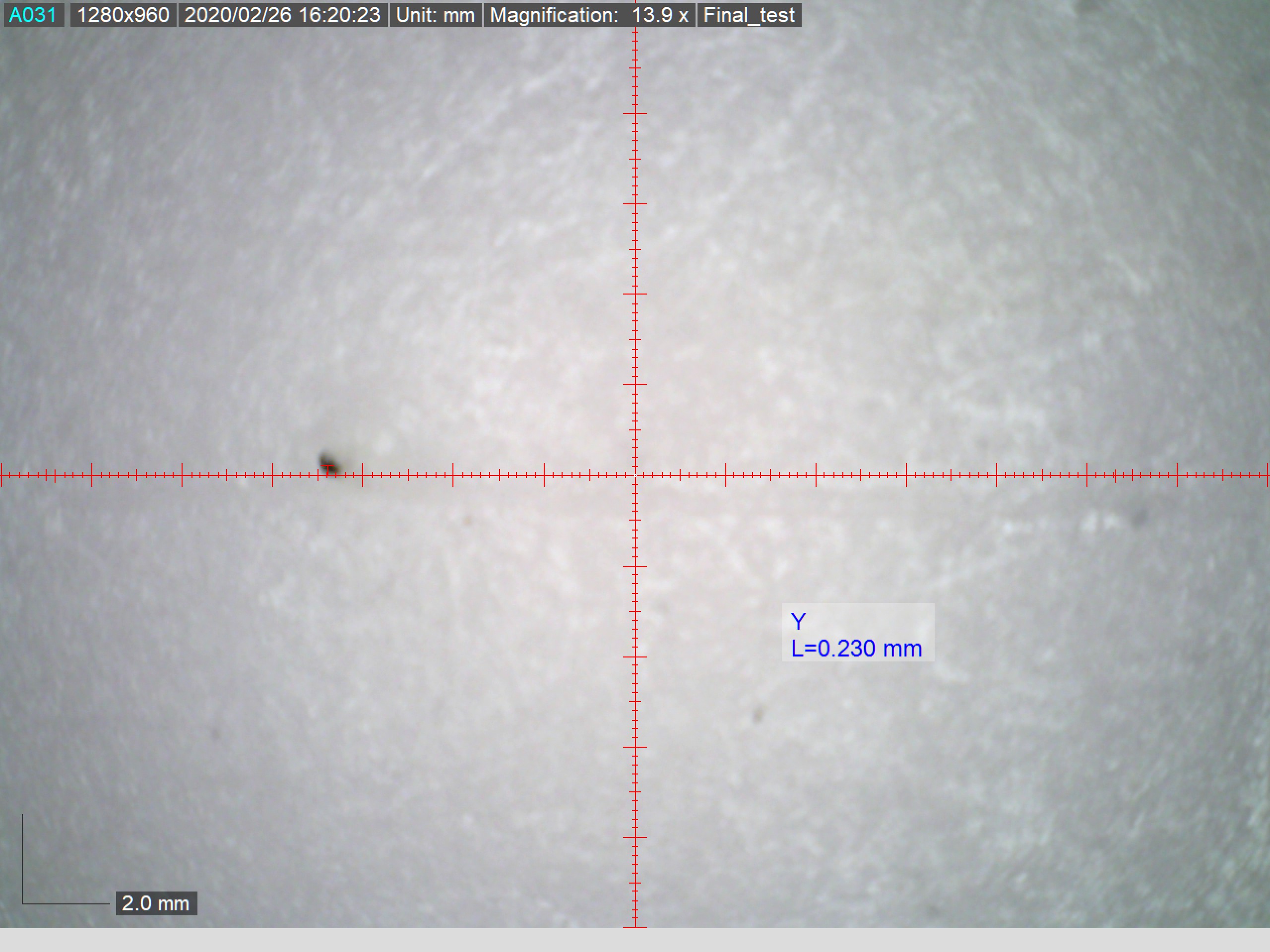

Supplement: S3 File — (ZIP) [file pone.0261089.s003.zip › Stiff phantom/fotos30.jpg]

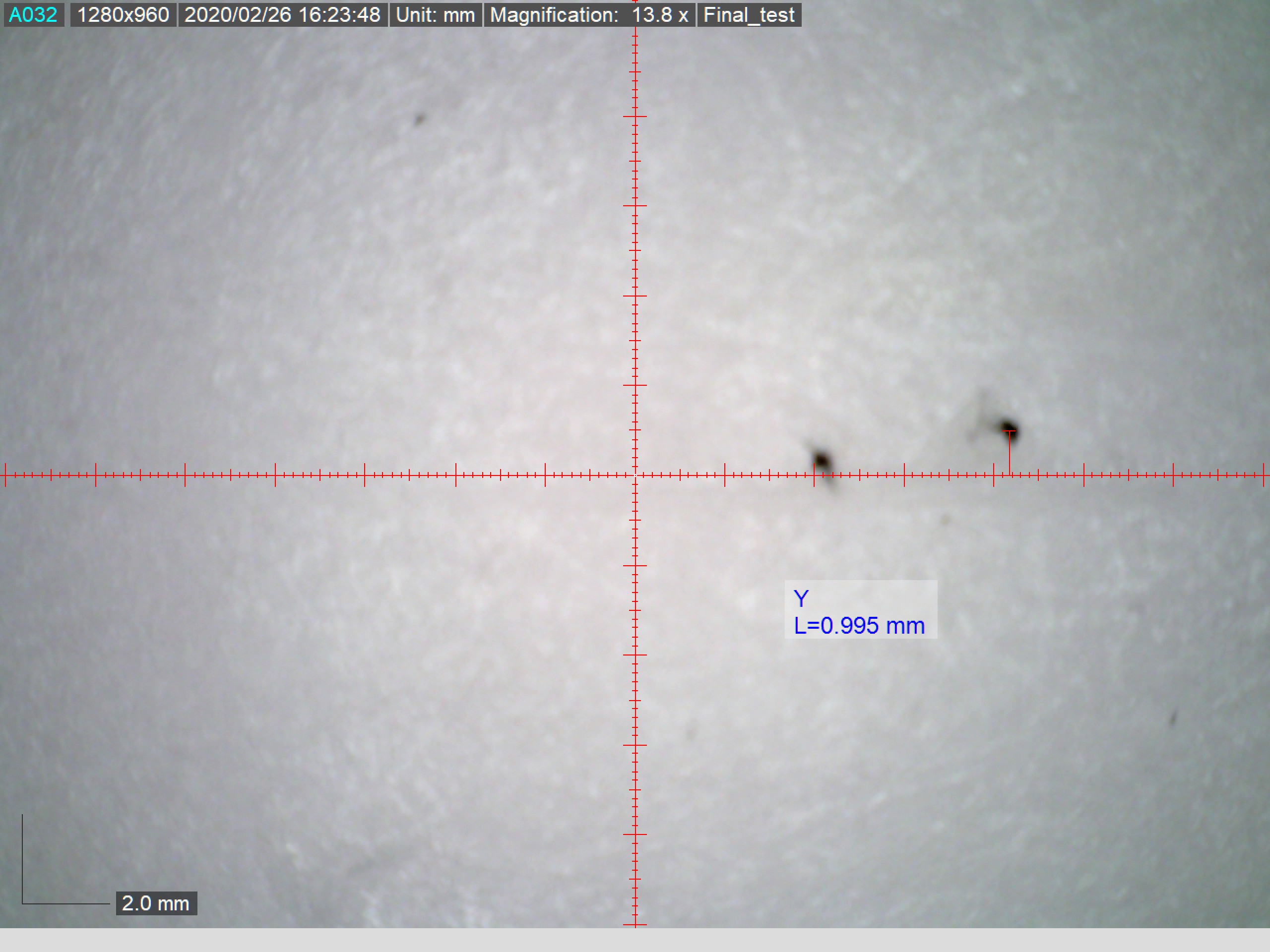

Supplement: S3 File — (ZIP) [file pone.0261089.s003.zip › Stiff phantom/fotos31.jpg]

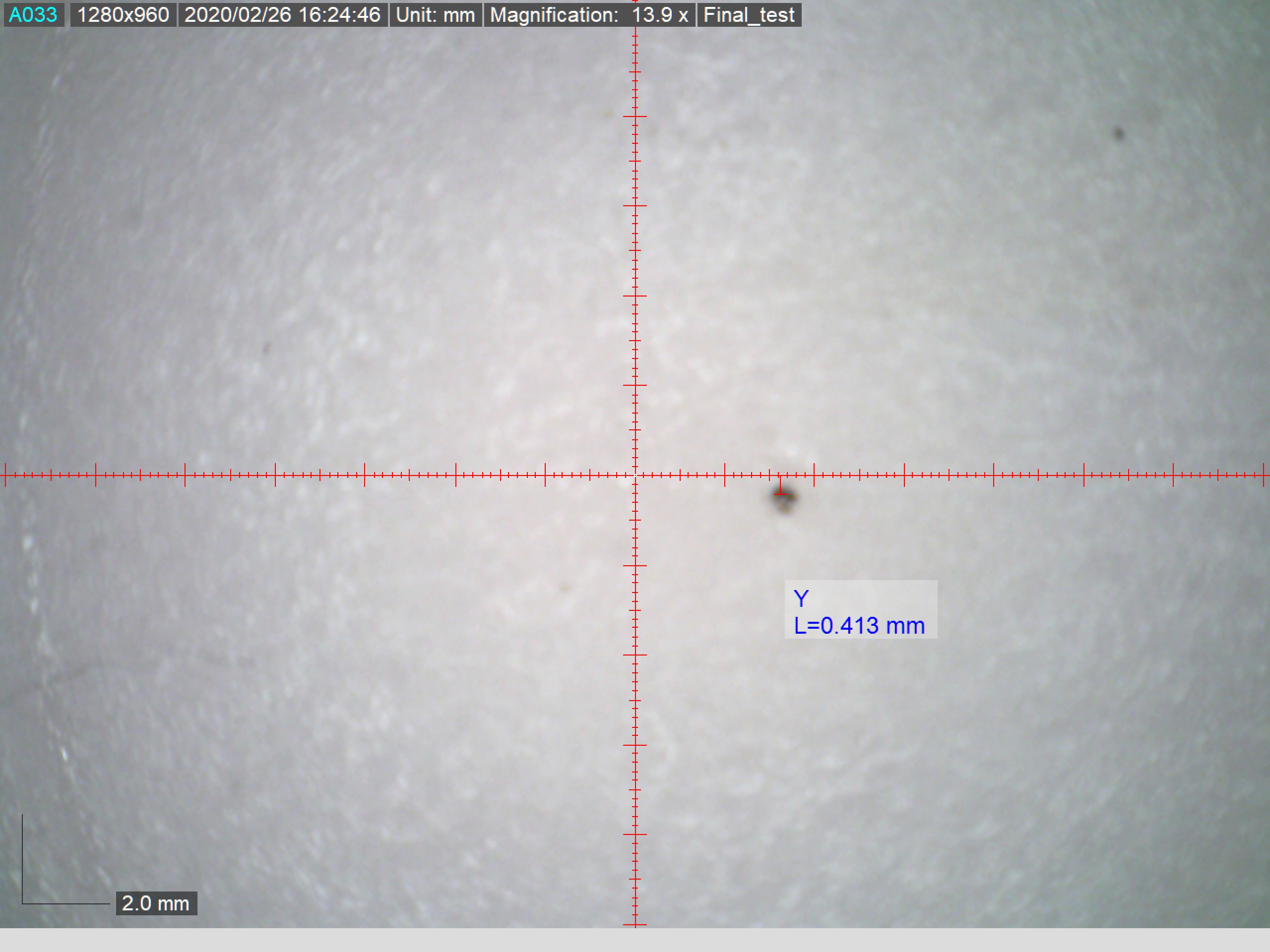

Supplement: S3 File — (ZIP) [file pone.0261089.s003.zip › Stiff phantom/fotos32.jpg]

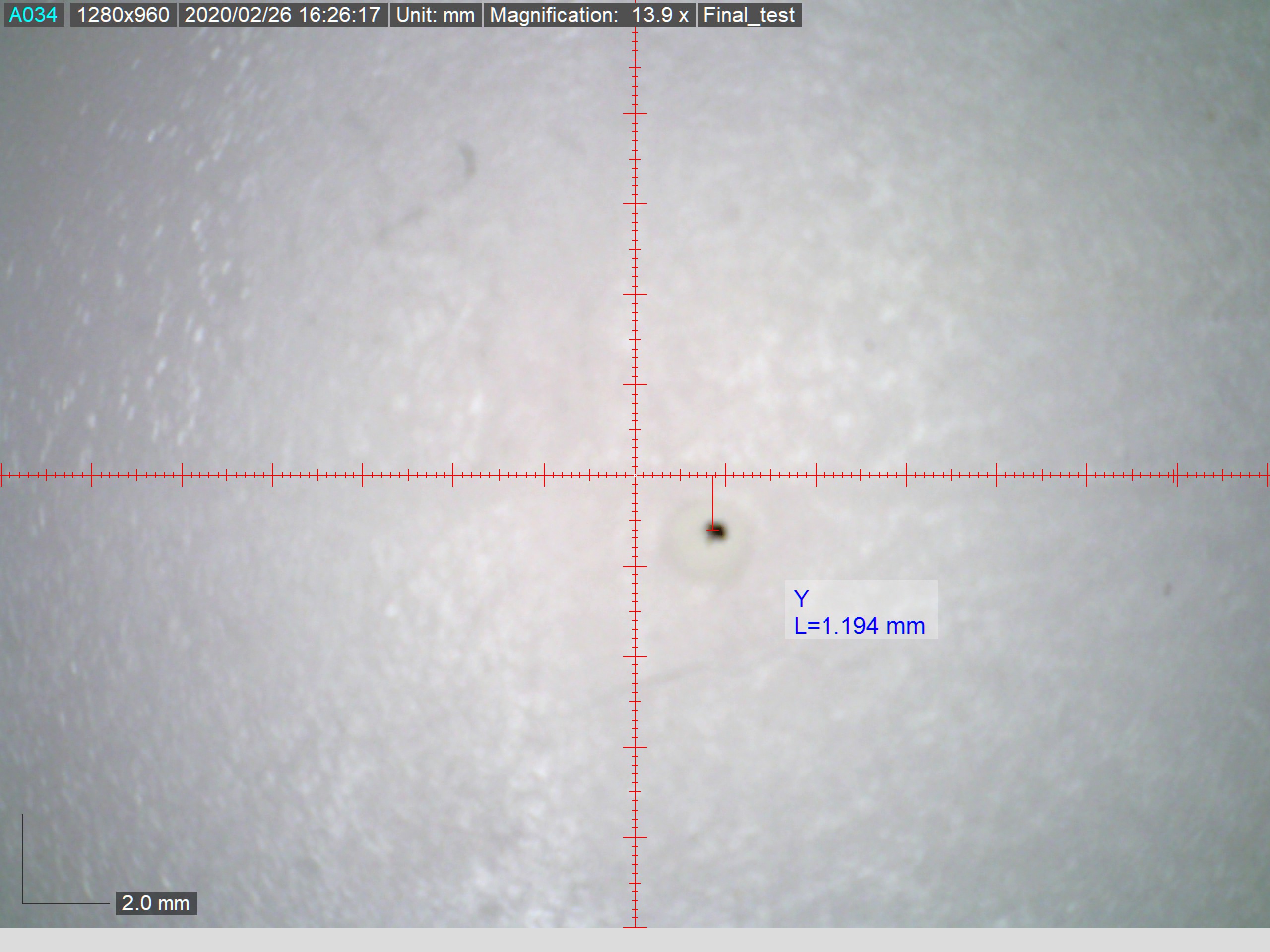

Supplement: S3 File — (ZIP) [file pone.0261089.s003.zip › Stiff phantom/fotos33.jpg]

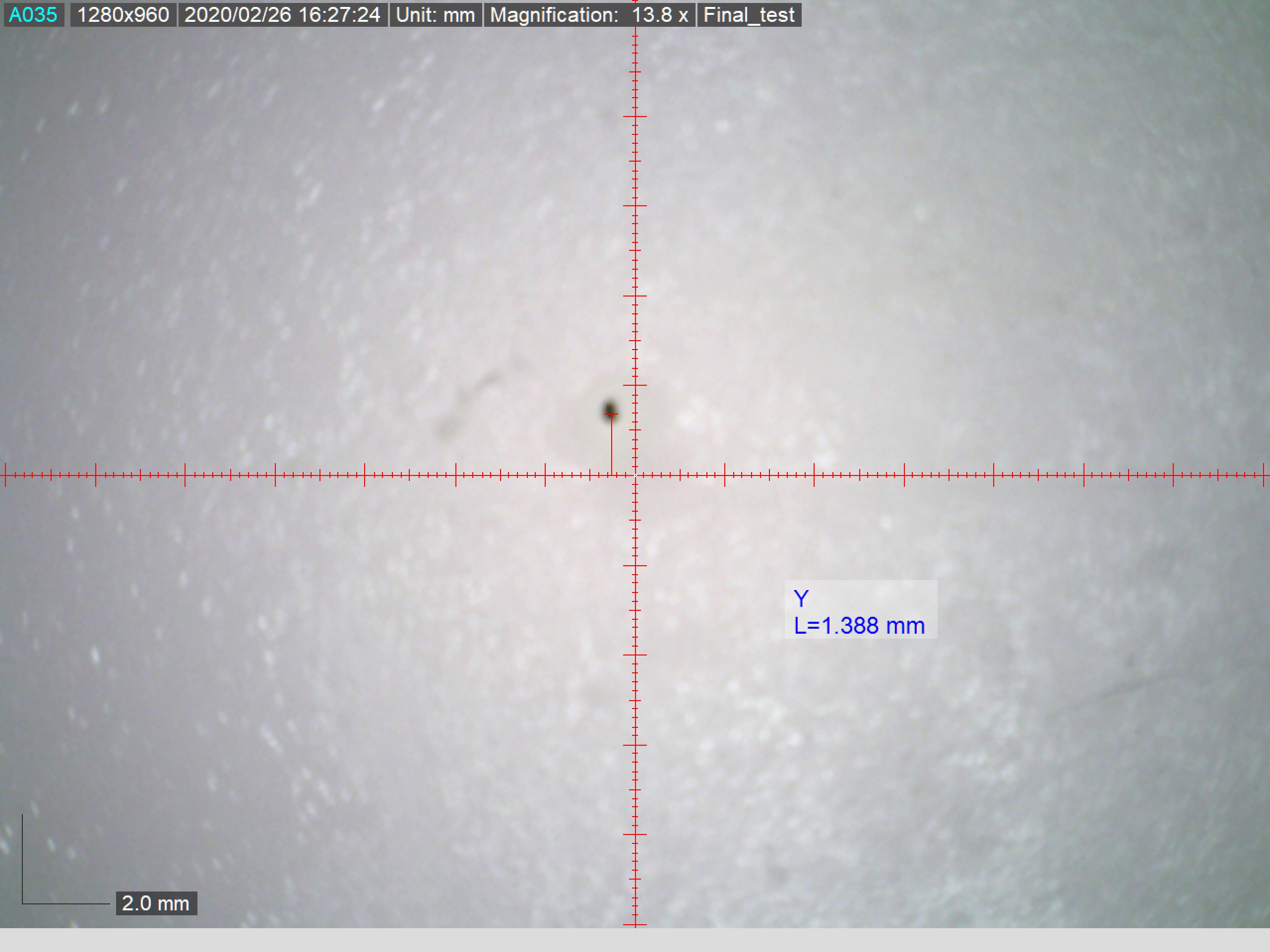

Supplement: S3 File — (ZIP) [file pone.0261089.s003.zip › Stiff phantom/fotos34.jpg]

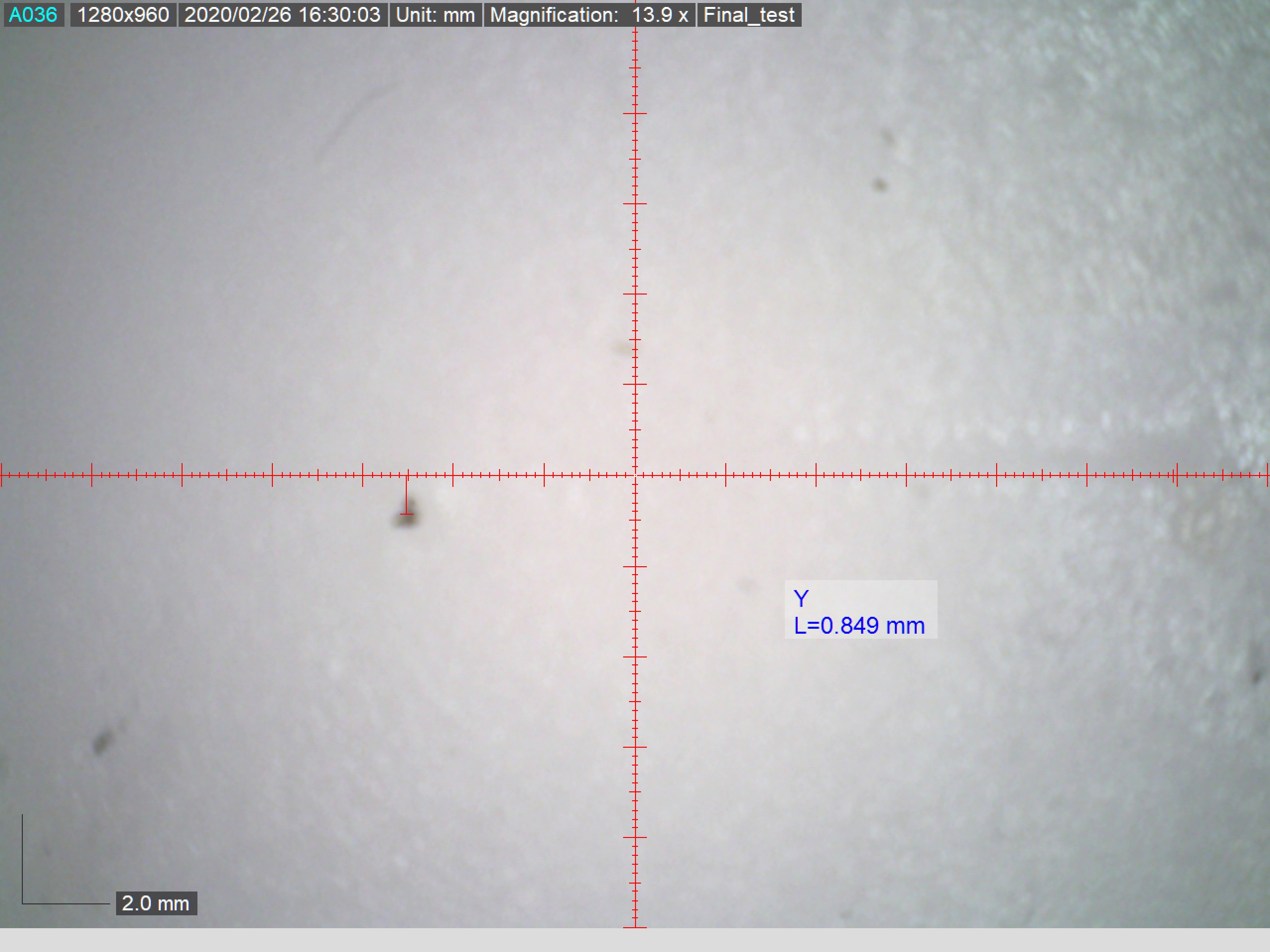

Supplement: S3 File — (ZIP) [file pone.0261089.s003.zip › Stiff phantom/fotos35.jpg]

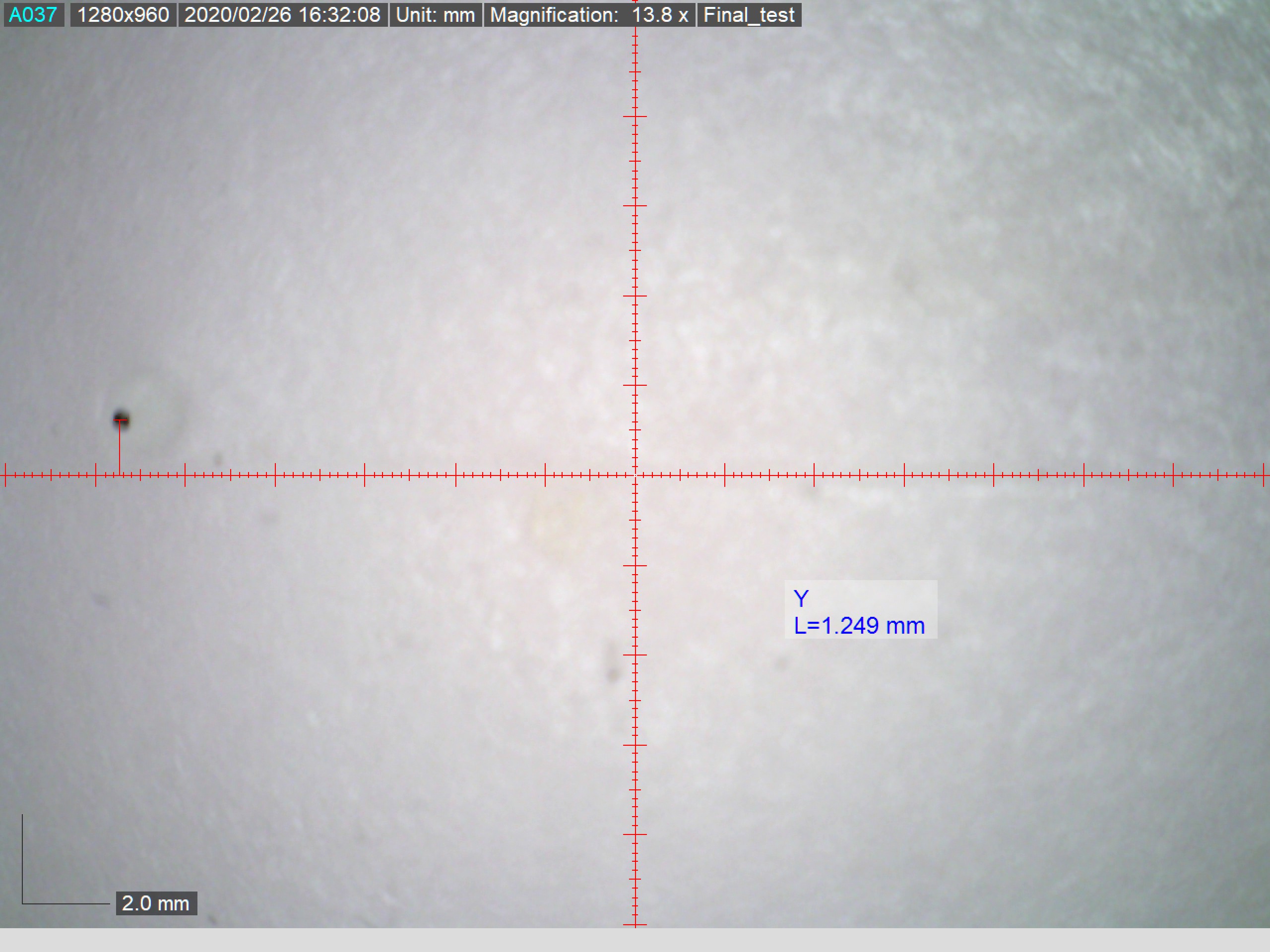

Supplement: S3 File — (ZIP) [file pone.0261089.s003.zip › Stiff phantom/fotos36.jpg]

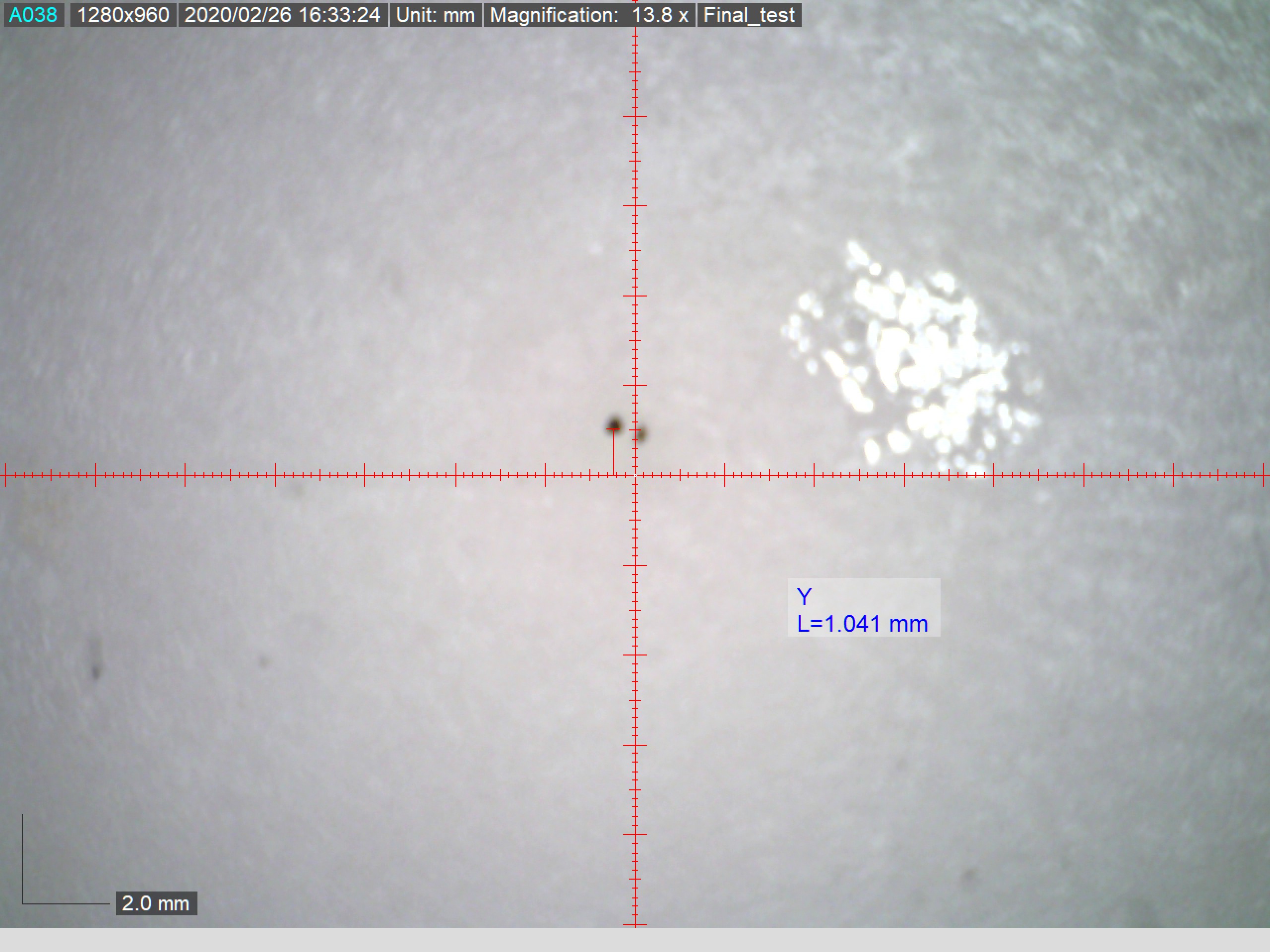

Supplement: S3 File — (ZIP) [file pone.0261089.s003.zip › Stiff phantom/fotos37.jpg]

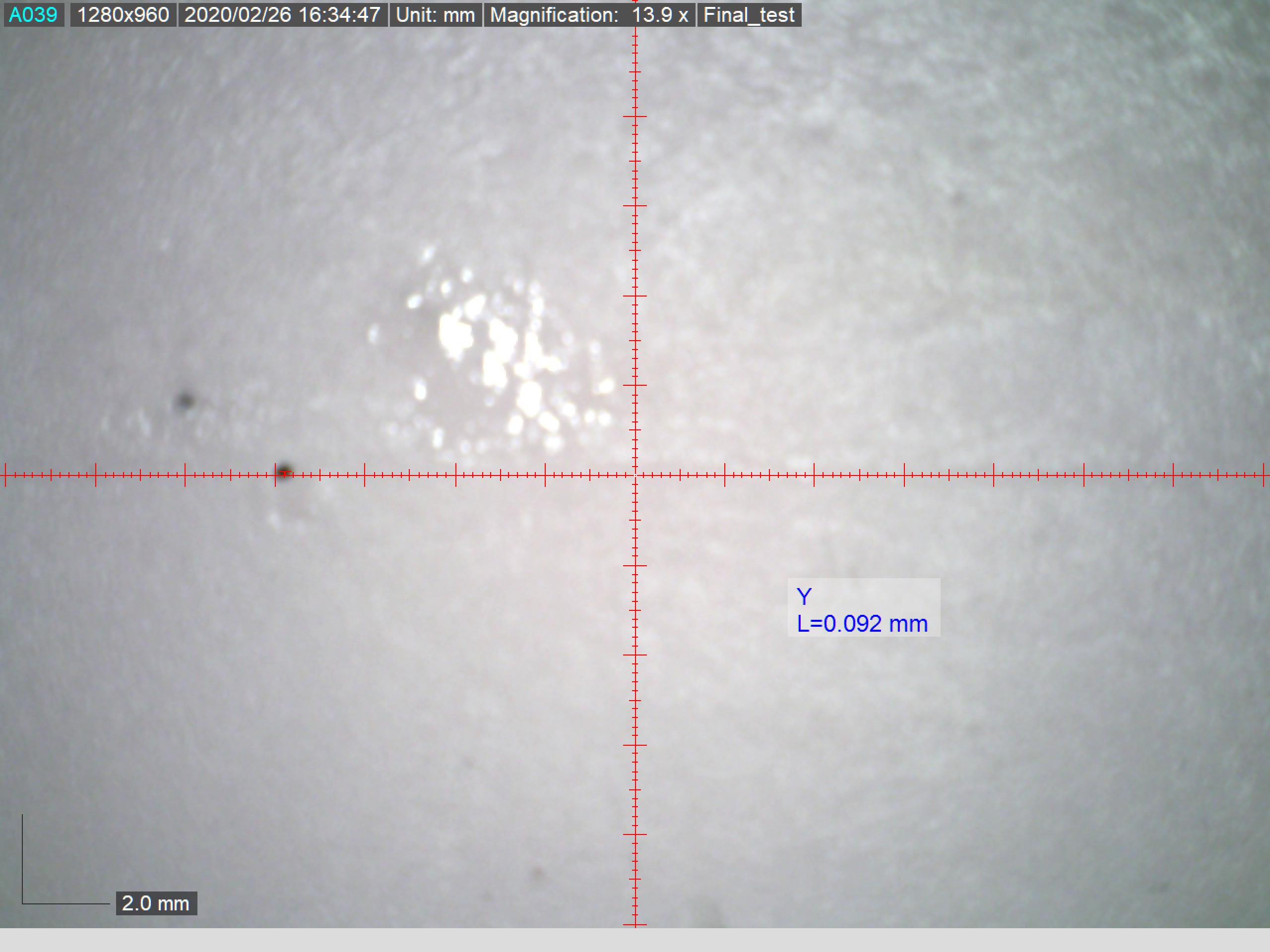

Supplement: S3 File — (ZIP) [file pone.0261089.s003.zip › Stiff phantom/fotos38.jpg]

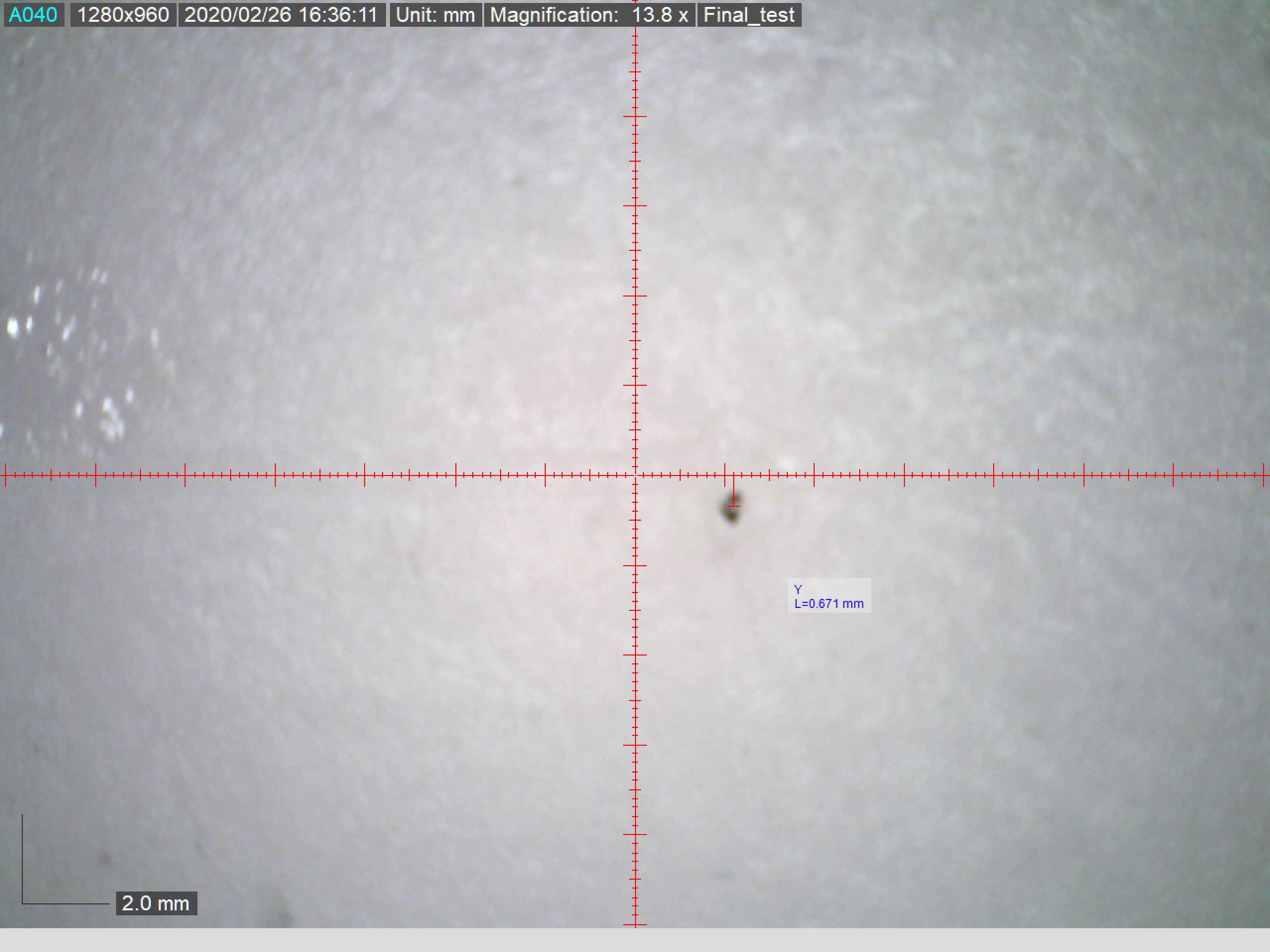

Supplement: S3 File — (ZIP) [file pone.0261089.s003.zip › Stiff phantom/fotos39.jpg]

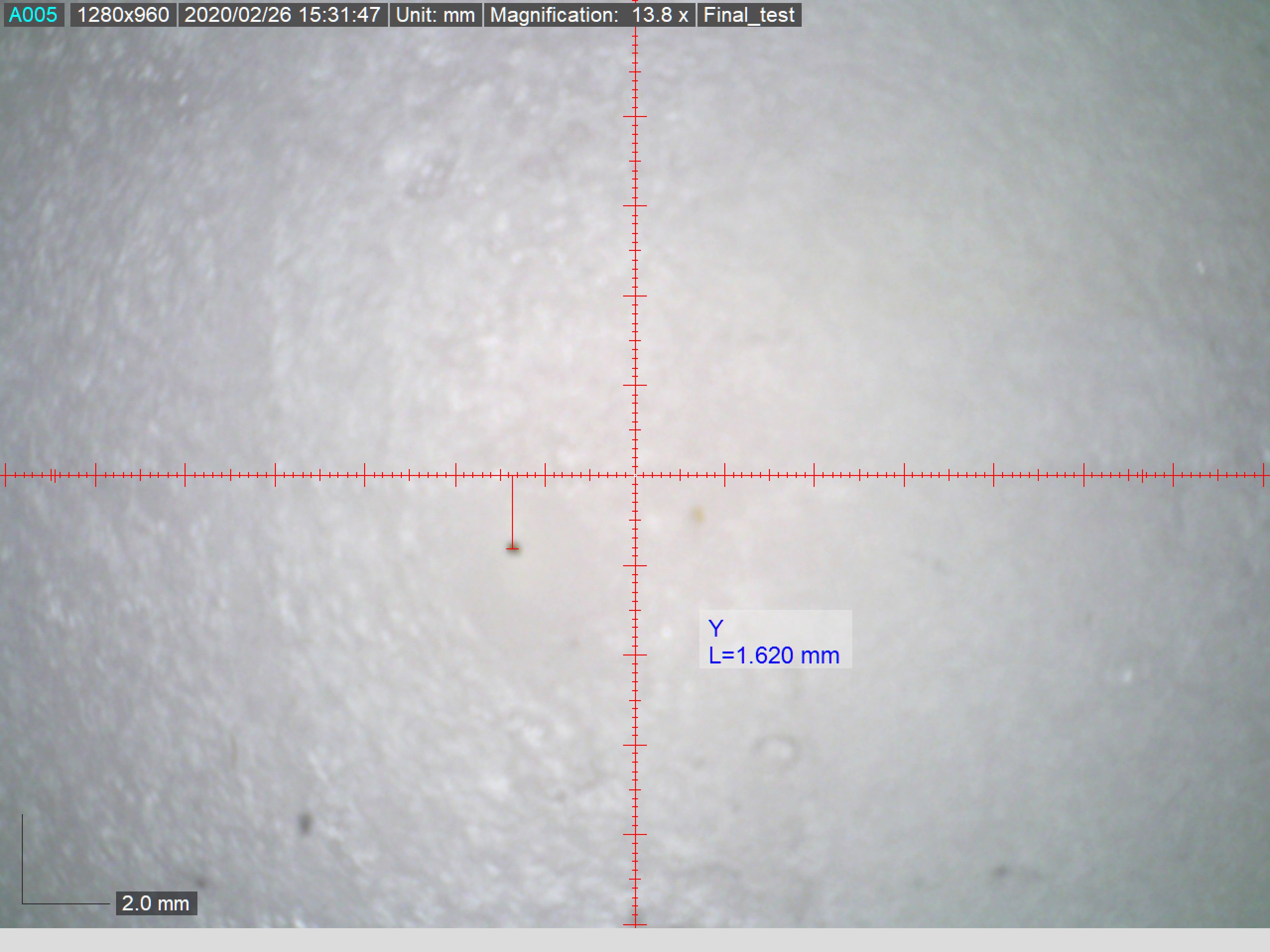

Supplement: S3 File — (ZIP) [file pone.0261089.s003.zip › Stiff phantom/fotos4.jpg]

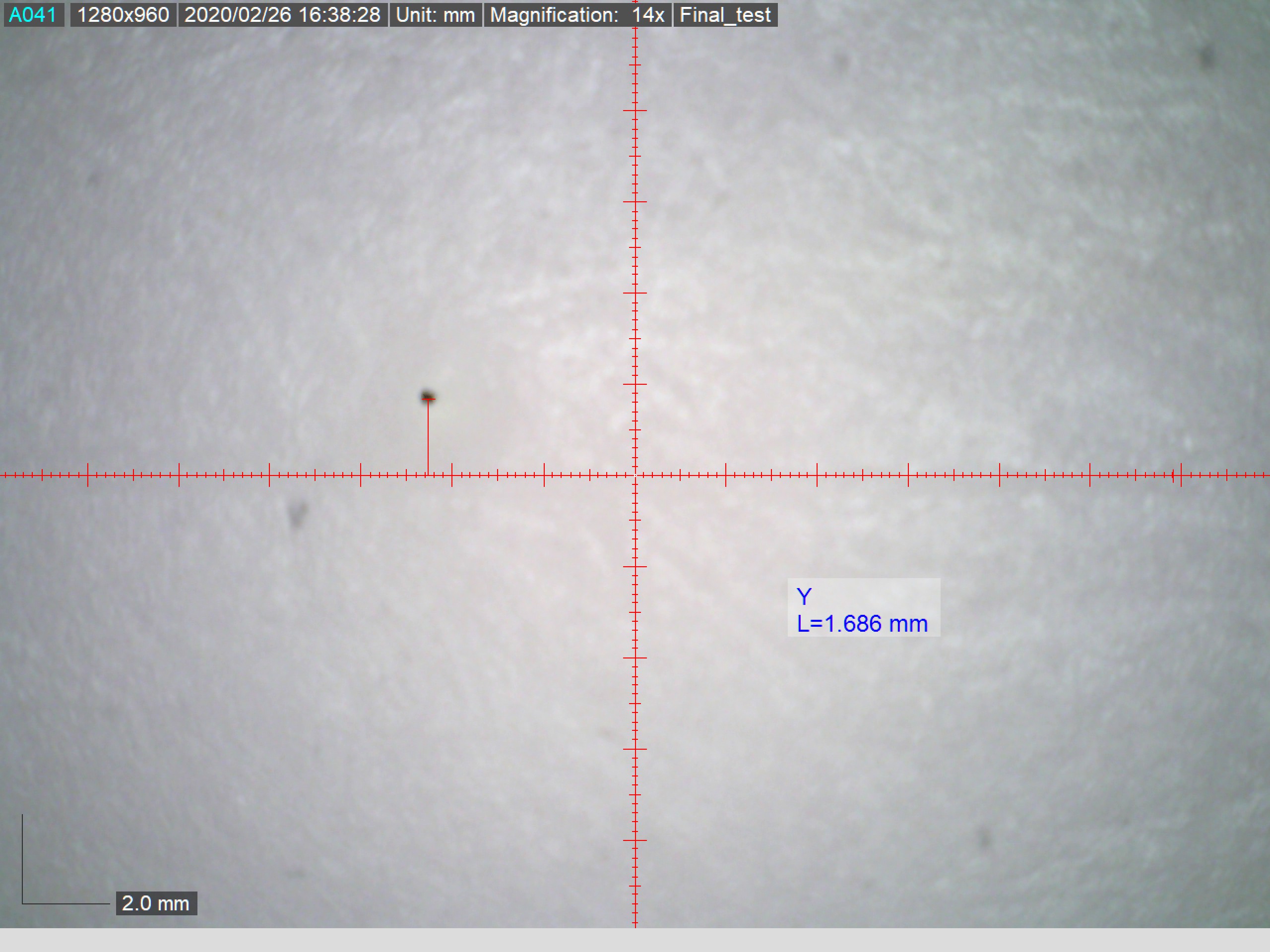

Supplement: S3 File — (ZIP) [file pone.0261089.s003.zip › Stiff phantom/fotos40.jpg]

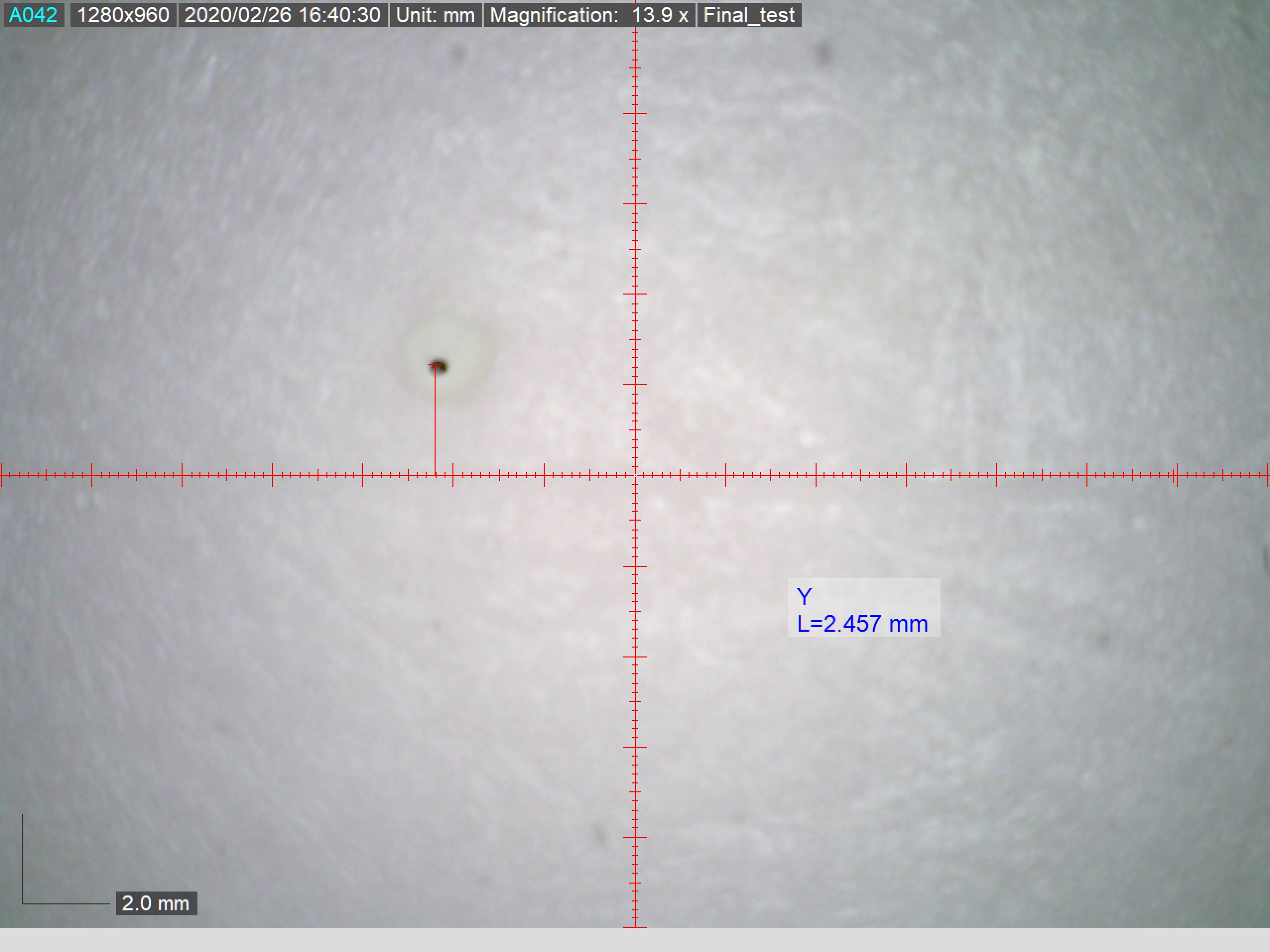

Supplement: S3 File — (ZIP) [file pone.0261089.s003.zip › Stiff phantom/fotos41.jpg]

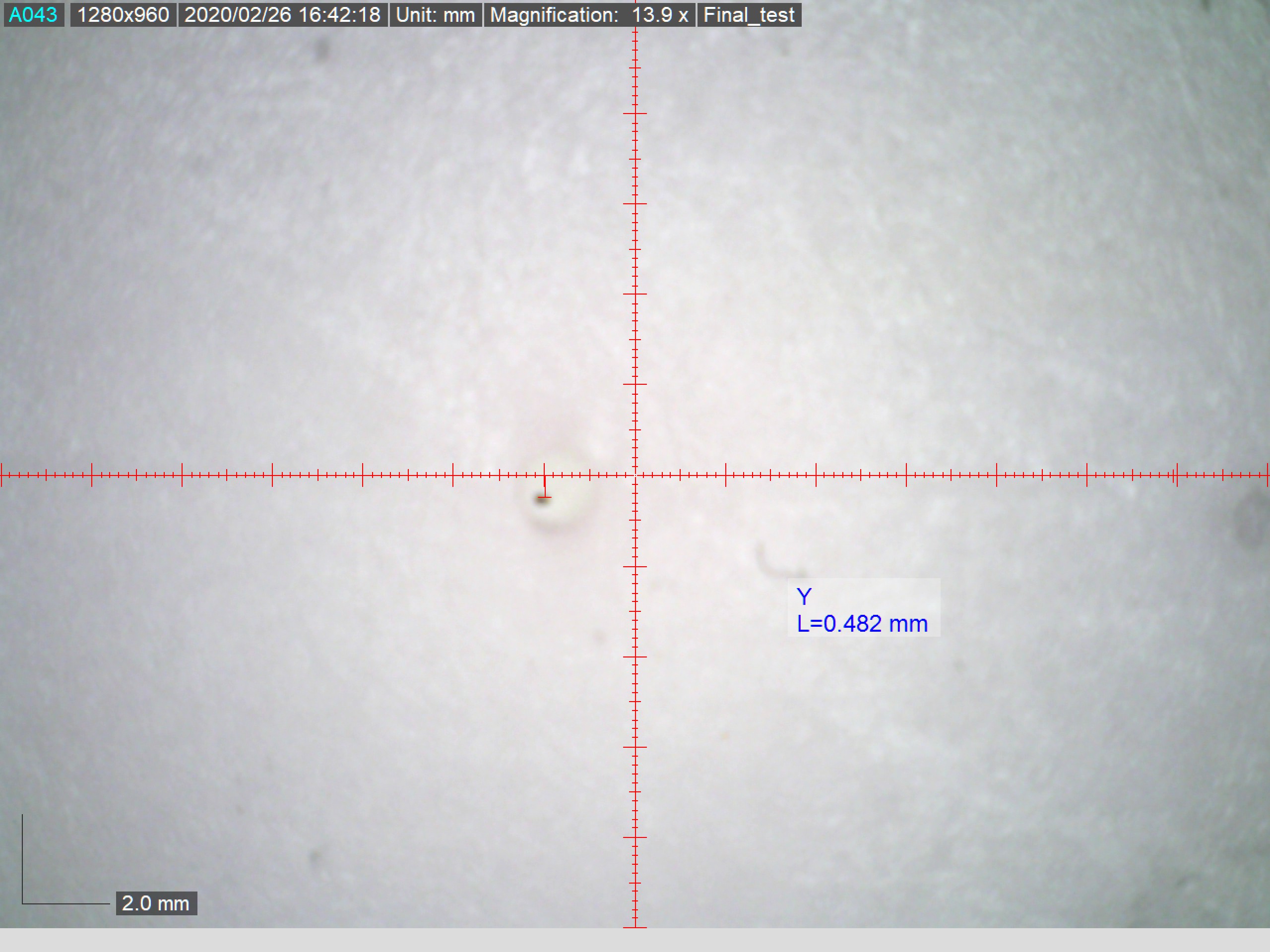

Supplement: S3 File — (ZIP) [file pone.0261089.s003.zip › Stiff phantom/fotos42.jpg]

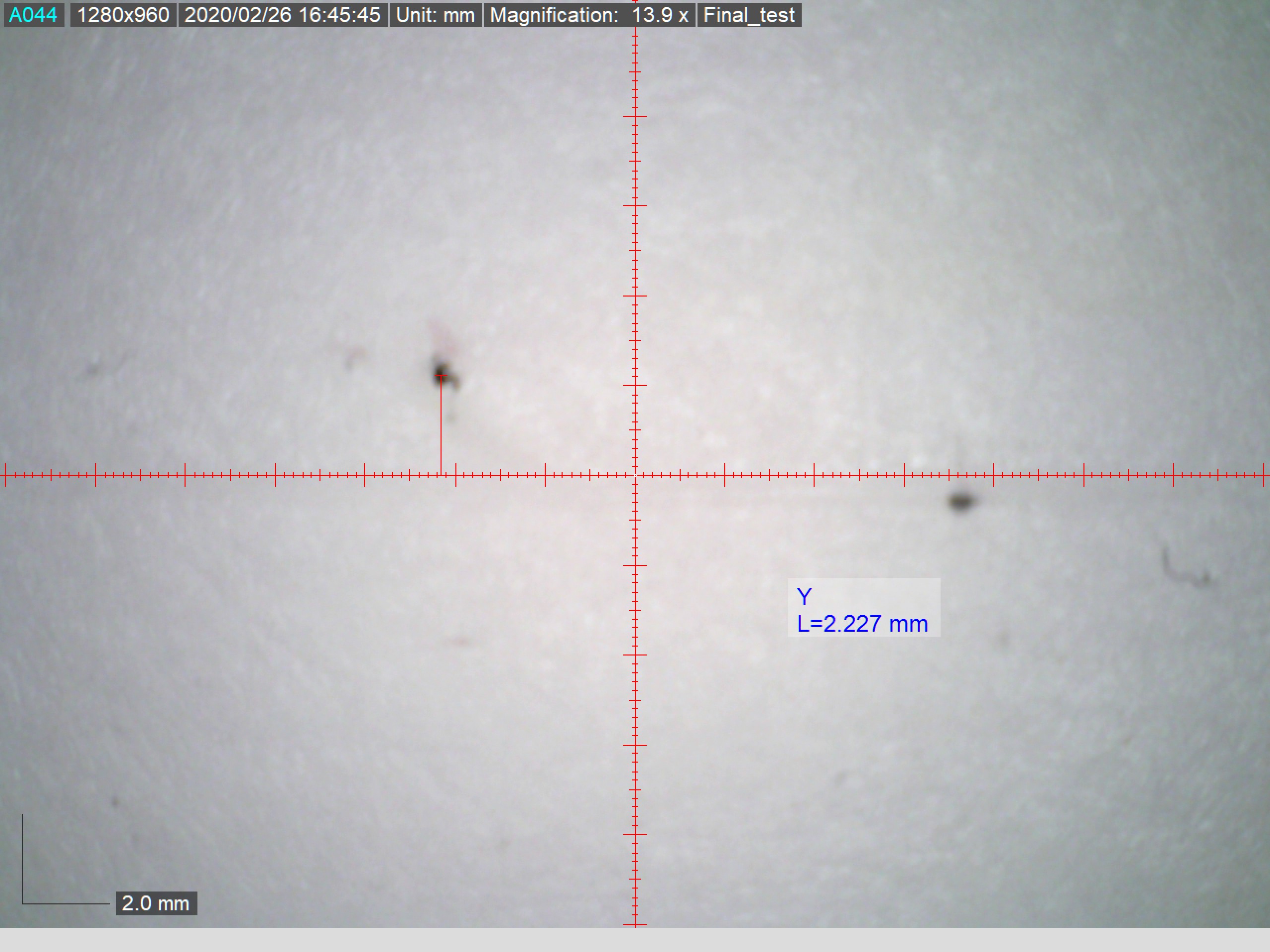

Supplement: S3 File — (ZIP) [file pone.0261089.s003.zip › Stiff phantom/fotos43.jpg]

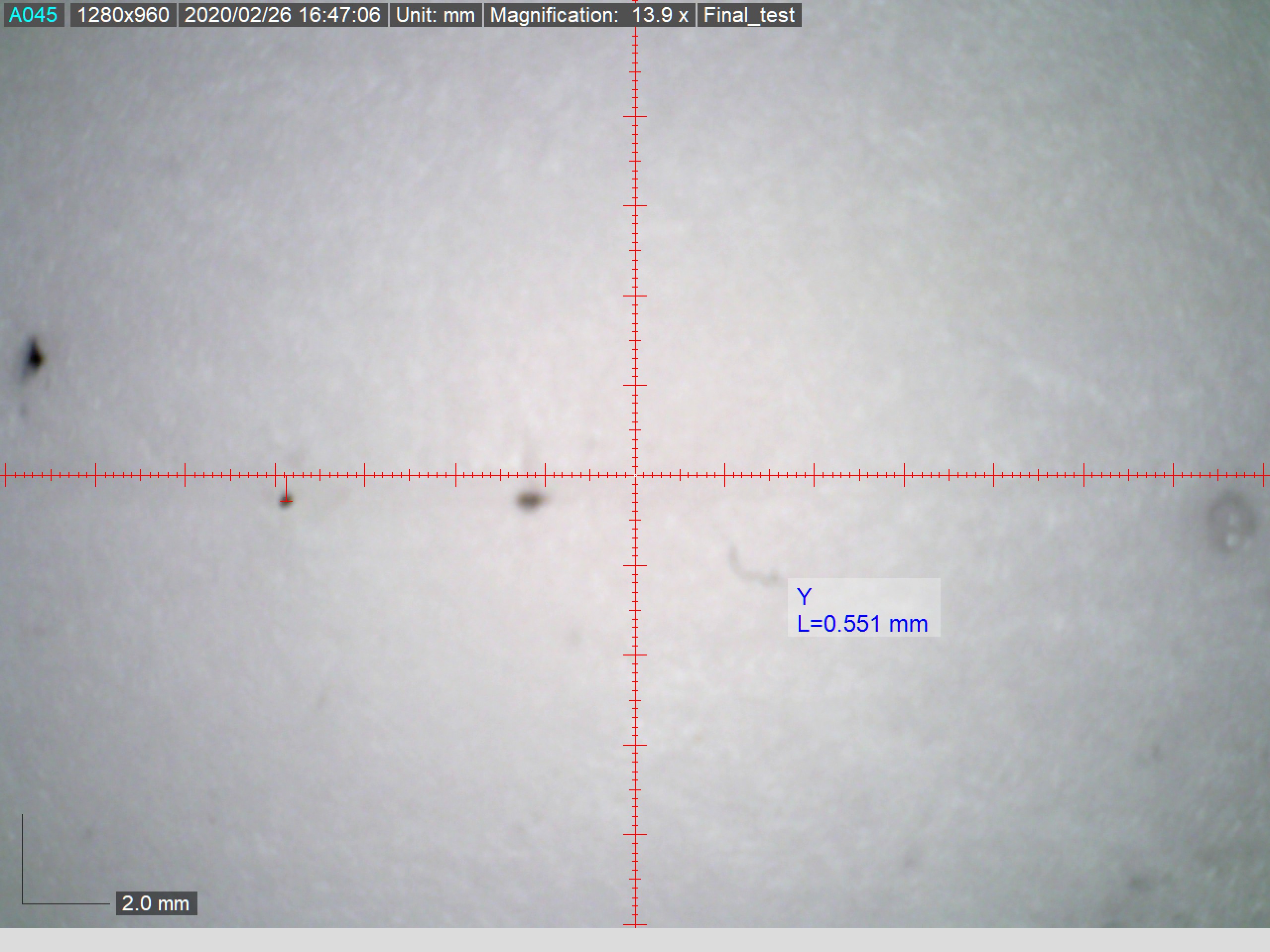

Supplement: S3 File — (ZIP) [file pone.0261089.s003.zip › Stiff phantom/fotos44.jpg]

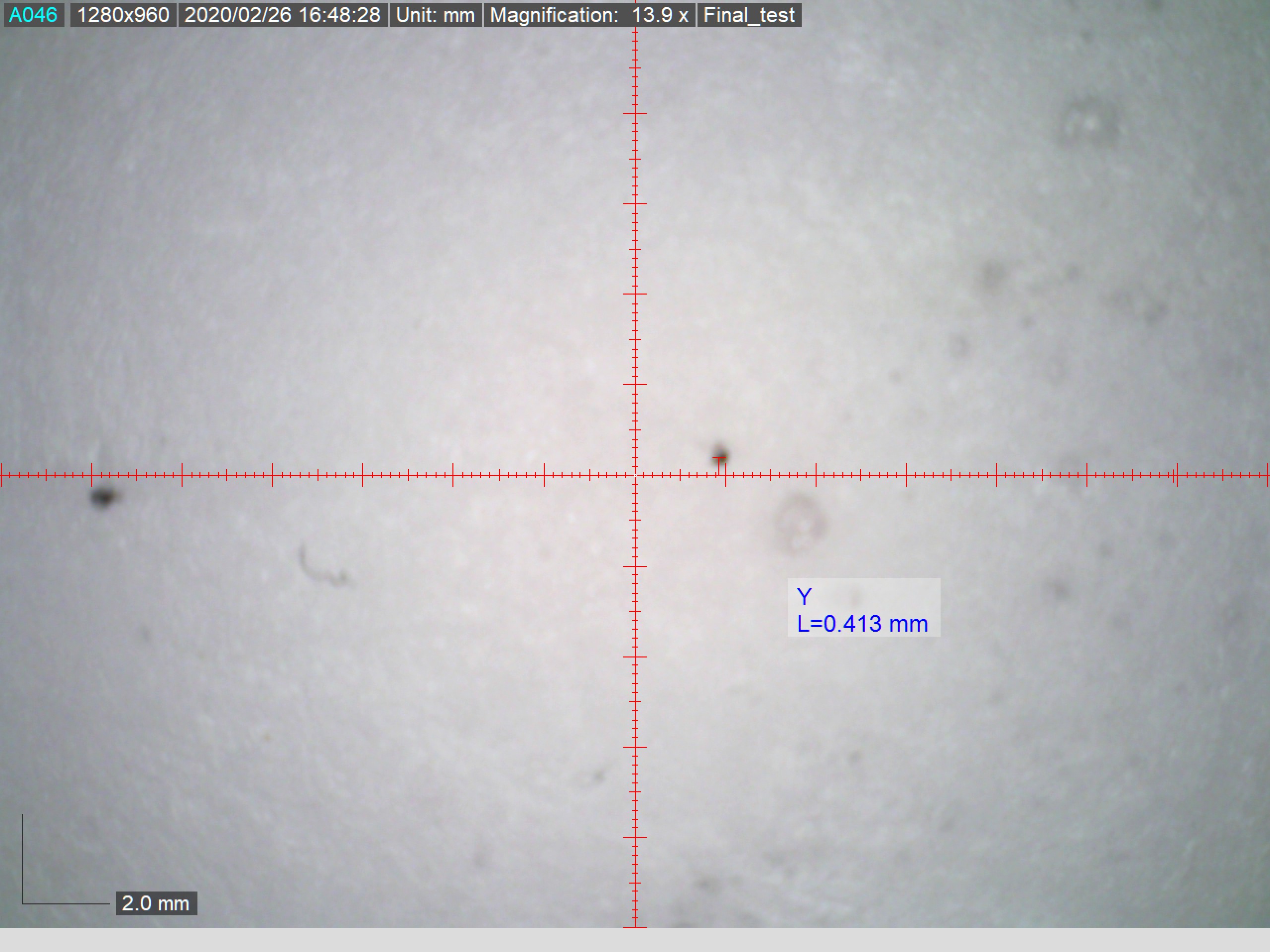

Supplement: S3 File — (ZIP) [file pone.0261089.s003.zip › Stiff phantom/fotos45.jpg]

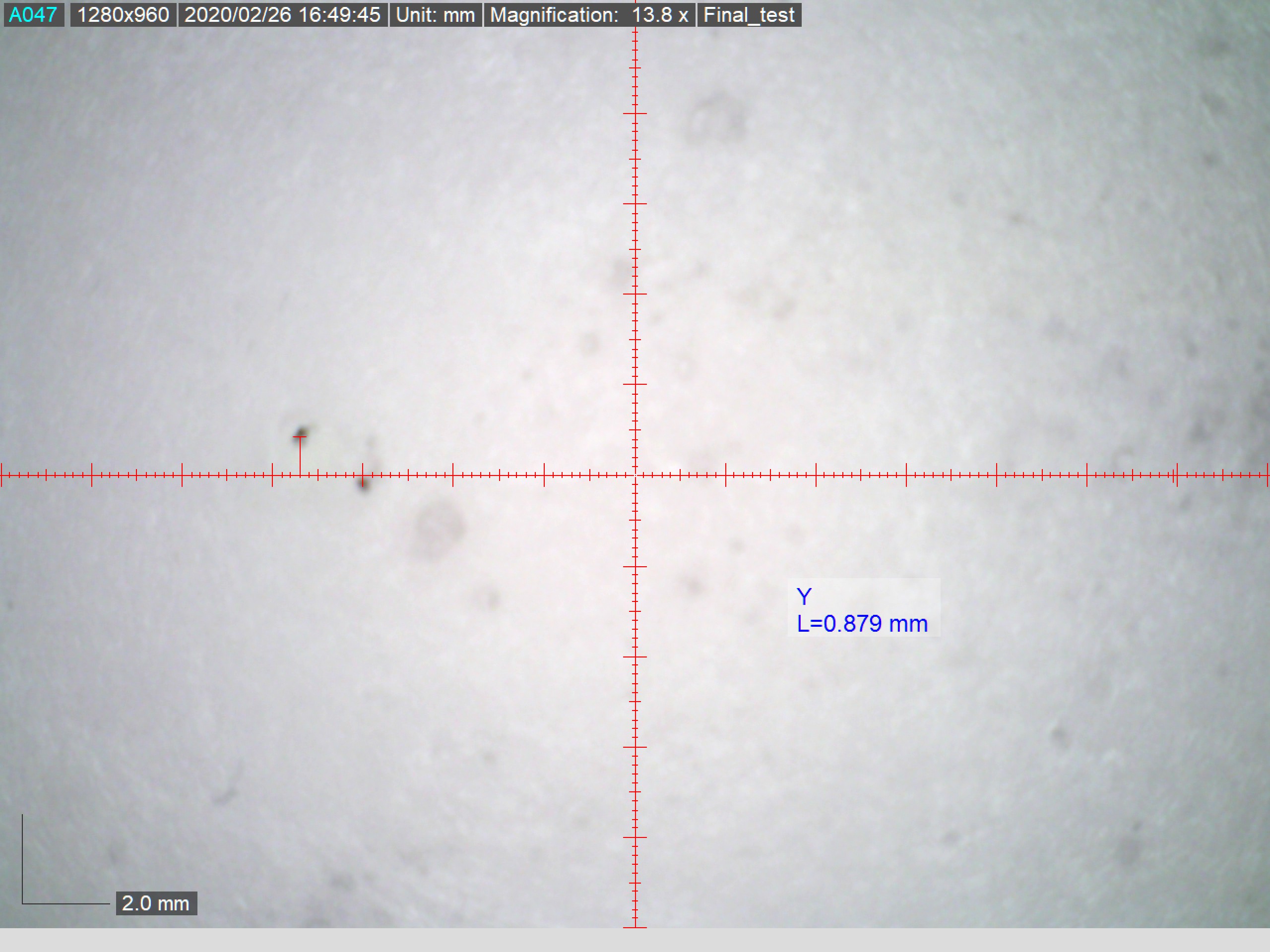

Supplement: S3 File — (ZIP) [file pone.0261089.s003.zip › Stiff phantom/fotos46.jpg]

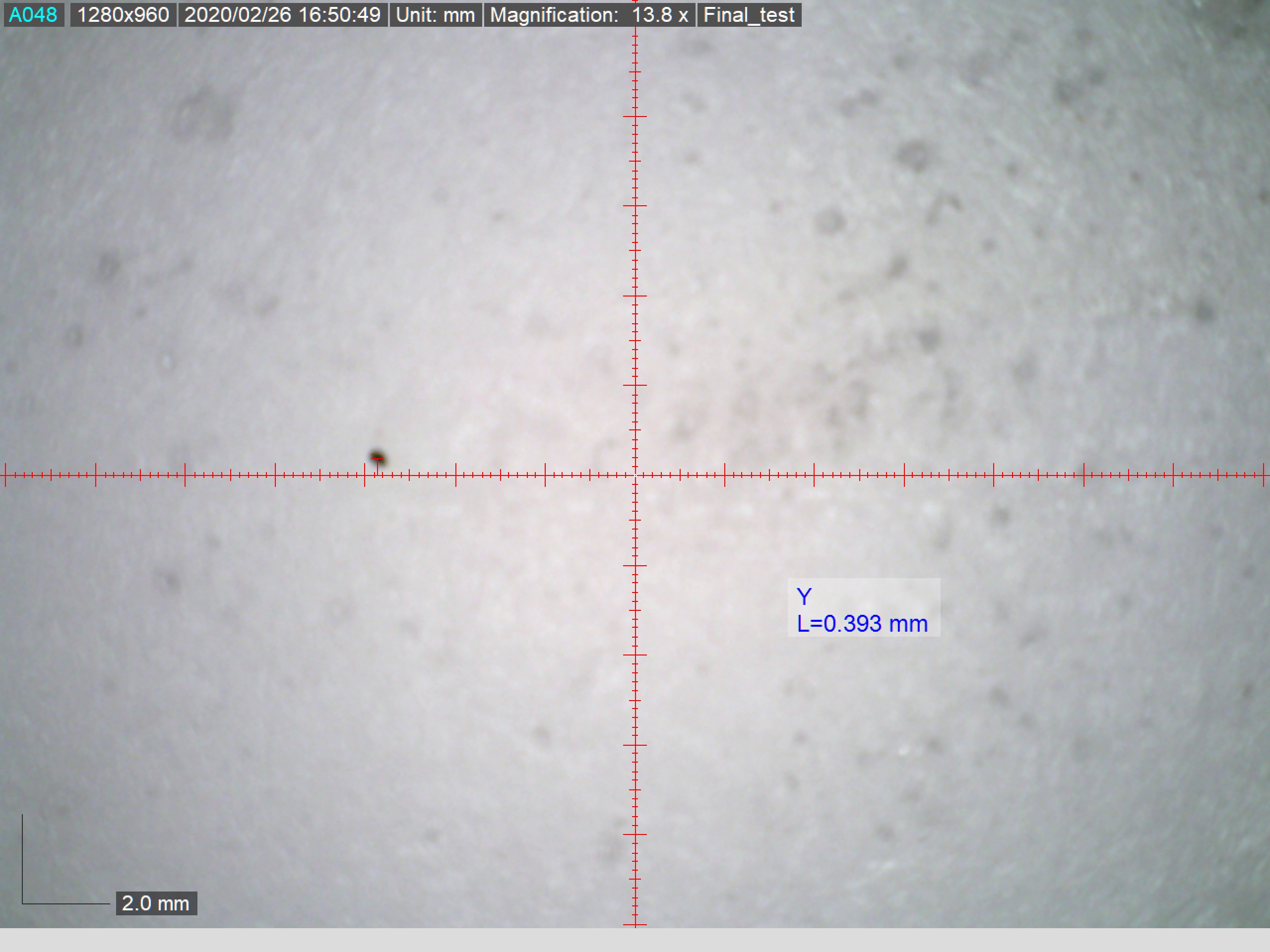

Supplement: S3 File — (ZIP) [file pone.0261089.s003.zip › Stiff phantom/fotos47.jpg]

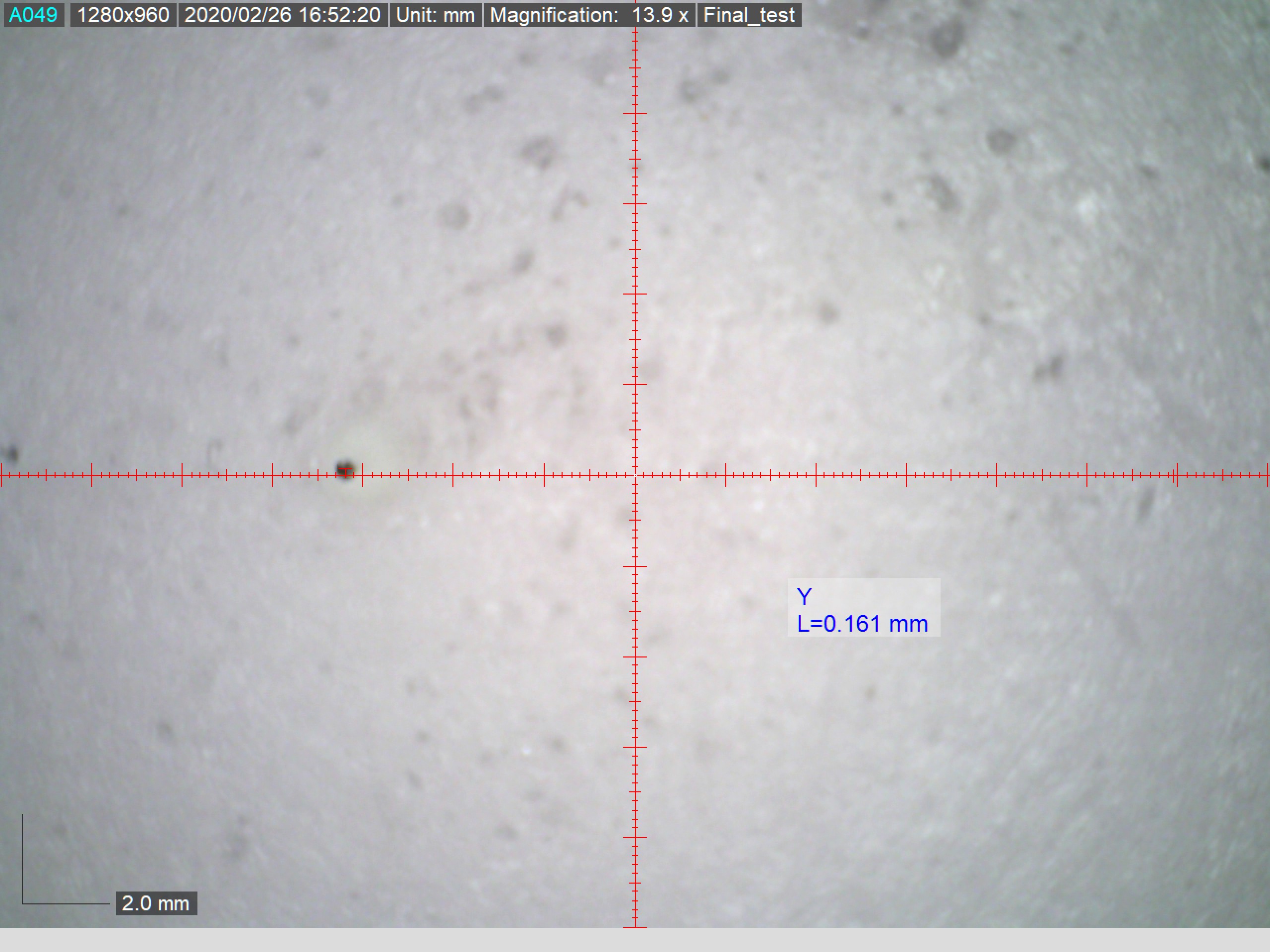

Supplement: S3 File — (ZIP) [file pone.0261089.s003.zip › Stiff phantom/fotos48.jpg]

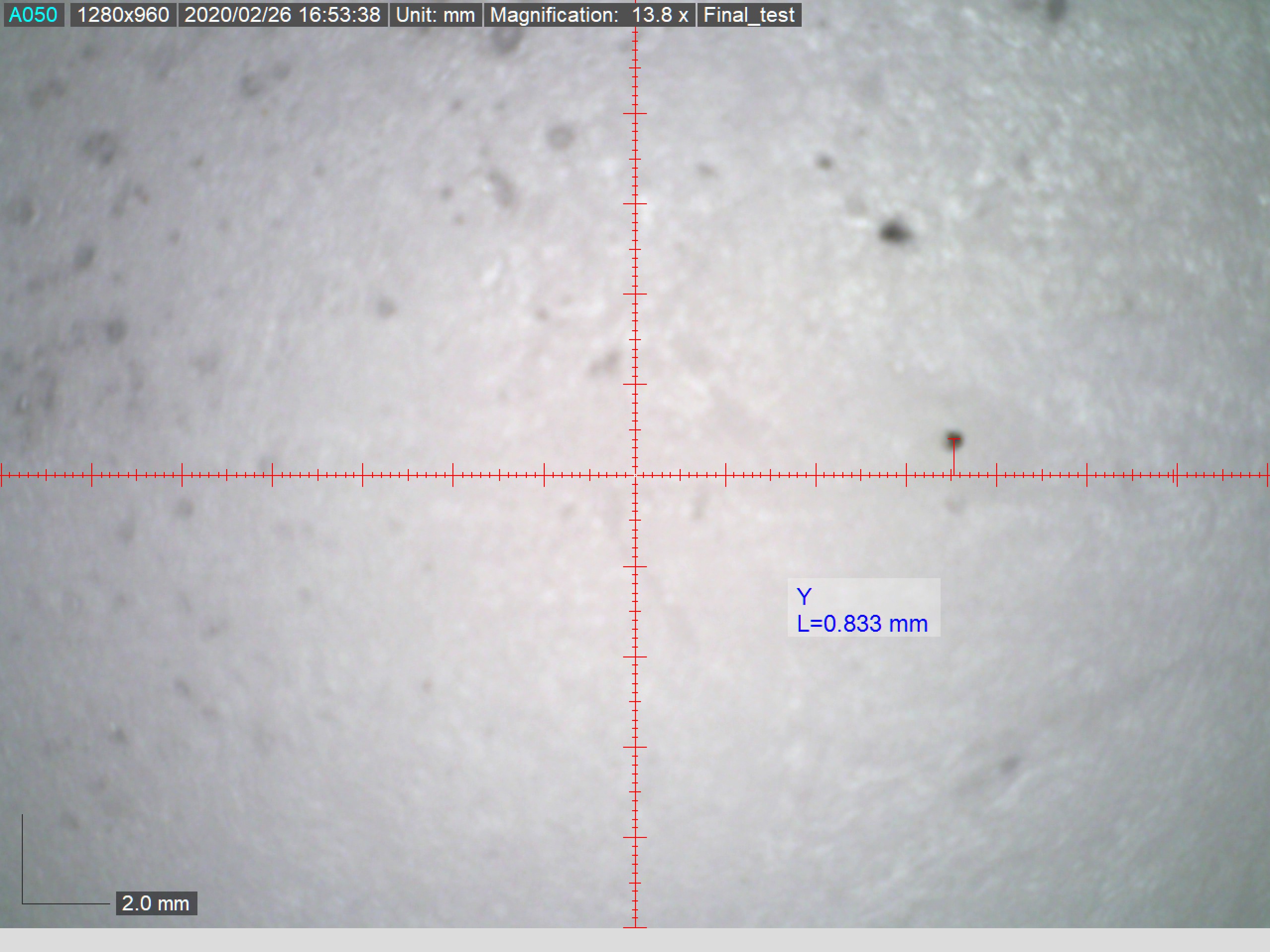

Supplement: S3 File — (ZIP) [file pone.0261089.s003.zip › Stiff phantom/fotos49.jpg]

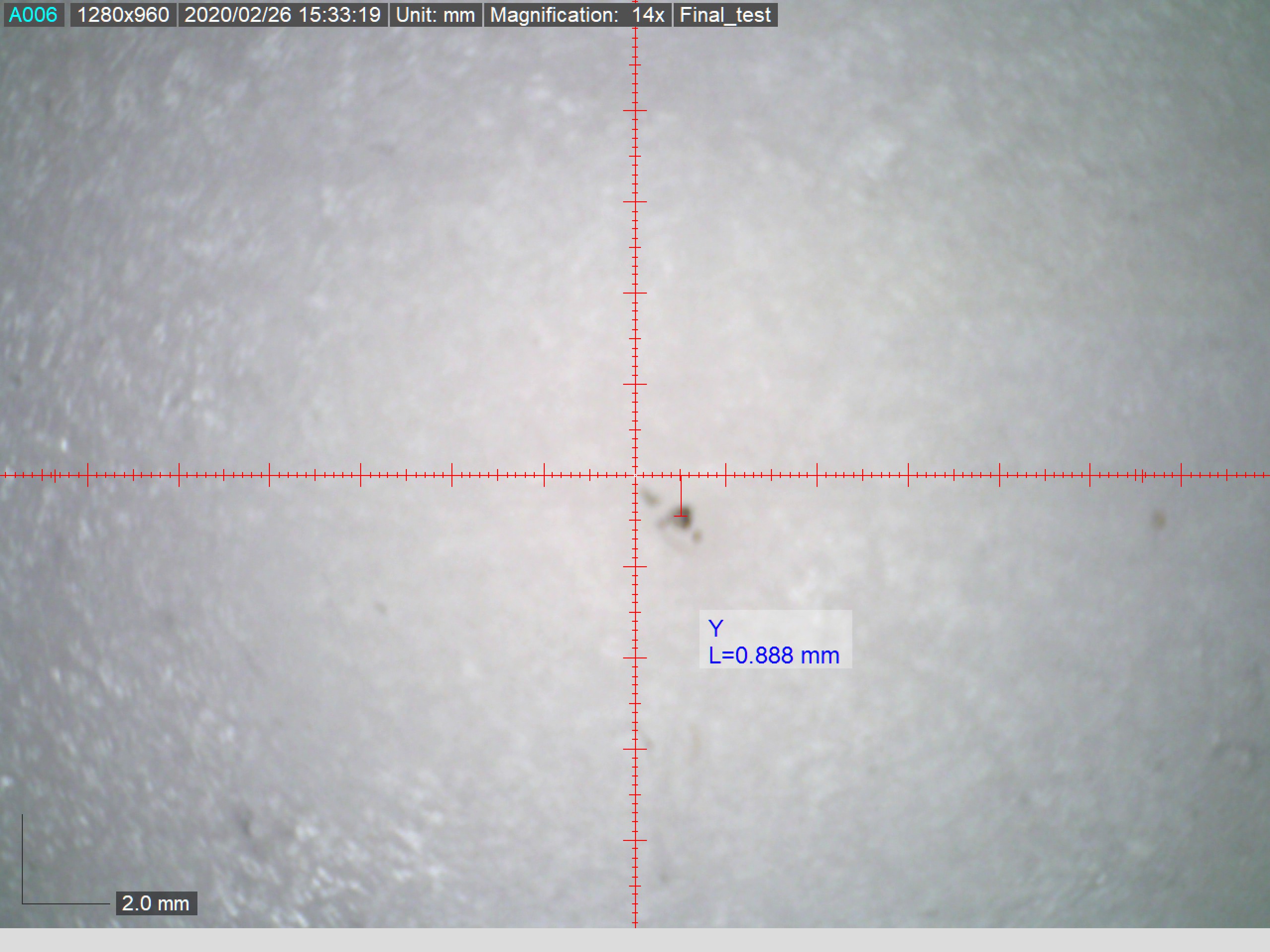

Supplement: S3 File — (ZIP) [file pone.0261089.s003.zip › Stiff phantom/fotos5.jpg]

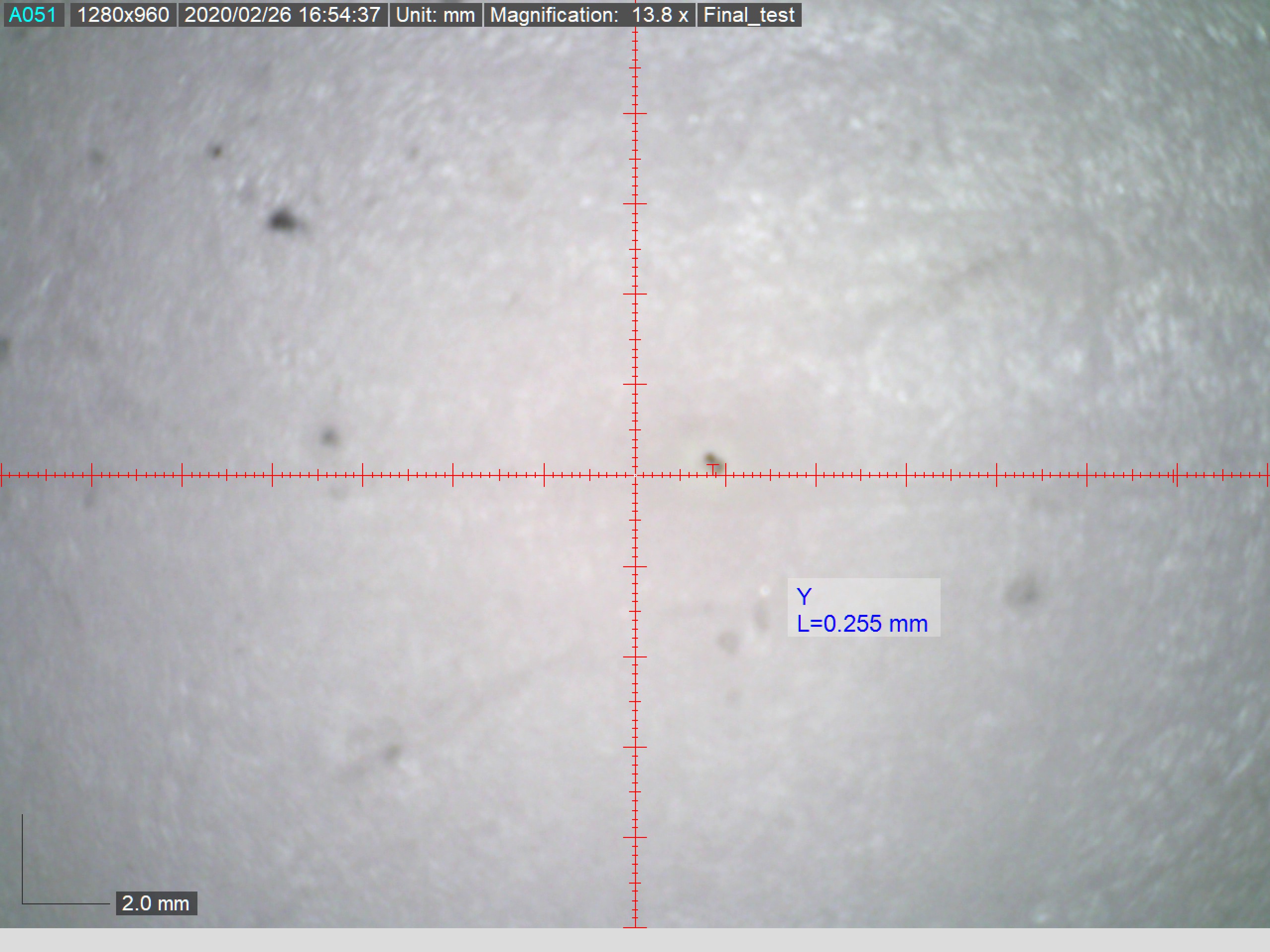

Supplement: S3 File — (ZIP) [file pone.0261089.s003.zip › Stiff phantom/fotos50.jpg]

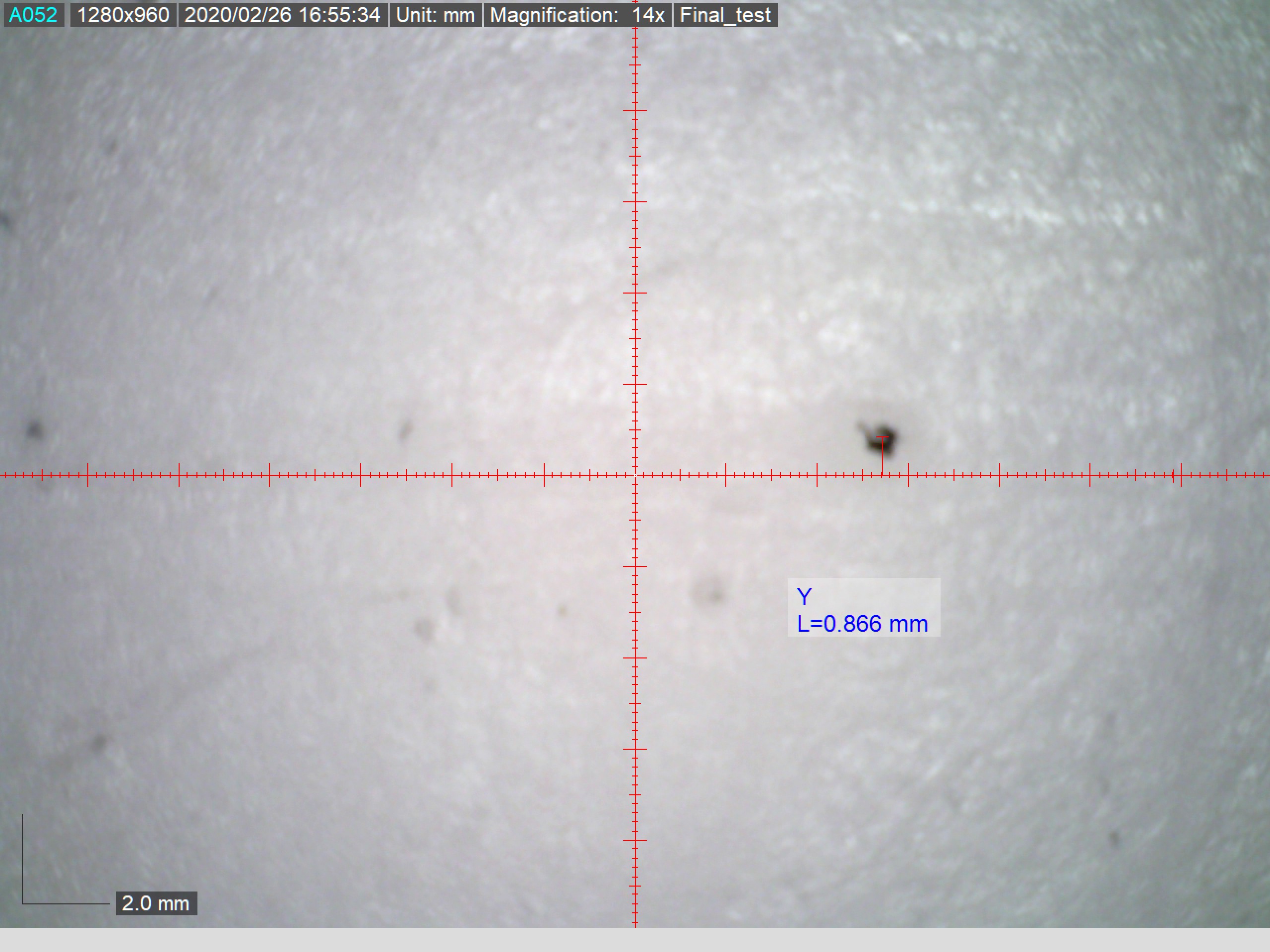

Supplement: S3 File — (ZIP) [file pone.0261089.s003.zip › Stiff phantom/fotos51.jpg]

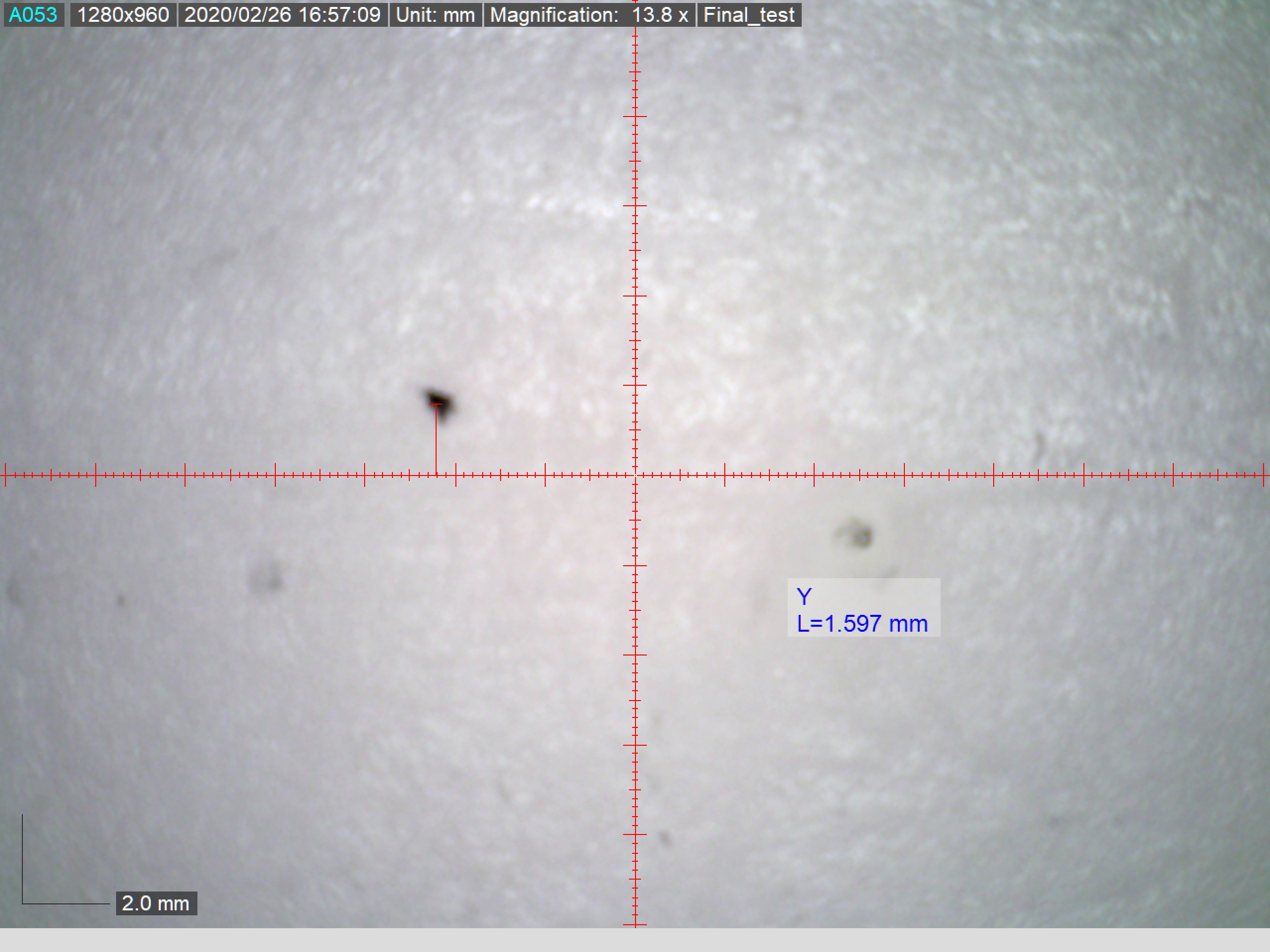

Supplement: S3 File — (ZIP) [file pone.0261089.s003.zip › Stiff phantom/fotos52.jpg]

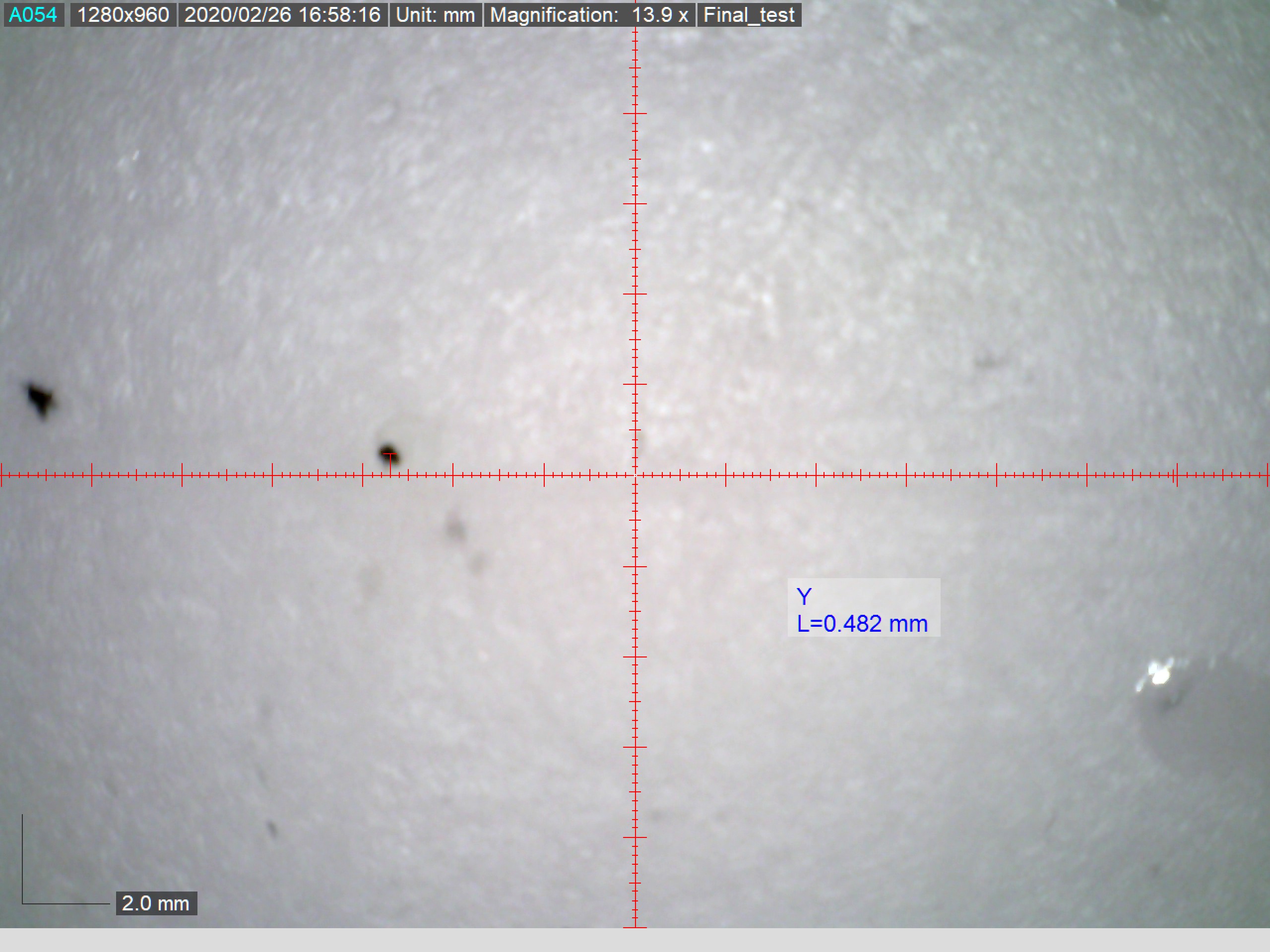

Supplement: S3 File — (ZIP) [file pone.0261089.s003.zip › Stiff phantom/fotos53.jpg]

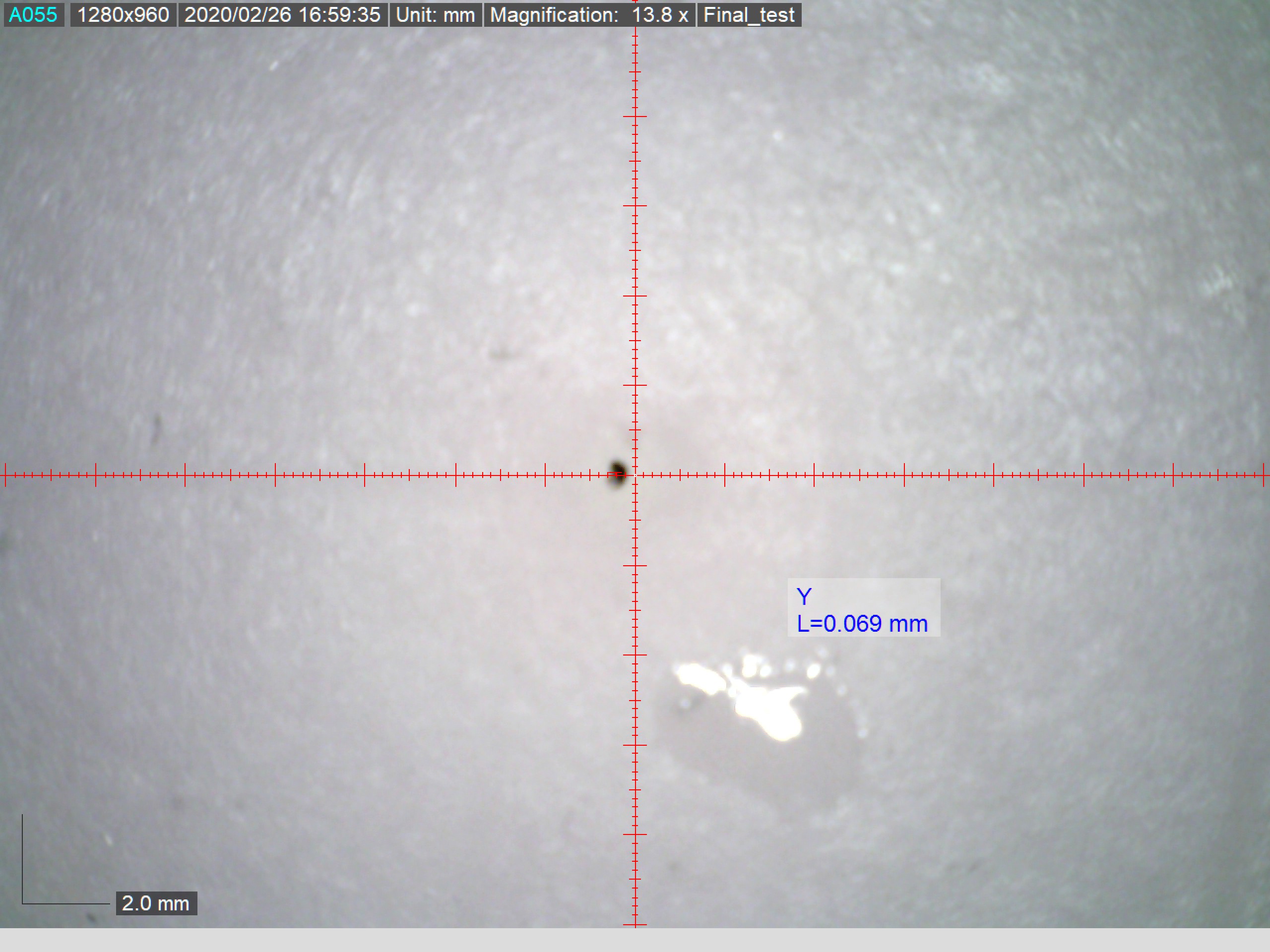

Supplement: S3 File — (ZIP) [file pone.0261089.s003.zip › Stiff phantom/fotos54.jpg]

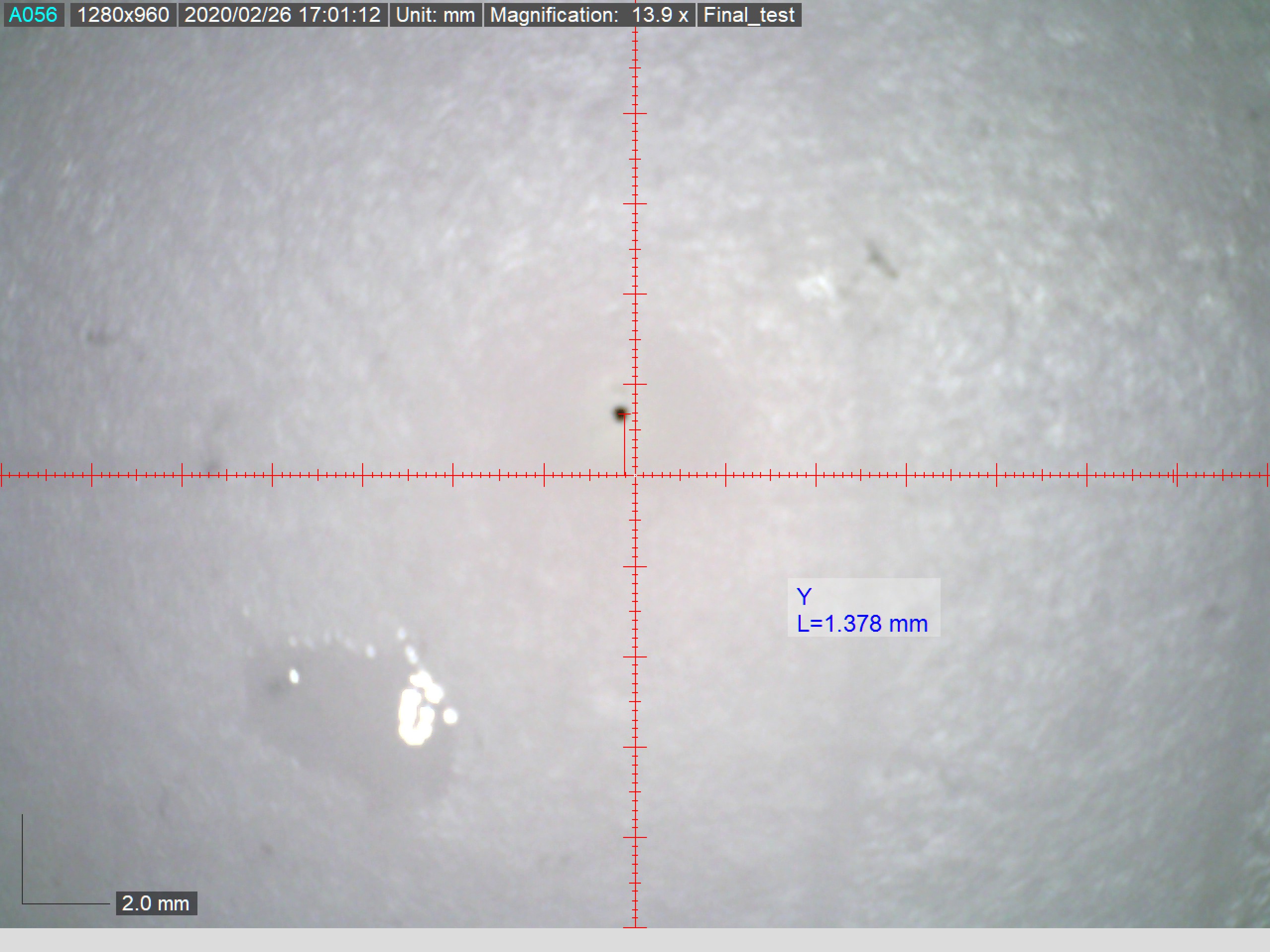

Supplement: S3 File — (ZIP) [file pone.0261089.s003.zip › Stiff phantom/fotos55.jpg]

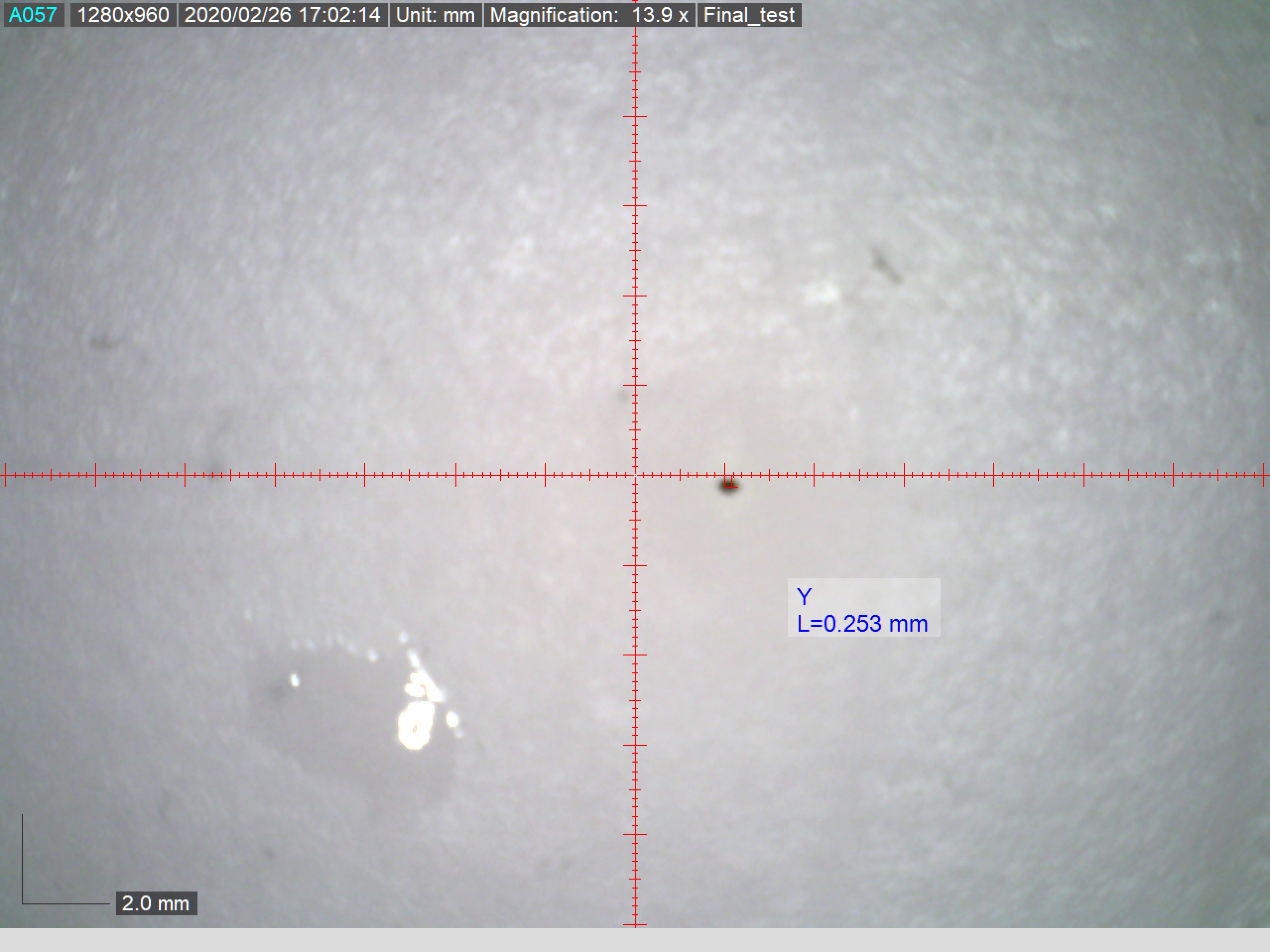

Supplement: S3 File — (ZIP) [file pone.0261089.s003.zip › Stiff phantom/fotos56.jpg]

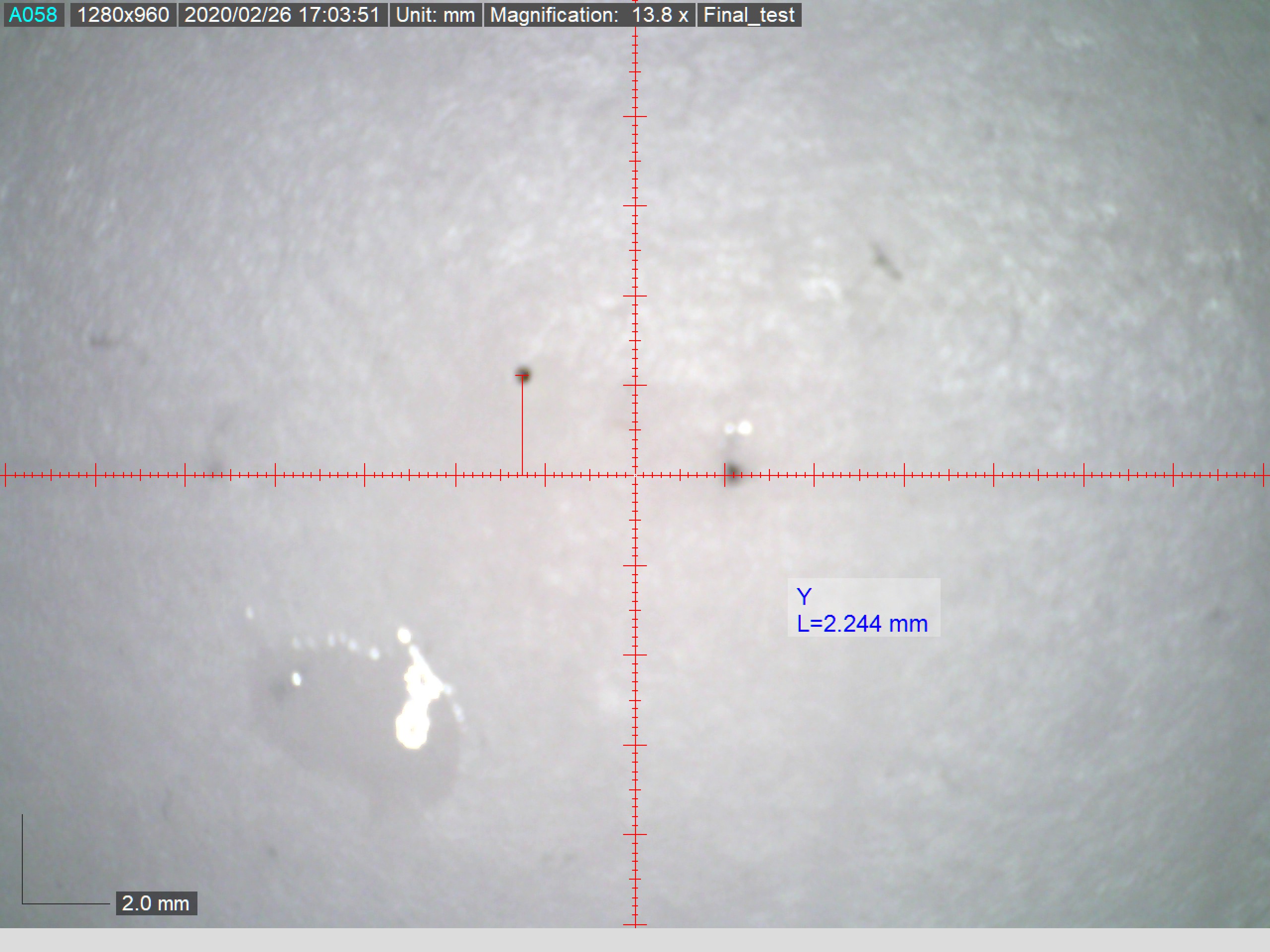

Supplement: S3 File — (ZIP) [file pone.0261089.s003.zip › Stiff phantom/fotos57.jpg]

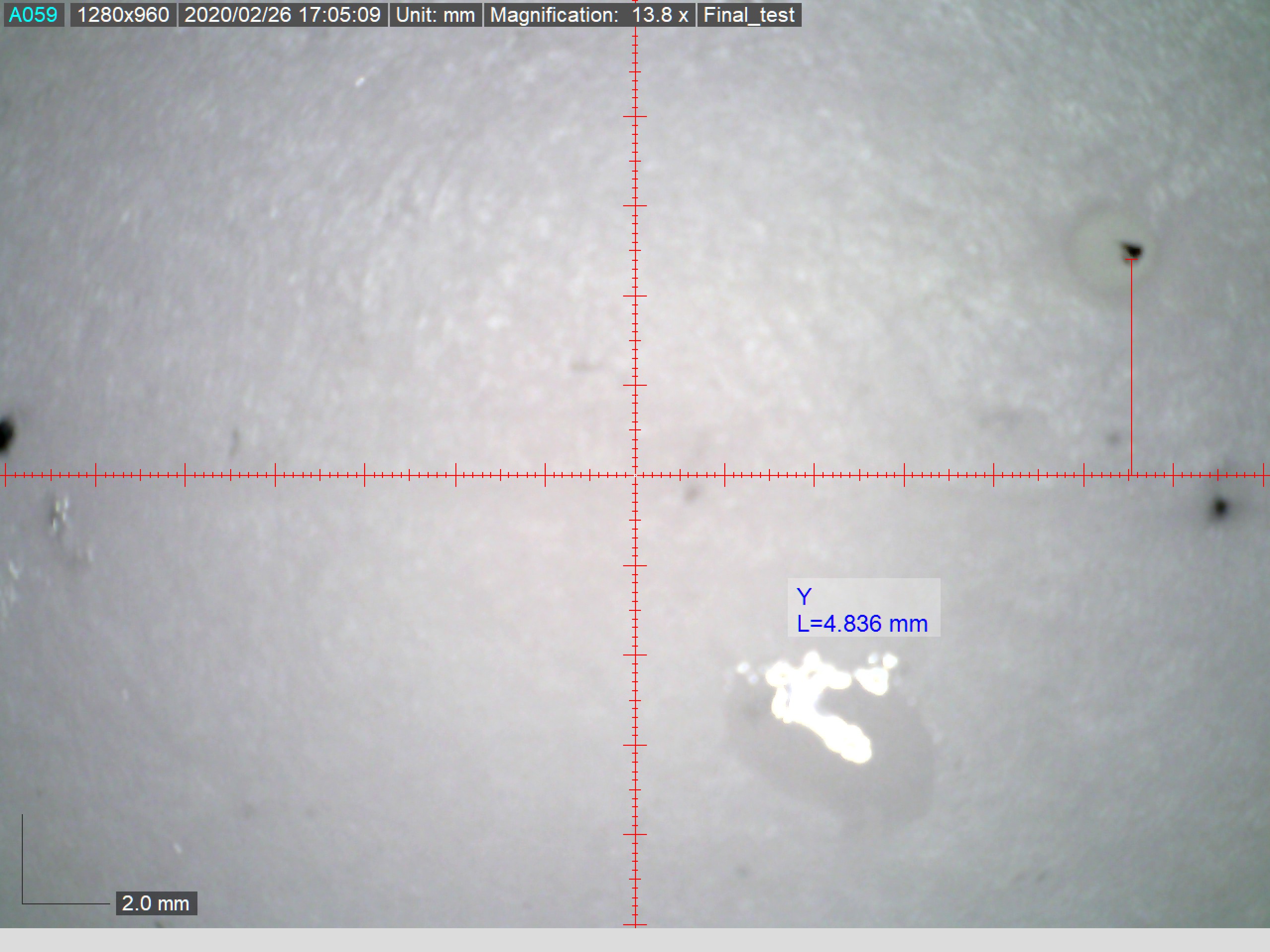

Supplement: S3 File — (ZIP) [file pone.0261089.s003.zip › Stiff phantom/fotos58.jpg]

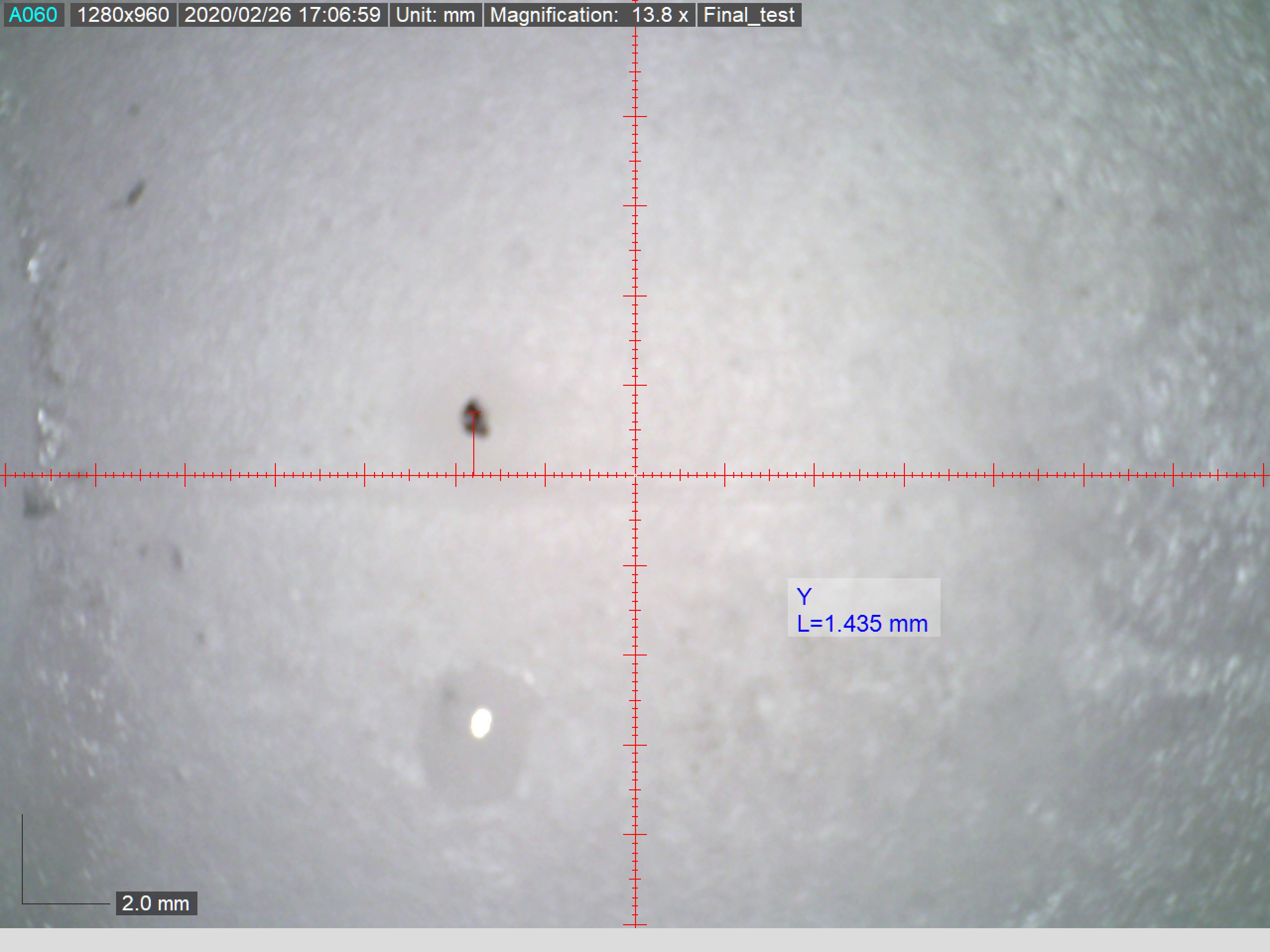

Supplement: S3 File — (ZIP) [file pone.0261089.s003.zip › Stiff phantom/fotos59.jpg]

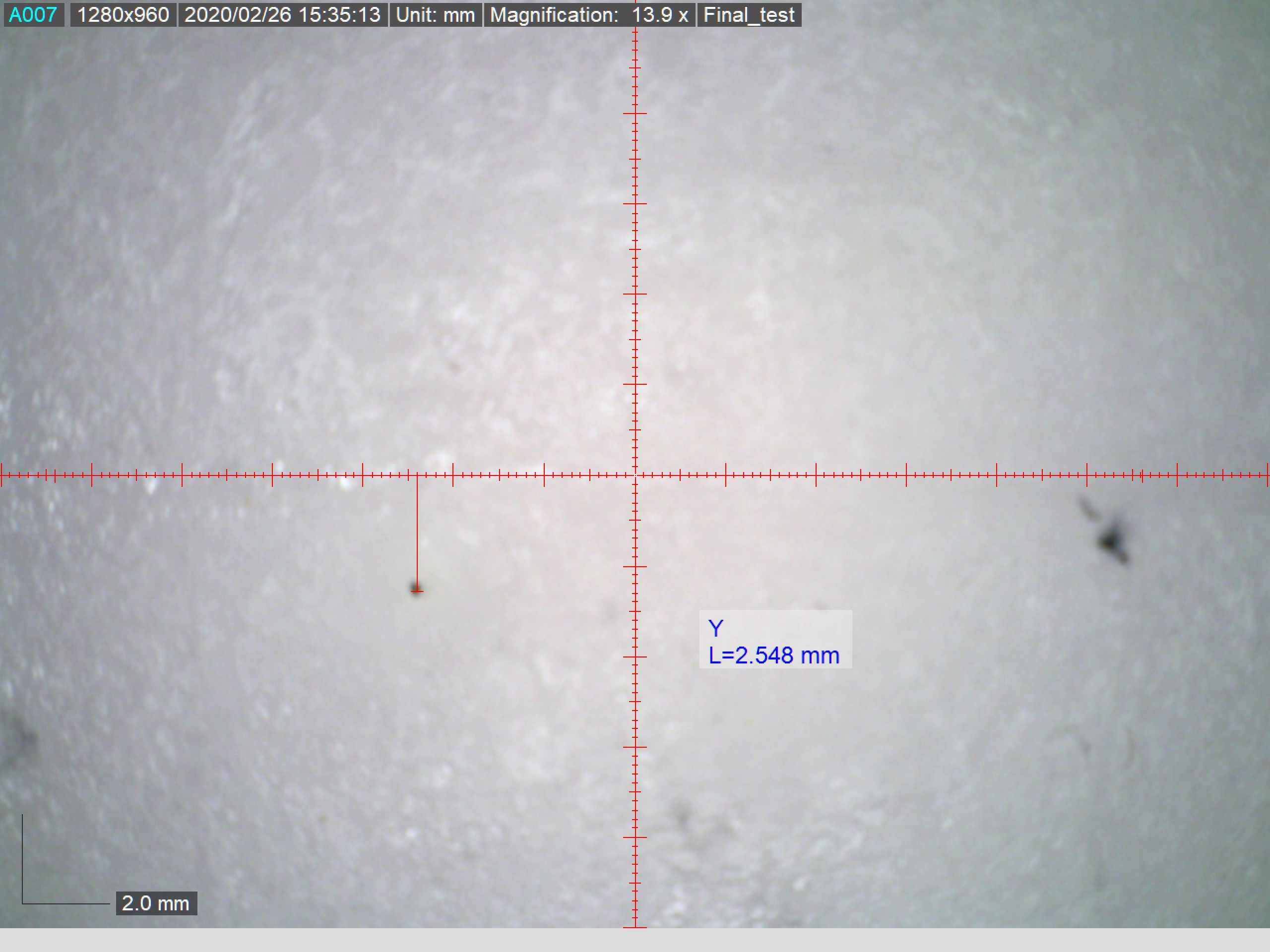

Supplement: S3 File — (ZIP) [file pone.0261089.s003.zip › Stiff phantom/fotos6.jpg]

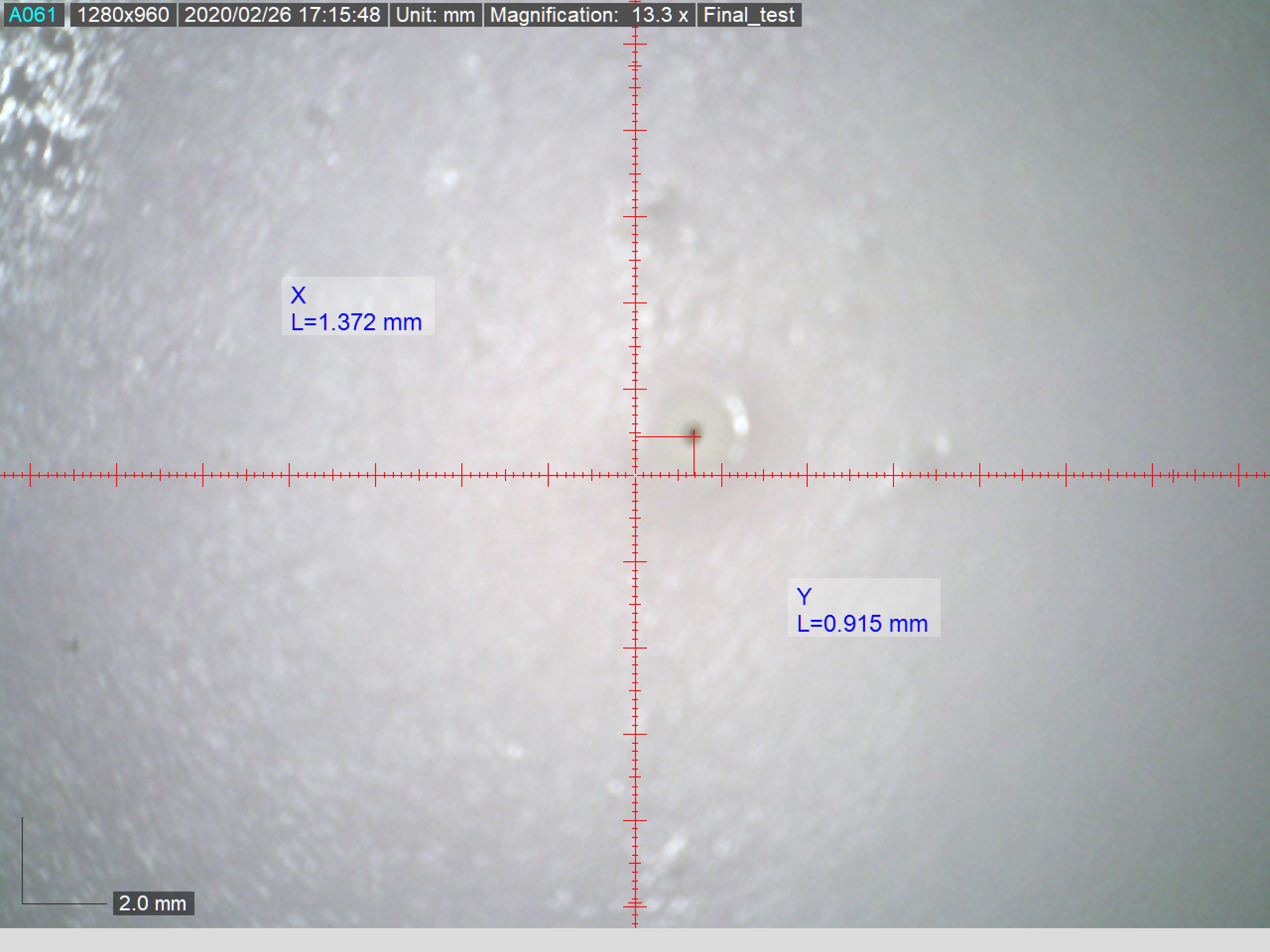

Supplement: S3 File — (ZIP) [file pone.0261089.s003.zip › Stiff phantom/fotos60.jpg]

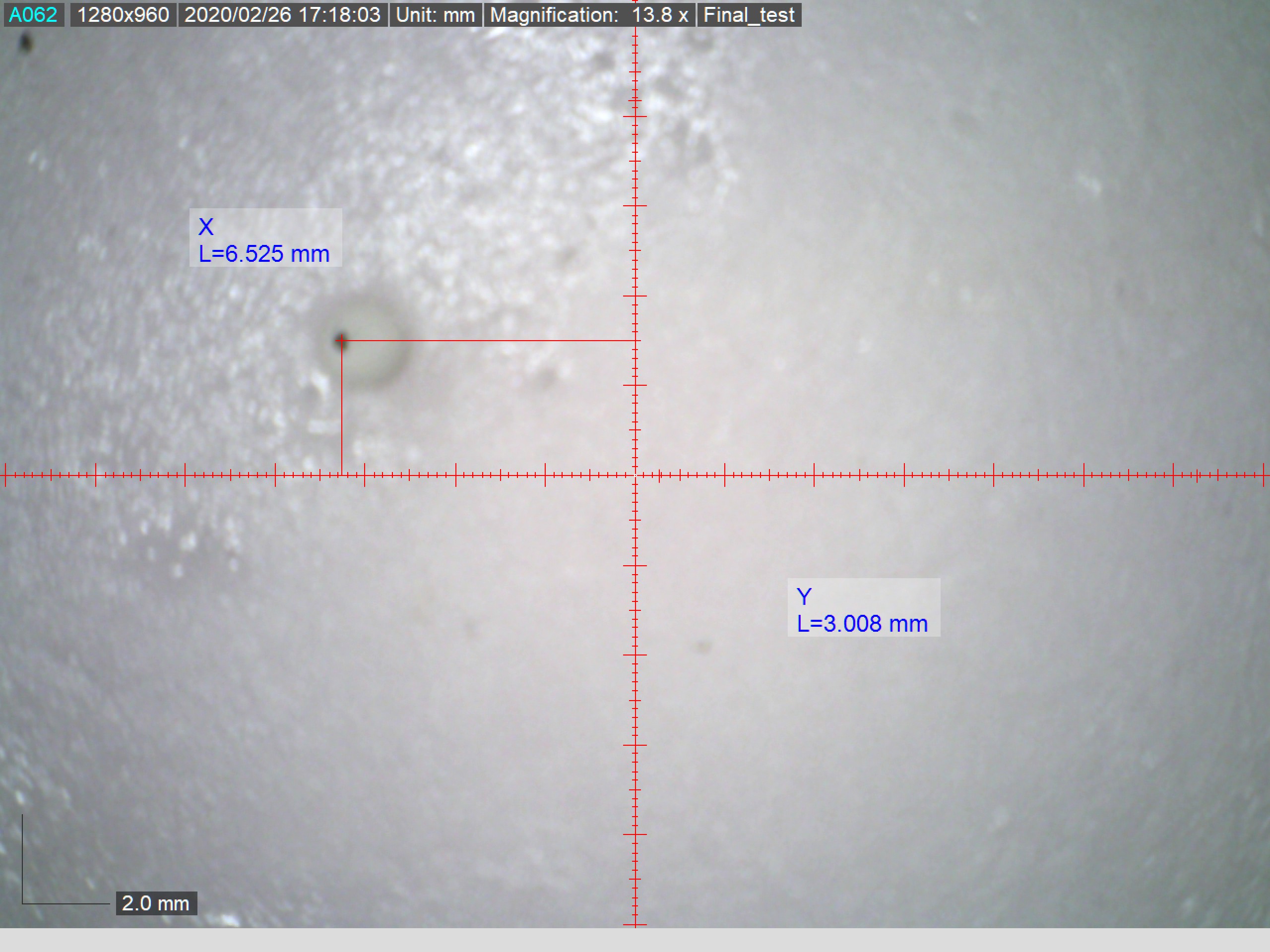

Supplement: S3 File — (ZIP) [file pone.0261089.s003.zip › Stiff phantom/fotos61.jpg]

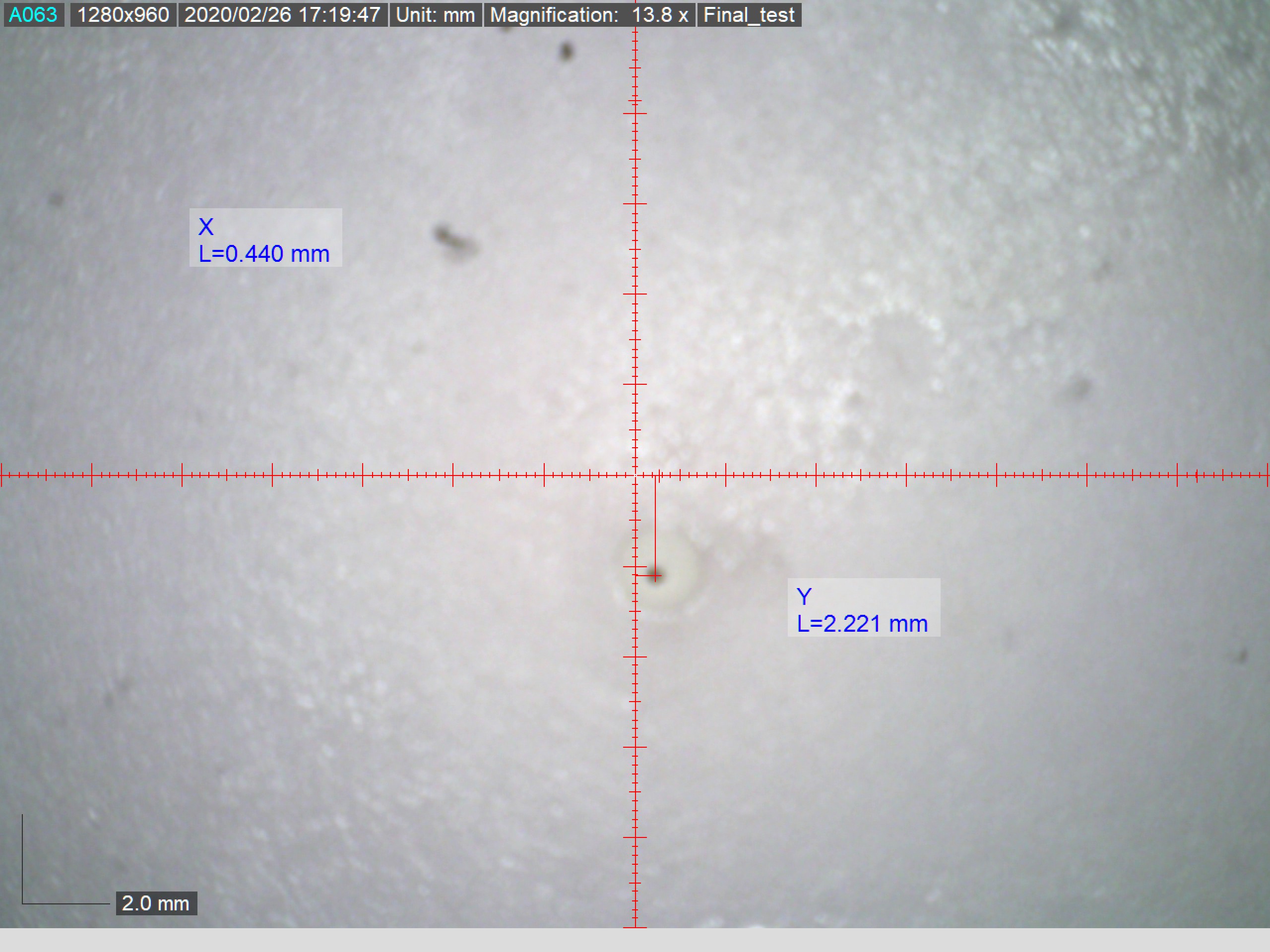

Supplement: S3 File — (ZIP) [file pone.0261089.s003.zip › Stiff phantom/fotos62.jpg]

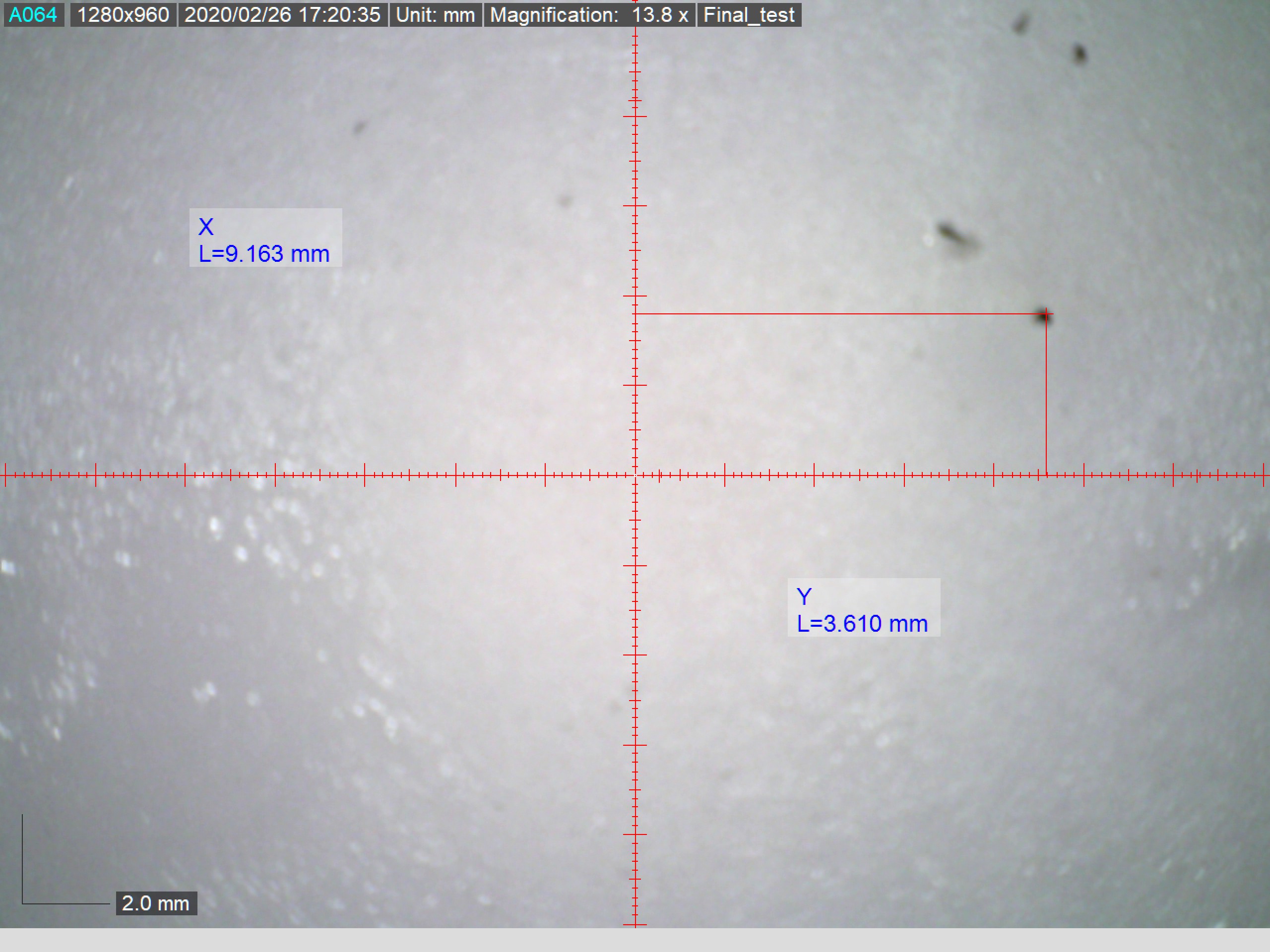

Supplement: S3 File — (ZIP) [file pone.0261089.s003.zip › Stiff phantom/fotos63.jpg]

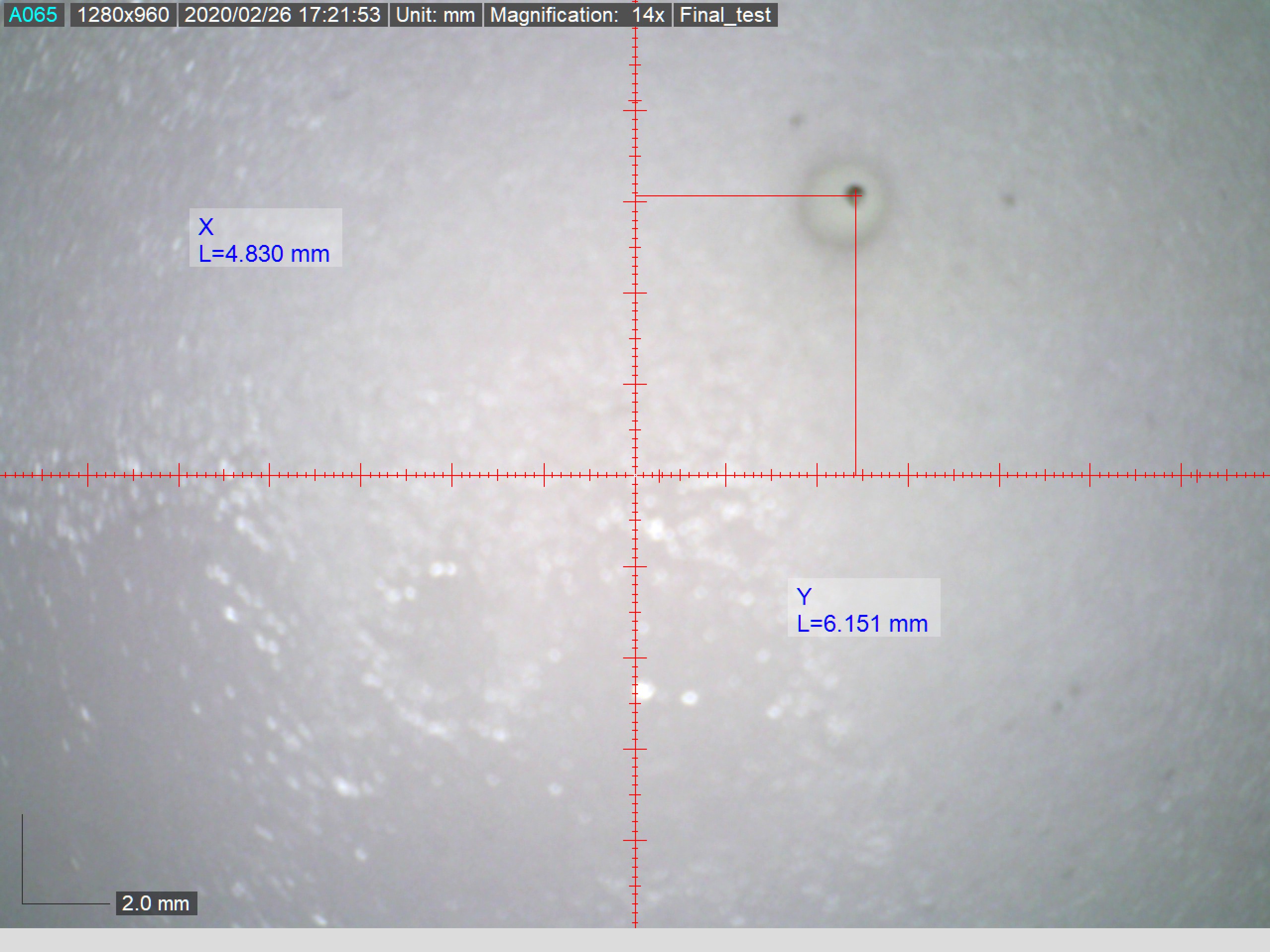

Supplement: S3 File — (ZIP) [file pone.0261089.s003.zip › Stiff phantom/fotos64.jpg]

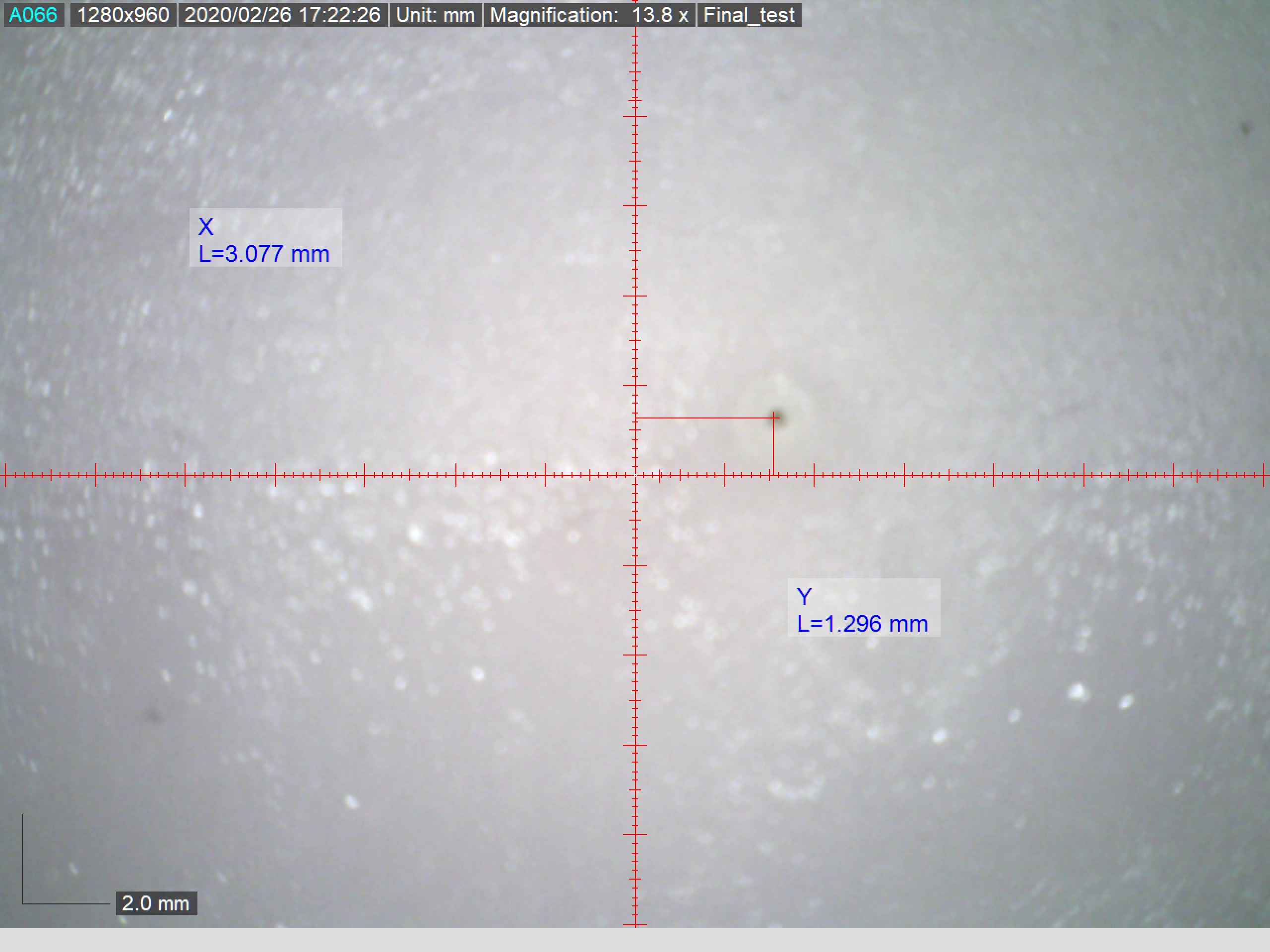

Supplement: S3 File — (ZIP) [file pone.0261089.s003.zip › Stiff phantom/fotos65.jpg]

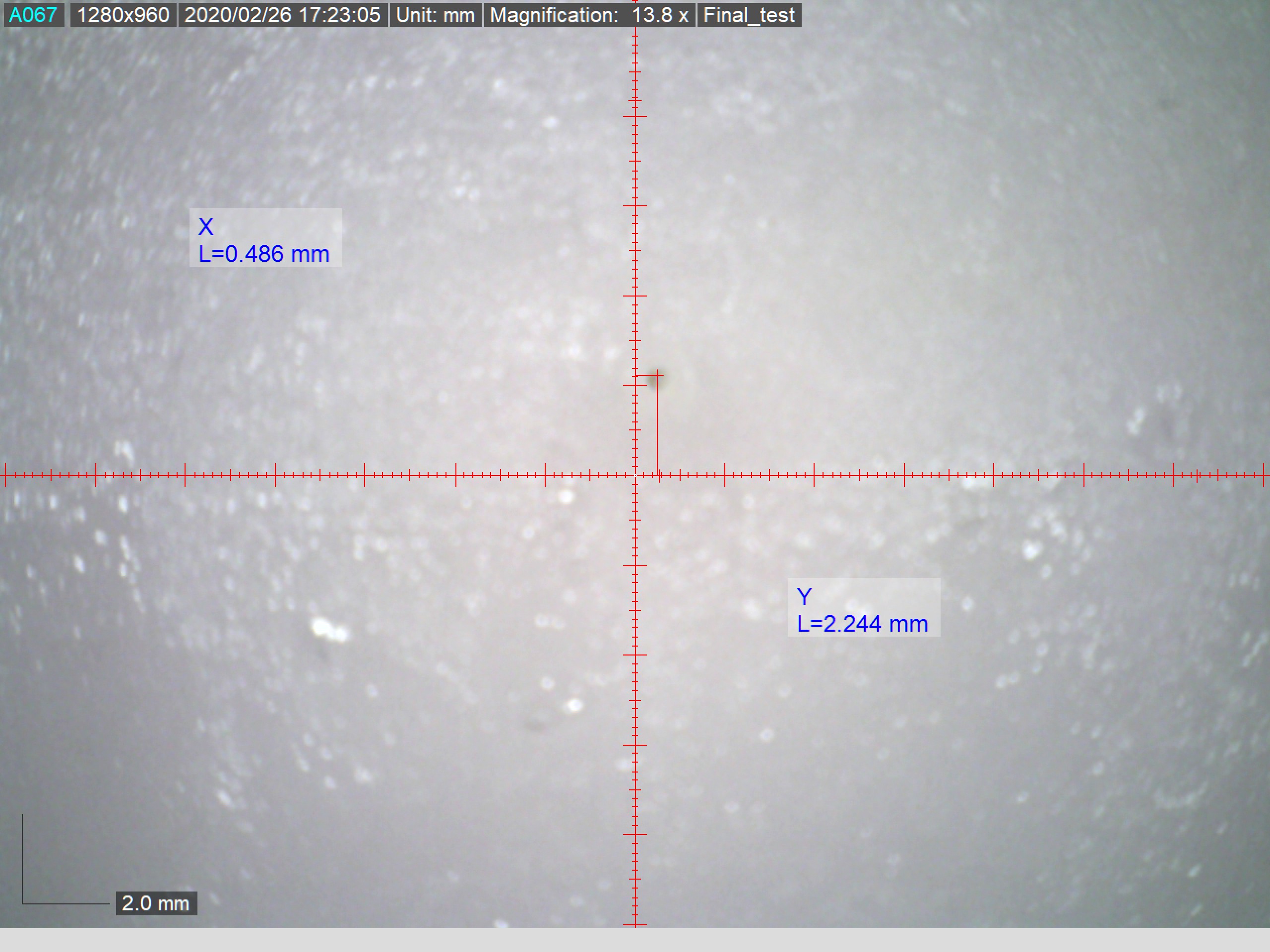

Supplement: S3 File — (ZIP) [file pone.0261089.s003.zip › Stiff phantom/fotos66.jpg]

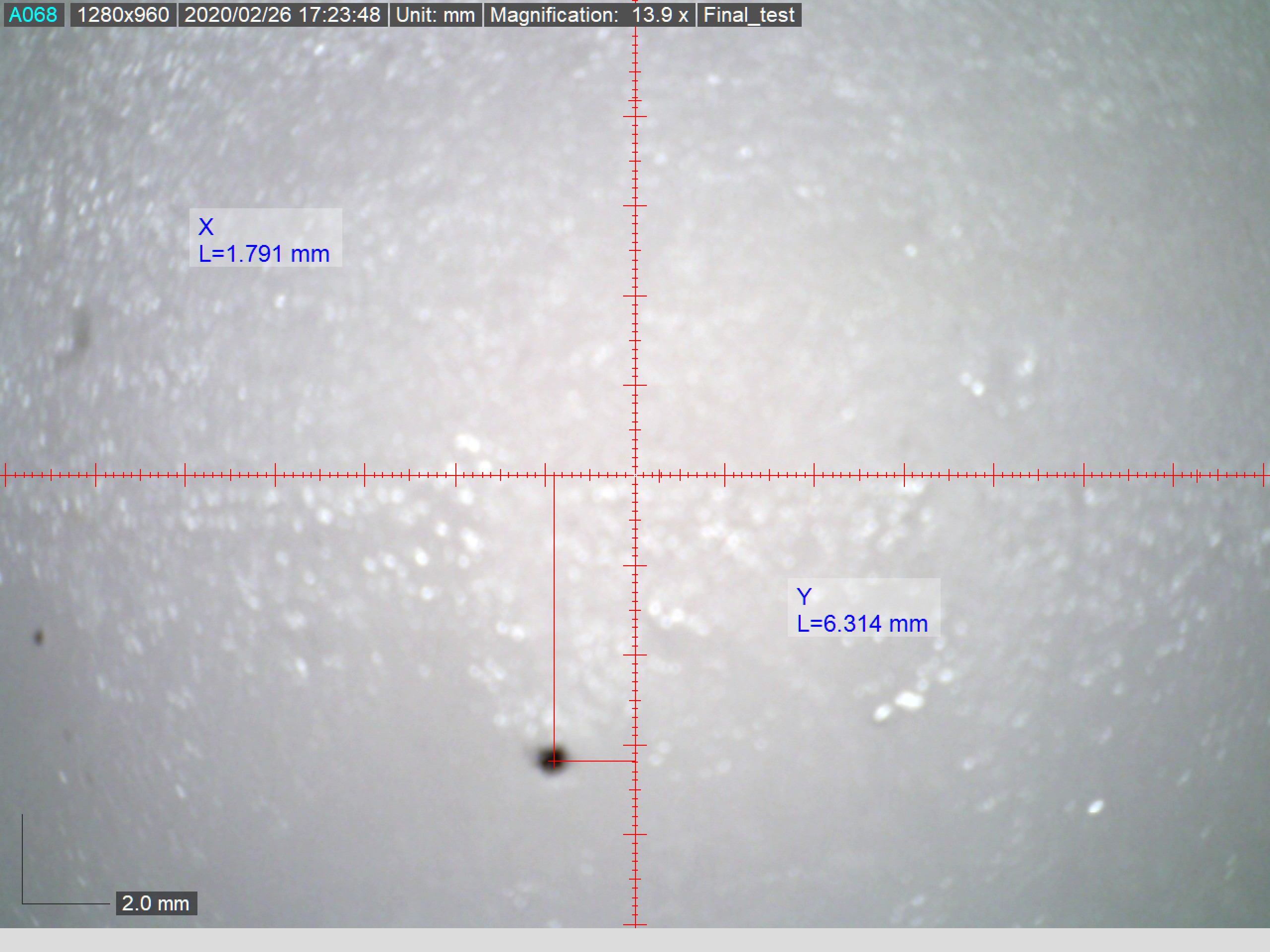

Supplement: S3 File — (ZIP) [file pone.0261089.s003.zip › Stiff phantom/fotos67.jpg]

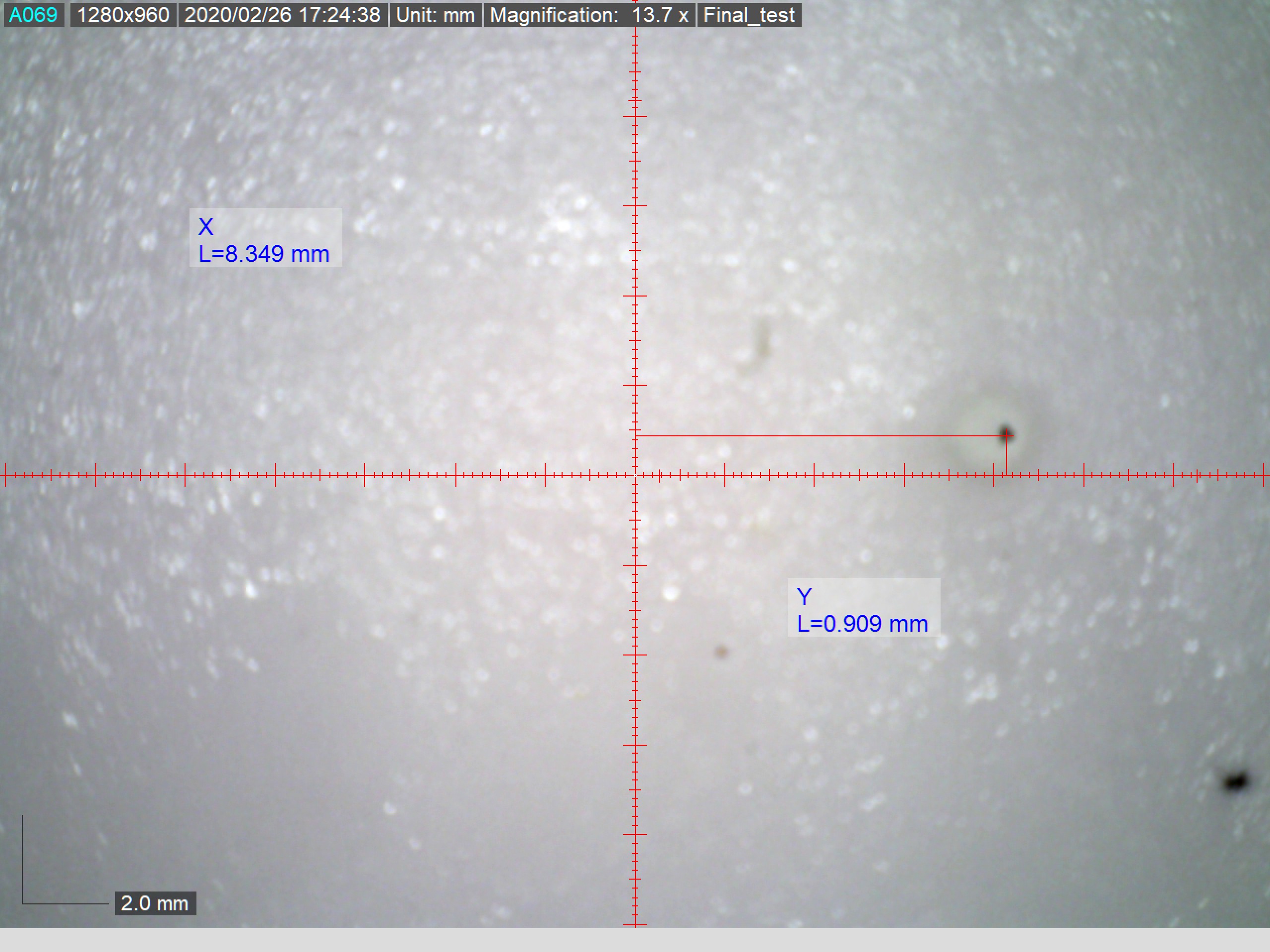

Supplement: S3 File — (ZIP) [file pone.0261089.s003.zip › Stiff phantom/fotos68.jpg]

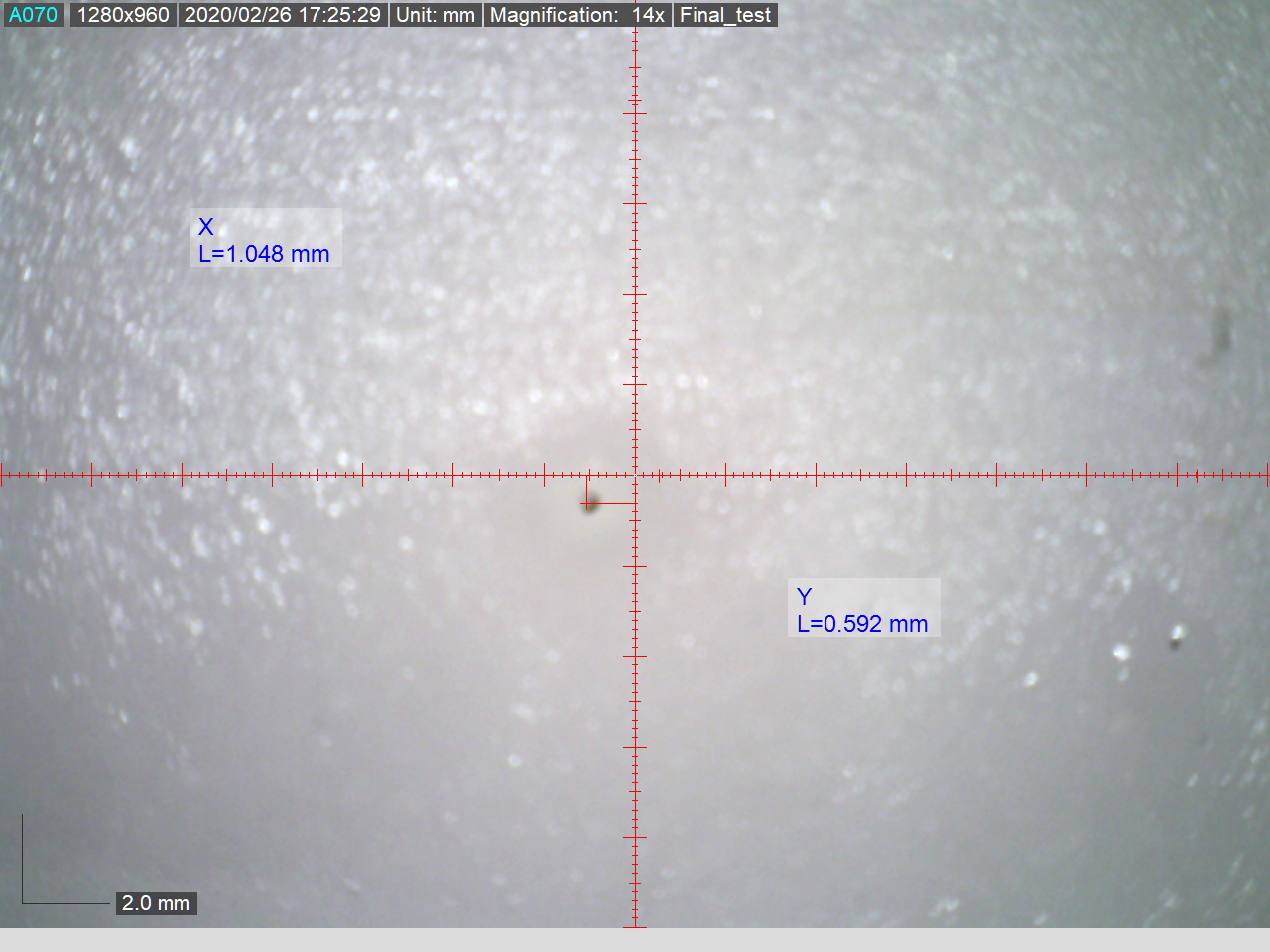

Supplement: S3 File — (ZIP) [file pone.0261089.s003.zip › Stiff phantom/fotos69.jpg]

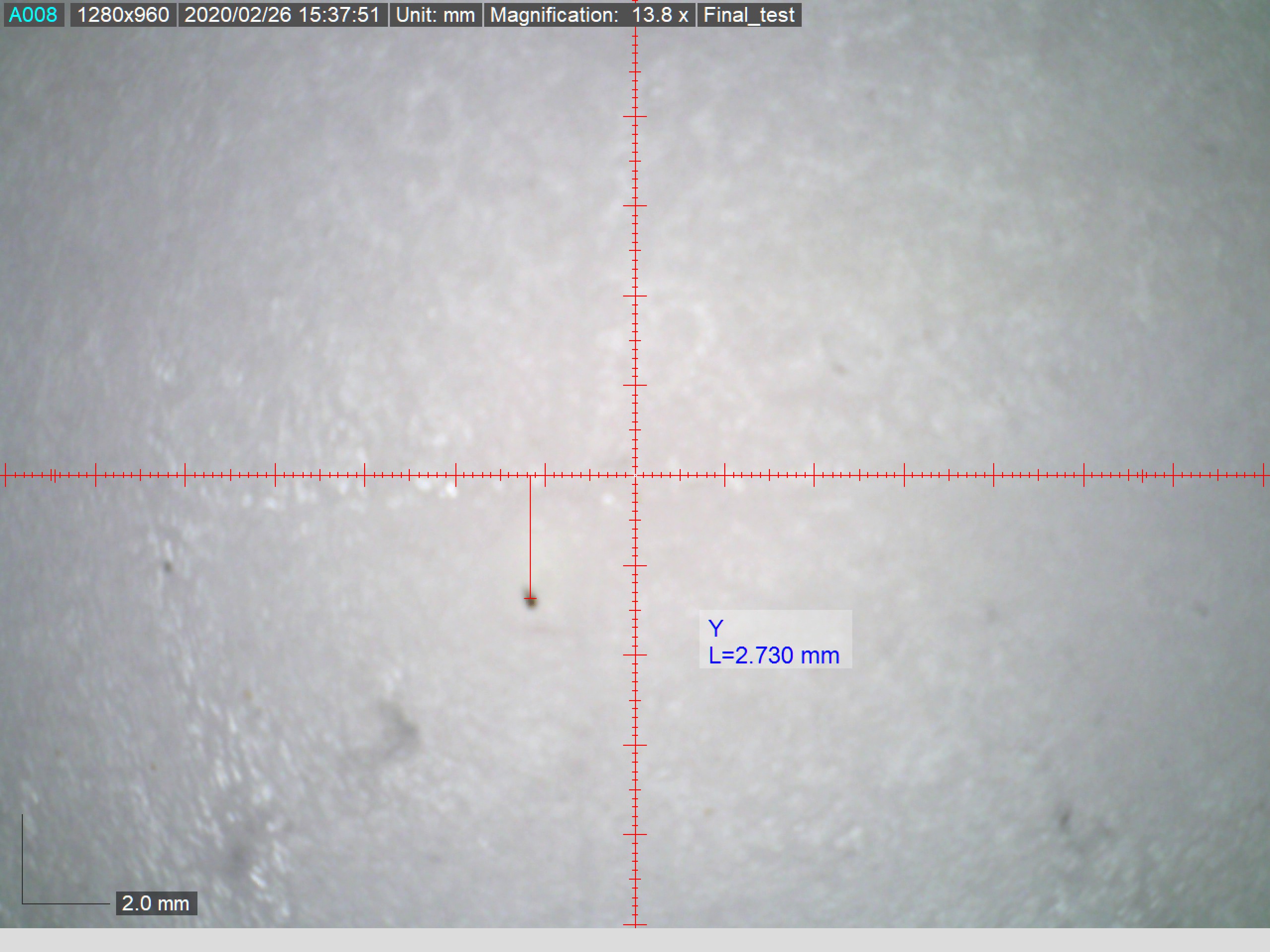

Supplement: S3 File — (ZIP) [file pone.0261089.s003.zip › Stiff phantom/fotos7.jpg]

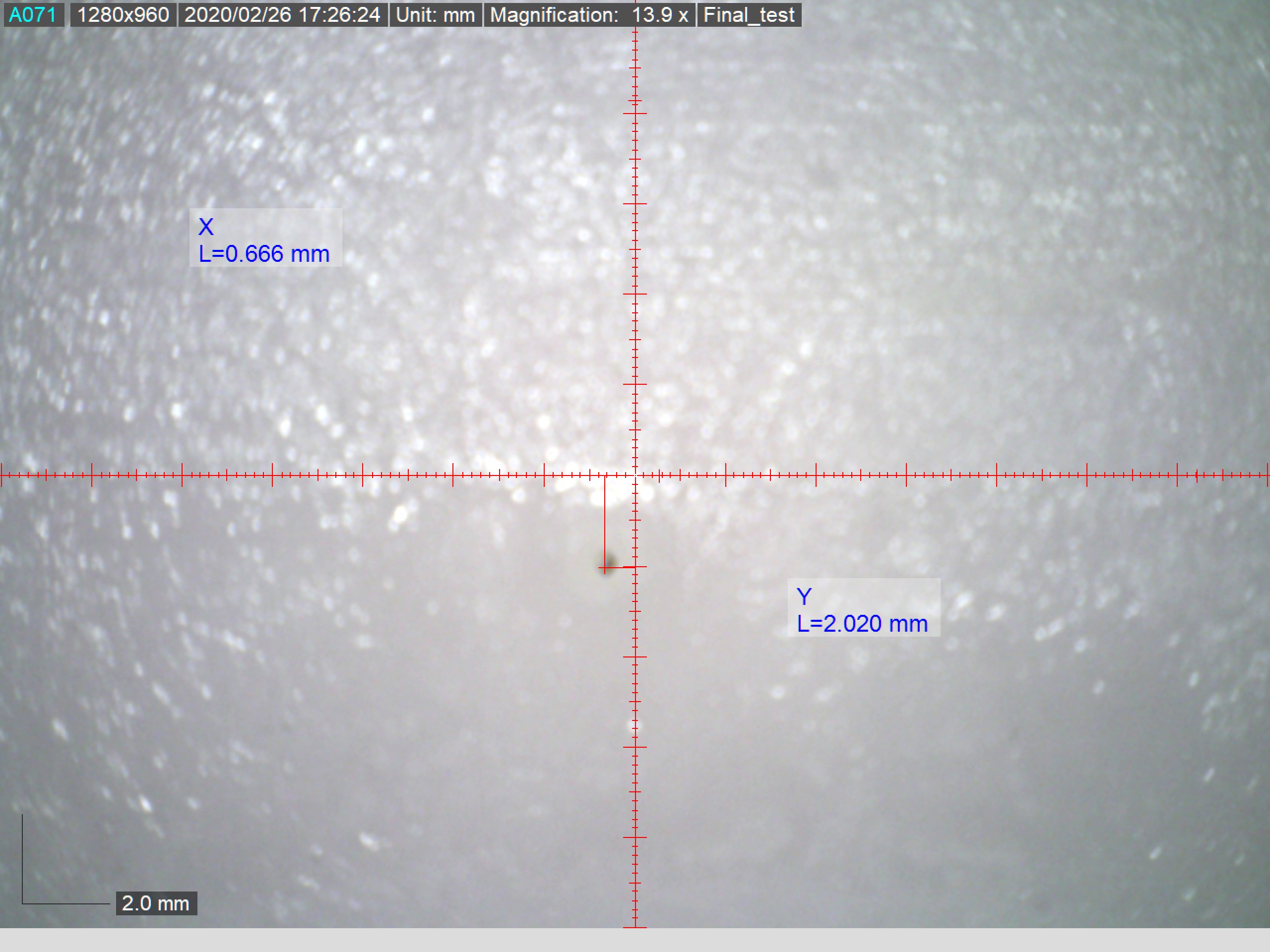

Supplement: S3 File — (ZIP) [file pone.0261089.s003.zip › Stiff phantom/fotos70.jpg]

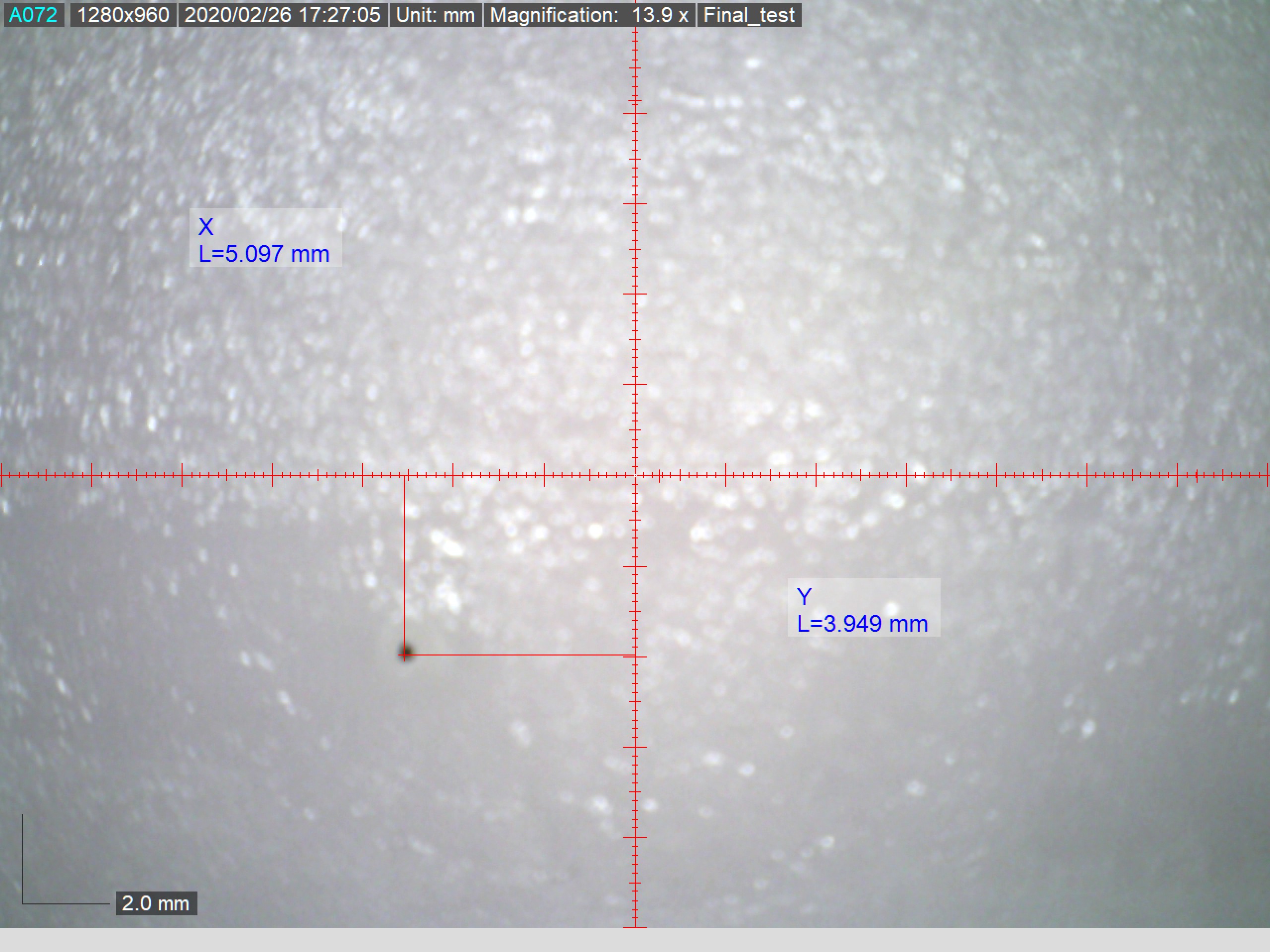

Supplement: S3 File — (ZIP) [file pone.0261089.s003.zip › Stiff phantom/fotos71.jpg]

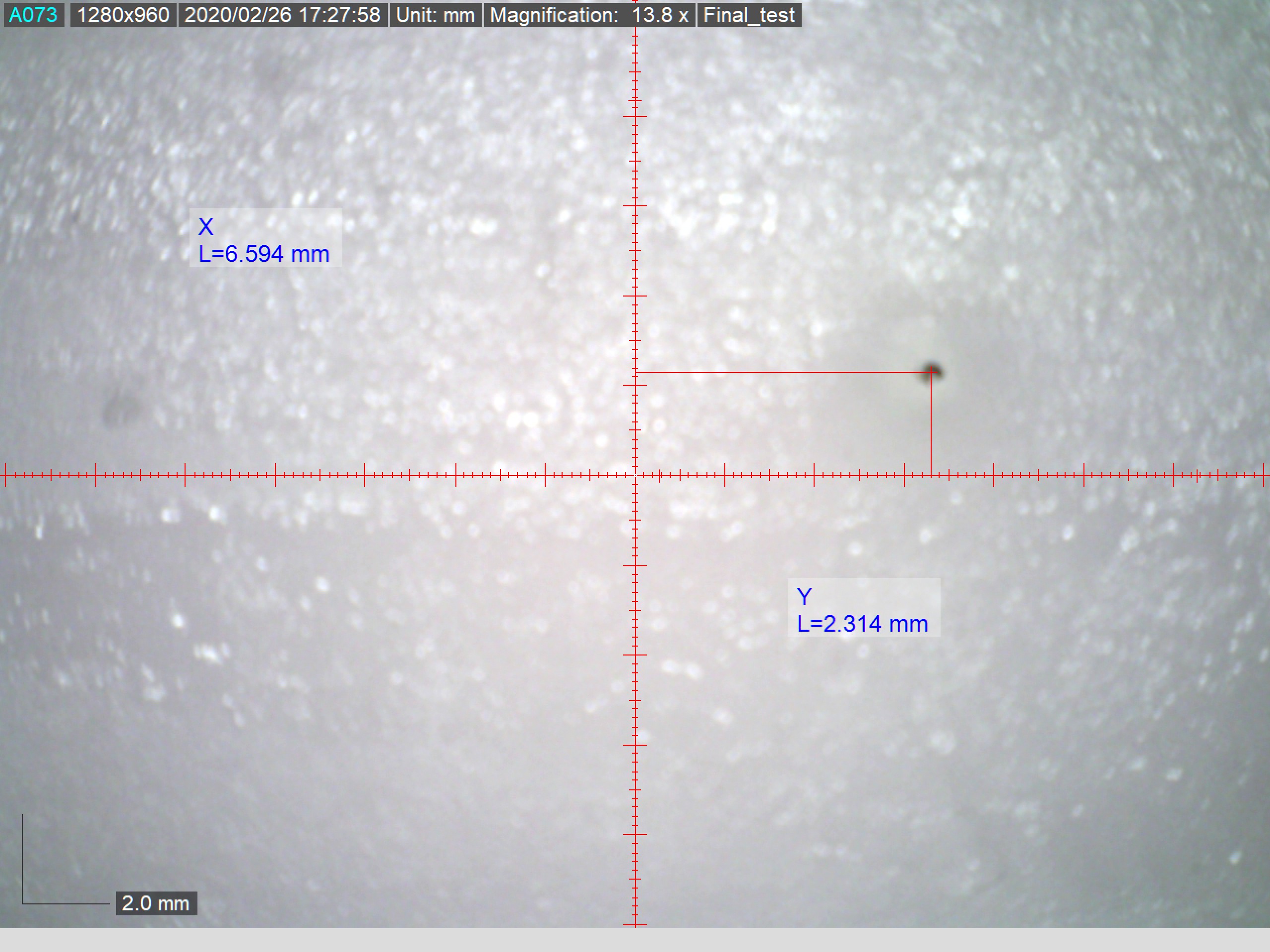

Supplement: S3 File — (ZIP) [file pone.0261089.s003.zip › Stiff phantom/fotos72.jpg]

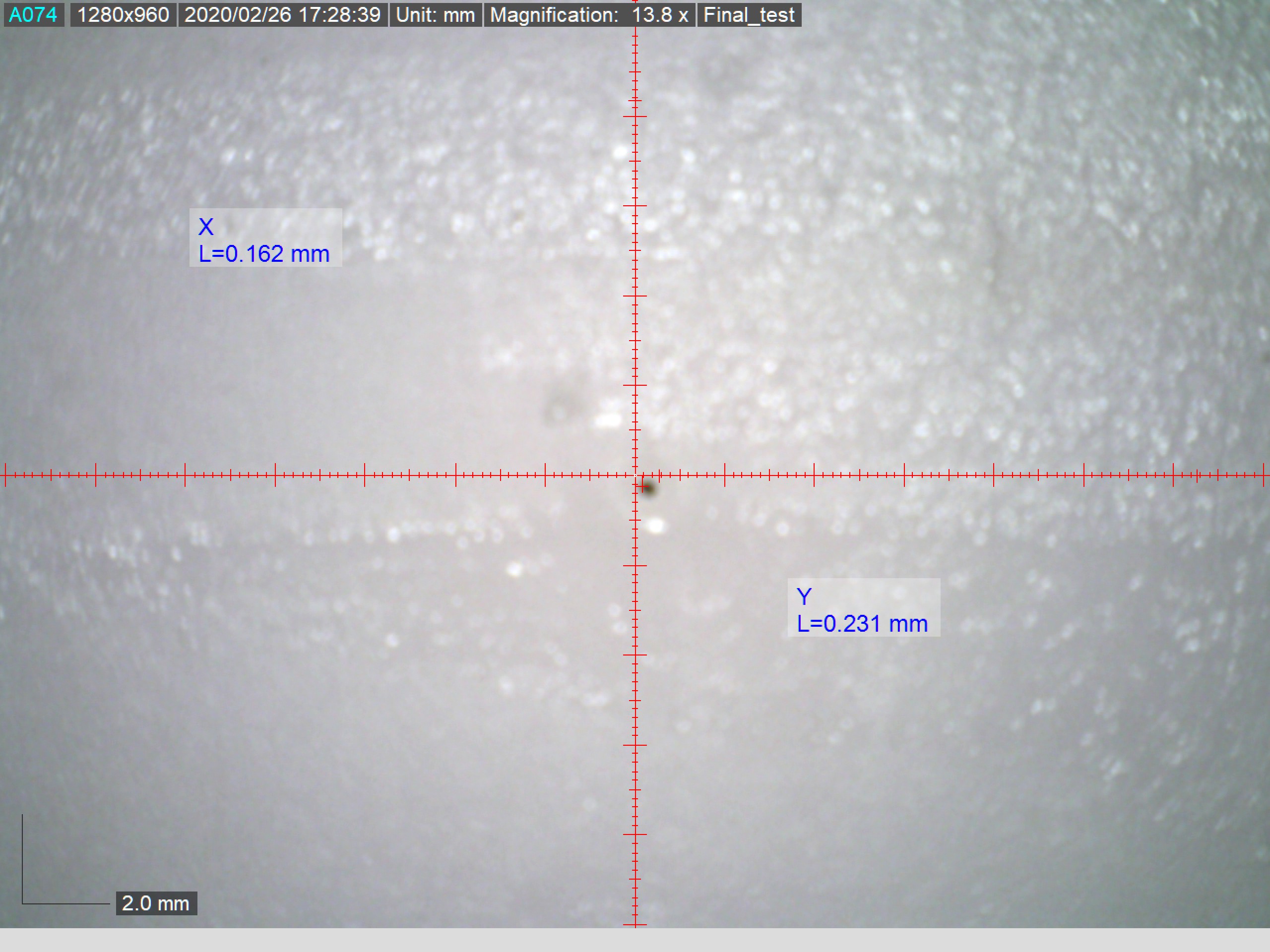

Supplement: S3 File — (ZIP) [file pone.0261089.s003.zip › Stiff phantom/fotos73.jpg]

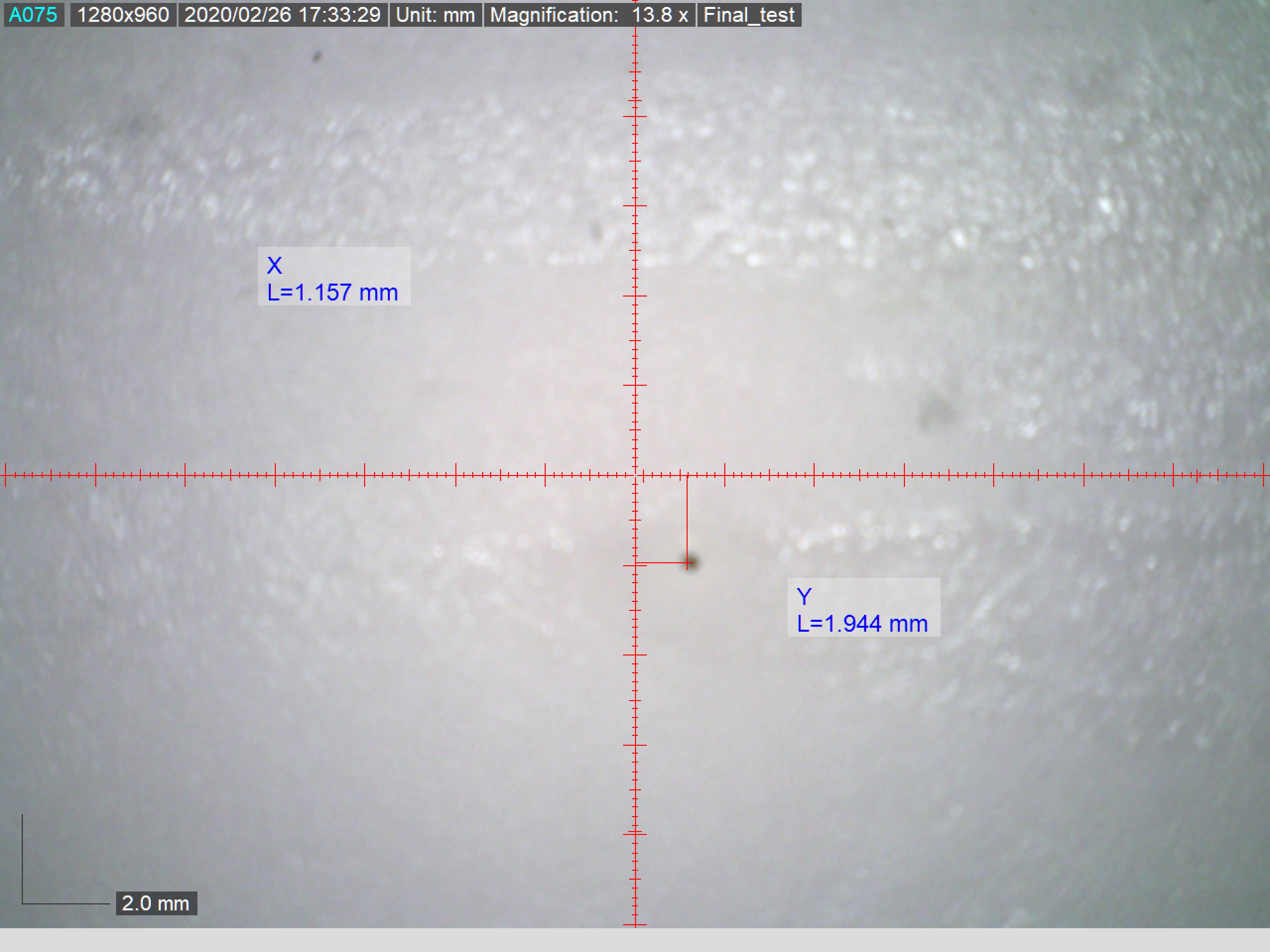

Supplement: S3 File — (ZIP) [file pone.0261089.s003.zip › Stiff phantom/fotos74.jpg]

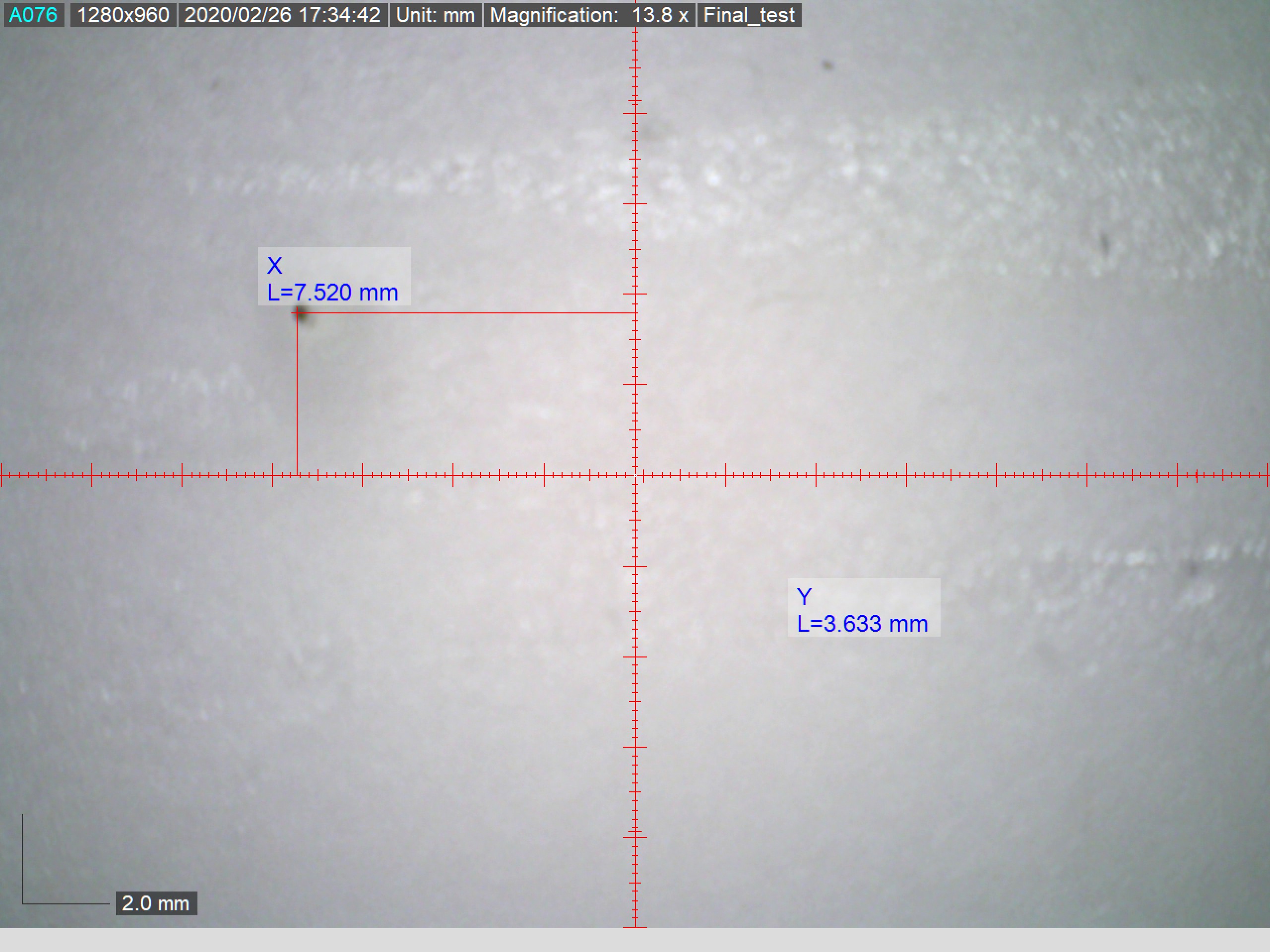

Supplement: S3 File — (ZIP) [file pone.0261089.s003.zip › Stiff phantom/fotos75.jpg]

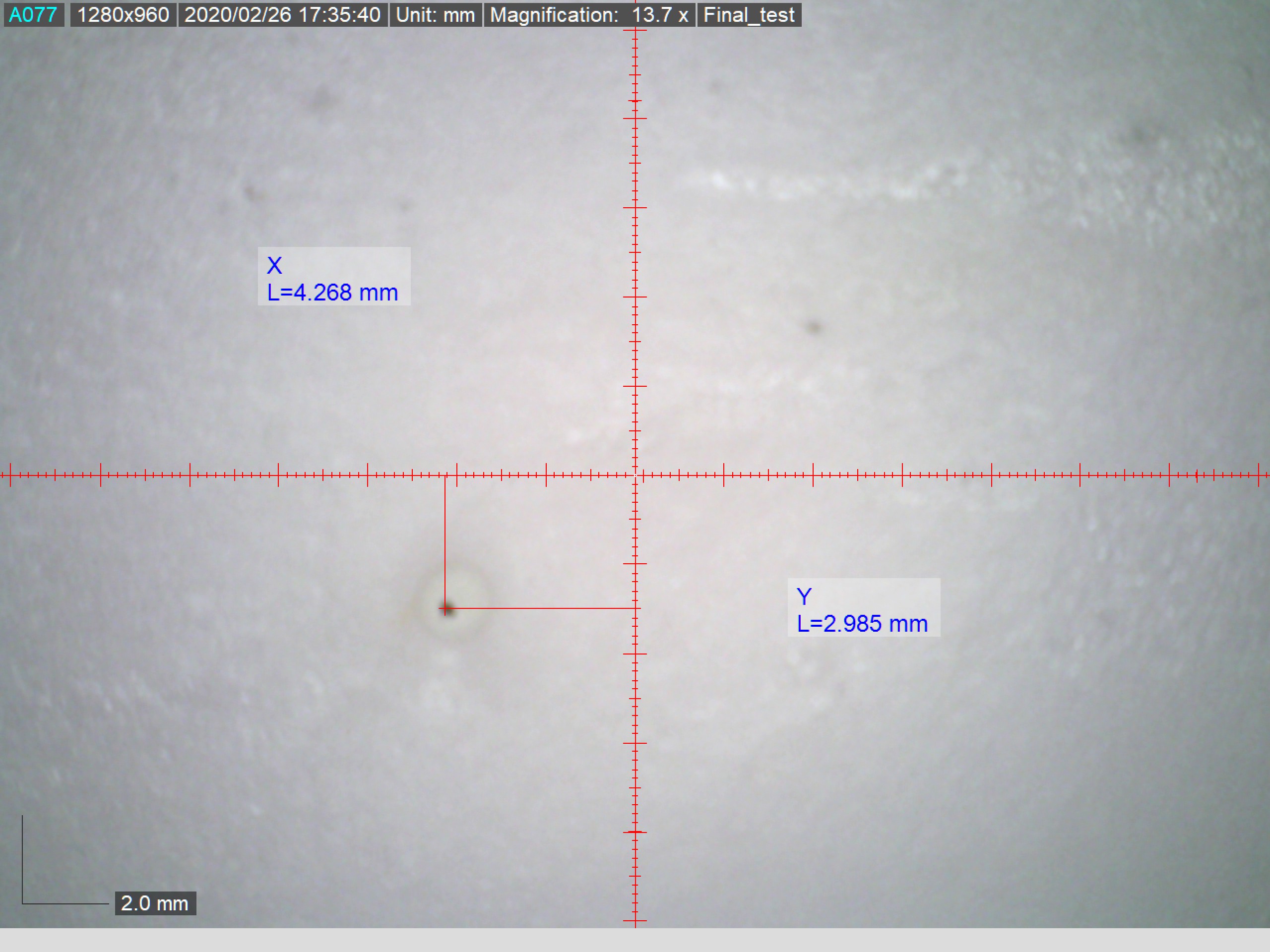

Supplement: S3 File — (ZIP) [file pone.0261089.s003.zip › Stiff phantom/fotos76.jpg]

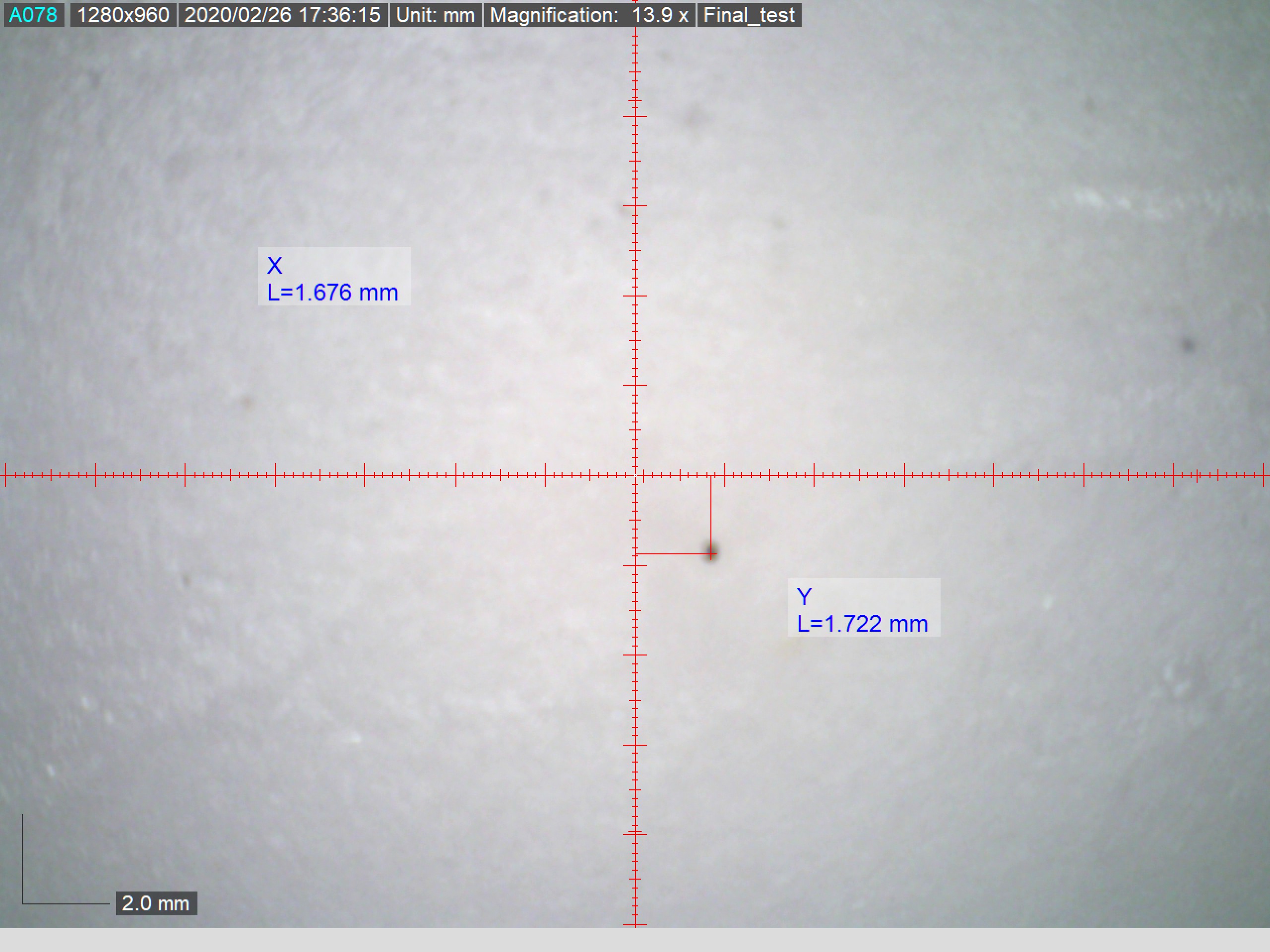

Supplement: S3 File — (ZIP) [file pone.0261089.s003.zip › Stiff phantom/fotos77.jpg]

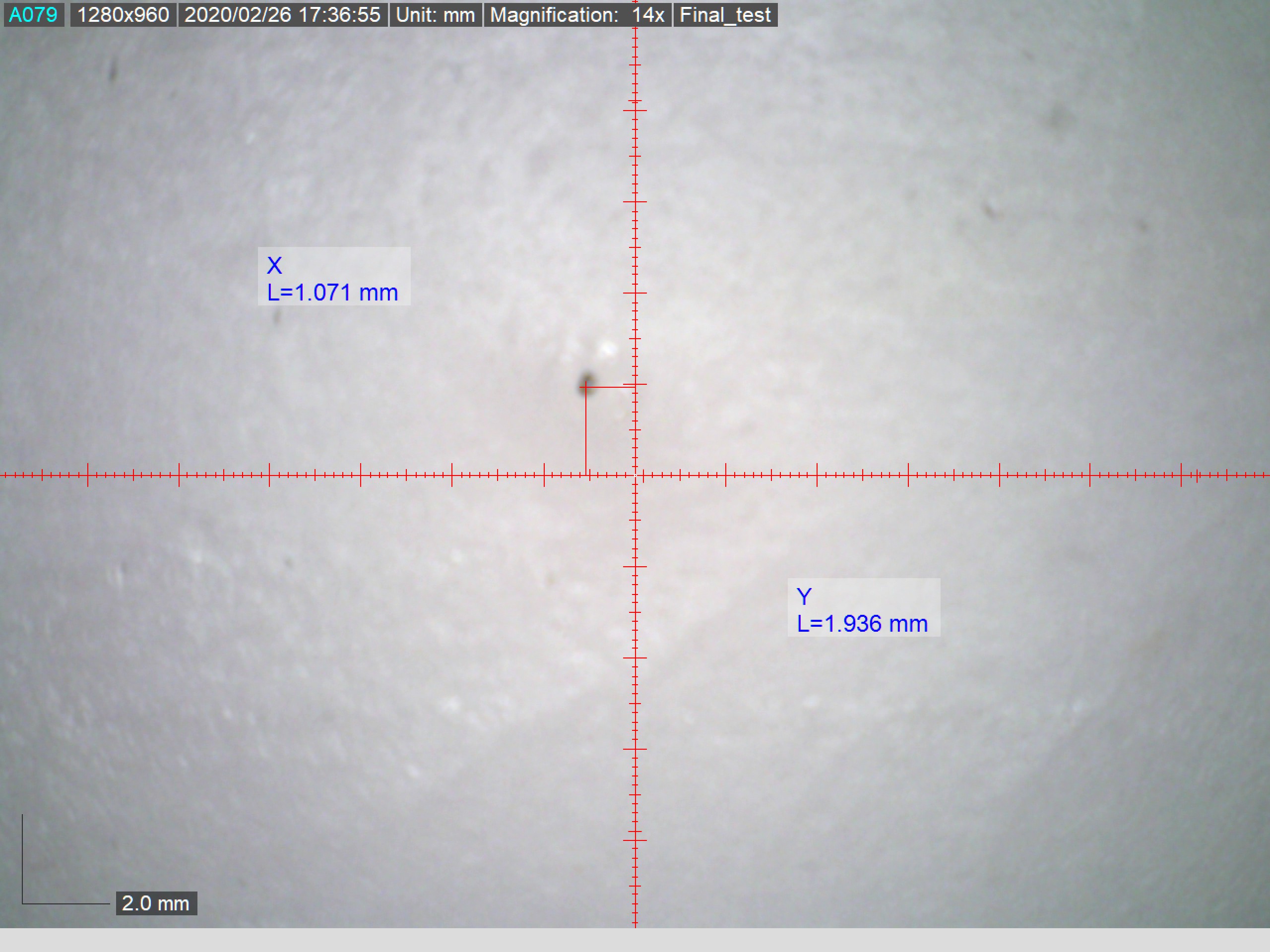

Supplement: S3 File — (ZIP) [file pone.0261089.s003.zip › Stiff phantom/fotos78.jpg]

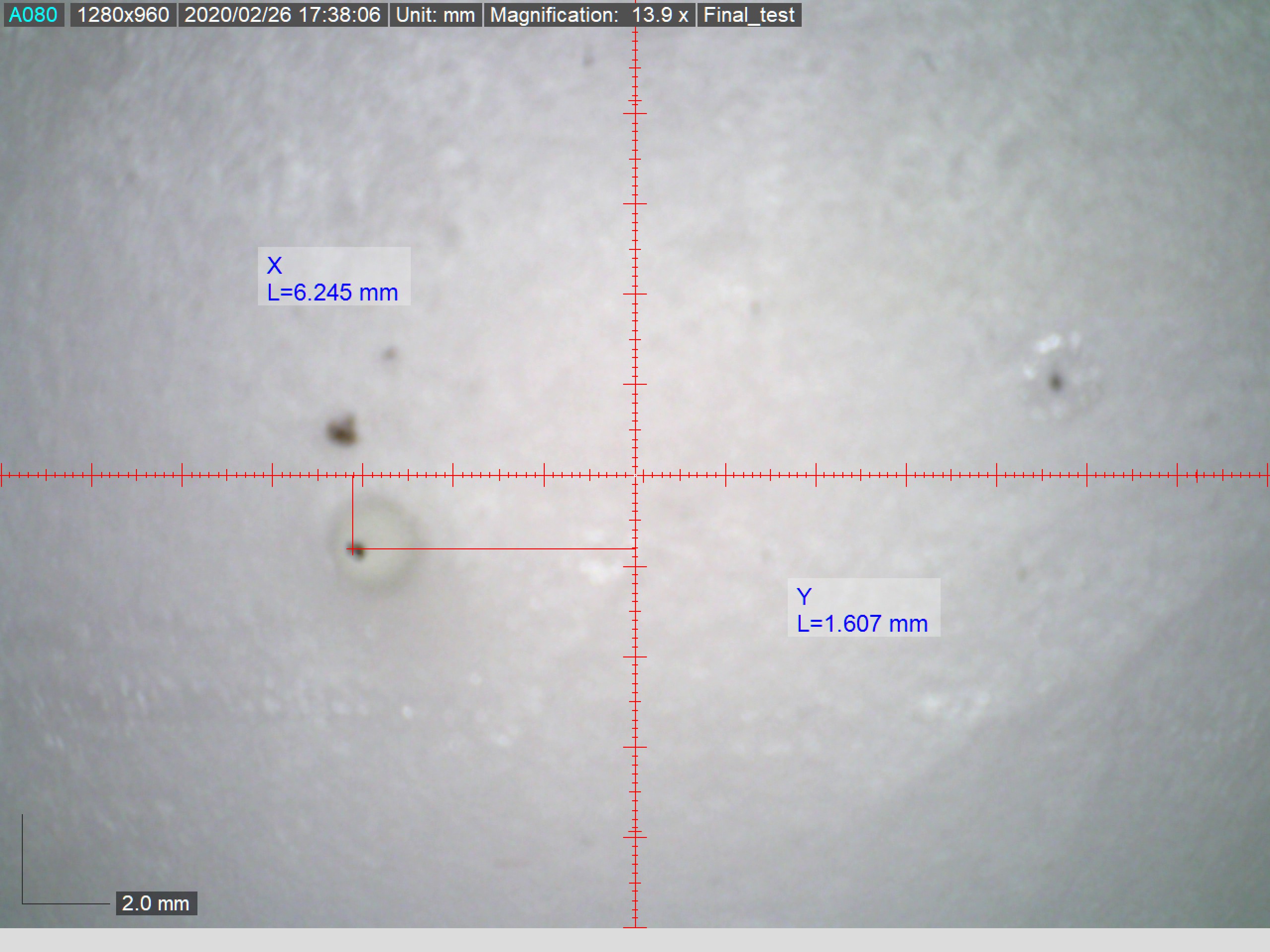

Supplement: S3 File — (ZIP) [file pone.0261089.s003.zip › Stiff phantom/fotos79.jpg]

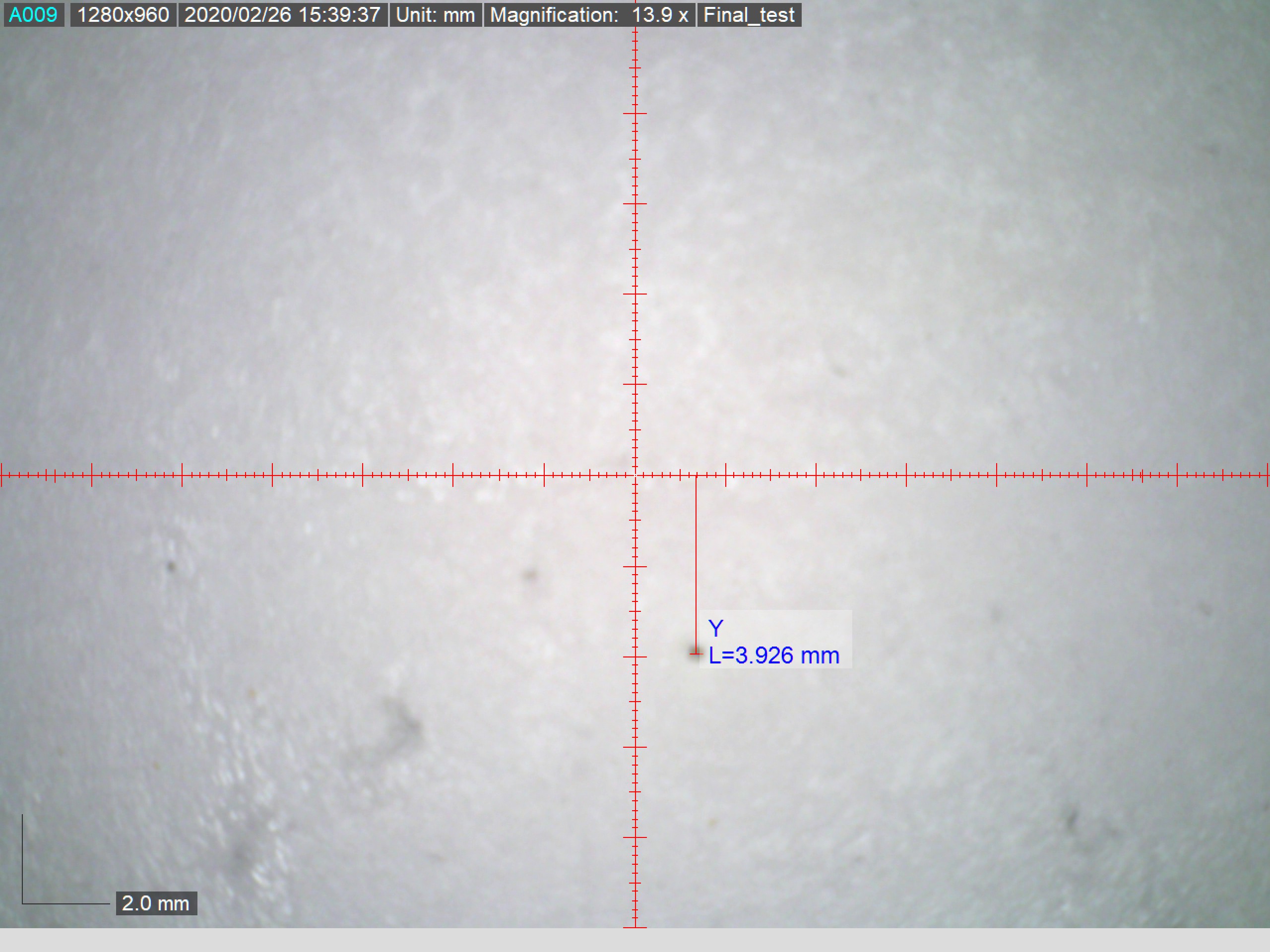

Supplement: S3 File — (ZIP) [file pone.0261089.s003.zip › Stiff phantom/fotos8.jpg]

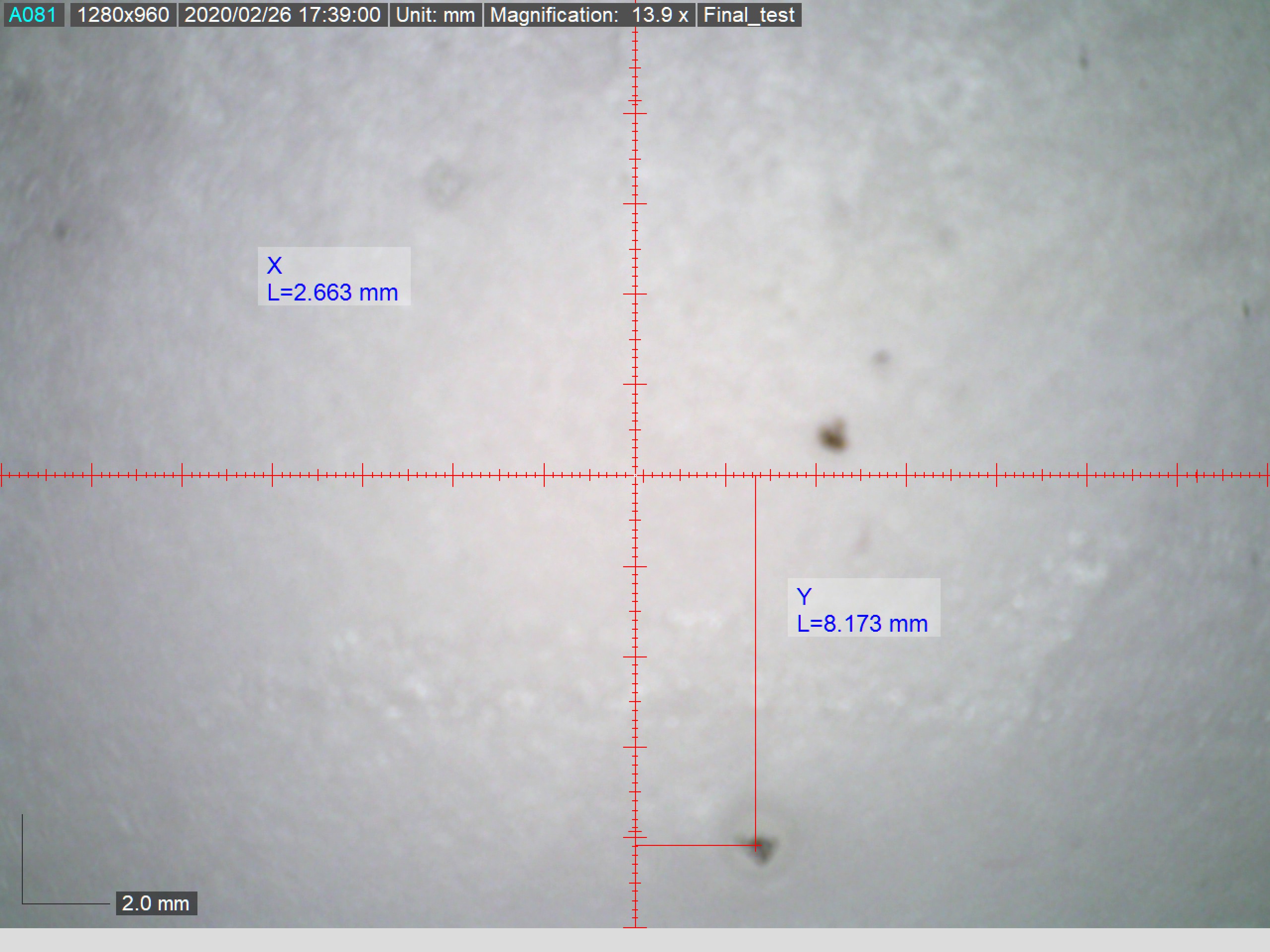

Supplement: S3 File — (ZIP) [file pone.0261089.s003.zip › Stiff phantom/fotos80.jpg]

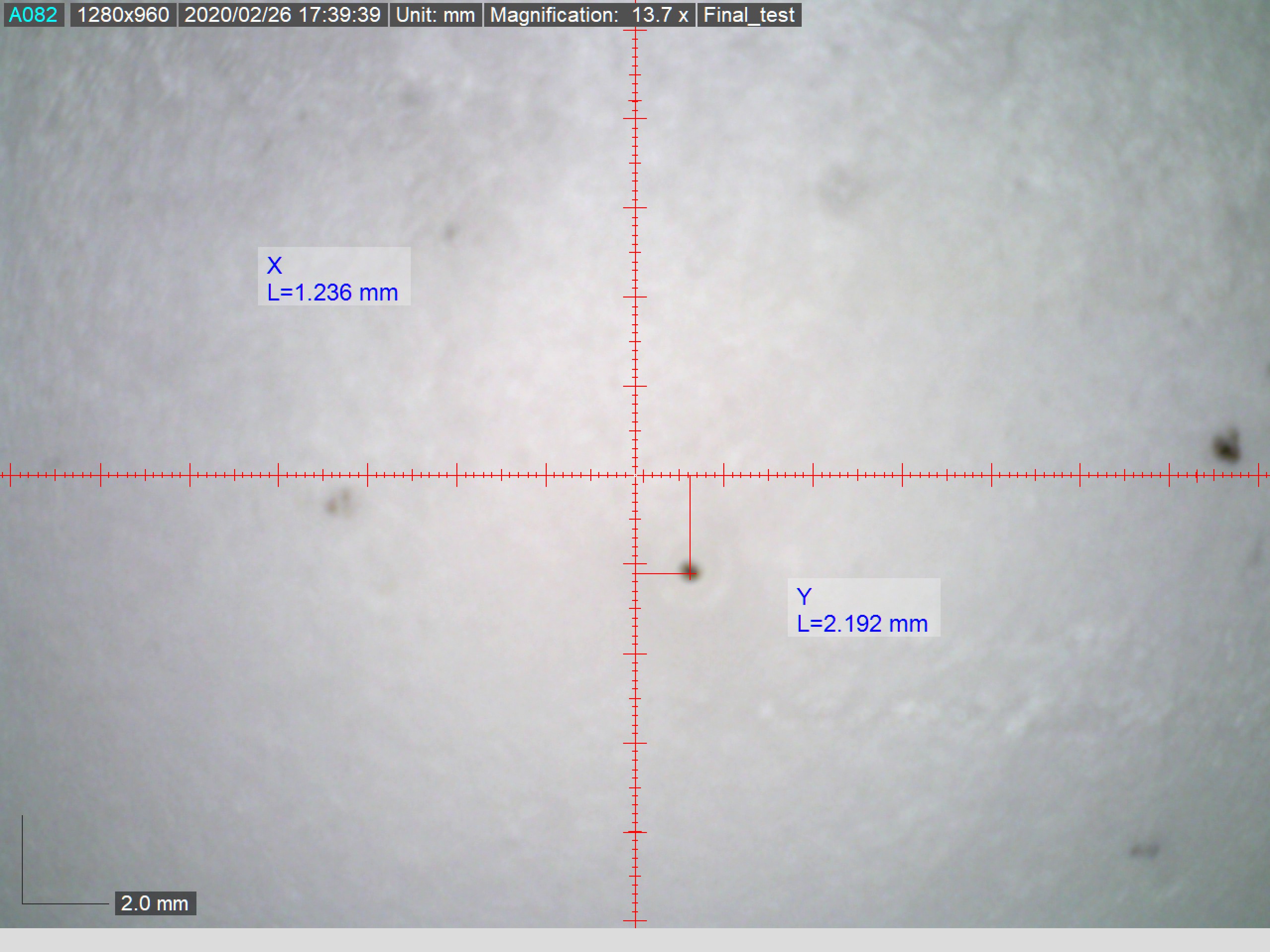

Supplement: S3 File — (ZIP) [file pone.0261089.s003.zip › Stiff phantom/fotos81.jpg]

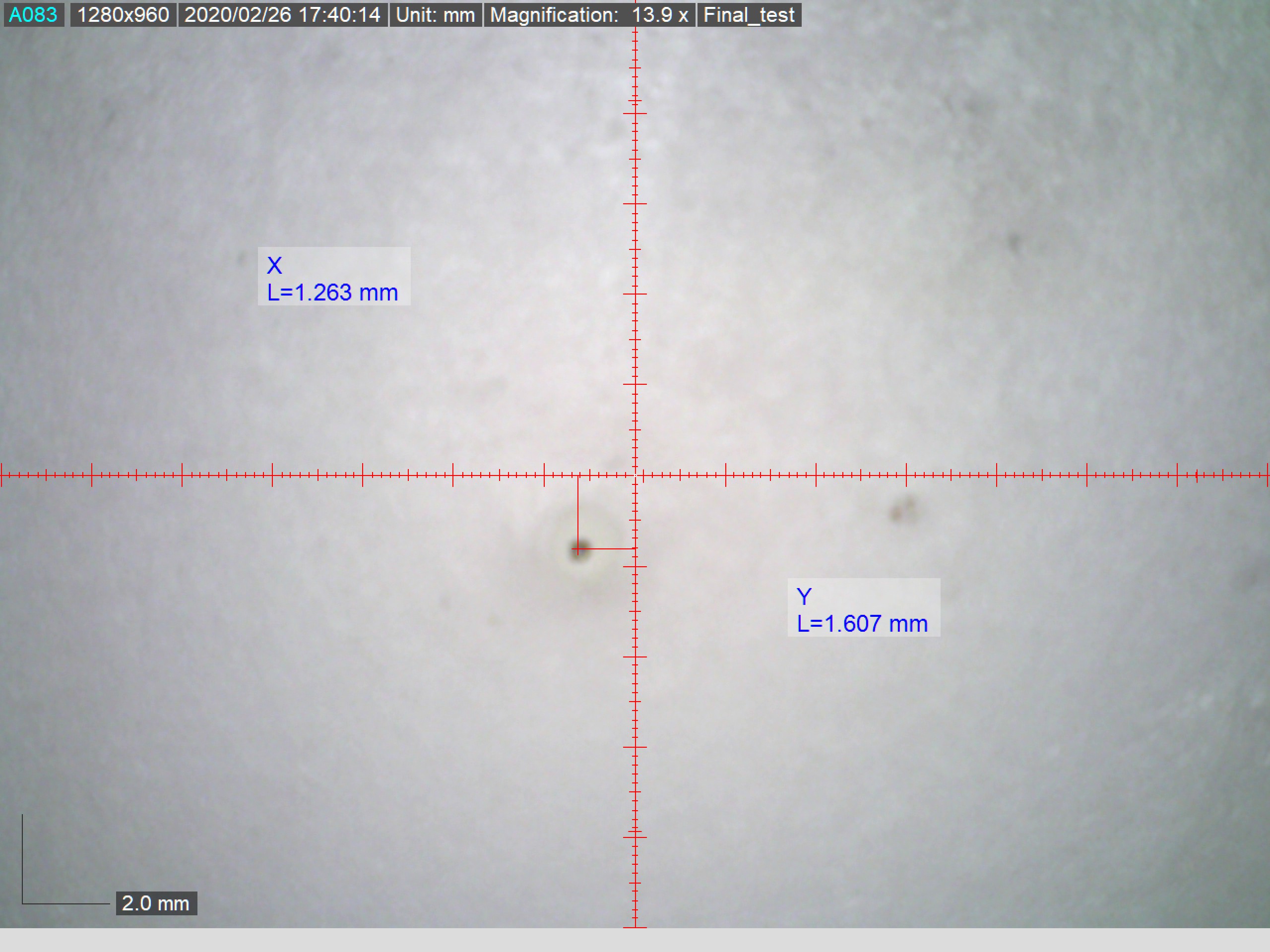

Supplement: S3 File — (ZIP) [file pone.0261089.s003.zip › Stiff phantom/fotos82.jpg]

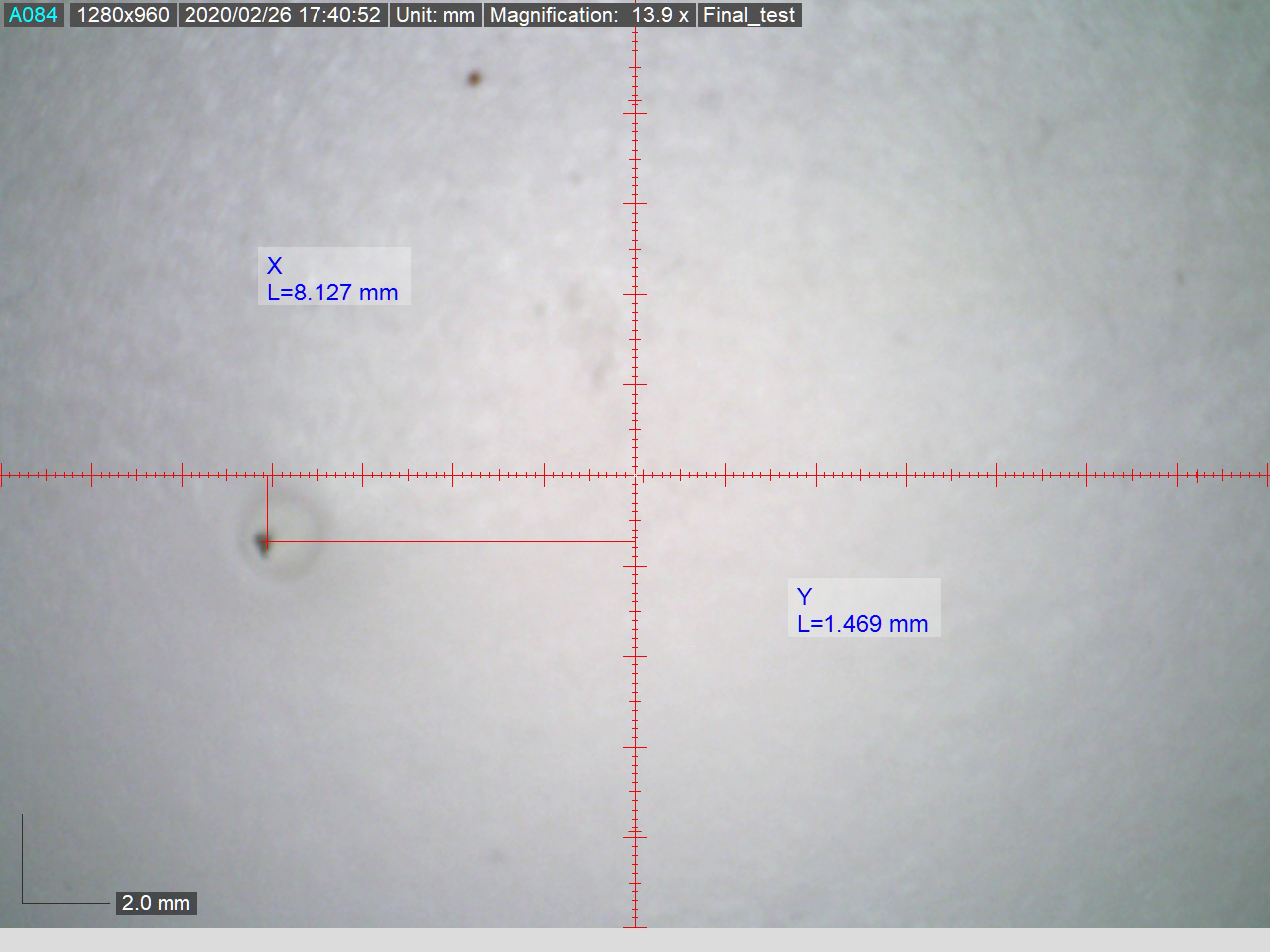

Supplement: S3 File — (ZIP) [file pone.0261089.s003.zip › Stiff phantom/fotos83.jpg]

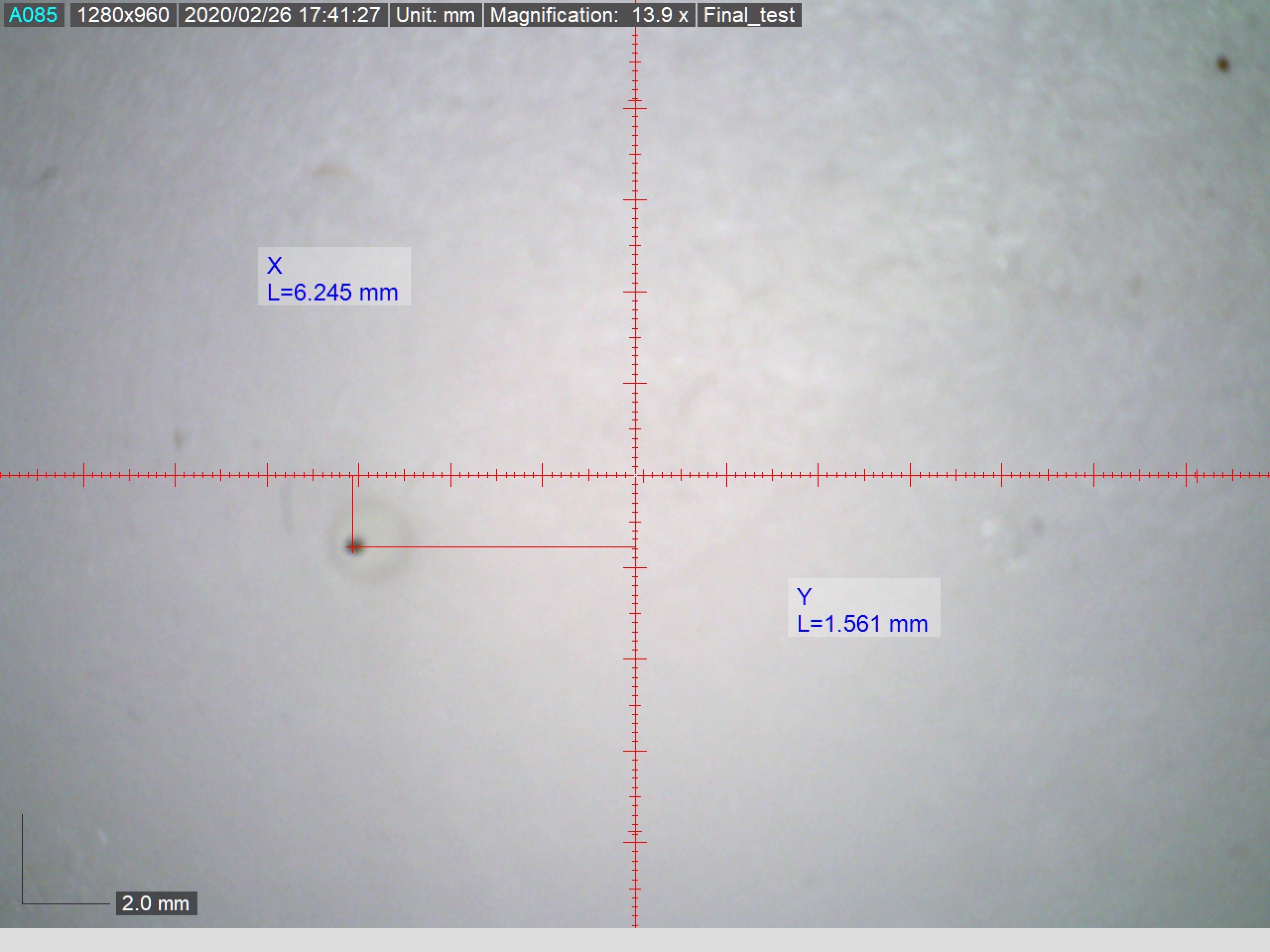

Supplement: S3 File — (ZIP) [file pone.0261089.s003.zip › Stiff phantom/fotos84.jpg]

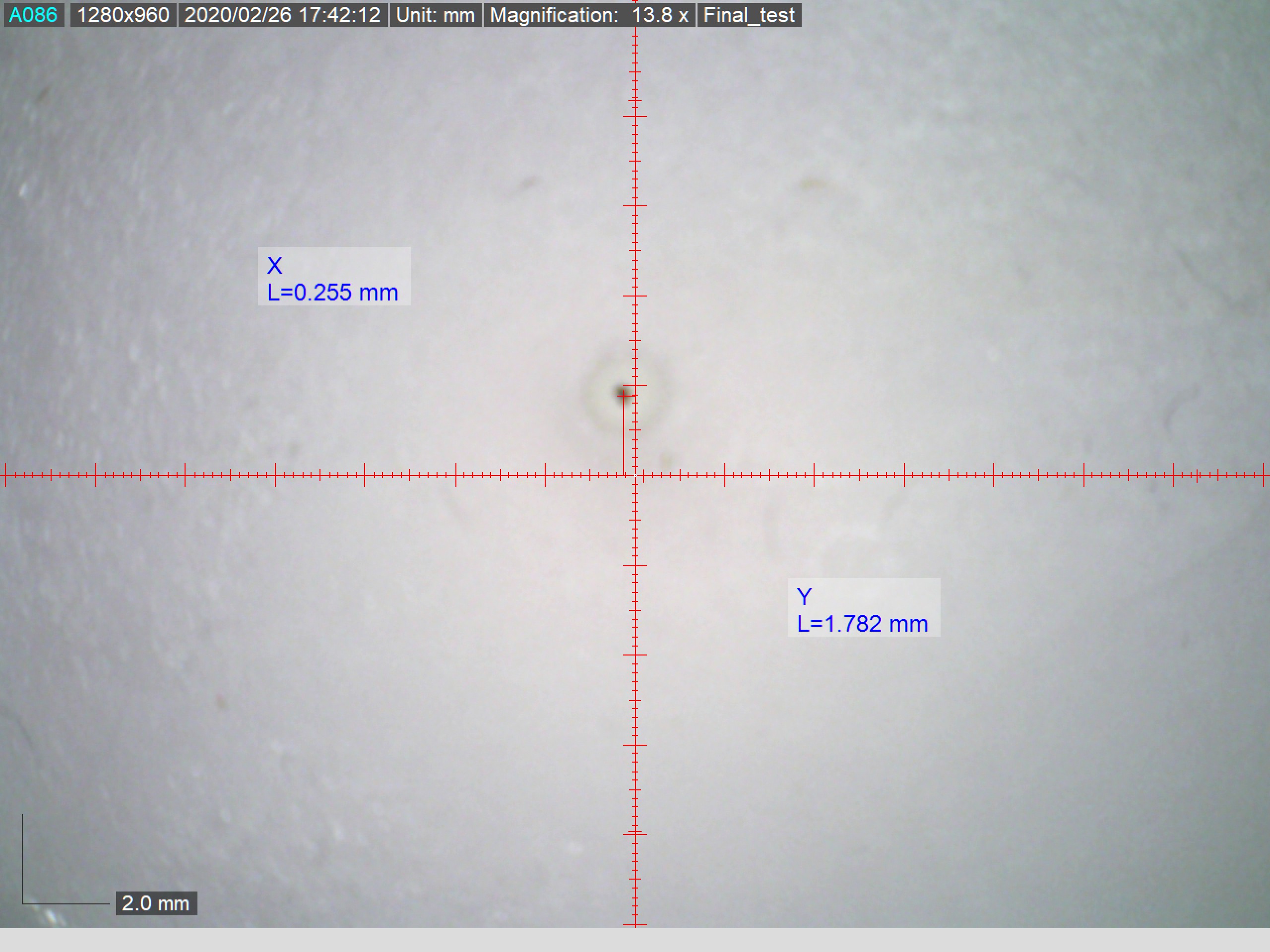

Supplement: S3 File — (ZIP) [file pone.0261089.s003.zip › Stiff phantom/fotos85.jpg]

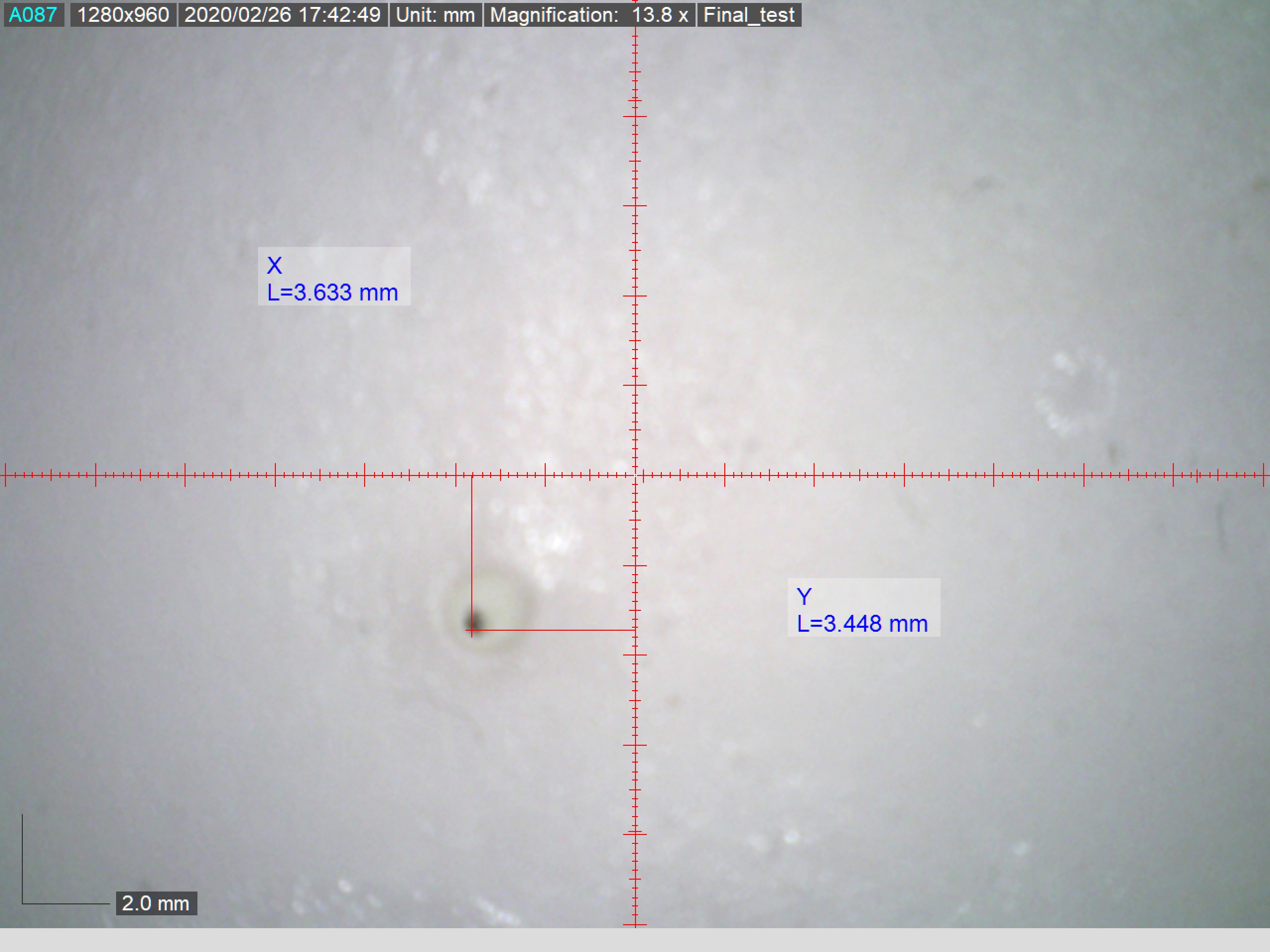

Supplement: S3 File — (ZIP) [file pone.0261089.s003.zip › Stiff phantom/fotos86.jpg]

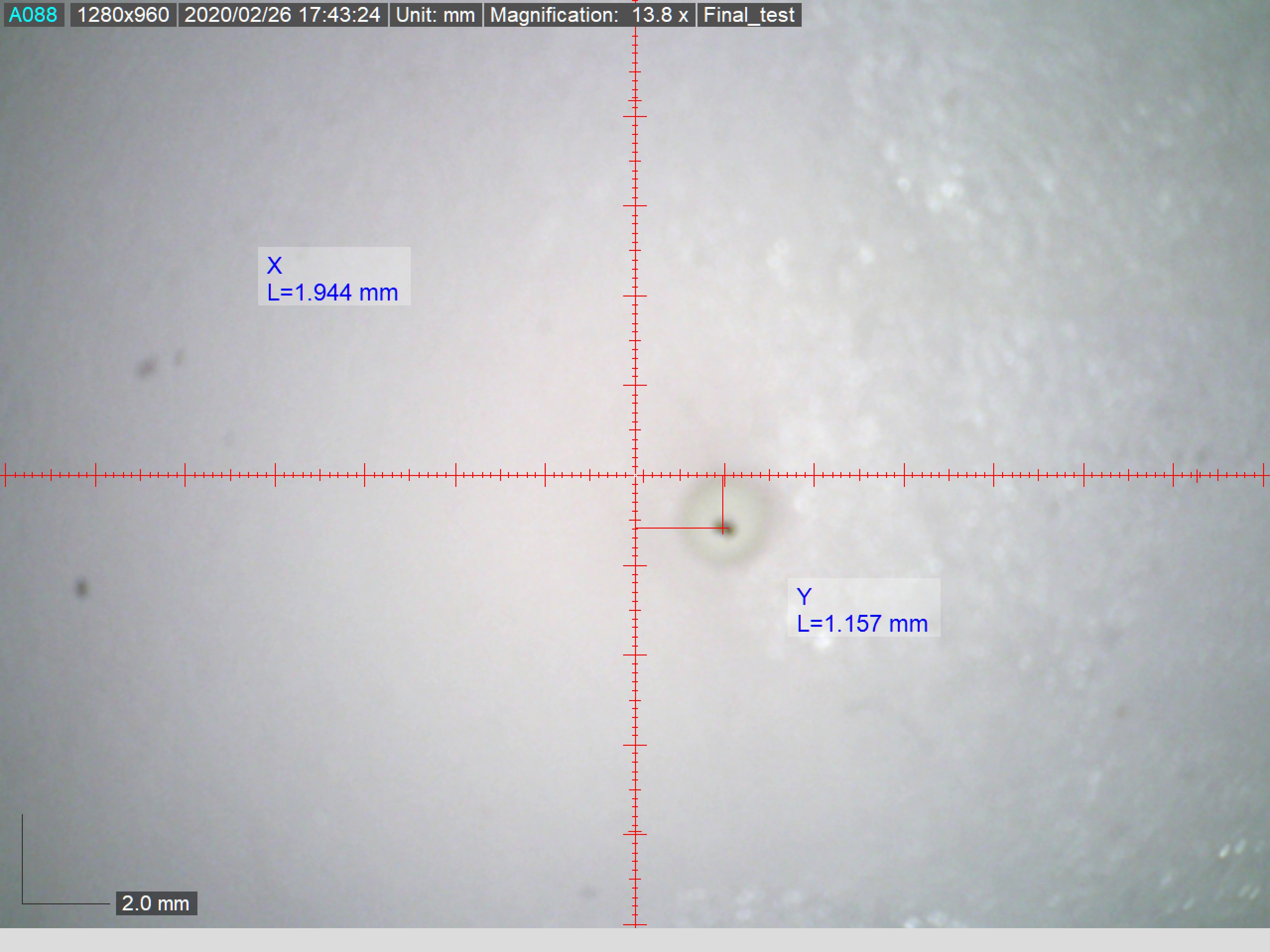

Supplement: S3 File — (ZIP) [file pone.0261089.s003.zip › Stiff phantom/fotos87.jpg]

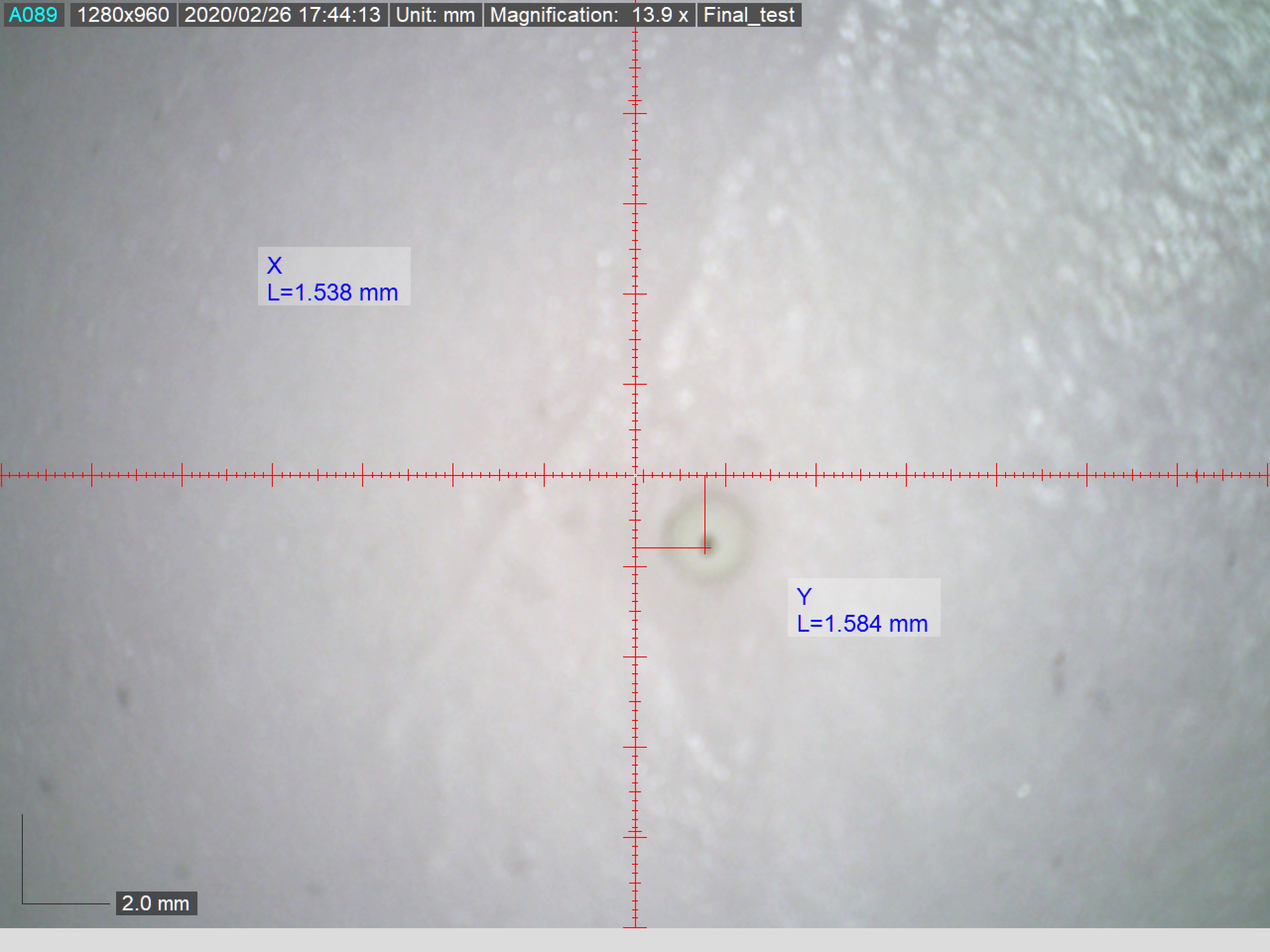

Supplement: S3 File — (ZIP) [file pone.0261089.s003.zip › Stiff phantom/fotos88.jpg]

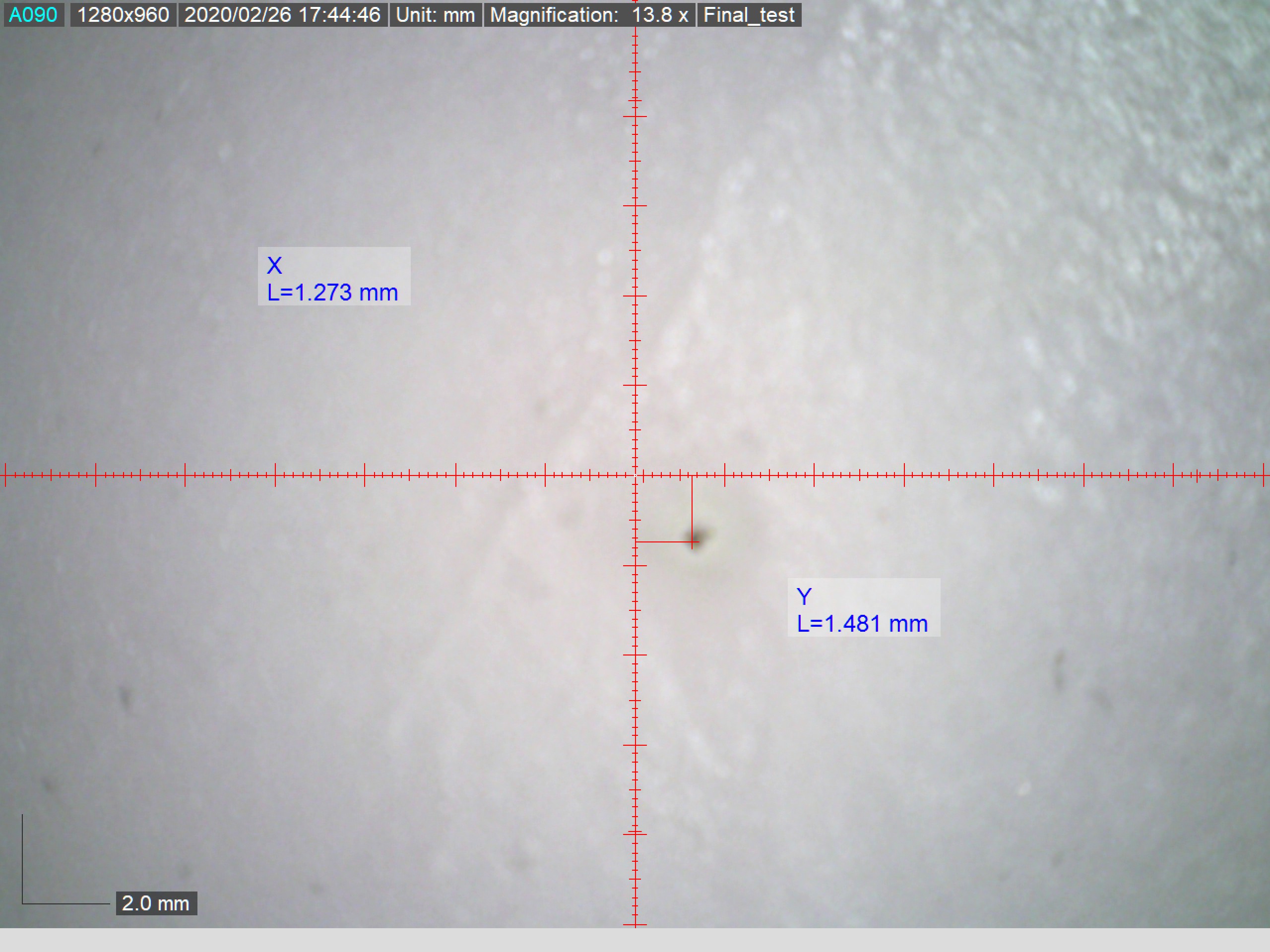

Supplement: S3 File — (ZIP) [file pone.0261089.s003.zip › Stiff phantom/fotos89.jpg]

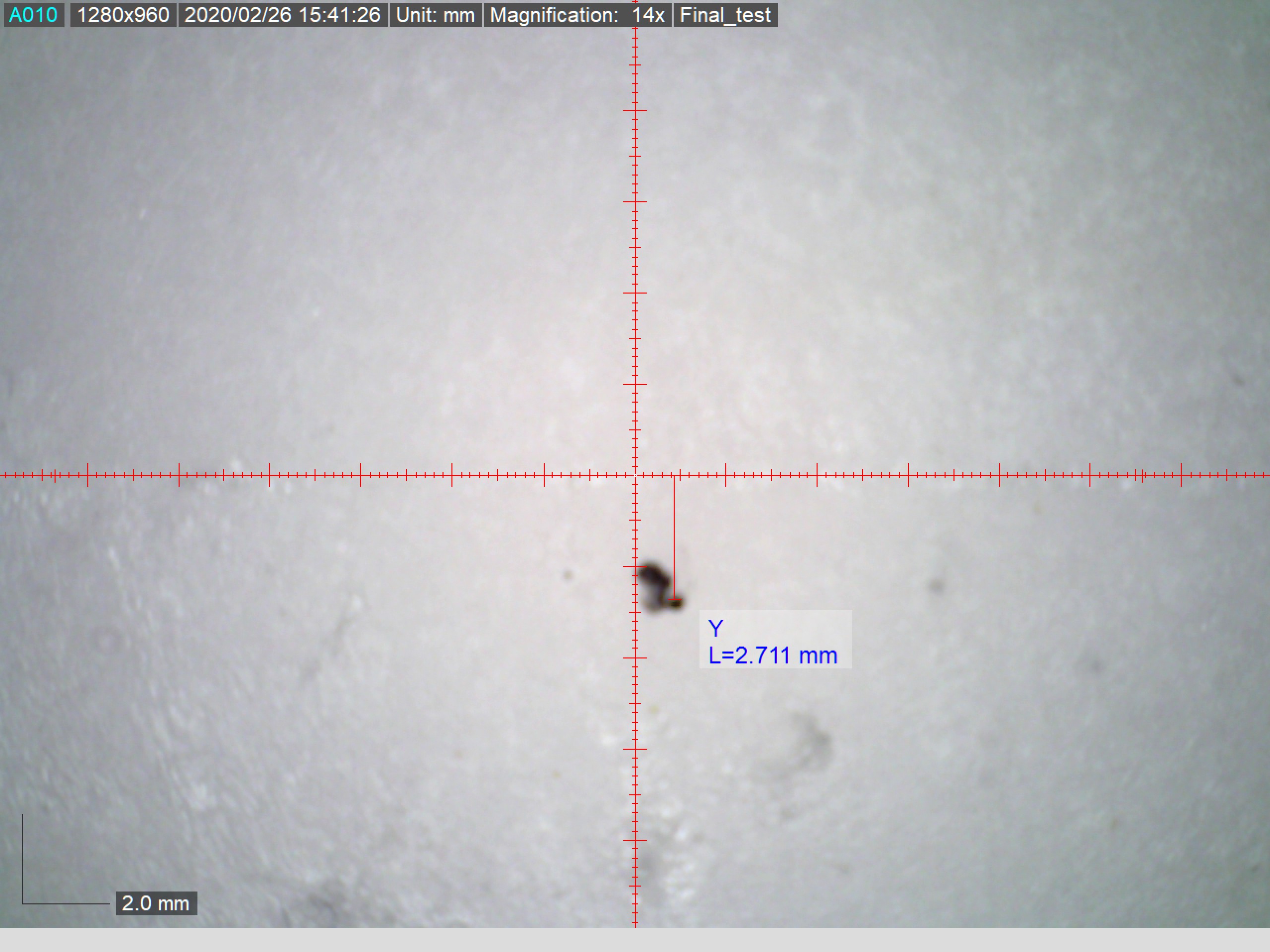

Supplement: S3 File — (ZIP) [file pone.0261089.s003.zip › Stiff phantom/fotos9.jpg]

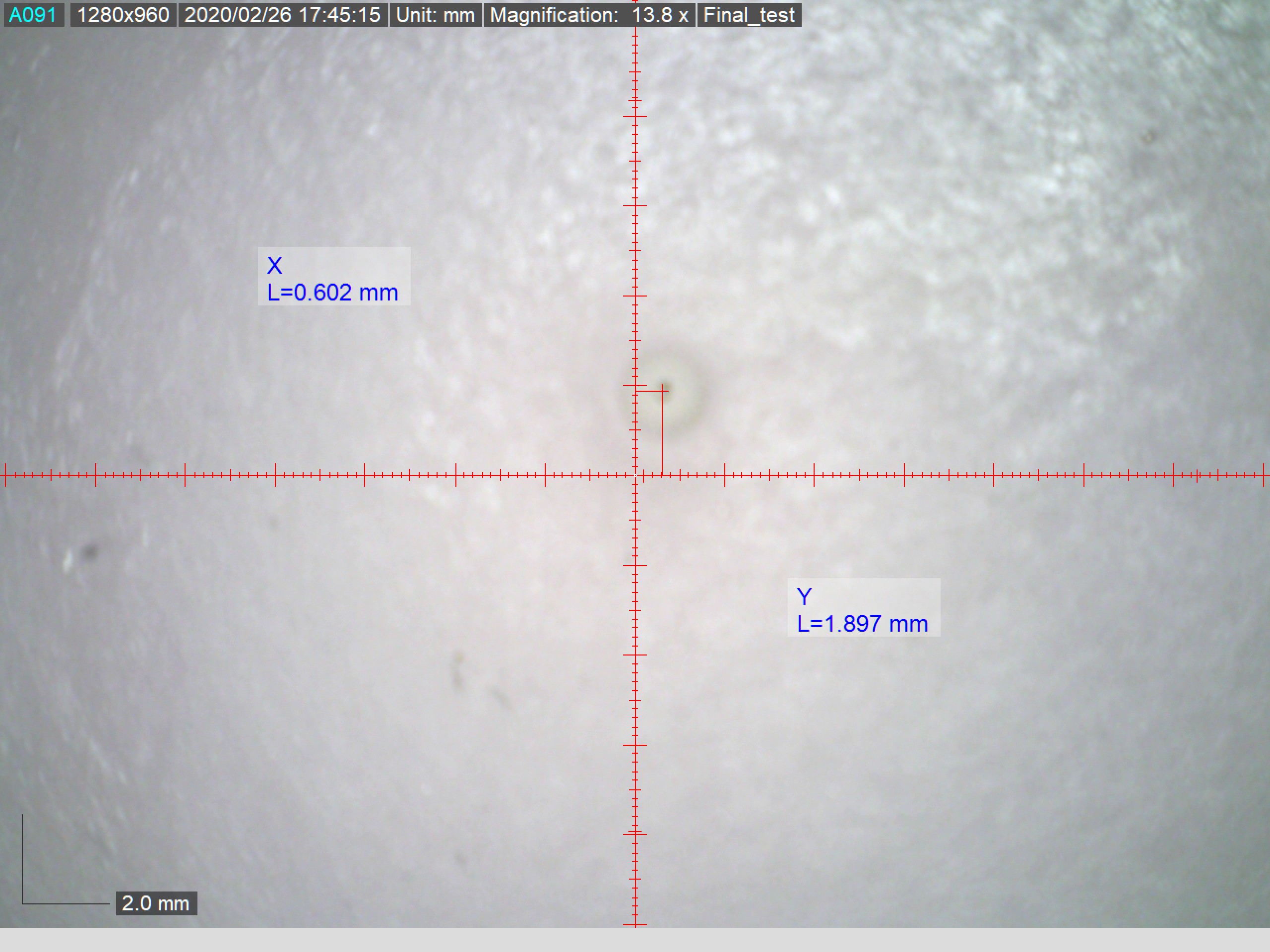

Supplement: S3 File — (ZIP) [file pone.0261089.s003.zip › Stiff phantom/fotos90.jpg]

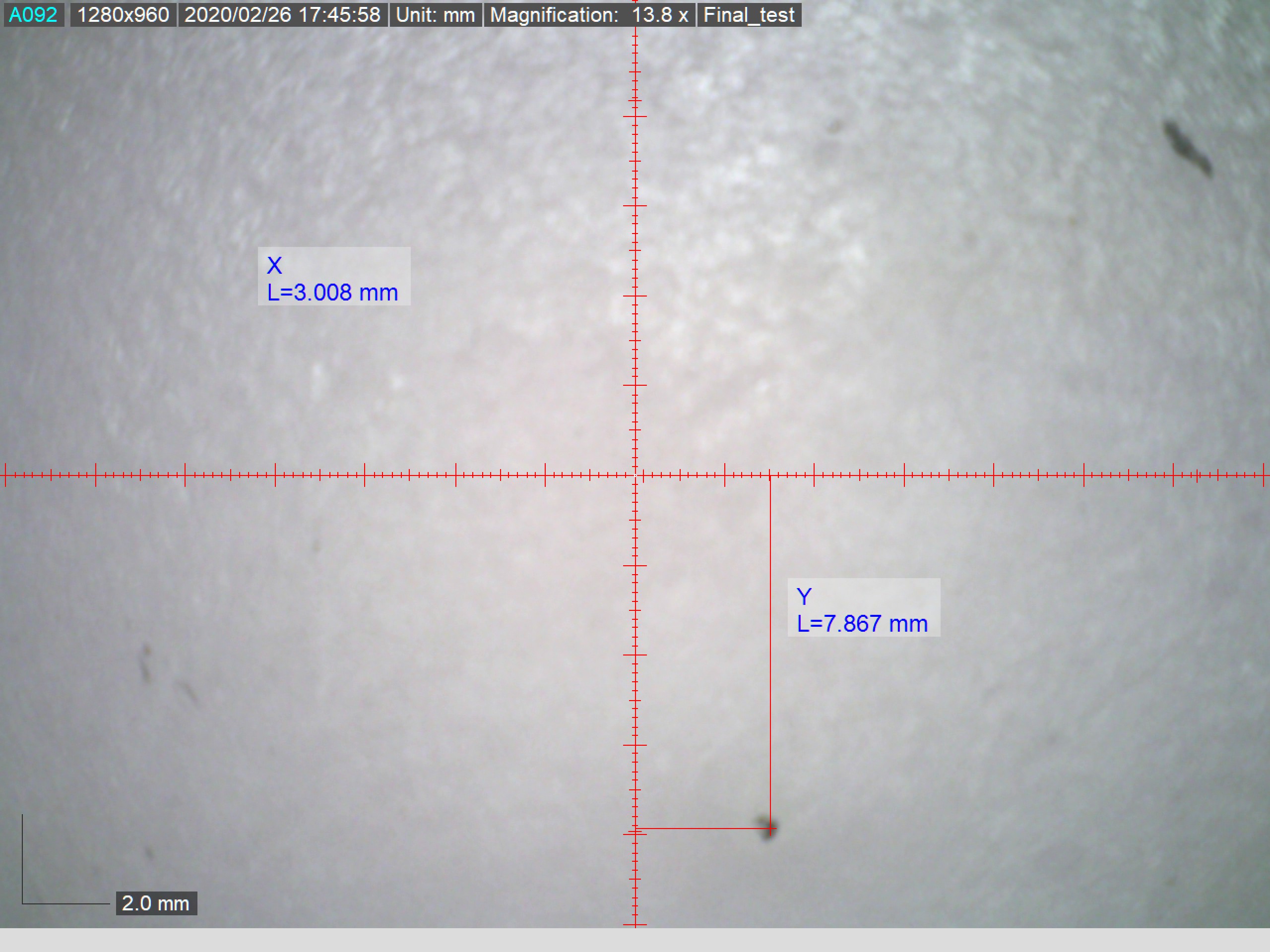

Supplement: S3 File — (ZIP) [file pone.0261089.s003.zip › Stiff phantom/fotos91.jpg]

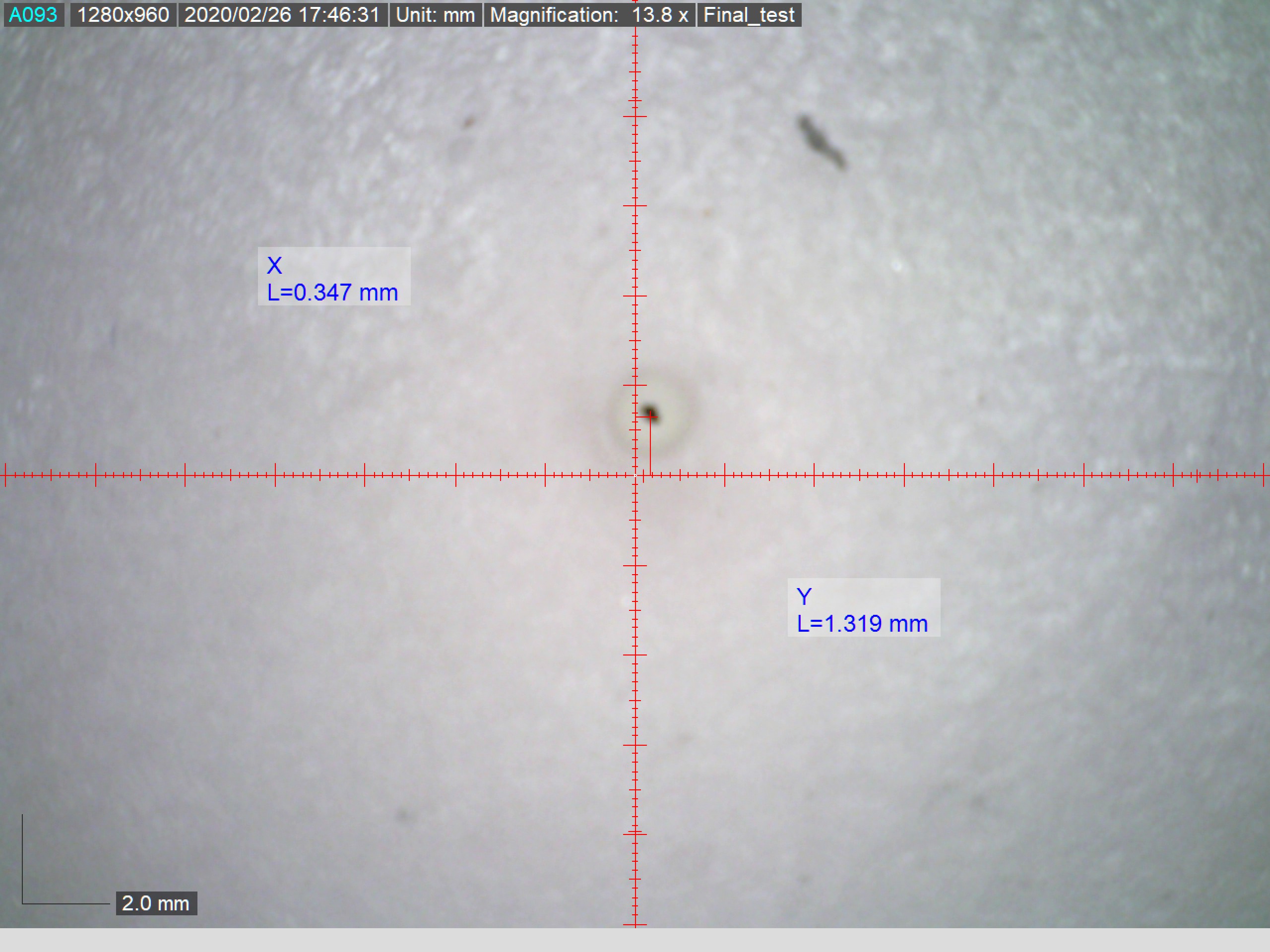

Supplement: S3 File — (ZIP) [file pone.0261089.s003.zip › Stiff phantom/fotos92.jpg]

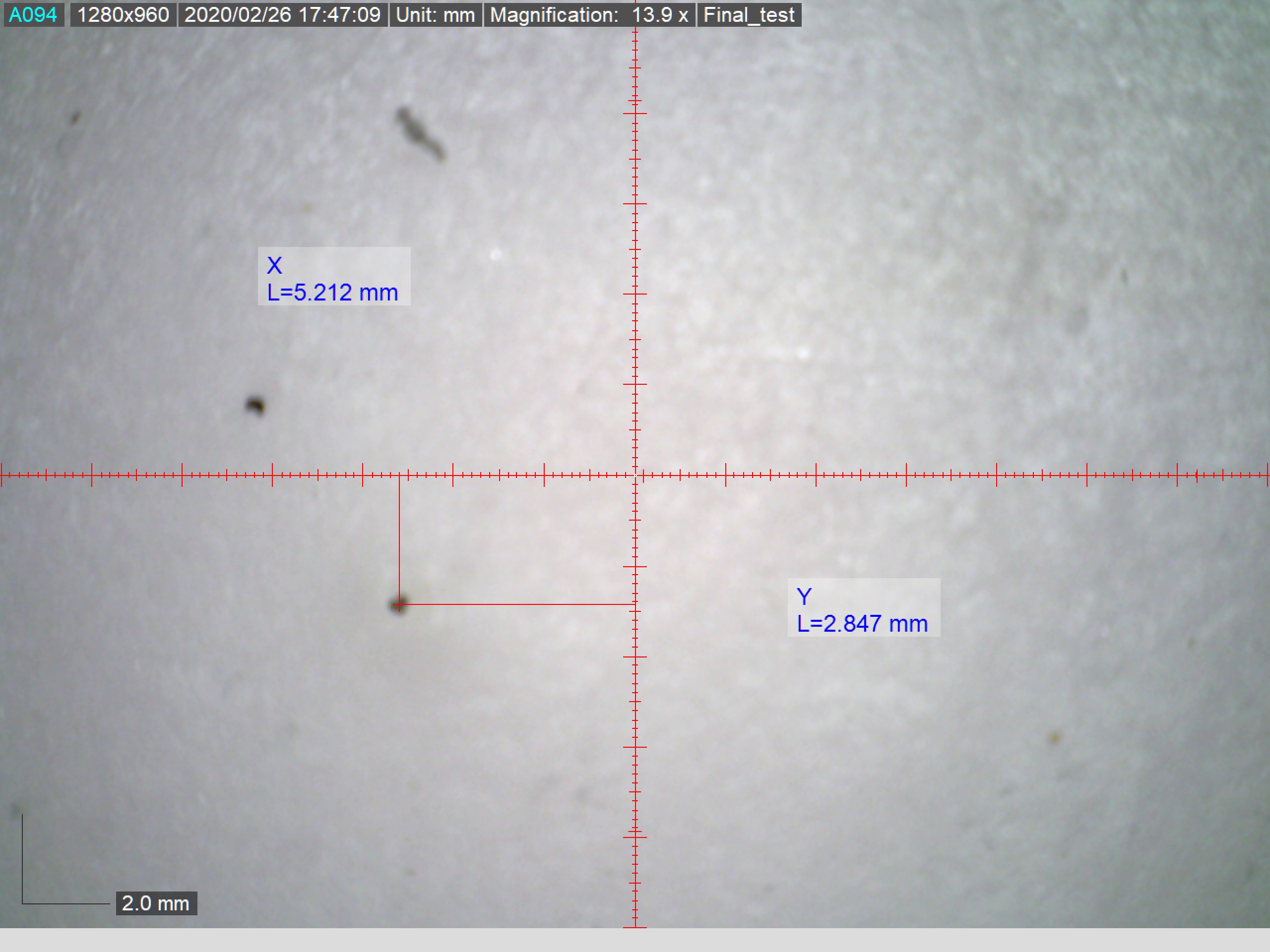

Supplement: S3 File — (ZIP) [file pone.0261089.s003.zip › Stiff phantom/fotos93.jpg]

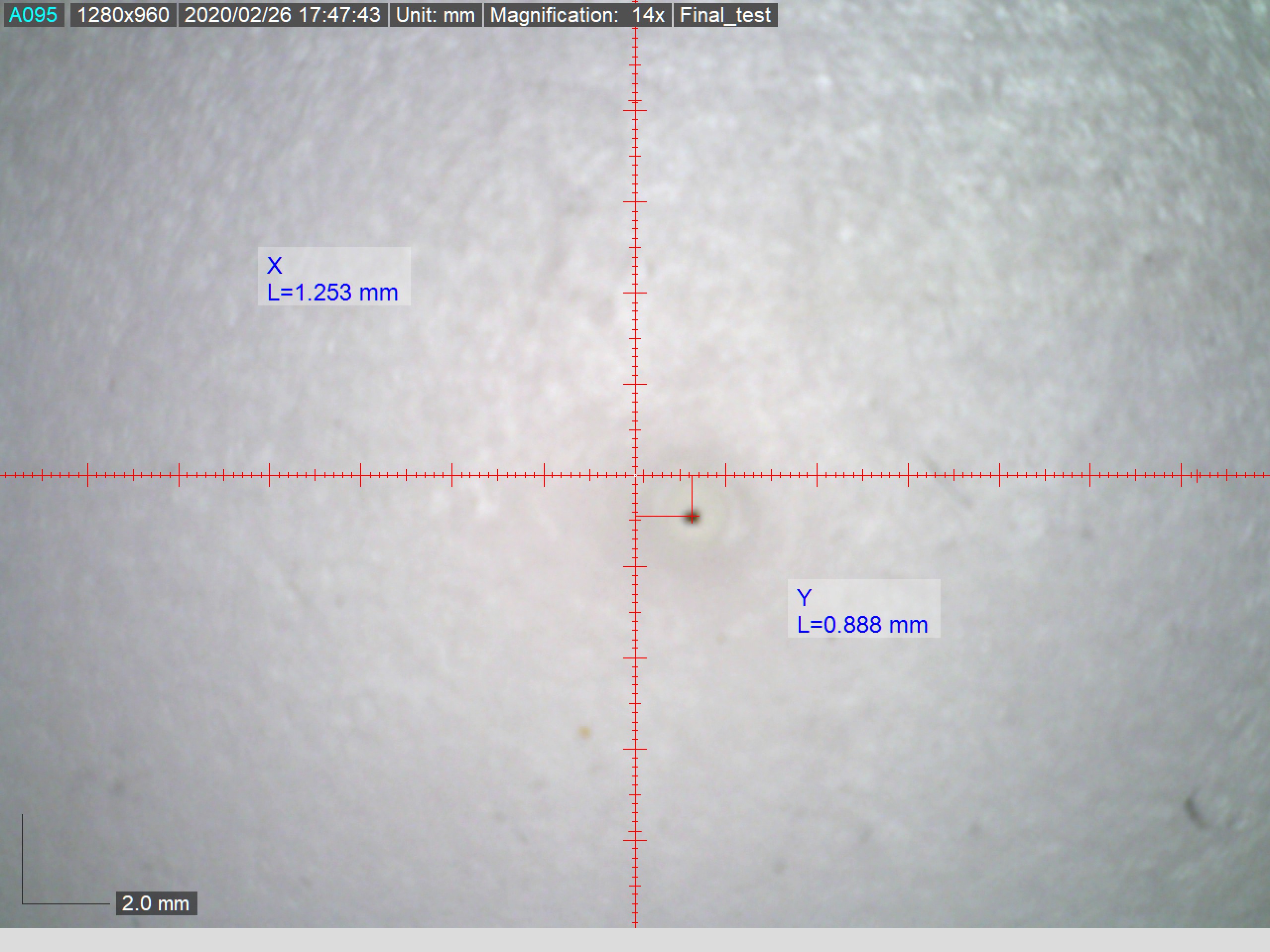

Supplement: S3 File — (ZIP) [file pone.0261089.s003.zip › Stiff phantom/fotos94.jpg]

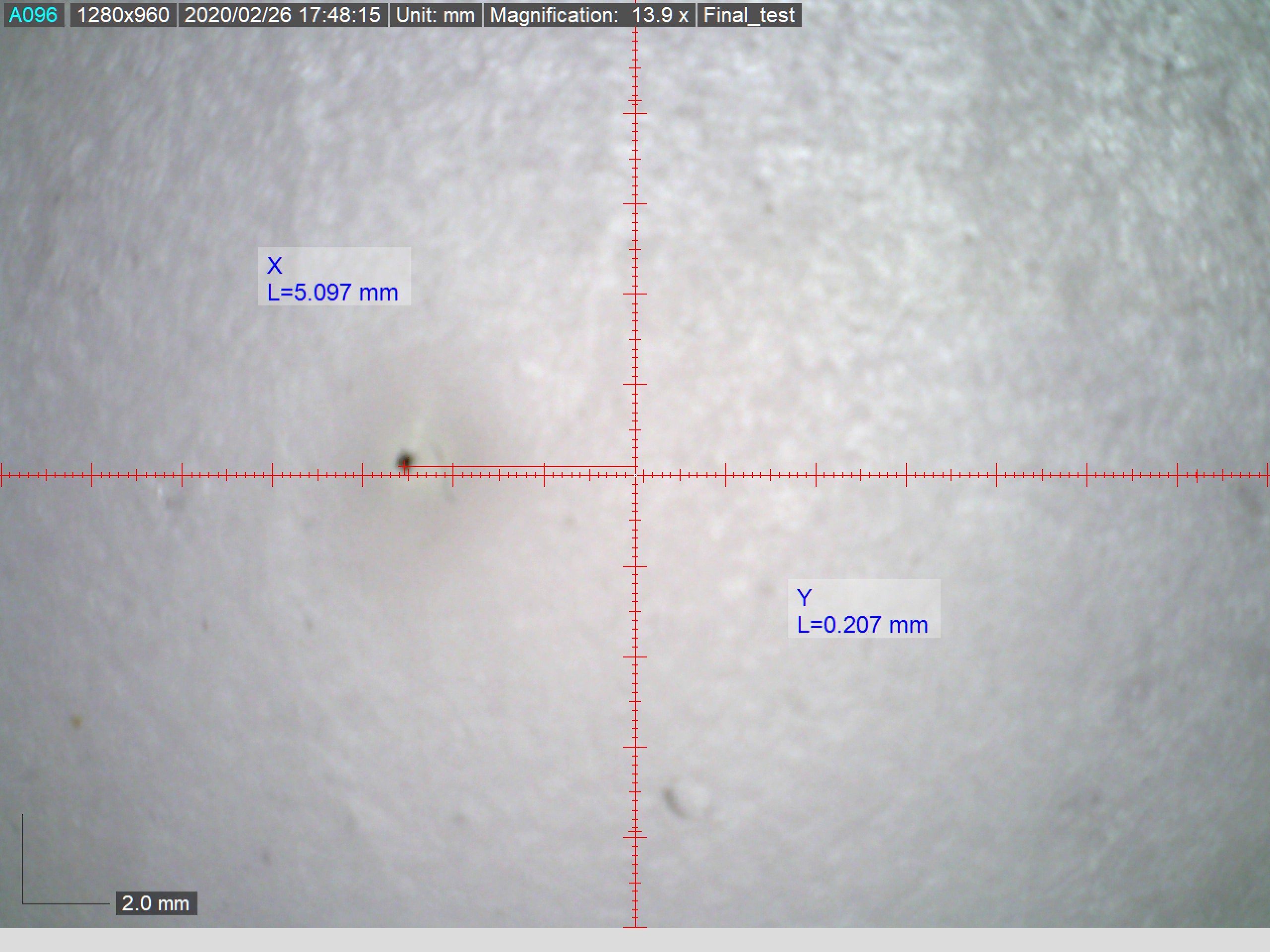

Supplement: S3 File — (ZIP) [file pone.0261089.s003.zip › Stiff phantom/fotos95.jpg]

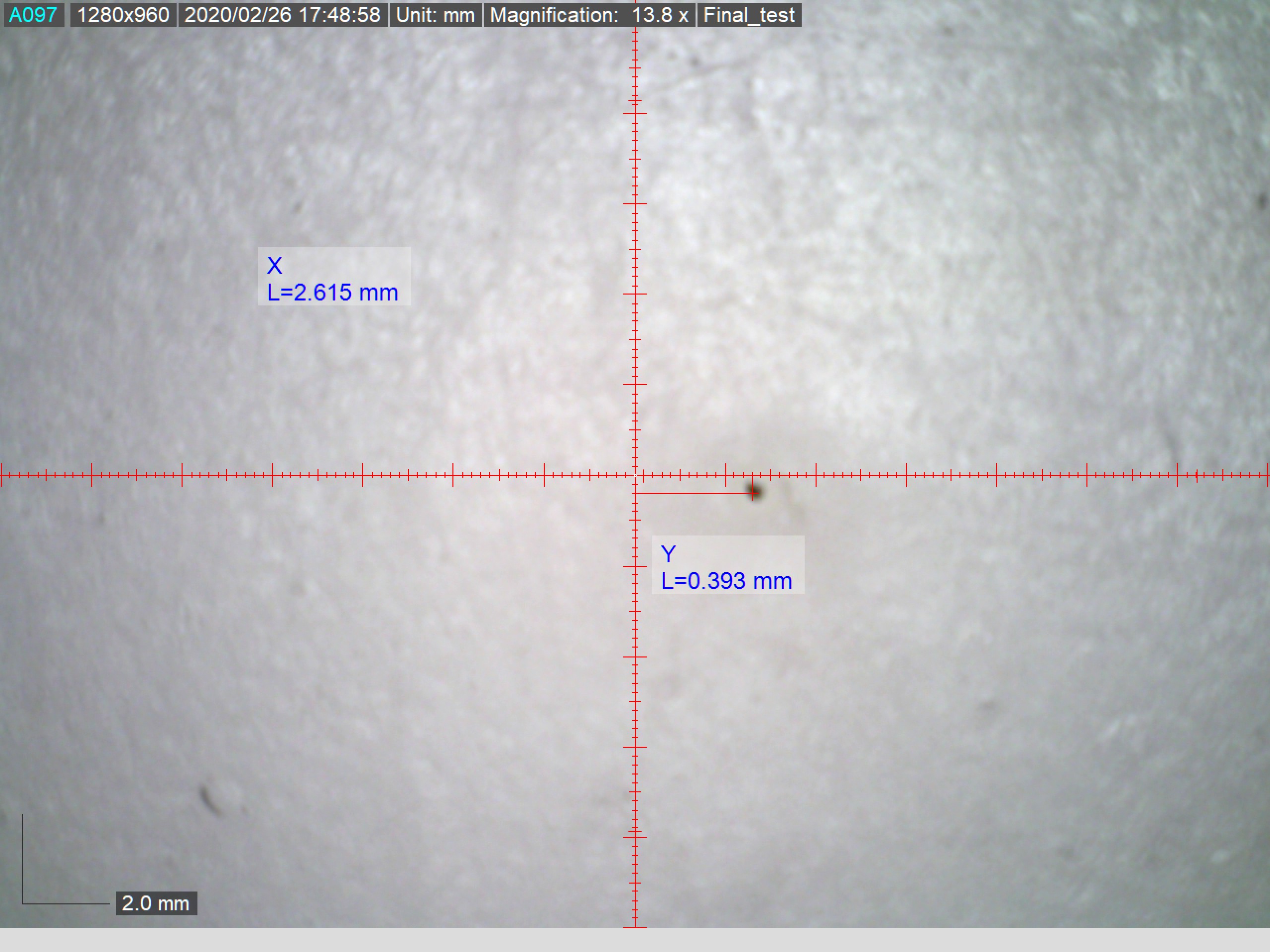

Supplement: S3 File — (ZIP) [file pone.0261089.s003.zip › Stiff phantom/fotos96.jpg]

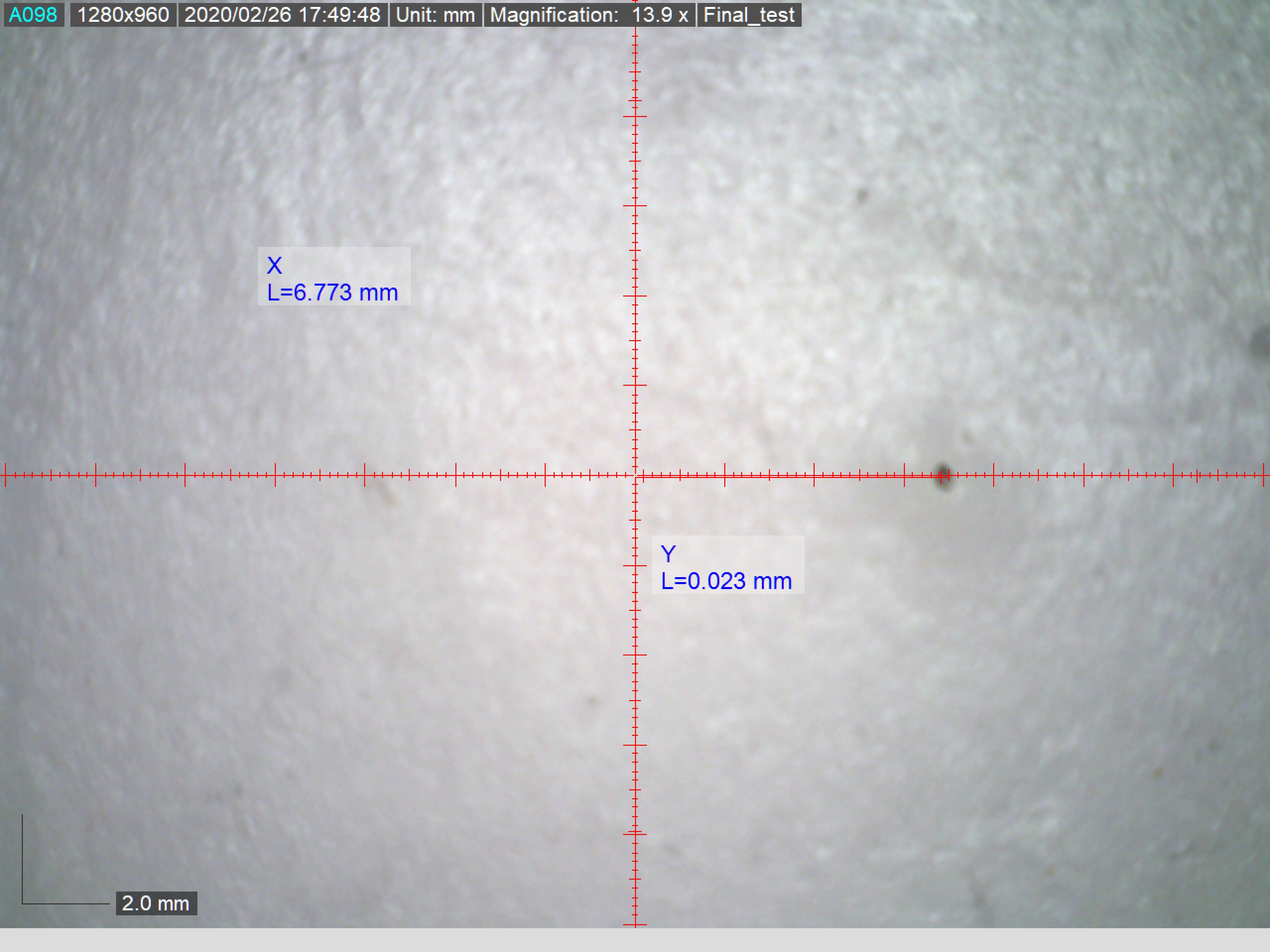

Supplement: S3 File — (ZIP) [file pone.0261089.s003.zip › Stiff phantom/fotos97.jpg]

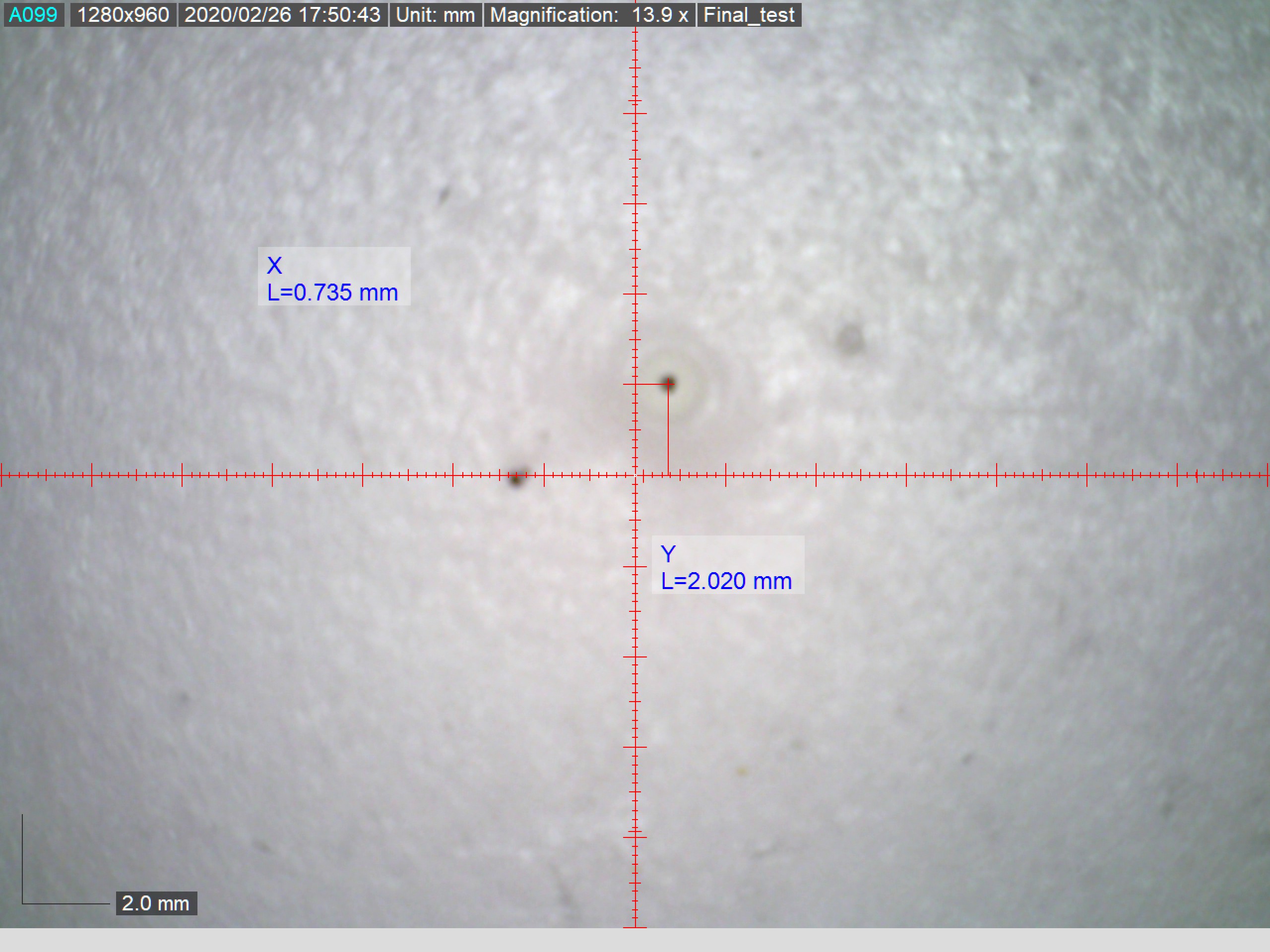

Supplement: S3 File — (ZIP) [file pone.0261089.s003.zip › Stiff phantom/fotos98.jpg]

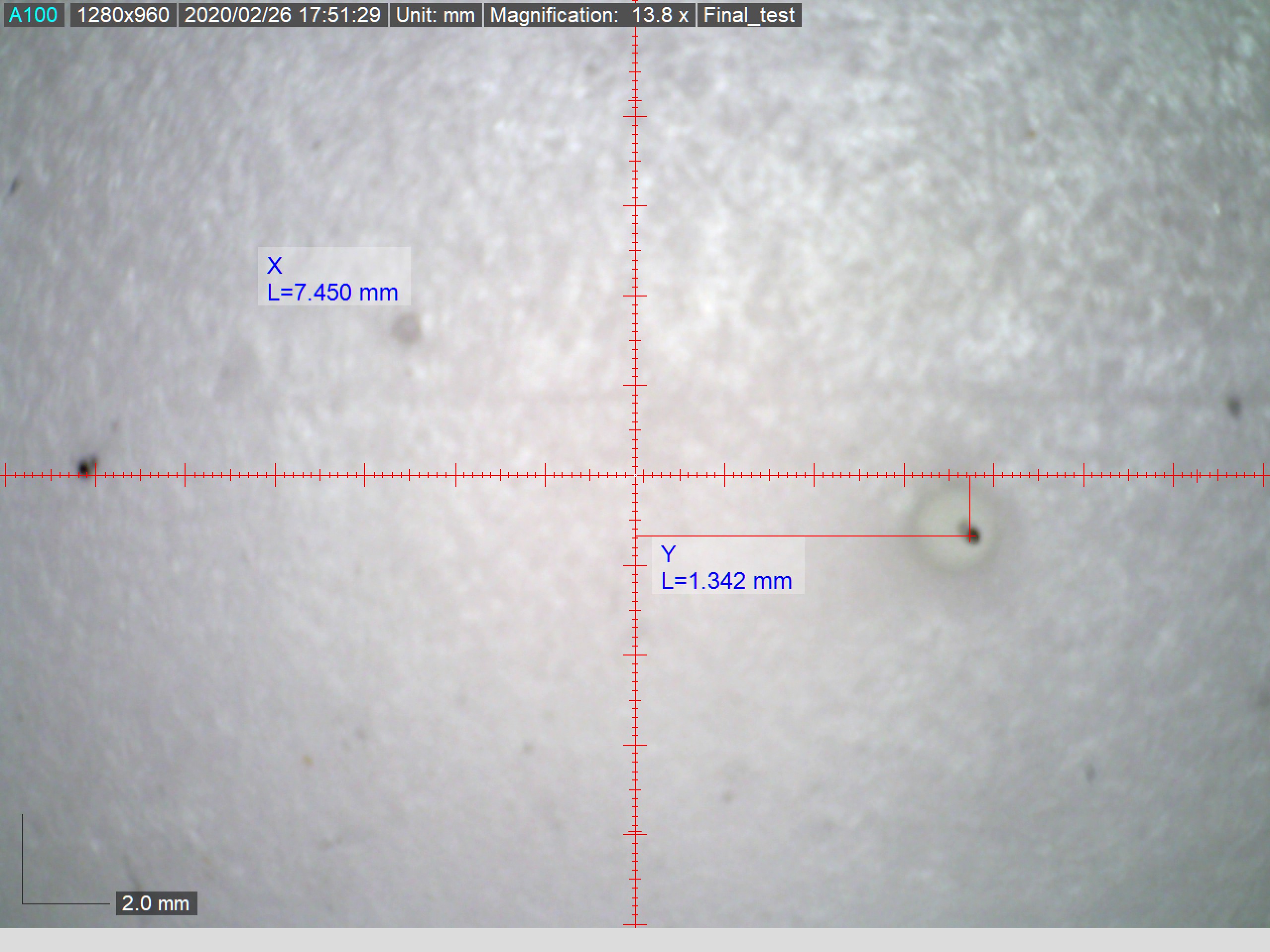

Supplement: S3 File — (ZIP) [file pone.0261089.s003.zip › Stiff phantom/fotos99.jpg]
